# A protocol for controlled reactivity shift in the 2,2-difluorovinyl motif used for selective S–^18^F and C–^18^F bond formation

**DOI:** 10.1038/s42004-024-01132-3

**Published:** 2024-04-29

**Authors:** Mudasir Maqbool, Jimmy Erik Jakobsson, Santosh Reddy Alluri, Vasko Kramer, Patrick Johannes Riss

**Affiliations:** 1 Department of Clinical Neurocience, OUS-Ullevål, Oslo, Norway; 2 Department of Chemistry, University of Oslo, Oslo, Norway; 3 Positronpharma SA, Rancangua, Santiago de Chile, Santiago, Chile; 4 Department of Chemistry, Johannes Gutenberg-University, Fritz-Strassmann-Weg 2, 55128 Mainz, Germany

**Keywords:** Nuclear chemistry, Diagnostic markers, Synthetic chemistry methodology

## Abstract

Positron emission tomography (PET) is a powerful imaging technique for biomedical research, drug development and medical diagnosis. The power of PET lies in biochemically selective radiotracers, labelled with positron emitters like fluorine-18 image chemical processes in vivo. A rapid and remarkably efficient, unprecedented protocol to select between S-F and C-F bond formation based on activation of 1,1-difluoroethylene groups followed by selective oxidation or reduction is described. While transition metal mediated conditions can be employed, the reaction proceeds in high yield using unobjectionable chemical reagents amenable to routine radiotracer production. The latter bodes well for facile clinical translation of the method. The new technique affords radiotracers and the labelling reagent 2,2-difluoro-2-(fluoro-^18^F)ethyl 4-methylbenzenesulfonate **(**[^18^F]**1b)** in excellent yield. Following oxygenation of the reaction mixture with medical oxygen or air, sulfonyl fluorides are obtained as the primary product. The new protocol was employed in a proof of principle to develop a radiometric assay for quantitation of sulfonylation yield with sulfonyl fluoride reagents. With operational ease and mild conditions, the method bodes a high potential for radiolabelling of biomolecules, known enzyme inhibitors and other temperature-sensitive compounds.

## Abstract (web summary)

The fluorine isotope ^18^F is a PET nuclide used for imaging studies, but the incorporation of [^18^F]HF into 2,2-difluorovinyl groups to generate radiotracers and labeling reagents has only been achieved in low yields. Here, using a combination of Et_3_SiH and TEMPO in the absence of oxidizing agents is shown to afford a higher yield, better product quality, and controllable selectivity between the formation of a S–F or C–F bond.

## Introduction

The neutron-deficient fluorine isotope ^18^F is one of the most used PET nuclide^1–8^ and the only radioactive isotope of fluorine of relevance in synthetic chemistry. This is due to a half-life of 109.7 min, which renders ^18^F suitable for multi-step reactions, commercial distribution of ^18^F-radiopharmaceuticals and convenient handling of the tracer in imaging studies^9–11^. ^18^F readily forms stable bonds to carbon atoms making possible the straightforward introduction of fluorine into most organic molecules. Fluorine-18 is produced via the ^18^O(p,n)^18^F nuclear reaction by irradiation of [^18^O]H_2_O liquid targets. This route furnishes no-carrier-added (n.c.a.) [^18^F]fluoride ion in high yield and high molar radioactivity, i.e. a high ratio of radioactive to non-radioactive compound (e.g. 1 GBq/nmol). High molar activity ( > 100 MBq/nmol) is a requirement to achieve genuine tracer conditions for PET-imaging of saturable biological systems^12–14^ in human subjects and particularly in small animals. In turn, n.c.a. radiochemistry involving fluoride ion is characterised by kinetic and thermodynamic peculiarities, not seen in stoichiometric organic fluorine chemistry.

The current report is an account of our ongoing efforts to develop new chemical reactions involving activated fluoride ion in sub-stoichiometric amounts as is the case for n.c.a. radiochemistry.

**Fig. 1:**
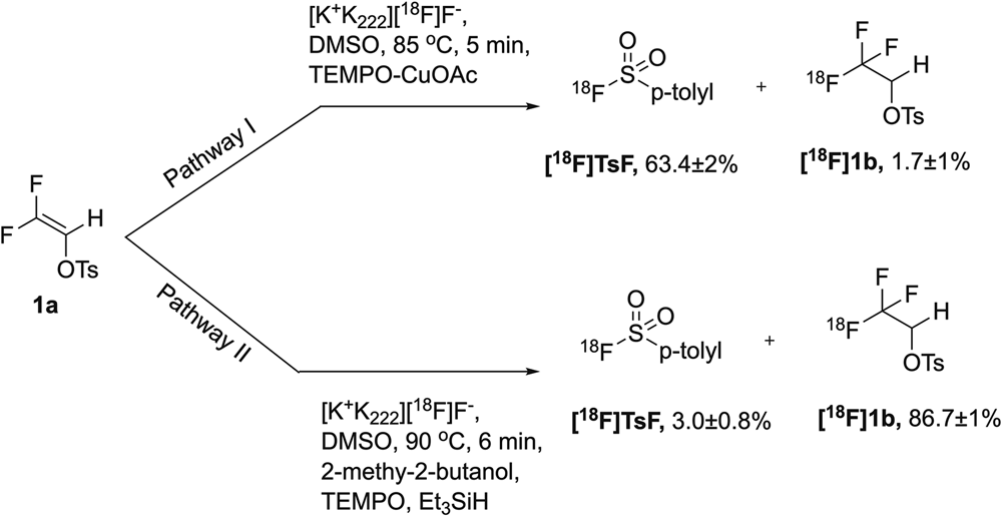
Overview of the reaction pathway facilitated by the new mechanism. The scheme depicts the protocol to select between S–F and C–F bond formation based on activation of 1,1-difluoroethylene groups followed by selective oxidation or reduction. Data are mean values ± SD for each reaction performed in triplicates.

Recently, we have reported a highly applicable and convenient methodology for direct nucleophilic radiofluorination of trifluoroalkyl ((CH_2_)_n_CF_3_) groups and translated it to clinical use^15–18^. Incorporation of the radiolabel was achieved by formal nucleophilic addition of [^18^F]HF to 2,2-difluorovinyl tosylate (**1a**) as shown in Fig. 1. Despite providing a unique scope for radiolabelling and imaging, the method’s shortcoming was a low isolated yield^17–19^ which scientists have struggled to mend for years^3,16,19^. Here we report the discovery of a single electron transfer (SET) mechanism leading to remarkable improvements in radiochemical yield and widely improved substrate scope.

## Results and Discussion

### Protocol for selecting between fluorine-sulfur and fluorine-carbon bonds

The 2,2-difluorovinyl motive carries an unusual, electrophilic carbon centre amenable to nucleophilic attack by fluoride ion. Formal addition of [^18^F]HF to the double bond allows for ^18^F-labelling of complex scaffolds and drug molecules by forming [^18^F]trifluoroalkyl derivatives. The pathway relies on a rapid and efficient protonation of the carbanion intermediate obtained upon C-F bond formation to avoid successive addition-elimination cycles. These cycles reduce molar activity of the labelled product.

Even though some progress was made to improve the reaction outcome, the reaction was never fully under control^15–18^.

We have now identified a new, superior reaction pathway which not only leads to higher yield and better product quality but also allows to select between the formation of a fluorine-sulfur bond or a fluorine-carbon bond. Through this protocol, ^18^F-labelled sulfonyl fluorides and trifluoroalkanes are obtained in high yields.

Arenesulfonyl fluorides are found in kinase-inhibiting drugs and in tool compounds for the site-selective modification of proteins in chemical biology. More recently, radiolabelled sulfonyl fluorides have been added to the scope of functional groups for PET^20–25^.

The present investigation was initiated by observation of partially deuterated products when the protonation mechanism depicted above was investigated in NMR experiments (Table S1, Figures S1, S2). Deuteration occurred in up to 60% of the product, with only minor differences in between additives and time of addition both, in the presence and absence of proton sources in neat NMR solvents.

**Fig. 2:**
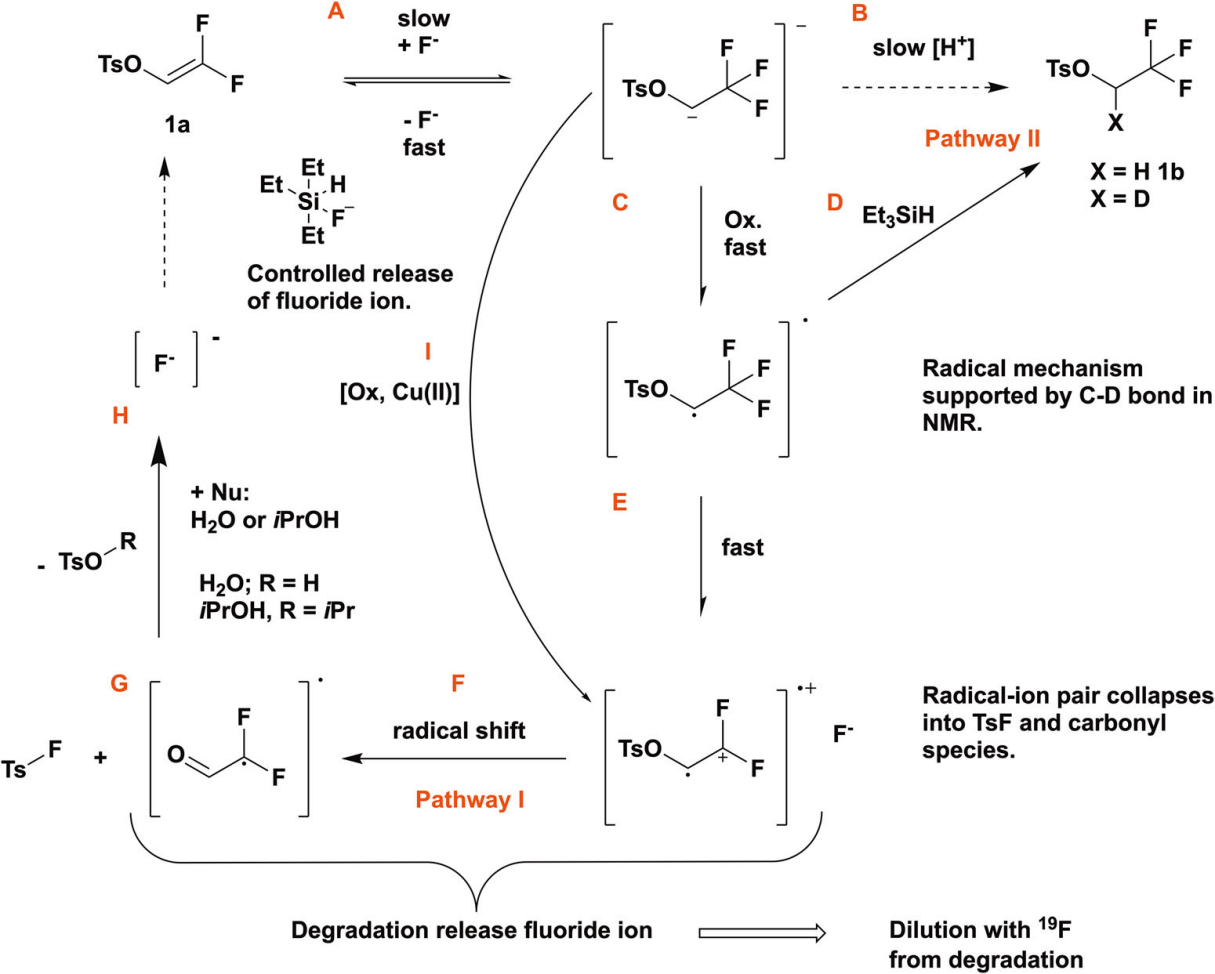
Mechanistic aspects of the reaction. The reaction in our understanding follows the SET oxidation route. Competing E2 elimination (**A**) leads to the formation of [^18^F]**1a**; irreversible protonation is too slow (**B**); oxidation of the carbanion (**C**, **D**) is the actual rate-limiting step, as a result, fluoride is trapped in the product (**D**) via a radical intermediate. Overoxidation (**E**) (pathway I) suppressed [^18^F]**1b** formation via radical shift (**F**) and dissociation (**G**), to form sulfonyl fluoride that is hydrolysed to toluenesulfonic acid (or esters, **H**), liberating fluoride ion in the process. Due to the fast reaction rate of the SET mechanism (Pathway I: **C**, **F**, **G** and Pathway II: **C**, **D**), both [^18^F]**1b** and sulfonyl [^18^F]fluoride can be obtained in n.c.a. quality now.

These findings imply that the formation of product [^18^F]**1b** must involve a SET oxidation of the intermediate carbanion leading to a radical. This would facilitate the abstraction of a deuterium radical from the solvent instead of the previously proposed protonation of an anionic intermediate. The oxidative route was further corroborated when we found that further oxidation of the carbon in question eventually leads to dissociation of the S-O bond followed by fluorination of the sulfonyl group (Scheme S1, Fig. 2).

In the light of the novelty and the potential synthetic utility of this previously undescribed mechanism to select between ^18^F-labelled trifluoroethanes and sulfonyl fluorides, we began investigating.

To test the oxidation hypothesis, we compared the outcome of the reaction under optimised conditions and with oxygenation of the reaction medium with air. A primary screen of carrier bases, oxidising and reducing agents against difluoroolefins in presence and absence of air was conducted (Supplementary methods, described in Tables S2–S6). Reactions showing a combined radiochemical yield >50% were replicated and thoroughly analysed by radioHPLC (Supplementary Data 2, chromatograms: Figures S39–S57). When comparing the outcome of the reaction under N_2_ atmosphere and air, a major change of the product distribution was observed. Compound [^18^F]**1a** was obtained as the main product and toluenesulfonyl fluoride was formed in about 15% radiochemical yield. The ratio between [^18^F]**1a** and [^18^F]**1b** was almost inverted (Table 1 entries 1 and 2). Addition of 2-propanol or water marginally reduced the detected amount of [^18^F]TsF (entry 3), this effect was most pronounced with degassing of the reaction mixture (entry 3 and 10).

**Table 1:** Reaction optimization for shifting the equilibrium of labelling among [^18^F]1b, [^18^F]TsF and [^18^F]1a

Entry (substrate)	Additive	RCY (%)		
		[^18^F]TsF	[^18^F]1a	[^18^F]1b
1 (**1a**)	Air	14.9 ± 0.8	51.1 ± 0.8	34.3 ± 0.8
2 (**1a**)	N_2_	4.9 ± 0.8	9.1 ± 2.1	62.3 ± 2.9
3 (**1a**)	*i-*PrOH, N_2_	3.3 ± 2	19.3 ± 0.6	63.3 ± 1
4 (**1a**)	N_2_/CuOAc, TEMPO	63.4 ± 2	19.6 ± 2	1.7 ± 1
5 (**1a**)	CuOAc, Air	9.4 ± 2	71.7 ± 2	traces
6 (**1a**)	CuCN, Air	29.6 ± 2	27 ± 1	14.5 ± 3
7 (1a)	CuBH_4_	traces	6.4 ± 1	71.5 ± 3
8 (**1a**)	N_2_/CuOAc TEMPO, phosphite	23.5 ± 2	29.0 ± 2	44.1 ± 3
9 (**1a**)	(Bu)_4_SnH	9.9 ± 0.6	4.1 ± 1	2.6 ± 1
10 (**1a**)	No additive	34.3 ± 2	31.4 ± 0.9	22.1 ± 1
11 (**1a**)	*i-*PrOH, air	16.5 ± 0.7	5.9 ± 1	52.6 ± 1.3
12 (**1a**)	Et_3_SiH, Ag (II)	37.9 ± 1	28.4 ± 2	6.5 ± 0.5
13 (**1a**)	TEMPO, Et_3_SiH, Ag (II)	25.8 ± 1	48.1 ± 1	19.8 ± 1
14 (**1a**)	Et_3_SiH, 2-propanol, Ag (II)	37.3 ± 1.3	6.9 ± 0.6	50.3 ± 2
15 (**1a**)	TEMPO, Et_3_SiH, 2-propanol, Ag (II)	22.1 ± 2	7.2 ± 2	61.3 ± 0.8
16 (**1a**)	TEMPO, Et_3_SiH, 2-propanol,	15.8 ± 0.7	8.2 ± 1	74.0 ± 1.6
17 (**1a**)	TEMPO, Et_3_SiH,2-methyl-2-butanol	3.0 ± 0.8	0 ± 1	86.7 ± 1
18 (**1a**)	TEMPO, Et_3_SiH, 2-methyl-2-butanol (1 mL) no DMSO	55.4 ± 2	0 ± 2	36.2 ± 2

The starting material **(1a)** 17 µmol was dissolved in DMSO (1 mL) containing Et_3_SiH (1 equiv.) and 2-methyl-2-butanol (10%, v:v). Radio-HPLC was used to confirm the product formation and the corresponding proportions. RCY= Radiochemical yields are given in %age. Data are mean values ± SD for each reaction performed in triplicates.

Copper salts in combination with organic aminoxyls like 2,2,6,6-tetramethyl-1-piperidinyloxy (TEMPO) provide an admirable redox potential for oxidation of alcohols to carbonyls^26^. Cuprous acetate formed in situ from TEMPO and cupric acetate further improved the yield of [^18^F]TsF (63%), with further reduction of [^18^F]**1b** formation down to less than 2% (entry 4). When cupric acetate was used in absence of TEMPO, [^18^F]**1a** was obtained as the main product (72%) together with [^18^F]TsF (9%) and [^18^F]**1b** (<1%) (entry 5). In the presence of CuCN and air, all three products were obtained in double-figure radiochemical yields between 14 and 30% (entry 6). Most interestingly, cupric borohydride (entry 7) suppressed the formation of [^18^F]TsF entirely to yield >72% of [^18^F]**1b** together with a small amount (6–7%) of [^18^F]**1a**, presumably through interception of the oxidative route. In the presence of bisethylhexyl phosphite as a hydryl radical (H) donor and CuOAc-TEMPO, [^18^F]**1b** formation was preferred (45%) albeit in conjunction with 23% [^18^F]TsF (entry 8). As common radical sources such as (Bu)_4_SnH performed worse (entry 9), we decided to investigate other reducing agents in presence and absence of oxidising agents as exemplified for Ag(II) in Table 1 entries 12–15. These efforts lead us to identify the combination of Et_3_SiH and TEMPO in absence of oxidising agents as key to success (Table 1 entries 15–17)_._ With some tweaking of the additives [^18^F]**1b** was formed in a radiochemical yield (RCY) of 87% and a [^18^F]**1b**-[^18^F]TsF ratio of 29 (Table 1 entry 17).

These experiments were paralleled by NMR studies to identify hypervalent fluorosilicates as the active species. In our theory, an ^18^F fluorosilicate intermediate (Et_3_SiH^18^F^-^) formed in situ controls ^18^F-attack and subsequent hydryl transfer to make for a most effective mediator of irreversible addition (Supplementary Note 1, a putative reaction mechanism section, Figs. S1, 2)_._ An excess of fluoride ion is not necessary to achieve high conversion of the precursor. Instead, 80% of the difluorvinyl precursor is converted into the trifluoroethyl motif. We surmised, that the fluorosilicate acts as a fluorine source by controlling the availability and hardness of fluoride nucleophile that mediates slow, controlled release of fluoride upon fluorination. This is supported by observation of the fluorosilicate in a mere 4% in NMR studies alongside the starting material **1a** (18%) and product **1b** (78%) which were formed rapidly from Cesium fluoride within 6 min (Fig. S1). Control reactions in absence of TEMPO, Et_3_SiH, alcohol and/or metal resulted in variable proportions of [^18^F]TsF, [^18^F]**1a** and [^18^F]**1b** (RCY = 20–30%), which is in stark contrast to both selectivity and yield of the effective reagent combination. A reaction time of 6 min with 85 °C in presence of TEMPO, Et_3_SiH, 2-methyl-2-butanol resulted in 87% yield of [^18^F]**1b**, 3% [^18^F]TsF and no [^18^F]**1a** (see Table 1 entry 17). Application of 2-methyl-2-butanol as the solvent resulted in the production of more [^18^F]TsF (RCY = 55%) than [^18^F]**1b** (RCY = 36%) (Table 1, 18).

The contrasting nature of our findings lead us to dismiss earlier theories on the reaction mechanism.

Instead, we are convinced that the mechanism involves the SET oxidation and, consequentially, allows for selection of different pathways as shown in Fig. 2:

### Plausible reaction mechanism

All things considered, the conditions required for efficient radiofluorination favour the competing E2 elimination (Fig. 2, **A**), which leads to the observable formation of labelled **1a** whenever one of the ^19^F-atoms is eliminated from the intermediate anion instead of the ^18^F-atom. Since a 10^4^-fold excess of precursor is present under n.c.a. conditions, addition-elimination eventually leads to sequestration of all available radionuclide in the form of labelled precursor [^18^F]**1a** instead of desired [^18^F]**1b**. We know that the rate constant for elimination is higher than the rate constant of protonation, which leads to a built-up of the labelled precursor [^18^F]**1a**. We now discovered that oxidation of the carbanion (Fig. 2C, D) is the actual rate-limiting step for the irreversible formation of [^18^F]**1b** and that protonation is too slow to mediate this (**B**).

Our experiments in the presence of TEMPO and reducing agents confirmed the above (Table 1, entries 13 and 15–18). Oxidation of the carbanion intermediate provides a means to suppress the elimination of fluoride ion but does not limit the nucleophilic attack. As a result, fluoride is trapped in the product (**D**) and the reversible mechanism turns into an irreversible pathway (Pathway II). The mechanism explains the partial deuteration of the product observed in NMR studies, even in the absence of ^2^H^+^ sources (Supplementary information, NMR signals of key components: Table S1). We deduced that not slow protonation but rather oxidation of the anion to a radical (**C**), followed by homolytic abstraction of a proton from (NMR-)solvent (**D**) were the primary pathways to [^18^F]**1b** as confirmed by deuteration seen in NMR. Overoxidation (**I**) suppressed [^18^F]**1b** formation in all experiments to become a minor product. This line of thought is particularly plausible when considering the role of additives such as alcohols or aqueous mixtures employed to ‘protonate’ the anionic intermediate (**H**).

Instead of protonation, it is rather likely that these additives react with the sulfonyl fluoride to form toluenesulfonic acid or esters, liberating fluoride ion in the process (Fig. 2F–H), which in turn attacks another substrate molecule (**A**) in a cycle of successive consumption of the starting material. These hypotheses are substantiated by progressive consumption of the substrate **1a**, which can be observed by HPLC and NMR.

Scope and limitations of the reaction are now apparent: The formation of [^18^F]**1a** is sensitive to rapid oxidation of the anionic intermediate. While unavoidable, rapid SET oxidation (**C**) followed by trapping of the radical (Fig. 2D) benefits the [^18^F]**1a**:[^18^F]**1b** ratio, as the cycle can be broken here. Oxidation of the carbon to a carbonyl oxidation state produces [^18^F]TsF as the main product in two stages via radical-ion pair formation (Fig. 2E–G) and subsequent collapse to the TsF (Table 1, entry 16, [^18^F]**1a**:[^18^F]**1b** = 1:29, Fig. 2G). In absence of water or alcohol, [^18^F]**1a** can be suppressed to a large extend due to the fast reaction rate of the SET mechanism (Pathway I: **C,** **F,** **G** and Pathway II: **C,** **D**). Thereby both [^18^F]**1b** and sulfonyl [^18^F]fluoride can be obtained in n.c.a. quality, i.e. dilution of the radioactive ^18^F with ^19^F released from the substrate **1a** can be avoided^15^. Thereby, high yields and high molar activities are finally within reach^1,17–19^.

With the help of the new protocol a much higher molar activity of up to 413 MBq/nmol was eventually achieved at the end of synthesis (27 min from end of beam (EOB), 5 GBq starting activity, ca. 10 nmol **1a** in 10 mL of 9% EtOH in 0.9% saline).

**Fig. 3:**
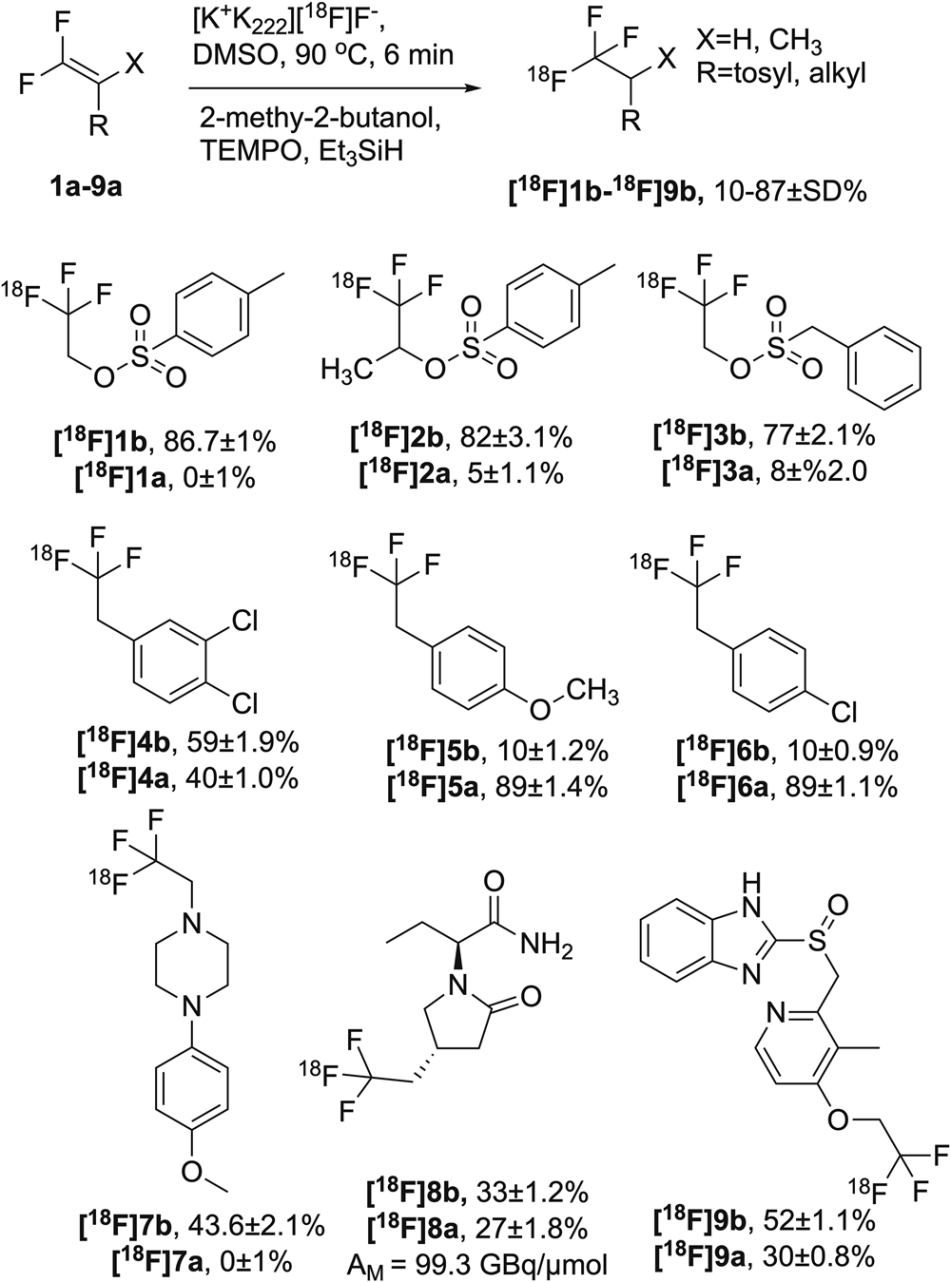
^18^F-labelling of different difluorovinyl-containing compounds by direct method as mentioned in Fig. 1 (pathway II). Data are means ± SD.

Encouraged by the positive outcome, we further developed the reaction we sought to establish the substrate scope made possible by the redox mechanism. When screening with [K^+^⊂crypt-_222_][^18^F]F^-^ cryptate under optimized conditions substrates were labelled in high yields and good selectivity of C–F and S–F bond formation was achieved (Figs. 3, 1–3). The reactivity observed with **1a** was largely conserved when 1,1-difluoropropene-2-yl tosylate (**2a**) was employed (Table S6), though **2a** was found to be a superior substrate to **1a** for production of [^18^F]TsF.

### Substrate scope of the method

The true impact of our new method became apparent when substrates devoid of sulfonate groups and complex drug compounds were employed (**4a–9a**, Fig. 3). Styrenes **4a–7a** were labelled in up to 59% RCY together with 40% labelled precursor (Fig. 3, [^18^F]**4b**, [^18^F]**4a**). Most strikingly, however, Seletracetam (UCB44212) (Fig. 3, [^18^F]**8a**), an SV2A binding nootropic drug candidate^27,28^, was labelled and purified by semi-preparative HPLC to give [^18^F]**8b** in 33% RCY, a molar activity (A_m_) of 99.3 GBq/µmol and a radiochemical purity (RCP) > 99% from 12.2 GBq starting activity. Lansoprazole had proven most challenging to label in previous years, however, under the new conditions we achieved a 50% RCY (Fig. 3, [^18^F]**9b**).

### Implementation of the protocol

With the optimized protocol in hand, we proceeded addressing the separation of [^18^F]**1a** and [^18^F]**1b** from labelled products for use in indirect labelling. The conditions for the introduction of the [^18^F]trifluoroethyl moiety into amine-containing drug molecules were optimised and DMSO with Cs_2_CO_3_ as a base at 140 °C were found to be the most effective conditions (Table S7).

**Fig. 4:**
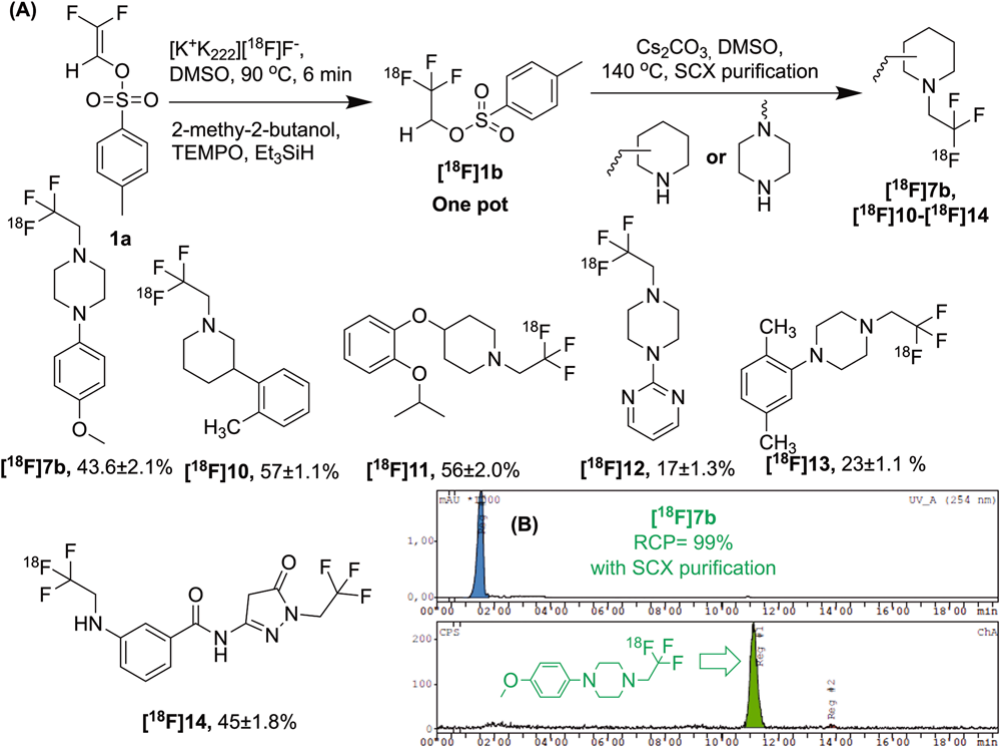
Labelling of potential radiotracers. **A** Synthesis scheme with a range of ^18^F-labelled piperazine and piperidine-based drug molecules, **B** Analytical HPLC chromatogram of [^18^F]7b with RCP > 99%. Data are means ± SD.

Following the alkylation reaction, the product was extracted from the reaction mixture by means of cation-exchange solid phase extraction (purity >99%), leaving behind the unreacted [^18^F]**1a** as well as other side products. The beauty of the protocol is one-pot radiolabeling of amines achieved by utilizing their different nature in the SPE purification step. Since tertiary amine derivatives like those employed here are prominent pharmacophores from the medicinal point of view^29–31^, the methodology is a key to PET. Piperazine and piperidine-based scaffolds including drug [^18^F]**14** were labelled indirectly in moderate to good RCY (17–57%, Fig. 4A) and RCP ≥ 99% (Fig. 4B) using [^18^F]trifluoroethyl tosylate.

### In vitro proof-of-concept with [^18^F]2a

All that was left at this point was to put the new method to a test for radiosynthesis of sulfonyl fluorides. As sulfonyl fluorides are often used to attach fluorescent labels to proteins or to inhibit the active centres of hydrolase and kinase enzymes by sulfonylation, we sought to combine labelling with a possible use of the labelled product. Since toluenesulfonyl fluoride is a non-selective hydrolase inhibitor we decided to scale the reaction up to batch production of [^18^F]TsF and test if it may become useful for monitoring protein modification based on release of [^18^F]fluoride ion. As a proof of principle, a straightforward radiometric assay capable of monitoring reagent consumption, non-specific binding and extend of functionalisation during sulfonylative protein modification was developed (Supplementary methods, section explaining in vitro quantitative analysis [^18^F]**2a**). We found that indeed, intact radiotracer as well as fluoride ion released in the protein-sulfonylation reaction could easily be quantified by counting the released [^18^F]fluoride activity, thus showing promise for application in chemical biology. Besides TsF the method will also prove well suited for labelling of sulfonyl fluorides in other radiopharmaceuticals.

## Conclusion

For the first time, the direct nucleophilic ^18^F-fluorination of trifluoroethyl groups was achieved in excellent radiochemical yields with practically useful molar activity. This sought-after achievement paves the path for the development of new radiotracers and drug candidates based on the abundant di- and trifluoroethyl motif. Besides, the utility of the fluorophile 2,2-difluoroethenyl sulfonate in radiochemistry is expanded to a highly useful application. Under oxidising conditions umpolung of the anionic alpha carbon followed by bond dissociation can be exploited to produce high yields of ^18^F-labelled sulfonyl fluorides in useful amounts radiotracer production.

## Methods

### General

Precursors for radiolabeling experiments and their references were produced via standard methods from commercially available starting materials and the identity was confirmed via comparison to literature reports^15^. Characterization techniques like NMR and mass spectrometry were used to confirm their structures. [^18^F]fluoride ion was produced using the ^18^O(p,n)^18^F nuclear reaction via proton bombardment (16.3 MeV) of an H_2_^18^O liquid target on a GE PETtrace cyclotron at a beam current of 30–40 µA for 5 to 10 min. To understand the reaction pathway, non-radioactive (cold) control experiments were conducted. In these experiments, the procedure developed in radioactive experiments were followed in presence of ^19^F. KF, TBAF and CsF were used in different experiments as the source of fluorine.

### Radiochemistry

The radionuclide was extracted from the enriched target water using anion exchanger cartridge (CO_2_^2--^form). A SupelTM-Select SAX SPE (30 mg) cartridge was preconditioned for [^18^F]fluoride extraction by slowly passing a K_2_CO_3_ solution (1 M, 5 mL) through it, followed by type I water (10 mL) followed by air (30 mL) with a syringe. Elution solution was prepared by adding 15 µL of 1 M K_2_CO_3_ to 11.2 mg of Kryptofix 222 and left for 5 min followed by addition of 985 µL of acetonitrile. An aliquot of the [^18^F]fluoride in [^18^O]H_2_O was withdrawn into the preconditioned cartridge (0.1–0.6 mL), and air was passed through (10 mL) to remove excess target water. The [^18^F]fluoride was eluted from the cartridge by passage of elution solution (1 mL) through it, and the eluent was collected into a V-vial. The solvent was then evaporated on a hot plate at 90 °C under an argon stream. Residual water was removed from the [^18^F]fluoride by additional 3 cycles of addition/evaporation of MeCN (1 mL). The vial was removed from the hot plate, capped, and cooled for about 30 s on an ice bath.

### Direct radiolabeling of 2,2-difluorovinyl-based precursors

The corresponding 2,2-difluorovinyl moiety-containing precursor (17 µmol) was dissolved in 1 mL DMSO. Triethylsilane (17 µmol), (2,2,6,6-Tetramethylpiperidin-1-yl)oxyl or (2,2,6,6-tetramethylpiperidin-1-yl)oxidanyl (TEMPO) (17 µmol) and 2-methylbutan-2-ol (0.9 mmol) were added at room temperature. The mixture was degassed into a screwcap vial by passing N_2_ gas for 10 min before the start of the labeling reaction. This reaction mixture was added to the dried and cooled [^18^F]fluoride and the mixture is homogenized for about a minute. Once the reagents have been mixed, the activity vial was measured and placed on the hot plate at 90 °C for 6 min. The seal of the vial was tightened carefully to avoid evaporation of the reagents. At the end point of the reaction, the reaction vial cooled on ice for 1–2 min to stop the further reaction followed by measurement of the activity. 100 µL from the reaction vial was transferred to an Eppendorf tube containing 1 mL solvent (acetonitrile-water, 1:1), out of which 10 µL was injected directly into an analytical HPLC.

### Indirect radiolabeling of piperidine/piperazine-based compounds

The synthesized 2,2-difluoro-2-(fluoro-^18^F)ethyl 4-methylbenzenesulfonate ([^18^F]**1b**) after confirmation from HPLC was used for indirect radiolabeling of different amines/phenols without purification. Piperidines/piperazine based amines (39 µmol) and Cs_2_CO_3_ (61  µmol) were added to the reaction mixture containing (90%) [^18^F]**1b**. A magnet bead was added to help dissolving the base in the reaction. After the activity check, the reaction vial was allowed for heating cum stirring for 40 min at 140 °C. At the end point of the reaction, the reaction vial was cooled on ice for 1–2 min to stop the further reaction followed by measurement of the activity. 100 µL from the reaction vial was transferred to an Eppendorf tube containing 1 mL solvent (acetonitrile: water =1:1), out of which 10 µL was injected directly into an HPLC.

### Quality control and purification

SCX cartridge (Merck) was conditioned by passing 5 mL 1 M HCl through followed by washing with water until the elute was neutral. For the purification of the different trifluoroethyl amines, the reaction mixture was diluted with water and neutralized using 1 M HCl. The reaction mixture was passed through preconditioned SCX cartridge. The cartridge was washed with water. The trapped product was eluted with 5 mL Dulbecco’s PBS (10x), 10 µL out of which was injected into HPLC for quality control. The radiochemical yield was determined by decay correction of the counted activity to the start of synthesis using Rad pro Calculator. At the end of the reaction, the total activity was compared to the activity of the pure extracted product by counting of the vials. Different HPLC systems were used for quality control, confirm, identify, and determine radiochemical purity of the products. Traces of the product in the SCX cartridge and the aqueous phase after extraction were neglected.

After the production of crude **8b**, the product was diluted with 5 mL MeCN-water; 30:70 and syringe filtered to remove any solids wastes. A semipreparative Luna PFP column (Phenomenex; 5 μm, 100 Å, 250 mm × 10 mm) with an isocratic mixture of MeCN-water; 30:70 was used at a flow rate of 5 mL/min for HPLC purification. The idea of product containing HPLC fractions was obtained roughly by dose meter. Later the fractions were checked using analytical HPLC for quality control. >99% pure fractions of **8b** were obtained and was counted using a dose calibrator. The radiochemical yield was determined by decay correction of the counted activity to the start of synthesis.

### Supplementary information

Supplementary information

Description of Additional Supplementary Files

Supplementary data 1

Supplementary data 2

## Data Availability

See the Supplementary information file containing the supplementary methods, procedures, results and discussions. For the NMR spectra and chromatograms, see Supplementary data files 1, 2, respectively.
