# Supplementary material for: A protocol for controlled reactivity shift in the 2,2-difluorovinyl motif used for selective S–18F and C–18F bond formation
Source: Commun Chem. 2024 Apr 29;7:97. doi: 10.1038/s42004-024-01132-3 (PMC11058245; doi:10.1038/s42004-024-01132-3)
Supplement: Supplementary file 1 — Supplementary information [file 42004_2024_1132_MOESM1_ESM.pdf]

Supplementary Information

A protocol for controlled reactivity shift in the 2,2-difluorovinyl motif used for selective S–^{18}F and C–^{18}F bond formation

Mudasir Maqbool[1,2], Jimmy Erik Jakobsson[1], Santosh Reddy Alluri[1], Vasko Kramer[3] and Patrick Johannes Riss[*1,2,4]
DOI: 10.xxxxxxxx

[1]*Department of Clinical Neurocience, OUS-Ullevål, Oslo, Norway.*
[2]*Department of Chemistry, University of Oslo, Oslo, Norway. Tel: +4795028669; E-mail: patrick.riss@kjemi.uio.no*
[3]*Positronpharma SA, Rancangua, Santiago de Chile, Chile*
[4]*Department of Chemistry, Johannes Gutenberg-University, Fritz-Strassmann-Weg 2, 55128 Mainz, Germany. Tel. +49 6131 39 28081; Email: priss@uni-mainz.de*

Table of contents

		Page
1.	Supplementary Note 1. A putative reaction mechanism (Scheme 1, pathway II).	2
2.	Supplementary methods	3
2.1.	General	3
2.2.	Non-radioactive control experiments	3
2.2.1.	General procedure for NMR experiments	3
2.2.2.	Experiments for identification of side products	4
2.2.3.	Degree of deuteration and reaction kinetics	6
2.3.	Concentration of the labelled product in reaction mixtures and in formulation:	6
2.4.	Screening of Tosyl sources	7
2.4.1.	Experimental procedure for sulfonyl activation studies:	7
2.4.2.	Screening of additives – broad spectrum screen.	8
2.4.3.	Experimental procedure for reaction scheme 1, addition pathway:	8
2.5.	General procedure for radiolabeling (reaction scheme 1, pathway II):	10
2.5.1.	Extraction, drying and preparation of radioactive fluoride.	10
2.5.2.	Degassing of solvent.	11
2.6.	^{18}F-labelling of other difluorovinyl-containing compounds by direct method:	11
2.7.	Indirect labeling (alkylation) by direct method.	11
2.8.	Purification of trifluoroethyl amines	11
2.9.	Calculation of radiochemical yield (RCY)	11
2.10.	Purification of product 8b using Seimipreperative HPLC	11
2.11.	Determination of molar activity of Seletracetam (8b)	11
2.12.	*In vitro* quantitative stability checks for [^{18}F]2a	12
3.	Supplementary results and discussion	12
3.1.	Screening of different reaction conditions to optimize the synthesis of [^{18}F]1b	12
3.2.	Reaction optimization conditions for labelling substrate 2a by direct method	13
3.3.	Conditions for the synthesis of 1-(2,2-difluoro-2-(fluoro-^{18}F)ethyl)-4-(4-methoxyphenyl)piperazine (7b).	14
3.4.	General synthesis of precursors and their reference molecules	15
3.4.1.	General procedure for the conversion of aldehydes into 2',2'-difluorostyrenes	18
3.4.2.	Synthesis of the reference compounds	17
4.	Supplementary References	18

1. Supplementary Note 1. A putative reaction mechanism (Scheme 1, pathway II).

Since F^- is known to form highly stable bonds to Si atoms, it is typically used as reagent to cleave off silicon containing protecting groups from nucleophilic sites such as carbon and oxygen. Therefore, silane and siloxide moieties with a high affinity for F^- can be seen as an ^{18}F acceptor. Fluorination of the silicon leads to formation of a fluorosilicate intermediate centered on a hypervalent silicon atom, which may enhance the fluorination reaction. The fluorosilicate anion provides a much-reduced hardness compared to fluoride ion, for example to avoid transition of metal catalysts into high oxidation states [1]. Fluorosilicates are well known to facilitate various synthesis routes[2], because combination of fluoride with silicon increases its redox potential to such extend that it can reduce aldehydes and ketones even without the help of a Lewis acid. For the sake of simplicity, the difluorvinyl moity may be regarded as an analogue of a carbonyl group and, following oxidation of the intermediate carbanion, its reduction paves the way to irreversible fluorination.

By means of rapid and strong Si–F bond formation, for example in fluorotriethylsilane (F–$SiEt_3$)[3], such silanes have been utilized as a fluoride trap. This concept has been extensively used in ^{18}F labeling of many desired drug-like molecules as well. [^{18}F]fluorotrimethylsilane ([^{18}F]FTMS) via Si–F bond formation has been reported back in 1985[4] from chlorotrimethylsilane (TMSCl) in which it was observed that F-replacement of Cl on Si has to be faster than the hydrolysis of Si–F bond as FTMS is prone to rapid hydrolysis, which in our case would have been reason for poor yield in reactions run in air and without degassing the solvents. In the same way, a biotin-conjugated tetra-[^{18}F]fluorosilicate product was synthesized with RCC >99% where Si–^{18}F bond formation was achieved by aqueous S_N of Si-centered ethyl esters in the presence of [^{19}F]KHF_2 under strongly acidic conditions at room temperature [5]. Organosilicates undergo autocatalysis and enhance radiofluorination in presence of potassium ions[6]. In our extension of the fluorosilicate chemistry, the reagent is used to both, control availability of fluoride ion to suppress undesired cycles of addition-elimination and provides the means to gently hydrogenate the neighboring 'carbonyl' carbon to make irreversible the fluorination reaction[7]. Content of water in such reactions inhibits the nucleophilic reactivity thus reducing the overall RCY. As seen above, the fluorosilicate enhanced condition provides a powerful new means to achieve a nucleophilic addition of fluoride to the fluorophilic difluorovinyl group. In contrast to earlier reports, an excess of fluoride ion is not necessary to achieve high conversion of the precursor. Instead, 80% of the difluorvinyl precursor is converted into the trifluoroethyl motif.

Fluorosilicates are known to be powerful fluorine sources which may release fluoride ion in a controlled fashion as opposed to fluoride salts [1]. Cu-thiophenecarboxylate and fluorosilicate as fluorine source for carboxylation[8–11], when combined with the excellent redox potentials of fluorosilicate reagents a high fluorination yield becomes achievable. Radiolabelling further illustrates the irreversible nature of the reaction mechanism, wherein the ^{18}F-labelled nucleophile moves through the silicate reagent to the electrophilic carbon center, without excessive formation of labelled precursor.

This is further corroborated by the near complete consumption of labelled silicate at end of reaction. In absence of the unsaturated substrate three species remain detectable in model studies with TBAF and $TiPSH_3$, namely TBAF, $TiPSHF^-$, and TiPSH. Notably, no side products were detected in these studies and complex drug-like structure were found to label in excellent yields and molar activities further supporting the new reaction pathway. In our eye, the fluorosilicate finally provides the means to control fluorination and suppress competing elimination reactions.

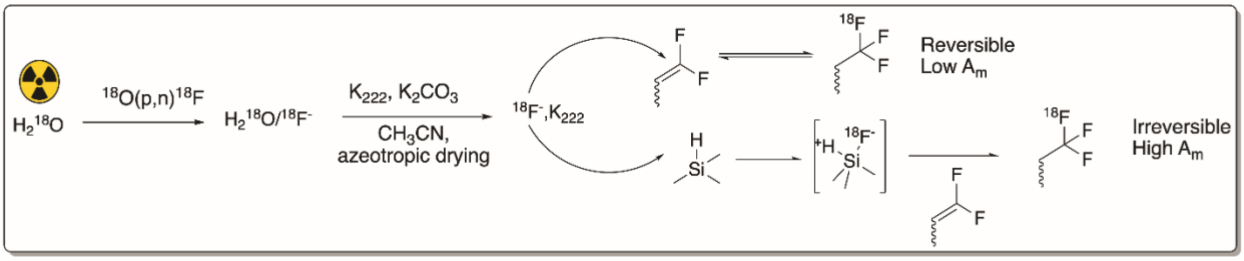

Scheme S1. A putative reaction mechanism depicted for substrate 1a.

2. Supplementary methods

2.1. General:

The solvents and reagents used in the present work were brought from Sigma-Aldrich (Sigma-Aldrich AS, Norway) in analytical quality unless specified otherwise. Starting materials were obtained from different commercial suppliers. Precursors for radiolabeling experiments and their references were produced via standard methods from commercially available starting materials and the identity was confirmed via comparison to literature reports [1]. Solid phase extraction (SPE) cartridges were purchased from VWR (VWR International, Darmstadt, Germany) and Sigma-Aldrich (Sigma-Aldrich AS, Norway). Nuclear magnetic resonance spectra were recorded on a Bruker AVII 400 NMR instrument (Bruker ASX Nordic AB). Chemical shifts (δ) for ^{1}H (400 MHz) and ^{13}C (100 MHz) resonances are reported in parts per million (ppm), relative to the solvent signal ($CDCl_3$ δ = 7.223 ppm), downfield from a theoretical tetramethylsilane signal (TMS, δ = 0 ppm). The multiplicities of NMR signals are designated as s (singlet), d (doublet), dd (double doublet), t (triplet), q (quartet), br (broad), m (multiplet, for unresolved lines). Mass spectrometry was conducted on a Q-Tof-2 mass analyser (Micromass, Q-Tof-2TM) using ESI ion source in positive mode. HPLC analysis of compound purity and quality control was conducted on a Hewlett-Packard 1100 HPLC system (Matriks AS, Agilent Technologies, Oslo, Norway) consisting of a quaternary pump, variable wavelength diode array detector and a Raytest Gina star radioactivity detector (Raytest GmbH, Straubenhardt, Germany) using GABI-star software (Raytest) for instrument control, data acquisition and processing. For determination of purity and stability of formulated radiotracer, a Luna PFP column (Phenomenex; 5 µm, 100 Å, 250 mm × 4.6 mm) with 5 systems of isocratic mobile phases were used: System A: MeCN: H_2O=50:50 was used at a flow rate of 1.5 mL/min and Injection volume = 10 uL. System B: MeCN-H_2O, 3:7; System C: MeCN-H_2O, 1:1; System D: MeCN-H_2O, 6:4 at a volume flow rate of 1.5 mL/min. and System E: 2.0 mL/min MeCN-H_2O, 1:1.UV signals were detected at a wavelength of 254 nm. All other radioactivity measurements during labelling experiments and radiotracer productions were performed using a Atomlab 300 dose calibrator (Biodex Medical Systems). Identity and purity of radioactive products was confirmed via HPLC analysis and compared to their authentic references. The Organic solutions were dried over anhydrous sodium sulfate.

2.2. Non-radioactive control experiments

To understand the reaction pathway, non-radioactive (cold) control experiments were conducted. In these experiments, the procedure developed in radioactive experiments were followed in presence of ^{19}F. KF, TBAF and CsF were used in different experiments as the source of fluorine, however, the reaction with KF was not found to produce any product. The reaction was conducted either DMSO-d_6 in non-deuterated DMSO in order to compare with the actual reaction. ^{19}F-NMR (F-decoupled) was recorded for **1a**, **1b** and the crude reaction mixture. The NMR integrals for fluorine signals were determined after Whittaker smoothing of the baseline. Signals were assigned to the structures drawn over the spectra for example, the reference compound (2,2,2-trifluoroethyl 4-methylbenzenesulfonate (**1b**)) shows a triplet at -72.78, the precursor compound 2,2-difluorovinyl 4-methylbenzenesulfonate (**1a**) showing two double doublets at -91.56 and -109.48. the crude reaction mixture containing 1a, TEMPO, Et_3SiH, 2-methylbutan-2-ol, CsF and DMSO after 6 minutes run at 90 °C showed peaks corresponding to **1a**, **1b** and Si-F at -130.6[12–15].

2.2.1. General procedure for NMR experiments

To a solution of vinyl tosylate **1a** (2 mg) in 1 mL DMSO, TEMPO (1.3 mg), Et_3SiH (1.3 µL) and 2-methylbutan-2-ol (0.1 mL) were added one by one. The reaction vial was capped, and purged with nitrogen 6.0 and shaken well until a homogeneous solution was obtained. CsF (1.3 mg, 1 eq.) was added, and reaction was heated at 90 °C and stirred for 6 minutes. The reaction mixture was filtered and produced for ^{19}F-NMR spectroscopy without any purification. The NMR signals obtained after a representative reaction time were as shown under:

2,2,2-trifluoroethyl 4-methylbenzenesulfonate (**1b**) (reference compound): ^{19}F-NMR (376 MHz, HDMSO) δ -72.77 (t, *J* = 8.6 Hz). 2,2-difluorovinyl 4-methylbenzenesulfonate (**1a**) (precursor): ^{19}F-NMR (376 MHz, HDMSO) δ -91.50 (dd, *J* = 53.2, 15.7 Hz), -109.40 (dd, *J* = 53.4, 3.9 Hz). Cold reaction mixture: ^{19}F NMR (376 MHz, HDMSO) δ -72.86 (t, *J* = 8.5 Hz), -91.62 (dd, *J* = 53.05, 15.3 Hz), -109.54 (dd, *J* = 54.5; 3.8 Hz), -130.06 (dd, *J* = 56.0, 4.0 Hz), -131.09 (dd, *J* = 55.5, 6.5 Hz).

As indicated in the main article, we were primarily interested in determining the mechanism of fluorine shift, we find that it occurs via an intermediate with Si-F-bonding in the reaction mixture. The signal at -131.06 is not from the added fluoride salts. As reported in the literature the signal witnesses for an Si-F[15] bond, complete with C-F coupling, showing the fluorosilicate acts as a source of fluorine in the reaction as well [1].

Table S1. NMR signals of key components in the reaction mixture.

	^{18}F / ppm
Cs-F	-131.09 (dd, *J* = 55.5; 6.5 Hz)
Si-F	-130.06 (dd, *J* = 56.0; 4.0 Hz)
Starting material 1a	-91.62 (dd, *J* = 53.05; 15.3 Hz);<br>-109.54 (dd, J = 54.5; 3.8 Hz)
Product 1b	-72.86 (t, *J* = 8.5 Hz)

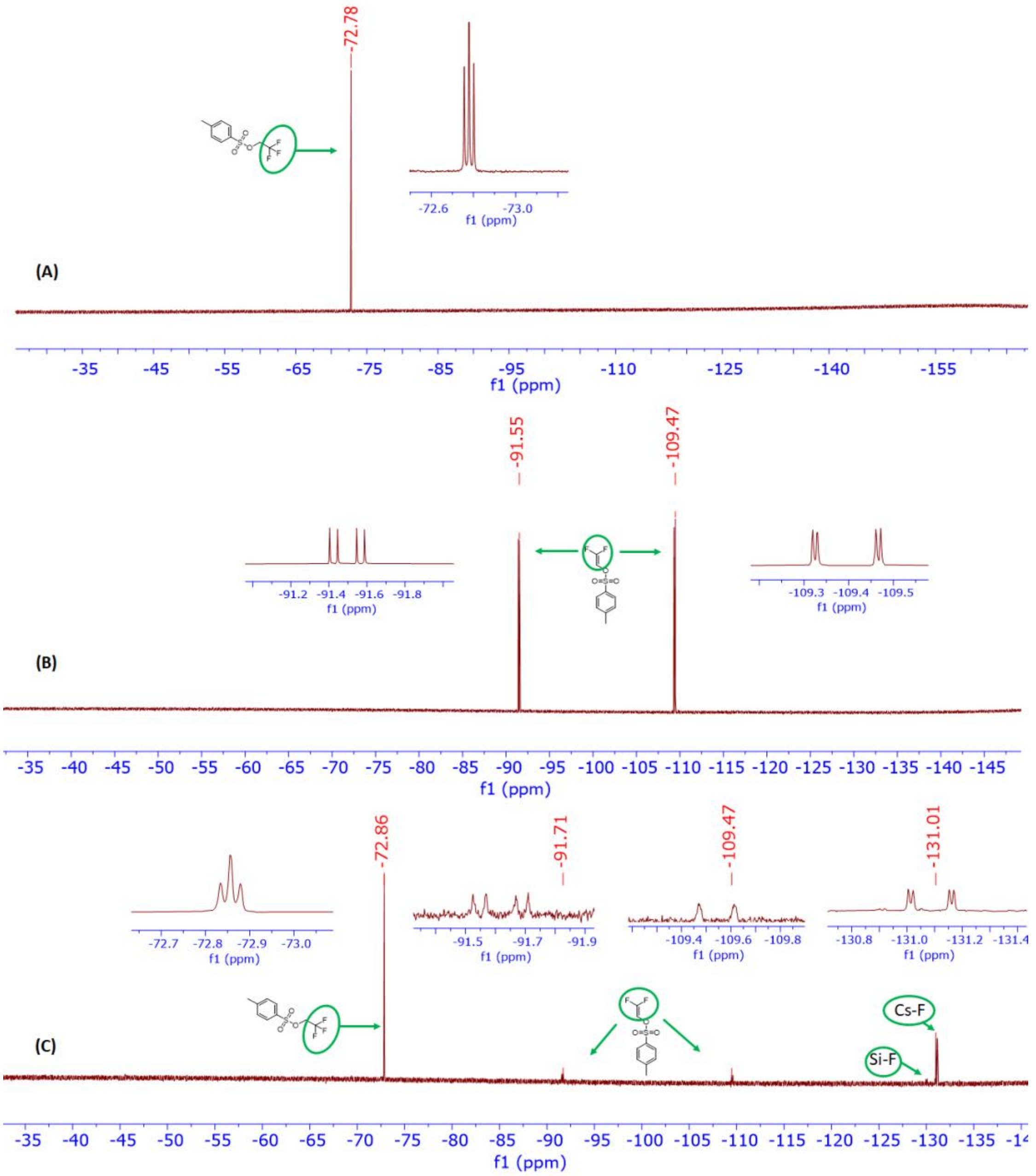

Figure S1: ^{19}F-NMR (F-decoupled) of 1a, 1b and cold reaction mixture under same conditions as that of hot reaction in non-deuterated DMSO in order to compare with the actual hot reaction. Signals were assigned to the structures drawn over the spectra. (A) ^{19}F-NMR of the reference compound (2,2,2-trifluoroethyl 4-methylbenzenesulfonate (1b)) shows a triplet at -72.78. (B) ^{19}F-NMR of the precursor compound 2,2-difluorovinyl 4-methylbenzenesulfonate (1a) showing two double doublets at -91.56 and -109.48. (C) ^{19}F-NMR of the crude reaction mixture containing 1a, TEMPO, Et_3SiH, 2-methylbutan-2-ol, CsF and DMSO after 6 minutes run at 90 °C. Peaks corresponding to 1a, 1b, Si-F at -130.06 and Cs-F at -131.19 were assigned.

NMR experiments were conducted using crypt-222, potassium fluoride, 1a and DMSO-d_6 under stoichiometric conditions. All manipulations were made without using a glovebox, but special care was taken to avoid moisture. Deuterated DMSO of highest quality was used and reaction mixtures were degassed with nitrogen prior to setting up the reaction. A mild stream of nitrogen was maintained throughout the reaction.

2.2.2. Experiments for identification of side products

Vinyl tosylate 1a (15 mg, 68 µmol), crypt-222 (25.6 mg, 68 µmol, 1 equiv.) and KF (3.95 mg, 68 µmol, 1 equiv.) were dissolved in DMSO-d_6 and mixed until a homogeneous solution was obtained. 4-*tert*-butylbenzotrifluoride (1 uL) was added as internal

standard, the vial was capped and shaken well. The reaction mixture was purged with nitrogen 6.0 and additives were added prior to transfer of the reaction vessel to a heating block at 85 ^{0}C. (see table S 2 for details). The reaction mixture was heated for the time indicated in table S2 with a constant, low flow of nitrogen after which NMR spectra were recorded.

We did not optimize the reaction under stoichiometric conditions.

Our attempts to prepare reactive $[K^{+}\subset K_{222}]F^{-}$ from crypt-222 and KF_{aq} were of little practical use, in most cases an oil was obtained even after 5 successive cycles of MeCN addition and evaporation. We ascribed the problem to the much higher affinity of KF for water and decided to work with dry KF-crypt-222. Fortunately, the obtained solution lead to formation of product as well as several postulated side products in the NMR spectrum.

As indicated in the main text, we were primarily interested in determining the mechanism of protonation, however, surprisingly both carbaldehyde proton as well as difluoromethyl protons were observed in the spectrum together with TsF.

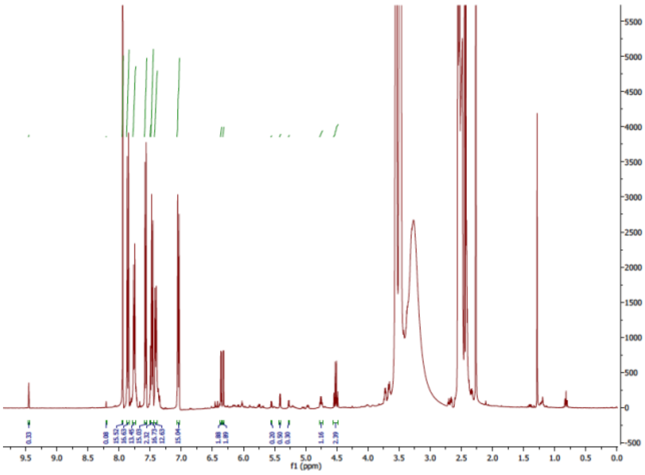

Entry	^{18}F	Substrate %	Product	% aldehyde
1	$CDCl_3$ quenched	0,45	0,26	0,29
2	MeOD 10µl	0,00	0,80	0,20
3	TEMPO	0,20	0.40	0.4
	^{1}H	sm	Product	aldehyde
1	$CDCl_3$ quenched	0,63	0,20	0,17
2	MeOD 10µl	0	0,45	0,55
3	TEMPO	0.5	0.25	0.25

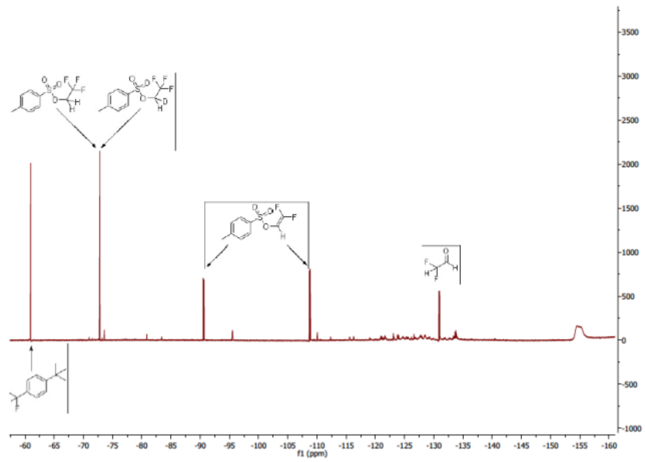

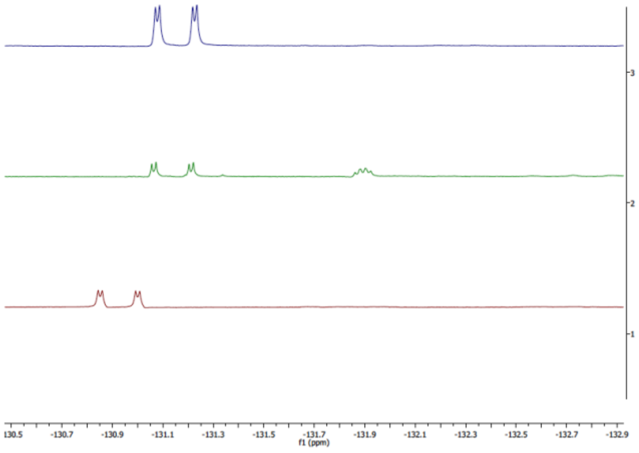

Figure S2: Top left : ^{1}H-NMR of a sample containing 1a, crypt-222 and KF in DMSO-d_6 after one hour of heating at 85 °C. The signal at 9.4 ppm is the carbonyl proton on difluoroacetic aldehyde, the signals at (and at 1.4) are proton resonances of the internal standard, the signal at 6.7 ppm represents the starting material 1a, the triplett of doublettes at ppm is trifluoroacetic aldehyde, and the signal at 4.7 ppm represents product **1b.** Top right: Degree of deuteration and product distribution by NMR.

Bottom left: ^{19}F-NMR (F-decoupled) of a sample containing 1a, crypt-222, KF in DMSO-d_6 after one hour of heating at 85 °C. Signals were assigned to the structures indicated in boxes: From the left the signal at ppm represents the internal standard, the signal at represents the product 1b, the doublett at −81.8 originates from trifluoroacetic aldehyde, the pair of signals at ppm and ppm represents the starting material 1a and the signal at 131.15 ppm represents difluoroacetic aldehyde. Bottom right: ^{19}F-NMR (F-decoupled) of a samples containing 1a, crypt-222, KF in DMSO-d_6 after one hour of heating at 85 °C. Blue trace: The reaction was quenched with D_2O. Green trace: The reaction profile when conducted in presence of MeOD; Red trace: The reaction profile in presence of TEMPO. Signals were assigned to difluoroacetaldehyde.

A carbonyl proton was observed at ppm, the signal intensity correlated with that of a concomitant difluoromethyl group (td at ppm) in all samples, albeit partial deuteration confounds integration to some extend. The ^{19}F spectrum shows the corresponding coupling pattern of the germinal difluoride. This combination of signals is indicative of difluoroacetaldehyde. Trifluoroacetaldehyde was also detected as a minor component of the reaction mixture.

2.2.3. Degree of deuteration and reaction kinetics

The degree of deuteration in each product in linked to the reaction mechanism. None of the components in the reaction is acidic enough to protonate the postulated intermediate carbanion. However, radical mechanisms can lead to abstraction of hydrogen atoms from the aminopolyether crypt-222 and the toluenesulfonate methyl group. The table in Figure S2 nicely illustrates the effect, with apparent deuteration obscuring the true yield of product 1b through deuteration.

It should be noted that it is not appropriate an assumption to translate directly the outcome under stoichiometric conditions to n.c.a radiochemistry. This is due to the reversibility of the initial reaction step, which will be affected by the concentration of fluoride ion. There is a 10^4-fold higher concentration of fluoride ion in these NMR experiments compared to n.c.a radiochemistry, which may lead to a higher absolute concentration of carbanion at any stage of the reaction, leading to alternative pathways of reaction.

The starting, material is consumed quickly during the reaction, in the presence of an alcohol as proton source, the starting material is completely converted within 60 minutes, which is indicative of extremely fast reaction kinetics. Aldehyde is a mere side product under these conditions with 20% relative to 80% 1b. TEMPO shifts the yield towards aldehyde as expected.

2.3. Concentration of the labelled product in reaction mixtures and in formulation:

Concentrations of the final product in representative reaction mixtures and in formulated radioactive products were determined by HPLC. Linearity of the HPLC UV absorbance signal versus concentration of non-radioactive reference was validated with 6 concentrations between 0.3 nmol/mL and 30 nmol/mL. The area of the UV absorbance peak was converted into mass of the carrier. Molar activity (Ci/µmol; MBq/nmol) was then computed as the ratio of radioactivity in the injected sample to the amount of injected substance (µmol), corrected for physical decay to the end of synthesis. These experiments illustrate that the [^{18}F]TsF is not diluted by additional ^{19}F under oxidative conditions.

Table S2: Analysis of the potential molar activity: Representative starting activity, volume of target employed, yield, mass concentrations and molar concentrations of radiolabelled products.

Product	Reaction conditions			radiochemical yield	
	reagents	RCY %	V_{final} mL	c_{mass} µg×mL^{-1}	c_{molar} nmol×mL^{-1}
[^{18}F]**TsF**	N_2	5	1	21	85
	N_2, *i*-PrOH,	3	0.75	43	171
	N_2, $AgNO_3$	50	1	0.76	3
	**N_2, CuOAc, TEMPO**	**66**	**0.75**	**7.8**	**3.1**
	Air, $CuBH_4$	8	0.75	19	76
	N_2, RhCl	65	0.75	0.6	2.4
[^{18}F]**1b**	N_2	62	1	10	38
	N_2, *i*-PrOH,	59	0.75	21	85
	N_2, $AgNO_3$	11	1	1.8	7
	**N_2, CuOAc, TEMPO**	**5**	**0.75**	**0.9**	**4**
[^{18}F]**2b**	N_2	5	0.75	1	4.4
	N_2, CuOAc, TEMPO	3	0.75	0.95	3.7
	**N_2, $CuBH_4$**	**81**	**0.75**	**365**	**1500**
	N_2, RhCl	7	0.75	1	3.8

2.4. Screening of Tosyl sources

To investigate whether the soft, Lewis acidic Cu^{I} cation may activate the toluenesulfonyl group leading to directed polarisation followed by nucleophilic attack of fluoride ion on the S^{V} centre we investigated a small range of available substrates.

2.4.1. Experimental procedure for sulfonyl activation studies:

Copper fluoride triphenyl phosphine complex (1mg, 3 µmol) was combined with a stoichiometric amount of substrate (3µmol, 1 equiv.) in a 3mL v-bottom reaction vessel. An aliquot (250µL) of radionuclide stock solution was added and the mixture was heated to 85 °C for 5 minutes. The reaction vessel was removed from the heat source and the reaction was quenched with MeCN-H_2O (500 µL). The outcome of the reaction was determined by radioTLC and radioHPLC. Product identity was confirmed by injection of the respective reference compound.

Table S3. Screening of substrates for **Ts[^{18}F]F synthesis in the presence of (Ph_3P)$_3$CuF**

Compound	reaction conditions	RCY/ %
	reagents	Ts[^{18}F]F
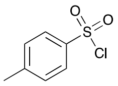	K_2CO_3, crypt-222, DMSO, 85 °C, 5 min, CuF x Ph_3P, Air	3.9
	K_2CO_3, crypt-222, DMSO, 85 °C, 5 min, Air	22.5
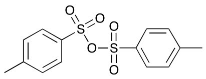	K_2CO_3, crypt-222, DMSO, 85 °C, 5 min, CuF x Ph_3P, Air	2.7
	KOAc, crypt-222, DMSO, 85 °C, 5 min, CuF x Ph_3P, Air	0.7
	KOAc, crypt-222, THF, 66 °C, 5 min, CuF x Ph_3P, Air	trace
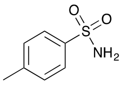	K_2CO_3, crypt-222, DMSO, 85 °C, 5 min, CuF x Ph_3P, Air	0.5
	KOAc, crypt-222, DMSO, 85 °C, 5 min, CuF x Ph_3P, Air	trace
	KOAc, crypt-222, THF, 66 °C, 5 min, CuF x Ph_3P, Air	no reaction
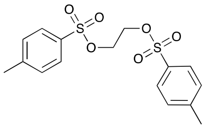	K_2CO_3, crypt-222, DMSO, 85 °C, 5 min, CuF x Ph_3P, Air	3.5
	KOAc, crypt-222, DMSO, 85 °C, 5 min, CuF x Ph_3P, Air	1.2
	KOAc, crypt-222, THF, 66 °C, 5 min, CuF x Ph_3P, Air	not formed
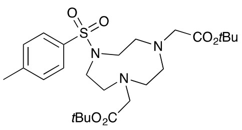	K_2CO_3, crypt-222, DMSO, 85 °C, 5 min, CuF x Ph_3P, Air	1.7
	KOAc, crypt-222, DMSO, 85 °C, 5 min, CuF x Ph_3P, Air	trace
	KOAc, crypt-222, THF, 66 °C, 5 min, CuF x Ph_3P, Air	no reaction
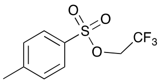	K_2CO_3, crypt-222, DMSO, 85 °C, 5 min, CuF x Ph_3P, Air	trace
	KOAc, crypt-222, DMSO, 85 °C, 5 min, CuF x Ph_3P, Air	not formed
	KOAc, crypt-222, THF, 66 °C, 5 min, CuF x Ph_3P, Air	not formed
	PIDA, CuI, purified **[^{18}F]1b**	not formed
	TEMPO, CuI, purified [^{18}F]**1b**	not formed
	K_2CO_3, crypt-222, DMSO, 85 °C, 5 min, CuF x Ph_3P, Air	22.7

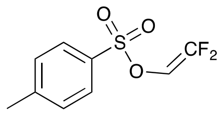	KOAc, crypt-222, DMSO, 85 °C, 5 min, CuF x Ph_3P, Air	6.1
	KOAc, crypt-222, THF, 66 °C, 5 min, CuF x Ph_3P, Air	trace

We found no indication for a Lewis acid assisted cleavage of toluenesulfonyl hetero atom bonds in the experiment above. In presence of Cu^I, only trace amounts of Ts[18F] are formed. As a matter of fact, the reaction in presence of CuF gave less than 20% of the yield obtained in absence of a metal additive (entry 1). Notably, no oxidation product was formed when HPLC purified [^{18}F]1b was reacted with oxidising agents in absence (not shown), or presence of Cu (I).

2.4.2. Screening of additives – broad spectrum screen.

The purpose of the broad-spectrum screen was to identify reagent combinations leading to significant consumption of the radionuclide to improve efficiency and throughput of the investigation.

Since radioHPLC analysis of the reaction mixture takes 20 minutes per run, this experiment was designed to reduce the number of radioHPLC runs to be conducted per screening experiment. Reactions were analysed by radioTLC using a combination of two systems per experiment, one system (selected from CH_2Cl_2-MeOH or $CHCl_3$) was used to determine the total conversion of fluoride ion into radioactive products. The second system (selected from 7.5% EtOAc or 20% Et_2O in Hexane) was used to get an indication on product distribution).

TLCs were read out using two complementary detectors, low resolution profiles were recorded using a photon detector system (raytest miniGITA), high resolution profiles were obtained using positron sensitive storage phosphor imaging (GEHC HR screens and a Duerr CR35 bio IP screen reader).

Experiments showing a minimum conversion of 50% of the starting activity into labelled products in duplicate were replicated with full analysis using radioHPLC, LCMS, and radioTLC.

2.4.3. Experimental procedure for reaction scheme 1, addition pathway:

Experiments were conducted in oven dried conical-bottom reaction vessels (3 or 5 mL total volume). Each vessel was charged with labelling precursor (0.5-3 mg), an equal volume of liquid additives and a spatula tip of solid additives. Vessels were numbered with pencil underneath. A radioactivity stock solution was prepared using a variety of pseudocarrier bases (see table S2) in the solvent of choice and purged with gas (table S2) aliquots (250 µL, 20-100 MBq) of the homogeneous solution were dispensed into 6 individual vials. Vials were counted in a dose calibrator, briefly agitated for mixing prior to heating in an aluminium-heating block at the desired temperature.

Six vials were processed in parallel from each stock solution. Reactions under air were conducted in open vessels; reactions involving specific atmospheres were conducted in capped vials with continuous bubbling of gas throughout the reaction time.

Vials were removed from the heating block after 1-6 minutes and quenched via addition of MeCN-H_2O (2:1, 500µL), followed by immediate removal of all needles from the capped vials. Vials were cooled to r.t., opened when necessary and samples were spotted on silica gel coated aluminium TLC plates and left for drying for 5 minutes prior to development in mobile phase filled glass chambers.

Table S4: Results of preliminary screening (TLC results represent relative product distribution in solution):

**Entry**	**Reaction conditions**		**radiochemical yield**	
	**reagents**	substrate	**^{18}F-consumption**	**product distribution S-F:C-F**
1	AgOTs	1a	64±2	n.d.
	AgOTf		9.8±0.5	n.d.
	AgOAc		25±1	n.d.
	$AgSO_4$		75±3	10:3
	AgF		9.8±2	2:25
	AgOAc	2a	27±2	3:2
	$AgSO_4$		77±3	40:3
	AgF		70±3	n.d.
	BH_3 x SMe_2	2a	trace	
	BH_3 x THF		trace	
	BpinH		trace	

	$CuBH_4$ x PPh_3	Air		84.5±2	3:10:1
	$CuBH_4$ x PPh_3	N_2		64.2±2.2	1:1:2
	CuBr			51±1	2:3
	CuCN			64±4	1:2
	CuF x PPh_3			44±3	1:2
	CuI			89.5±4.6	1:5:1
	CuOAc	Air, KOAc		75±0.4	5:1
	$CuOAc_2$				
3	CuOAc	Air,<br>KOAc, TEMPO	1a	80±1.5<br>78±1	15:1<br>10:1
	Cu^I thiophen-2-yl-carboxylate	Air, K_2CO_3		7.5±0.3	
	CuOTf x Ph-H	Air, K_2CO_3		n.d.	
	CuOTf x MeCN4	Air, K_2CO_3		n.d.	
	CuOTf x Ph-Me	Air, K_2CO_3		84±3	1:9
	$CuSO_4$	Air, K_2CO_3		no reaction	
	CuOTf x Ph-Me	Air, K_2CO_3		trace	0:1
	$CuBH_4$ x PPh_3	Air, K_2CO_3		79±3	20:1
	$CuBH_4$ x PPh_3	Air, KOAc		68±1	
	CuBr	Air, K_2CO_3		47±4	5:8
	CuCN	Air, K_2CO_3		68±3	2:3
	CuF x PPh_3	Air, K_2CO_3		56±2	2:1
	CuI	Air, K_2CO_3		48±5	2:3
	CuOAc	Air, K_2CO_3		65±0	60:6:1
	CuOAc	Air, K_2CO_3, 2-propanol		65±0.1	20:1
	CuOAc	K_2CO_3, N_2, TEMPO		87±2	
	CuOAc	K_2CO_3, Air, TEMPO	2a	82±1.4	
		KOAc, Air, TEMPO		80.5±1.2	
	CuOAc	KOBz, Air, TEMPO		77.7±0.4	
	CuOAc	Air, KOAc		51.8±0.3	
	$CuOAc_2$	Air, K_2CO_3		75±3	
	Cu^I thiophen-2-yl-carboxylate	Air, KOAc		10.5±0.7	
	CuOTf x Ph-Me	Air, K_2CO_3		6.5±0.7	1:3
	CuOTf x $MeCN_4$	Air, K_2CO_3		6.7±0.9	1:3
	CuOTf x Ph-H	Air, K_2CO_3		84±3	
	$CuSO_4$	Air, KOAc		0.9±0.1	1:3
	$NiCl_2$ x $(FPMe_3)_2$	Air, K_2CO_3	1a	24±1.1	n.d.
	$NiCl_2$ x $(PMe_3)_2$	Air, K_2CO_3	2a	9±1	n.d.
	Ph_3SiH	Air, K_2CO_3		79±4	2:9:1
	Ph_3SiH	N_2, K_2CO_3		80.2±1	2:1:18
	$-[SiCH_4-O]_n-$	Air, K_2CO_3	1a	84±2.1	2:8:1
	$-[SiCH_4-O]_n-$	N_2, K_2CO_3		87.2±1.4	1:7:5
	RhCl x 3 Ph_3P	K_2CO_3, Air		69.7±0.7	2:20:1
	RhCOCl x 3 Ph_3P	K_2CO_3, Air		n.d.	
	RhCl x 3 Ph_3P	K_2CO_3, Air		82.5±0.5	
	RhCl x 3 Ph_3P	K_2CO_3, Air, 2-propanol	2a	62.6±0.2	25:1
	RhCl x 3 Ph_3P	KOAc, Air		29.5±0.5	
	RhCOCl x 3 Ph_3P	K_2CO_3, Air		85±1.5	

RhCOCl x 3 Ph_3P	KOAc, Air		24±1.7	
TEMPO	n.a.	1a		
TEMPO	n.a.	2a	75.5	
PIDA	n.a.	1a	95.1±4.7	
PIDA	n.a.	2a	86.5±3	
I_2		2a	47	n.d.
ICl		2a	55	n.d.
Ph_3PI_2		1a	85.3±3	n.d.
Ph_3PI_2		2a	80	n.d.
2-NO_2-imidazole	N_2	2a	19	8:5:2
bis 3-ethylhexyl phosphite	N_2, CuOAc, TEMPO	1a	70±3	10:1:36
bis 3-ethylhexyl phosphite	N_2	1a	81±1.3	1:2:34
2-propanol	Air	1a	71.2±2.4	2:1:3
2-propanol	N_2	2a	69.3±4	1:1:6
none	Air	1a	92±3	2:1
none	Air	2a	74±2.2	
none	N_2	1a	93±3	1:2.5
none	N_2	2a	46.5±5	2:5:1
molecular sieve	Air	2a	20.5±3.2	1:40
Ph_3P	Air	2a	65±3	1:3:1
Bu_3SnCl	N_2, AgOAc	1a	84.5	3:10:1
Bu_6Sn_2	N_2, AgOAc	1a	87	n.d.
$(Bu_3Sn)_2O$	N_2, -$[SiCH_4$-$O]_n$-', Ag	1a	16	5:7:1
H_2	n.a.	1a	87	1:7:5
CO_2	n.a.	1a	83	n.d.

When investigating π-active metal additives, the aerobic oxidation mechanism became increasingly apparent. Therefore, the screen was extended to oxidising and reducing agents as well as sources of hydryl radicals.

With respect to the latter, we reasoned that the relative reaction velocity of SET oxidation allows for suppression of elimination following fluoride attack provided a radical source is added to the solution.

Conditions for n.c.a radiofluorination are characterised by a stoichiometric deficit of [^{18}F]fluoride ion relative to all other constituents. To overcome the low reaction cross section in such high dilution, a pseudocarrier (e.g. potassium carbonate, oxalate or acetate)-phase transfer catalyst mixture is used to form a potassium cryptate complex with fluoride ion ([$K^+ \subset K_{222}$][^{18}F]F^-), which essentially governs the reactivity of the radionuclide. Trace water must be eliminated during the formation of the cryptate complex.

In our hands, potassium carbonate is the reagent showing the best overall performance. However, presence of carbonate ions in solution is problematic when aiming to engage transition metals in the reaction. In theory, silver and copper carbonate species could be formed from most precursors and the solubility of possible species formed is not known. In presence of potassium carbonate the poorly soluble silver carbonate (1:1 molar ratio) is known to become more soluble. 'Basic' Cu^{II}carbonate ($Cu_2CO_3(OH)_2$) is practically insoluble, however, the presence of phase transfer catalysts and other compounds may have a positive effect on the solubility.

2.5. General procedure for radiolabeling (reaction scheme 1, pathway II):

[^{18}F]Fluoride ion was produced using the $^{18}O(p,n)^{18}F$ nuclear reaction via proton bombardment (16.3MeV) of an $H_2{}^{18}O$ liquid target on a GE PETtrace cyclotron at a beam current of 30-40 µA for 5 to 10 minutes. The radionuclide was extracted from the enriched target water via solid phase extraction on a waters accell plus light QMA strong anion exchanger cartridge ($CO_2{}^{2-}$-form). Reactive [^{18}F]F^- was obtained by elution of the cartridge as described below.

Labelling precursor dissolved in the appropriate solvent was added to the residue and heated to the desired temperature. Aliquots were withdrawn from the reaction mixture (100 ml) at multiple timepoints and transferred into a mixture of MeCN-H_2O, 1:1 (1mL).The resultant sample was directly injected into radioHPLC or used for radioTLC.

2.5.1. Extraction, drying and preparation of radioactive fluoride:

A SupelTM-Select SAX SPE (30 mg) cartridge was preconditioned for [^{18}F]fluoride extraction by slowly passing a K_2CO_3 solution (1 M, 5 mL) through it, followed by type I water (10 mL) and and finally air (30 mL) from a syringe. Elution solution was prepared by adding 15µL of 1M

K_2CO_3 to 11.2 mg of Kryptofix 222 and left for 5 minutes followed by addition of 985µL of acetonitrile. An aliquot of the [^{18}F]fluoride in [^{18}O]H_2O was withdrawn into the preconditioned cartridge (0.1-0.6 mL), and air was passed through (10 mL) to remove excess target water. The [^{18}F]fluoride was eluted from the cartridge by passage of elution solution (1 mL) through it, and the eluent was collected into a V-vial. The solvent was then evaporated on a hot plate at 90 °C under an argon stream. Residual water was removed from the [^{18}F]fluoride by additional 3 cycles of addition/evaporation of MeCN (1 mL). The vial was removed from the hot plate, capped and cooled for about 30 seconds on an ice bath.

2.5.2. Degassing of solvent:

All solvents were degassed by taking passing N_2 gas through for 10–15 minutes.

2.6. ^{18}F-labelling of other difluorovinyl-containing compounds by direct method:

The corresponding 2,2-difluorovinyl moiety-containing precursor (17 µmol) was dissolved in 1 mL DMSO. Triethylsilane (17 µmol), (2,2,6,6-Tetramethylpiperidin-1-yl)oxyl or (2,2,6,6-tetramethylpiperidin-1-yl)oxidanyl (TEMPO) (17 µmol) and 2-methylbutan-2-ol (0.9 mmol) were added at room temperature. The mixture was degassed into a screwcap vial by passing N_2 gas for 10 minutes before the start of the labeling reaction. This reaction mixture was added to the dried and cooled [^{18}F]fluoride and the mixture is homogenized for about a minute. Once the reagents have been mixed, the activity vial was measured and placed on the hot plate at 90°C for 6 minutes. The seal of the vial was tightened carefully to avoid evaporation of the reagents. At the end point of the reaction, the reaction vial cooled on ice for 1-2 minutes to stop the further reaction followed by measurement of the activity. 100µL from the reaction vial was transferred to an Eppendorf tube containing 1mL solvent (acetonitrile-water, 1:1), out of which 10µL was injected directly into an analytical HPLC.

2.7. Indirect labeling (alkylation) by direct method

The synthesized 2,2-difluoro-2-(fluoro-^{18}F)ethyl 4-methylbenzenesulfonate ([^{18}F]**1b**) after confirmation from HPLC was used for indirect radiolabeling of different amines/phenols without purification. Piperidines/piperazine based amines (39 µmol) and Cs_2CO_3 (61 µmol) were added to the reaction mixture containing (90%) [^{18}F]**1b**. A magnet bead was added to help dissolving the base in the reaction. After the activity check, the reaction vial was allowed for heating cum stirring for 40 minutes at 140°C. At the end point of the reaction, the reaction vial cooled on ice for 1-2 minutes to stop the further reaction followed by measurement of the activity. 100µL from the reaction vial was transferred to an Eppendorf tube containing 1mL solvent (acetonitrile: water =1:1), out of which 10µL was injected directly into an HPLC.

2.8. Purification of trifluoroethyl amines

SCX cartridge (Merck) was conditioned by passing 5 mL 1M HCl through followed by washing with water until the elute was neutral. For the purification of the different trifluoroethyl amines, the reaction mixture was diluted with water and neutralized using 1M HCl. The reaction mixture was passed through preconditioned SCX cartridge. The cartridge was washed with water. The trapped product was eluted with 5mL Dulbecco's PBS (10x), 10µL out of which was injected into HPLC for QC. The radiochemical yield was determined by decay correction of the counted activity to the start of synthesis using Rad pro Calculator.

2.9. Calculation of radiochemical yield (RCY)

Fluoride adsorption by a glass reaction vial, HPLC stationary phase and evaporation of volatiles from TLC plates are the unavoidable reasons for direct assessment of radiochemical yield by HPLC and TLC. Despite the challenges for the practicality of the %activity isolated at the end of a radiotracer production; it provides a better outcome for screening. The % of activity in the product fraction at the end of syntheses was used to calculate the isolated radiochemical yield. For ease of screening, unreacted fluoride was separated from labelled products via solid phase extraction. At the end of the reaction, the total activity was compared to the activity of the pure extracted product by counting of the vials. Different HPLC systems (mentioned below) were used for quality control, confirm, identify and determine radiochemical purity of the products. Traces of the product in the SCX cartridge and the aqueous phase after extraction were neglected. The activity corresponding to the product was divided by the total activity at this time point and multiplied with 100% to give the RCY (Equation 1).

$$RCY\ (\%) = \frac{(RCP\ .\ isolated\ activity)}{Total\ activity}.100\%$$

Equation S1. Method for the calculation of RCY. *RCP* – radiochemical purity of the product.

2.10. Purification of product 8b using Seimipreperative HPLC

After the production of crude **8b**, the product was diluted with 5 mL MeCN-water; 30:70 and syringe filtered to remove any solids wastes. A semipreparative Luna PFP column (Phenomenex; 5 µm, 100 Å, 250 mm × 10 mm) with an isocratic mixture of MeCN-water; 30:70 was used at a flow rate of 5 mL/min for HPLC purification. The idea of product containing HPLC fractions was obtained roughly by dose meter. Later the fractions were checked using analytical HPLC for quality control. >99% pure fractions of **8b** were obtained and was counted using a dose calibrator. The radiochemical yield was determined by decay correction of the counted activity to the start of synthesis.

2.11. Determination of molar activity of Seletracetam (8b)

Molar activity was determined via HPLC (UV detection, 254 nm). In brief, linearity of the UV detector response (calibration curve) was confirmed for the range of concentrations expected for the formulated radiotracer using 5 concentrations (1 µg/mL, 10 µg/mL, 100 µg/mL,

1000 µg/mL and 10000 µg/mL). The product was counted in a dose calibrator and the concentration of the product was determined by HPLC. Activity at the start of the reaction was 12.22 GBq and the activity at the end for the pure product was 1.52 GBq. Molar concentration was 0.0153 µmol/mL.Finally, the activity was divided by the molar concentration of the product to obtain the molar activity. The calibration curve used to determine the molar activity of Seletracetam is as under:

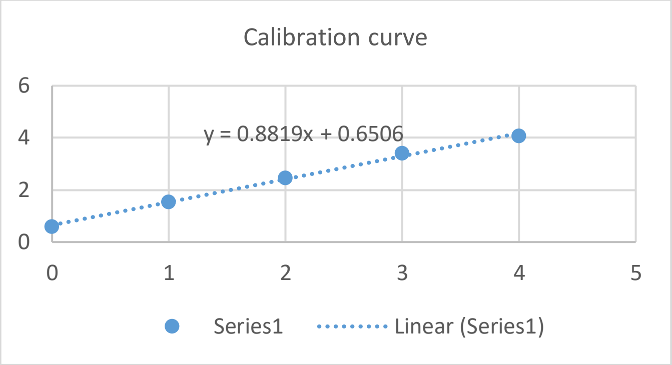

2.12. *In vitro* quantitative stability checks for [^{18}F]2a

Following preparation of the radionuclide stock solution, **2a** was added heated to 85 °C for 5 minutes followed by the addition of two volumes of MeCN-H_2O 2:1. The mixture was filtered and purified by HPLC and isolated via solid phase extraction to furnish a clear, colourless solution of Ts[^{18}F]F (0.32 µg/mL) formulated in Dulbeccos phosphate buffered saline containing 1% DMSO. As shown by radioHPLC analysis the product was obtained in a chemical purity >99% and a radiochemical purity >99%. The product was incubated with pig liver esterase, lipase from pig liver, bovine serum albumin and neat buffer for 4 hours. The mixtures were filtered using protein recovery membranes with an exclusion limit of 10 kDa and the protein residue on the filter was counted in a well counter. The filtrate was loaded on solid phase extraction cartridges, vacuum was applied to pass the mixture through the cartridge and collect the filtrate in separate 1 mL PE tubes. Tubes and cartridges were counted, and the filtrate was analysed by radioTLC.

3. Supplementary results and discussion

3.1. Screening of different reaction conditions to optimize the synthesis of [^{18}F]1b

Replacement of DMSO with other solvents like DMF, MeCN and NMP did not provide the product in considerable yields. Different reaction times were tried for the labeling of difluorovinyl compounds; however 6 min was found to constitute an optimal reaction time. More reaction time like 10, 20 and 30 minutes were found to have no effect on the progress of the reaction. Addition of 2-propanol and/or 1,1,1,3,3,3-hexafluoro-2-propanol in place of 2-methyl-2-butanol as H-source were not found favorable for the good yields. Different metal additives like Cu (II) and Ag (II) were also tried but not found to produce a measureable effect. Other than Kryptofix 222, bases like K_2CO_3, KH_2PO_4, KOTf and NaOTf were used to make the eluent for getting [^{18}F]fluoride from [^{18}O]H_2O, however the former was found to work best. Different proportions of 2-methyl-2-butanol were also evaluated (see table S5).

Table S5: Different reaction conditions for the synthesis of [^{18}F]**1b**.

Entry	Additive	Time (min)	RCY (%)		
			**[^{18}F] TsF**	**[^{18}F]1a**	**[^{18}F]1b**
1.	Difluorovinyl tosylate (**1a**), TEMPO, Et_3SiH, DMSO, 90°C, 2-methyl-2-butanol	1	24.20±1	16.60±2	19.82±3.5
2.		2	6.38±3	6.60±3	28.02±2
3.		4	14.55±0.6	22.99±2.2	34.98±1.2
4.		**6**	3.04±0.8	0±1	86.71±1
5.		10	10.44±2	11.09±1	74.89±1
6.		20	8.85±3	9.73±2.6	78.46±1.5
7.		30	7.00±0.9	3.43±2	84.05±2.8
8.	**1a**, TEMPO, Et_3SiH, DMSO, 90°C, 2-propanol	1	53.89±1	1.37±1	10.15±2
9.		2	60.24±0.6	2.13±2.1	16.65±1
10.		4	60.65±3	3.37±3.2	23.08±2
11.		**6**	15.80±1	8.22±1	74.04±3.6
12.	**1a**, TEMPO, Et_3SiH, DMSO, 90°C, 1,1,1,3,3,3-hexafluoro-2-propanol.	6	7.02±0.8	1.17±0.7	0.95±2.2

		**Additive**			
13.	**1a**, TEMPO, Et3SiH, DMSO, 90°C, 2-propanol, 6 min	Ag (II)	22.06±0.4	7.24±0.8	61.30±2.2
14.	**1a**, Et_3SiH, DMSO, 90°C, 2-propanol, 6 min	Ag (II)	37.30±1	6.87±1	50.34±1
15.	**1a**, Et_3SiH, DMSO, 90°C, 6 min	Ag (II)	37.91±1	28.43±0.3	6.54±2.4
16.	**1a**, TEMPO, Et_3SiH, DMSO, 90°C, 6 min	Ag (II)	25.81±4	48.13±0.9	19.83±1.3
17.	**1a**, $(Bu)_4SnH$, TEMPO, DMSO, 90°C, 6 min	Ag (II)	4.96±1	3.61±1	2.76±4
18.	**1a**, DMSO, 90°C, 6 min	-	34.25±3.2	31.42±1	22.11±1.1
19.	**1a**, DMSO, 90°C, 2-propanol, 6 min	-	16.48±0.7	5.96±2.7	52.61±1
20.	**1a**, DMSO, 90°C, Et_3SiH, 6 min	Cu (II)$(Py)_4$OTf	3.56±1	1.11±3.2	1.06±3
21.	**1a**, DMSO, 90°C, $(Bu)_4SnH$, 6 min	Cu (II)	2.26±1	0±1	0±2.8
22.	**1a**, TEMPO, Et_3SiH, DMSO, 90°C, 6 min	Cu (II)	1.26±1.5	0±1.8	0±4
23.	**1a**, TEMPO, $(Bu)_4SnH$, DMSO, 90°C, 6 min	Cu (II)	1.37±4	0.90±2	1.52±1.2
		**Bases used in the eluent**			
24.	**1a**, TEMPO, Et_3SiH, DMSO, 90°C, 2-methyl-2-butanol, 6 min.	K_2CO_3	3.04±0.8	0±1	86.71±1
25.		KH_2PO_4	30.52±1	14.06±2	37.69±2
26.		KOTf	33.74±0.8	14.55±1	41.29±1.3
27.		NaOTf	26.17±2	19.61±0.9	47.66±4
		**Amount of 2-methyl-2-butanol (mL)**			
28.	**1a**, TEMPO, Et_3SiH, DMSO, 90°C, 6 min.	**0.1**	3.04±0.8	0±1	86.71±1
29.		0.2	14.58±2	8.98±0.6	70.98±1.1
30.		0.3	20.34±1	13.41±2	60.62±3
31.		0.4	12.99±2.6	12.03±3	73.89±2.8
32.		0.5	20.24±1	10.0±0.8	68.42±1.1
33.		1 mL, without DMSO	55.40±1	0±2	36.24±1.8
34.	**1a**, N_2 DMSO, 90°C, 6 min.	$AgNO_3$	50.1±4	22.5±3	10.8±3

3.2. Reaction optimization conditions for labelling substrate 2a by direct method

In the presence of air, a control reaction furnished 46% of [^{18}F]TsF, and double figured yields pertained even under nitrogen atmosphere and with 2-propanol in the reaction mixture (Table S6 entries 2–4). This is in stark contrast to substrate **1a**, which would produce only one third of the [^{18}F]TsF under air and less than 5% under nitrogen atmosphere. Moreover, air alone furnished the desired product in a higher yield than $AgNO_3$ and CuCN, which gave 34 and 37%, respectively (Table S6, entries 4 and 5). The TsF yields increased beyond the metal free control reaction when cupric acetate was used in conjunction with air both with (55%) and without 2-propanol (67%) (Table S6 entries 6 and 7). Highest [^{18}F]TsF yields were obtained with cuprous acetate in the presence of air (77%) (Table S6 entry 8). The CuOAc-TEMPO combination performed equally well, although a somewhat higher standard deviation can be observed (Table S6 entry 9). Copper triflate furnished the desired product in about 60% yield (Table S6 entry 10). Cupric borohydride performed equally well in suppressing the oxidative degradation of the starting material with **2b** being formed as the major product (80%) (Table S6 entry 11).

Table S6: Additive effect observation for ^{18}F-labelling of substrate 2a using reaction scheme 1.

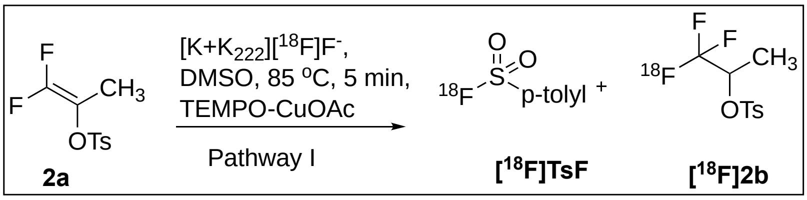

Entry (substrate)	Additive	RCY (%)		
		**[^{18}F] TsF**	**[^{18}F]2a**	**[^{18}F]2b**
1 (**2a**)	Air	46.4±5	20.2±3	2.9±0.5
2 (**2a**)	N_2	25.7±0.8	17.6±2	5.2±3
3 (**2a**)	N_2, *i*-PrOH	14.8±1	22.7±2	25.4±2
4 (**2a**)	N_2, $AgNO_3$	33.9±0.5	38.2±3	16.9±2
5 (**2a**)	CuCN, Air	37±1	3.8±1.0	0-1%

6 (**2a**)	CuOAc, *i*-PrOH, Air	55.7±2	4.1±0.5	2.2±0.2
7 (**2a**)	CuOAc, Air	67.1±3.2	11.5±1.1	2.7±1
8 (**2a**)	$Cu(OAc)_2$, Air	77.1±1	13.6±1	-
9 (**2a**)	N_2/CuOAc TEMPO	69.4±5	5.5±0.2	-
10 (**2a**)	CuOTf<br>Air	59.5±2.1	11.6±1.1	3.8±0.4
11 (**2a**)	$CuBH_4$	0±0.3	4.9±1	80.4±3

3.3. Conditions for the synthesis of 1-(2,2-difluoro-2-(fluoro-^{18}F)ethyl)-4-(4-methoxyphenyl)piperazine (7b).

After the optimization of the direct labeling method, different reaction conditions were tried for the development of 1-(2,2-difluoro-2-(fluoro-^{18}F)ethyl)-4-(4-methoxyphenyl)piperazine (**7b**) through indirect labeling route. Highest yield of 44% was obtained at 140˚C, which was found to be less at lower ranges and degrade at higher ranges of temperature. Same way, different reaction times were examined from which 40-minute reaction time was observed to produce highest amount of the desired product. In addition, different bases were tried for the alkylation reaction like, NAH, pyridine DIPEA, K_2CO_3, KH_2PO_4, KOTf, NaOTf and Cs_2CO_3, among which the latter was found to work best. Combinations of K_2CO_3 and Cs_2CO_3 with KI and NaI respectively were found to produce no product. The reaction was found to produce no product without any bases (see table S5).

Table S7: Different reaction conditions for the development of 1-(2,2-difluoro-2-(fluoro-^{18}F)ethyl)-4-(4-methoxyphenyl)piperazine (7b).

Entity	Conditions	Temperature (°C)	RCY (%)			
			1-(2,2-difluoro-2-(fluoro-^{18}F)ethyl)-4-(4-methoxyphenyl)piperazine **(7b)**	[^{18}F] TsF	[^{18}F]**1a**	[^{18}F]**1b**
1.	[^{18}F]difluoro ethyltosylate **([^{18}F]1b)**, 1-(4-methoxyphenyl)piperazine, DMSO, Cs_2CO_3, 40 min.	130	38.02±2	0±1	0±1.9	26.77±1
2.		140	43.67±2.1	0±0.8	0±1	8.20±1.6
3.		150	29.08±1	0±1.1	0±1.6	1.05±3
4.		170	23.01±2	0±2	0±1.8	0±2
		**Reaction time (min)**				
5.	[^{18}F]difluoro ethyltosylate **([^{18}F]1b)**, 1-(4-methoxyphenyl)piperazine, DMSO, Cs_2CO_3, 140°C.	10	7.31±1	0±2	0±1.7	71.66±1.9
6.		30	26.81±1.7	0.46±0.6	0±1.1	33.16±2
7.		40	43.67±2.1	0±0.8	0±1	8.20±1.6
		**Base (2.3 eq.)**				
8.	[^{18}F]difluoro ethyltosylate **([^{18}F]1b)**, 1-(4-methoxyphenyl)piperazine, DMSO, 40 min, 140°C.	No base	NP	15.90±3	13.42±3	60.77±2.2
9.		DIPEA	NP	10.18±2	16.94±1.2	65.28±1.1
10		KH_2PO_4	NP	9.74±2	17.66±2	65.08±1
11		KOTf	NP	27.53±2.8	15.71±1	51.39±3
12		NaOTf	NP	16.21±1	16.22±1.8	61.64±2
13		Cs_2CO_3	43.67±2.1	0±0.8	0±1	8.20±1.6
14		K_2CO_3	0±1.2	21.07±1.8	5.42±1.0	57.37±2.8
15	[^{18}F]difluoro ethyltosylate **([^{18}F]1b)**, 1-(4-methoxyphenyl)piperazine, DMF, 15 min, 120°C.	NaH	NP	0±1.6	0±0.6	0±0.9
16		DIPEA	NP	16.83±2	16.76±1	60.50±1.1
17		Pyridine	NP	11.76±1	15.98±2	68.72±2.3
18		Cs_2CO_3	NP	36.83±4	12.94±2	47.70±2.1
19		K_2CO_3	NP	6.33±1	0±1.3	84.55±1
20		K_2CO_3 +KI	NP	1.54±0.9	0±1	88.22±1
21		Cs_2CO_3 +KI	NP	1.86±0.6	0±1.2	86.89±3.2
22		K_2CO_3 +NaI	NP	13.79±1	13.78±1	68.78±1.3
23		Cs_2CO_3 +NaI	NP	10.68±1.2	5.46±2	77.88±2
24		No base	NP	13.32±2	9.86±4	72.90±2

NP = No Product

3.4. General synthesis of precursors and their reference molecules:

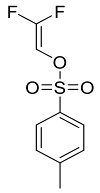

2,2-difluorovinyl 4-methylbenzenesulfonate (1a)[1]: 2,2,2-trifluoroethyl 4-methylbenzenesulfonate **(1b)** (1.3 g, 5.1 mmol) was dissolved in anhydrous THF (8 ml) and the mixture was cooled to -78°C. n-Butyl lithium (1.6 M in hexanes, 4 mL, 10.2 mmol) was added dropwise and the resultant mixture was stirred at -78°C for 40 minutes. A mixture of water (2.27 g, 12.7 mmol) and THF (5 ml) was added and the mixture was allowed to warm to room temperature. The organic phase was obtained in diethyl ether and dried over Na_2SO_4 and concentrated. The residue was purified by flash chromatography on silica gel (7% diethyether in hexanes). 1.2 g of a colorless liquid **(1a)** was obtained for further radiolabeling experiments. ^{1}H NMR (400 MHz, Chloroform-*d*) δ 7.87 – 7.80 (m, 2H), 7.44 – 7.36 (m, 2H), 6.09 (dd, *J* = 14.3, 3.9 Hz, 1H), 2.49 (s, 3H). ^{13}C NMR (101 MHz, $CDCl_3$) δ 159.89, 157.06, 156.95, 154.13, 146.16, 131.21, 130.08, 128.41, 101.26, 101.11, 100.67, 100.52, 77.37, 77.05, 76.74, 21.73. ^{19}F NMR (376 MHz, Chloroform-*d*) δ -90.39 (d, *J* = 50.8 Hz), -109.03 (d, *J* = 50.7 Hz). MS (ESI), $[M+Na]^+$ = 257.005, $C_9H_8F_2O_3S$ requires 234.016.

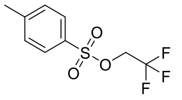

Synthesis of 2,2,2-trifluoroethyl 4-methylbenzenesulfonate (1b)[1]: 2,2,2-trifluoroethanol (1g, 10 mmol) and triethylamine (3.2 mL, 23 mmol) were dissolved in dichloromethane (DCM) (100 ml). Toluenesulfonyl chloride (1. 5g, 8 mmol) was added in portions at 0°C. The mixture was stirred for approximately 4 hours at room temperature, until the toluenesulfonyl chloride had been consumed. The reaction mixture was diluted with 100 mL DCM and water. The organic phase was obtained in DCM, which was washed with ice cold water (200 mL x3) in order to get rid of the excessive trimethylamine, followed by a wash with saturated solution of $NaHCO_3$ and brine. The organic phase was dried over Na_2SO_4 and filtered. The filtrate was concentrated in vacuo. Pure white crystals of 2a were obtained without any purification. ^{1}H NMR (400 MHz, Chloroform-*d*) δ 7.91 – 7.75 (m, 2H), 7.40 (t, *J* = 7.9 Hz, 2H), 4.36 (p, *J* = 7.5 Hz, 2H), 2.48 (q, *J* = 4.7, 4.0 Hz, 3H). ^{13}C NMR (101 MHz, $CDCl_3$) δ 145.98, 131.81, 130.17, 128.09, 123.28, 120.52, 65.09, 64.71, 64.34, 63.96, 21.71. ^{19}F NMR (376 MHz, $CDCl_3$) δ -73.84, -73.86, -73.88. MS (ESI), $[M+Na]^+$ = 277.011, $C_9H_9F_3O_3S$ requires 254.022.

3.4.1. General procedure for the conversion of aldehydes into 2’,2’-difluorostyrenes: [1,6]

Sodium 2-chloro-2,2-difluoroacetate (2 eq.) was dissolved in DMF at room temperature. Triphenyl phosphine (2 eq.) and aldehyde (1 eq.) were added to the reaction mixture one by one. The reaction was stirred and heated slowly upto 100˚C until CO_2 evolution stops. The reaction was cooled to room temperature followed by the addition of methyl iodide (2.5 eq.) was added to the reaction inorder to consume the unreacted triphenyl phosphine. 50–100 mL was added to the reaction mixture and the organic phase extracted with ethyl acetate (50 mL x 3). The organic layer was dried over Na_2SO_4 and concentrated. The difluorovinyl compounds **(4a–7a)** were purified by column chromatography using ethyl acetate in hexanes (20–30%) as colorless liquids.

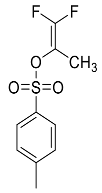

1,1-difluoroprop-1-en-2-yl 4-methylbenzenesulfonate (2a)

Synthesized according to the method described in the literature [1]. Colorless liquid, ^{1}H NMR (400 MHz, Chloroform-*d*) δ 7.86 (d, *J* = 8.3 Hz, 2H), 7.39 (d, *J* = 8.1 Hz, 2H), 2.49 (s, 3H), 1.95 (t, *J* = 4.2 Hz, 3H).^{13}C NMR (101 MHz, $CDCl_3$) δ 145.76, 132.56, 129.90, 128.34, 77.34, 77.02, 76.71, 21.76, 13.13. ^{19}F NMR (376 MHz, Chloroform-*d*) δ -94.69 (q, *J* = 4.1 Hz), -94.83 (q, *J* = 4.1 Hz), -108.26 (q, *J* = 4.4 Hz), -108.40 (q, *J* = 4.1 Hz). MS (ESI), $[M+Na]^+$ = 271.021, $C_{10}H_{10}F_2O_3S$ requires 248.032.

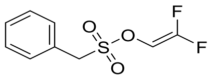

2,2-difluorovinyl phenylmethanesulfonate (3a)

Synthesized as per the reported literature [1]. White solid powder. ^{1}H NMR (400 MHz, Chloroform-*d*) δ 7.45 (s, 5H), 6.09 (dd, *J* = 14.2, 3.8 Hz, 1H), 4.51 (s, 2H). ^{13}C NMR (101 MHz, $CDCl_3$) δ 159.24, 130.73, 129.61, 129.14, 126.37, 101.98, 101.83, 101.37, 101.22, 77.34, 77.03, 76.71, 56.96. ^{19}F NMR (376 MHz, $CDCl_3$) δ -89.93, -89.95, -89.97, -90.07, -90.10, -90.11, -109.85, -109.86, -109.99, -110.01. MS (ESI), $[M+Na]^+$ = 257.005, $C_9H_8F_2O_3S$ requires 234.016.

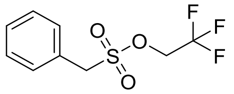

2,2,2-trifluoroethyl phenylmethanesulfonate (3b)

Synthesized as per the reported literature [1]. White solid powder. ^{1}H NMR (400 MHz, Chloroform-*d*) δ 7.45 (s, 5H), 4.50 (s, 2H), 4.26 (q, *J* = 7.9 Hz, 2H). ^{13}C NMR (101 MHz, $CDCl_3$) δ 130.76, 129.61, 129.17, 126.68, 77.34, 77.03, 76.71, 65.11, 64.73, 64.35, 57.73. ^{19}F NMR (376 MHz, $CDCl_3$) δ -74.11, -74.13, -74.15. MS (ESI), $[M+Na]^+$ = 277.011, $C_9H_9F_3O_3S$ requires 254.022.

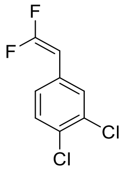

1,2-dichloro-4-(2,2-difluorovinyl)benzene (4a)

Synthesized from 3,4-dichlorobenzaldehyde (1 g, 5.7 mmol), triphenylphosphine (3 g, 11.4 mmol), sodium 2-chloro-2,2-difluoroacetate (1.74 g, 11.4 mmol) in 15 mL DMF at 100 ˚C for 3 hours. At the end of the reaction, the reaction was cooled to room temperature and MeI (2.28 mL, 14.2 mmol) was added to consume the excessive triphenylphosphine. The product was purified via column chromatography using diethyl ether in hexanes (1%). Colorless oil (0.61 g, yield 51.4%) was obtained. ^{1}H NMR (400 MHz, Chloroform-*d*) δ 7.47 – 7.39 (m, 2H), 7.17 (dd, *J* = 8.4, 1.9 Hz, 1H), 5.24 (dd, *J* = 25.5, 3.4 Hz, 1H). ^{13}C NMR (101 MHz, $CDCl_3$) δ 159.51, 156.62, 156.54, 153.65, 132.85, 130.98, 130.96, 130.93, 130.58, 130.50, 130.44, 130.43, 130.37, 129.28, 129.24, 129.21, 129.18, 126.82, 126.79, 126.75, 126.72, 81.09, 80.95, 80.78, 80.65, 77.34, 77.02, 76.70, 31.61, 26.93, 22.67, 14.11. ^{19}F NMR (376 MHz, Chloroform-*d*) δ -80.03 (d, *J* = 26.4 Hz), -81.83 (d, *J* = 26.4 Hz). MS (ESI), $[M+1]^+$ = 208.002, $C_8H_4Cl_2F_2$ requires 207.966.

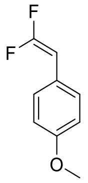

1-(2,2-difluorovinyl)-4-methoxybenzene (5a)

Synthesized from anisaldehyde (1 g, 7.3 mmol), triphenylphosphine (3.8 g, 14.5 mmol), sodium 2-chloro-2,2-difluoroacetate (2.23 g, 14.6 mmol) in 15 mL DMF at 100 ˚C for 3 hours. At the end of the reaction, the reaction was cooled to room temperature and MeI (1.15 mL, 18.3 mmol) was added to consume the excessive triphenylphosphine. The product was purified via column chromatography using diethyl ether in hexanes (1%). Colorless oil (0.5 g, yield 40%) was obtained. ^{1}H NMR (400 MHz, Chloroform-*d*) δ 7.29 (d, *J* = 8.8 Hz, 2H), 6.91 (d, *J* = 8.8 Hz, 2H), 5.25 (dd, *J* = 26.4, 3.9 Hz, 1H), 3.84 (s, 3H). ^{13}C NMR (101 MHz, $CDCl_3$) δ 158.73, 158.57, 155.89, 155.79, 152.94, 128.84, 128.80, 128.78, 128.74, 122.77, 122.70, 122.64, 114.18, 114.18, 81.76, 81.62, 81.47, 81.33, 77.36, 77.04, 76.72, 55.25. ^{19}F NMR (376 MHz, $CDCl_3$) δ -84.60, -84.67, -84.70, -84.77, -86.43, -86.44, -86.53, -86.54. MS (ESI), $[M+1]^+$ = 171.060, $C_9H_8F_2O$ requires 170.054.

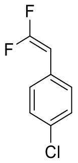

1-chloro-4-(2,2-difluorovinyl)benzene (6a)

Synthesized from p-chlorobenzaldehyde (1 g, 7.1 mmol), triphenylphosphine (3.7 g, 14.10 mmol), sodium 2-chloro-2,2-difluoroacetate (2.1 g, 13,7mmol) in 15 mL DMF at 100 ˚C for 3 hours. At the end of the reaction, the reaction was cooled to room temperature and MeI (1.1 mL, 17.6 mmol) was added to consume the excessive triphenylphosphine. The product was purified via column chromatography using 100% hexanes. Colorless oil (0.55 g, yield 44.5 %) was obtained. ^{1}H NMR (400 MHz, Chloroform-*d*) δ 7.34 (d, *J* = 8.7 Hz, 2H), 7.28 (d, *J* = 8.7 Hz, 2H),

5.27 (dd, J = 25.9, 3.6 Hz, 1H). ^{13}C NMR (101 MHz, $CDCl_3$) δ 168.47, 146.86, 133.31, 129.87, 123.76, 122.08, 91.41, 77.35, 77.03, 76.71, 40.78, 40.43, 40.07, 39.71, 35.93, 30.33, 25.31, 22.24, 13.74. ^{19}F NMR (376 MHz, Chloroform-*d*) δ -68.58, -80.90 (d, J = 33.1 Hz), -95.16 (d, J = 33.2 Hz). MS (ESI), $[M+1]^+$ = 175.003, $C_8H_5ClF_2$ requires 174.001.

3.4.2. Synthesis of the reference compounds

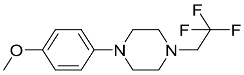

1-(4-methoxyphenyl)-4-(2,2,2-trifluoroethyl)piperazine (7b)

1-(4-methoxyphenyl)piperazine hydrochloride (0.5 g, 1.8 mmol) was dissolved in 20 mL DMF, followed by the addition of potassium carbonate (0.5 g, 3.6 mmol) at room temperature. 2,2,2-trifluoroethyl 4-methylbenzenesulfonate (0.47 g, 1.8 mmol) was added to the reaction mixture. The reaction was allowed to stir under reflux conditions over night. Progress of the reaction was observed by TLC. On completion of the reaction, water (50 mL) was added to the reaction mixture, the organic phase was extracted with ethyl acetate (30 mL x 3) and dried over Na_2SO_4. The crude was purified by column chromatography using ethyl acetate in hexanes (10–20%) to obtained white solid (0,35 g, yield = 71%) pure product. ^1H NMR (400 MHz, Chloroform-*d*) δ 6.95 – 6.91 (m, 2H), 6.90 – 6.85 (m, 2H), 4.54 (q, J = 8.5 Hz, 2H), 3.80 (s, 3H), 3.73 – 3.65 (m, 4H), 3.07 (s, 4H). ^{13}C NMR (101 MHz, $CDCl_3$) δ 154.50, 153.28, 145.33, 119.08, 114.54, 62.05, 61.69, 61.33, 60.97, 55.56, 50.86, 44.25. ^{19}F NMR (376 MHz, Chloroform-*d*) δ -74.23 (t, J = 8.5 Hz). MS (ESI), $[M+1]^+$ = 358.146, $C_{14}H_{18}F_3N$ requires 357.139.

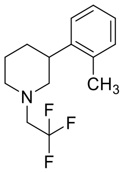

3-(o-tolyl)-1-(2,2,2-trifluoroethyl)piperidines (10)

3-(o-tolyl)piperidine hydrochloride (0.2 g, 0.9 mmol) was dissolved in 15 mL DMF, followed by the addition of potassium carbonate (0.4 g, 2.8 mmol) at room temperature. 2,2,2-trifluoroethyl 4-methylbenzenesulfonate (0.24 g, 0.9 mmol) was added to the reaction mixture. The reaction was allowed to stir under reflux conditions over night. Progress of the reaction was observed by TLC. On completion of the reaction, water (30 mL) was added to the reaction mixture, the organic phase was extracted with ethyl acetate (30 mL x 3) and dried over Na_2SO_4. The crude was purified by column chromatography using ethyl acetate in hexanes (15–20%) to obtained white solid (0.16 g, yield = 73%) pure product. ^1H NMR (400 MHz, Chloroform-*d*) δ 7.25 – 7.10 (m, 4H), 4.64 – 4.43 (m, 2H), 4.25 (d, J = 35.5 Hz, 2H), 3.10 – 2.98 (m, 1H), 2.95 – 2.73 (m, 2H), 2.39 (s, 3H), 2.02 – 1.83 (m, 2H), 1.79 – 1.59 (m, 2H). ^{13}C NMR (101 MHz, $CDCl_3$) δ 142.07, 135.97, 135.79, 130.70, 130.49, 126.63, 126.28, 126.19, 126.10, 125.64, 125.30, 61.63, 61.27, 54.52, 50.49, 38.52, 30.38, 25.99, 25.58, 19.45. ^{19}F NMR (376 MHz, $CDCl_3$) δ -68.86, -68.88, -68.91, -74.21, -74.23, -74.26. MS (ESI), $[M+1]^+$ = 258.146, $C_{14}H_{18}F_3N$ requires 257.139.

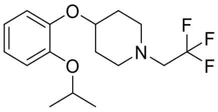

4-(2-isopropoxyphenoxy)-1-(2,2,2-trifluoroethyl)piperidine (11)

4-(2-isopropoxyphenoxy)piperidine (0.2 g, 0.84 mmol) was dissolved in 10 mL DMF, followed by the addition of potassium carbonate (0.35 g, 2.5 mmol) at room temperature. 2,2,2-trifluoroethyl 4-methylbenzenesulfonate (0.21 g, 0.84 mmol) was added to the reaction mixture. The reaction was allowed to stir under reflux conditions over night. Progress of the reaction was observed by TLC. On completion of the reaction, water (50 mL) was added to the reaction mixture, the organic phase was extracted with ethyl acetate (30 mL x 3) and dried over Na_2SO_4. The crude was purified by column chromatography using ethyl acetate in hexanes (20%) to obtained pale yellow solid (0.16 g, yield = 61%) pure product.

^1H NMR (400 MHz, Chloroform-*d*) δ 7.06 – 6.77 (m, 4H), 4.65 – 4.24 (m, 4H), 3.94 – 3.42 (m, 4H), 1.89 (s, 2H), 1.36 (dd, J = 6.1, 1.5 Hz, 6H), 1.30 (d, J = 10.3 Hz, 1H). ^{13}C NMR (101 MHz, $CDCl_3$) δ 153.37, 149.55, 147.97, 122.85, 121.52, 119.48, 117.47, 77.39, 77.07, 76.75, 73.82, 71.59, 61.95, 61.59, 61.23, 60.87, 41.11, 30.66, 30.32, 22.25. ^{19}F NMR (376 MHz, $CDCl_3$) δ -74.27, -74.29, -74.31. MS (ESI), $[M+1]^+$ = 318.167, $C_{15}H_{21}F_3NO_2$ requires 317.160.

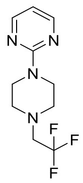

2-(4-(2,2,2-trifluoroethyl)piperazin-1-yl)pyrimidine (12)

2-(piperazin-1-yl)pyrimidine (0.61 g, 3.7 mmol) was dissolved in 20 mL DMF, followed by the addition of potassium carbonate (1.5 g, 11.1 mmol) at room temperature. 2,2,2-trifluoroethyl 4-methylbenzenesulfonate (0.94 g, 3.7 mmol) was added to the reaction mixture. The reaction was allowed to stir under reflux conditions over night. Progress of the reaction was observed by TLC. On completion of the reaction, water (50 mL) was added to the reaction mixture, the organic phase was extracted with ethyl acetate (30 mL x 3) and dried over Na_2SO_4. The crude was purified by column chromatography using ethyl acetate in hexanes (20–30%) to obtained white solid (0.59 g, yield = 65%) pure product. ^{1}H NMR (400 MHz, Chloroform-*d*) δ 8.35 (d, *J* = 4.7 Hz, 2H), 6.56 (t, *J* = 4.7 Hz, 1H), 4.55 (q, *J* = 8.5 Hz, 2H), 3.87 (s, 4H), 3.68 – 3.56 (m, 4H). ^{13}C NMR (101 MHz, $CDCl_3$) δ 161.53, 157.81, 153.45, 124.54, 121.78, 110.54, 62.08, 61.72, 61.36, 60.99, 43.94, 43.35. ^{19}F NMR (376 MHz, $CDCl_3$) δ -74.19, -74.21, -74.23. MS (ESI), $[M+1]^+$ = 358.146, $C_{14}H_{18}F_3N$ requires 357.139.

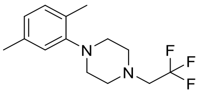

1-(2,5-dimethylphenyl)-4-(2,2,2-trifluoroethyl)piperazine (13)

^{1}H NMR (400 MHz, $CDCl_3$) δ 7.09 (d, *J* = 7.6 Hz, 1H), 6.92 – 6.82 (m, 2H), 3.08 (q, *J* = 9.6 Hz, 2H), 2.99 (t, *J* = 4.7 Hz, 4H), 2.88 (t, *J* = 4.6 Hz, 4H). ^{13}C NMR (101 MHz, $CDCl_3$) δ 136.23, 130.97, 129.31, 126.84, 124.06, 119.89, 77.35, 77.04, 76.72, 59.02, 58.72, 58.41, 58.11, 54.10, 51.74, 21.20, 17.48. ^{19}F NMR (376 MHz, $CDCl_3$) δ -68.85.

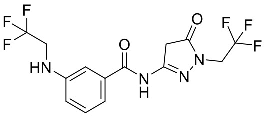

3-amino-N-(5-oxo-1-(2,2,2-trifluoroethyl)-4,5-dihydro-1H-pyrazol-3-yl)benzamide (14)

3-amino-N-(5-oxo-1-(2,2,2-trifluoroethyl)-4,5-dihydro-1H-pyrazol-3-yl)benzamide (0.2 g, 0.6 mmol) was dissolved in 5 mL DMF, followed by the addition of potassium carbonate (0.27 g, 1.9 mmol) at room temperature. 2,2,2-trifluoroethyl 4-methylbenzenesulfonate (0.17 g, 0.6 mmol) was added to the reaction mixture. The reaction was allowed to stir under reflux conditions for two hours. Progress of the reaction was observed by TLC. On completion of the reaction, water (20 mL) was added to the reaction mixture, the organic phase was extracted with ethyl acetate (30 mL x 3) and dried over Na_2SO_4. The crude was purified by column chromatography using ethyl acetate in hexanes (50%) to obtained cream yellow solid (0.15 g, yield = 69%) pure product. ^{1}H NMR (400 MHz, Chloroform-*d*) δ 8.41 (s, 1H), 7.25 (d, *J* = 7.8 Hz, 1H), 7.22 – 7.15 (m, 2H), 6.87 (ddd, *J* = 7.9, 2.3, 0.9 Hz, 1H), 6.46 (s, 1H), 4.51 (p, *J* = 8.1 Hz, 4H), 3.87 (s, 2H). ^{13}C NMR (101 MHz, $CDCl_3$) δ 165.09, 152.97, 147.03, 146.96, 134.73, 130.16, 129.76, 128.11, 118.68, 116.50, 113.73, 79.79, 77.35, 77.03, 76.71, 68.84, 68.47, 47.40. ^{19}F NMR (376 MHz, $CDCl_3$) δ -71.23, -71.26, -71.28, -73.81, -73.83, -73.86, -74.29, -74.31, -74.33. MS (ESI), $[M+Na]^+$ = 405.075, $C_{14}H_{12}F_6N_4O_2$ requires 382.086.

4. Supplementary references

1. Rotstein, B. H. *et al.* Synthesis of [11 C]Bexarotene by Cu-Mediated [11 C]Carbon Dioxide Fixation and Preliminary PET Imaging. *ACS Med. Chem. Lett.* **5**, 668–672 (2014).

2. Chuit, C., Corriu, R., Perz, R. & Reyé, C. Improved Procedure for the Selective Reduction of Carbonyl Compounds and Carboxylic Acid Esters by Potassium Salt-Induced Hydrosilylation. *Synthesis (Stuttg).* **1982**, 981–984 (2002).

3. Vela, J. *et al.* Synthesis and Reactivity of Low-Coordinate Iron(II) Fluoride Complexes and Their Use in the Catalytic Hydrodefluorination of Fluorocarbons. *J. Am. Chem. Soc.* **127**, 7857–7870 (2005).

4. Rosenthal, M. S., Bosch, A. L., Nickles, R. J. & Gatley, S. J. Synthesis and some characteristics of no-carrier added [18F]fluorotrimethylsilane. *Int. J. Appl. Radiat. Isot.* **36**, 318–319 (1985).

5. Ting, R., Adam, M. J., Ruth, T. J. & Perrin, D. M. Arylfluoroborates and Alkylfluorosilicates as Potential PET Imaging Agents: High-Yielding Aqueous Biomolecular 18 F-Labeling. *J. Am. Chem. Soc.* **127**, 13094–13095 (2005).

6. Al-huniti, M. H., Lu, S., Pike, V. W. & Lepore, S. D. Enhanced nucleophilic fluorination and radiofluorination of organosilanes appended with potassium-chelating leaving groups. *J. Fluor. Chem.* **158**, 48–52 (2014).

7. Mita, T., Michigami, K. & Sato, Y. Iridium- and Rhodium-Catalyzed Dehydrogenative Silylations of C(sp 3)⍰H Bonds Adjacent to a Nitrogen Atom Using Hydrosilanes. *Chem. - An Asian J.* **8**, 2970–2973 (2013).

8. Prakash, G. K. S. & Yudin, A. K. Perfluoroalkylation with Organosilicon Reagents. *Chem. Rev.* **97**, 757–786 (1997).

9. Tyrra, W. *et al.* Fluoride-mediated selective cross-coupling reactions of alkyl halides and trimethyl(perfluoroalkyl)silanes, Me3SiRf (Rf=CF3, C2F5) in the absence of any catalysts. *J. Fluor. Chem.* **128**, 813–817 (2007).

10. Alonso, C., Martínez de Marigorta, E., Rubiales, G. & Palacios, F. Carbon Trifluoromethylation Reactions of Hydrocarbon Derivatives and Heteroarenes. *Chem. Rev.* **115**, 1847–1935 (2015).

11. Miyake, Y., Ota, S. & Nishibayashi, Y. Copper-Catalyzed Nucleophilic Trifluoromethylation of Allylic Halides: A Simple Approach to Allylic Trifluoromethylation. *Chem. – A Eur. J.* **18**, 13255–13258 (2012).

12. Finney, W. F., Wilson, E., Callender, A., Morris, M. D. & Beck, L. W. Reexamination of Hexafluorosilicate Hydrolysis by 19 F NMR and pH Measurement. *Environ. Sci. Technol.* **40**, 2572–2577 (2006).

13. Breliere, C. *et al.* Preparation and NMR studies of hexacoordinated fluorosilicates: nondissociative fluorine site exchange within the octahedral complexes in solution. *Organometallics* **11**, 1586–1593 (1992).

14. Lataste, E. *et al.* Highly Fluorinated Silica Obtained by Direct F 2 -Gas Fluorination: Stability and Unprecedented Fluorosilicate Species Revealed by Solid State NMR Investigations. *J. Phys. Chem. C* **113**, 18652–18660 (2009).

15. Sadoc, A. *et al.* NMR parameters in alkali, alkaline earth and rare earth fluorides from first principle calculations. *Phys. Chem. Chem. Phys.* **13**, 18539 (2011).
