# Supplementary material for: A protocol for controlled reactivity shift in the 2,2-difluorovinyl motif used for selective S–18F and C–18F bond formation
Source: Commun Chem. 2024 Apr 29;7:97. doi: 10.1038/s42004-024-01132-3 (PMC11058245; doi:10.1038/s42004-024-01132-3)
Supplement: Supplementary file 2 — Description of Additional Supplementary Files [file 42004_2024_1132_MOESM2_ESM.pdf]

Description of Additional Supplementary Files

File name: Supplementary Data 1

Description: NMR spectra

File name: Supplementary Data 2

Description: Radio chromatograms
