# Supplementary material for: A protocol for controlled reactivity shift in the 2,2-difluorovinyl motif used for selective S–18F and C–18F bond formation
Source: Commun Chem. 2024 Apr 29;7:97. doi: 10.1038/s42004-024-01132-3 (PMC11058245; doi:10.1038/s42004-024-01132-3)
Supplement: Supplementary file 3 — Supplementary data 1 [file 42004_2024_1132_MOESM3_ESM.pdf]

Supplementary Data 1

Controlled reactivity shift in the 2,2-difluorovinyl motif: A protocol for selective S–^{18}F and C–^{18}F bond formation

Mudasir Maqbool[1,2], Jimmy Erik Jakobsson[1], Santosh Reddy Alluri[1], Vasko Kramer[3] and Patrick Johannes Riss[*1,2,4]
DOI: 10.xxxxxxxx

[1]*Department of Clinical Neurocience, OUS-Ullevål, Oslo, Norway.*
[2]*Department of Chemistry, University of Oslo, Oslo, Norway. Tel: +4795028669; E-mail: patrick.riss@kjemi.uio.no*
[3]*Positronpharma SA, Rancangua, Santiago de Chile, Chile*
[4]*Department of Chemistry, Johannes Gutenberg-University, Fritz-Strassmann-Weg 2, 55128 Mainz, Germany. Tel. +49 6131 39 28081; Email: priss@uni-mainz.de*

NMR Spectra

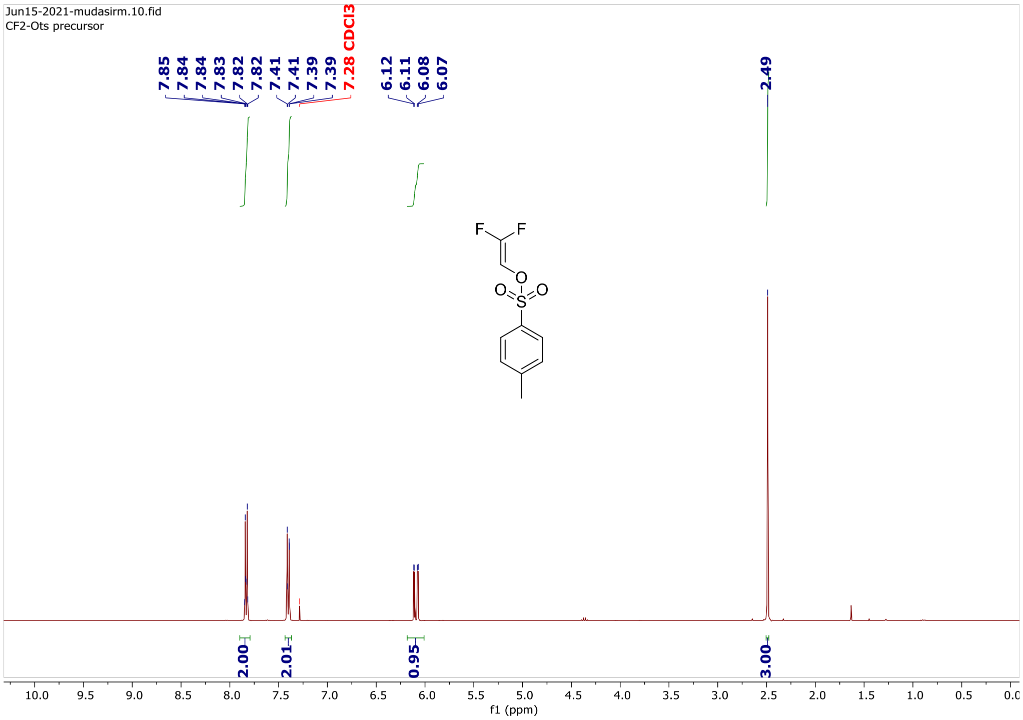

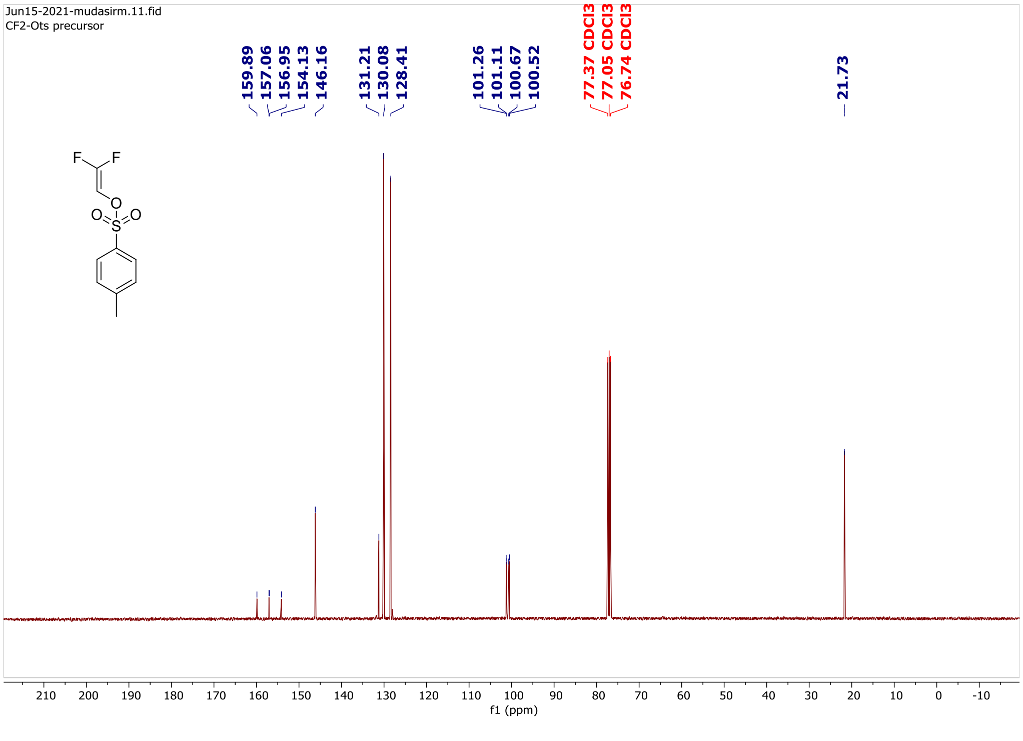

Figure S3: Top: 400 MHz ^{1}H-NMR of 2,2-difluorovinyl 4-methylbenzenesulfonate **(1a)**. Bottom: 100 MHz ^{13}C-NMR of 2,2-difluorovinyl 4-methylbenzenesulfonate **(1a)**.

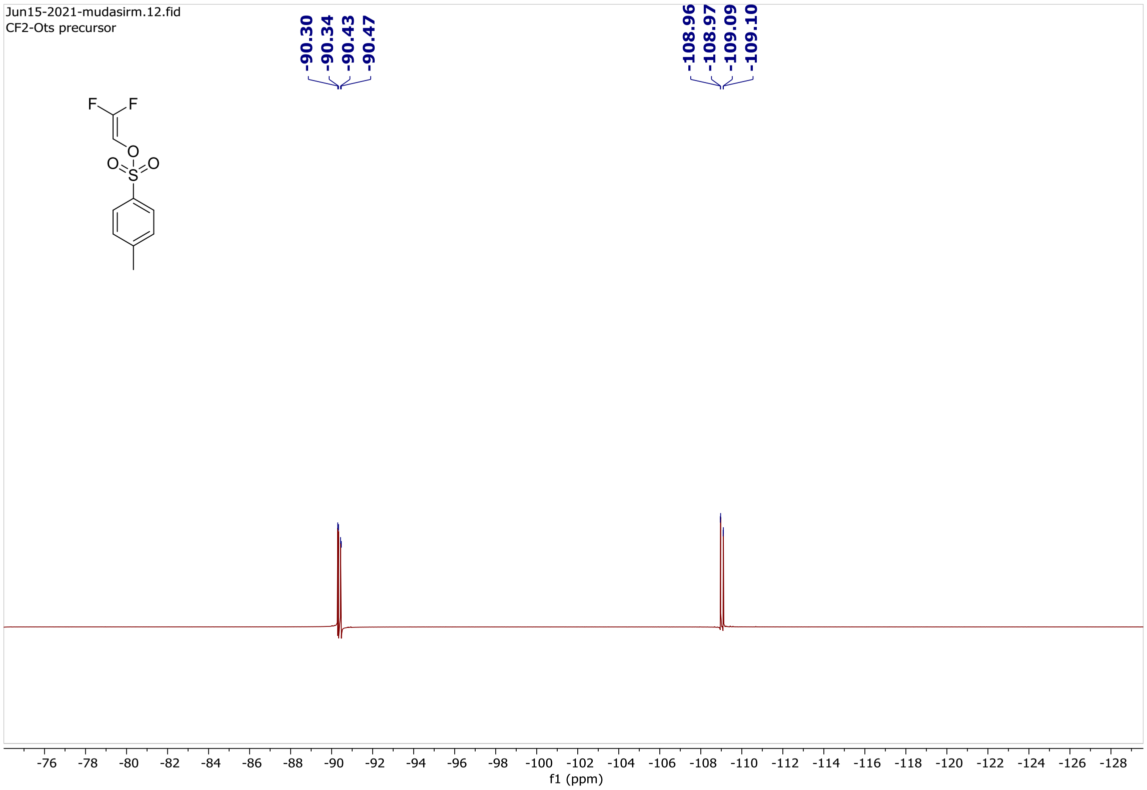

Figure S4: 376 MHz ^{19}F-NMR of 2,2-difluorovinyl 4-methylbenzenesulfonate **(1a)**.

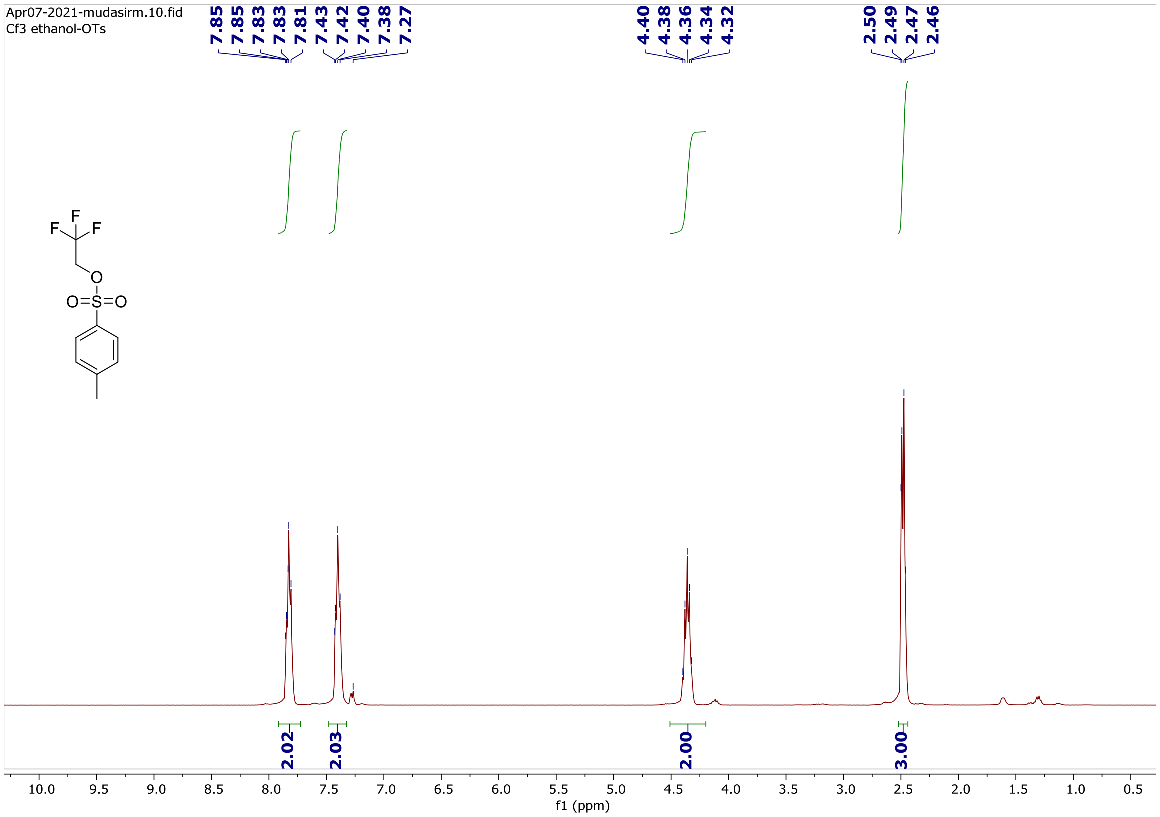

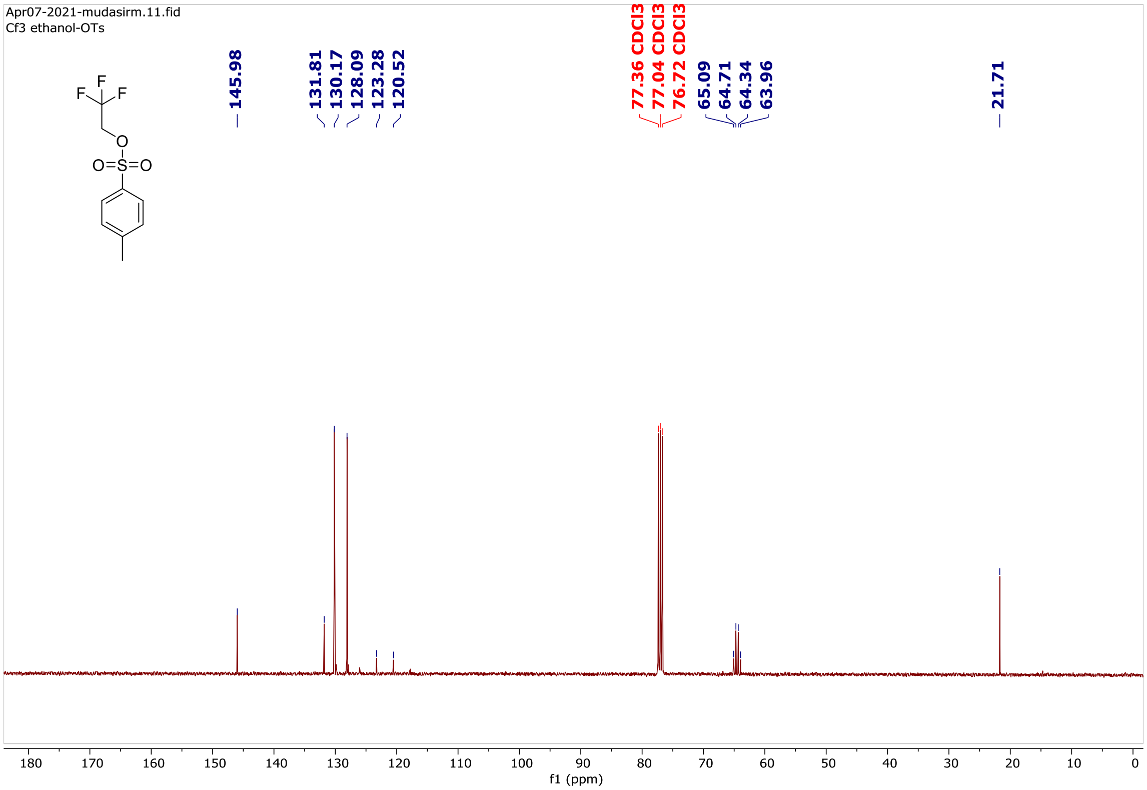

Figure S5: Top: 400 MHz ^{1}H-NMR of 2,2,2-trifluoroethyl 4-methylbenzenesulfonate **(1b)**. Bottom: 100 MHz ^{13}C-NMR of 2,2,2-trifluoroethyl 4-methylbenzenesulfonate **(1b)**.

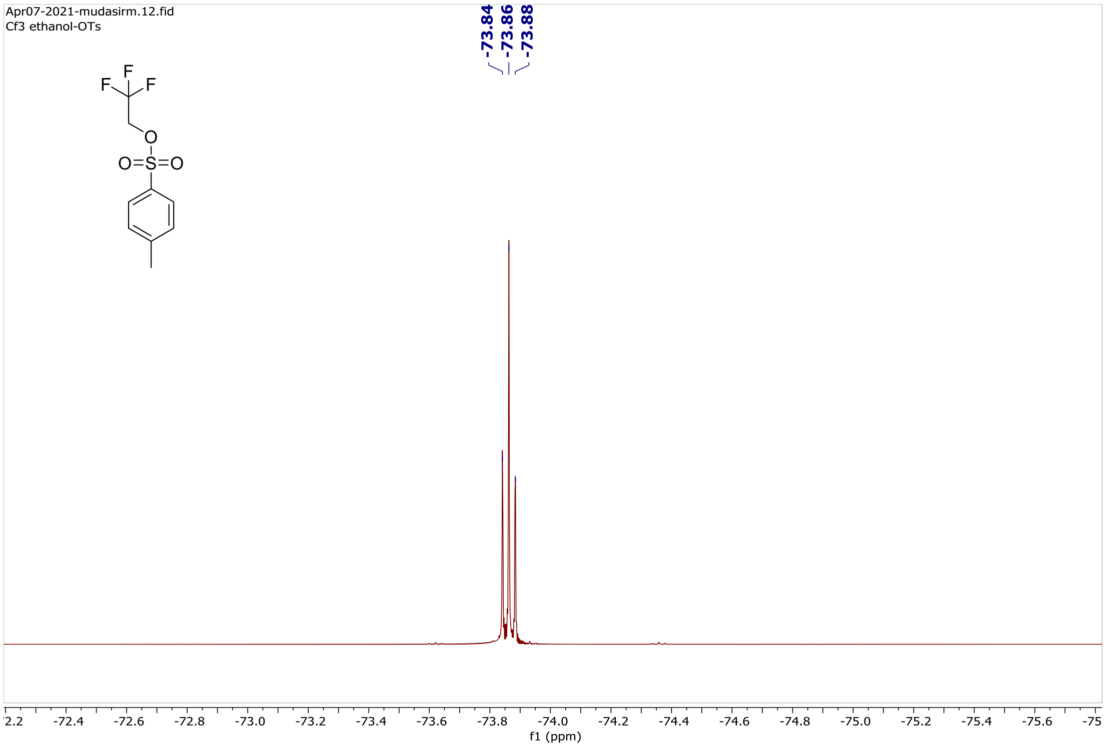

Figure S6: 376 MHz [19] F-NMR of 2,2,2-trifluoroethyl 4-methylbenzenesulfonate **(1b)**.

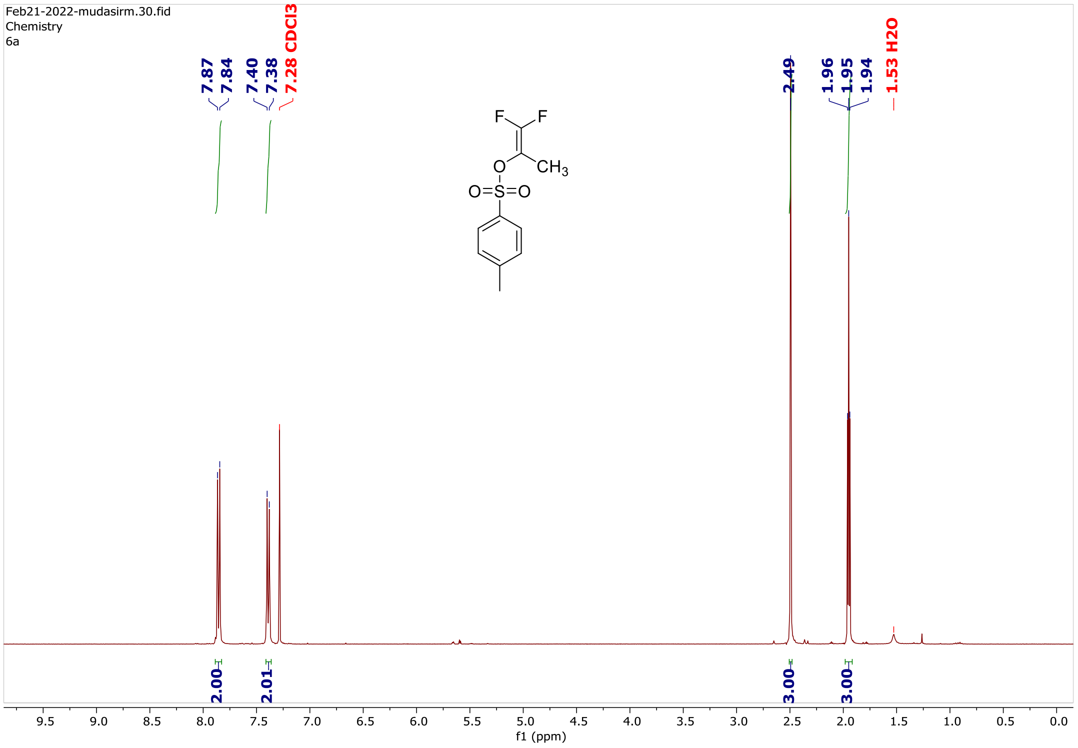

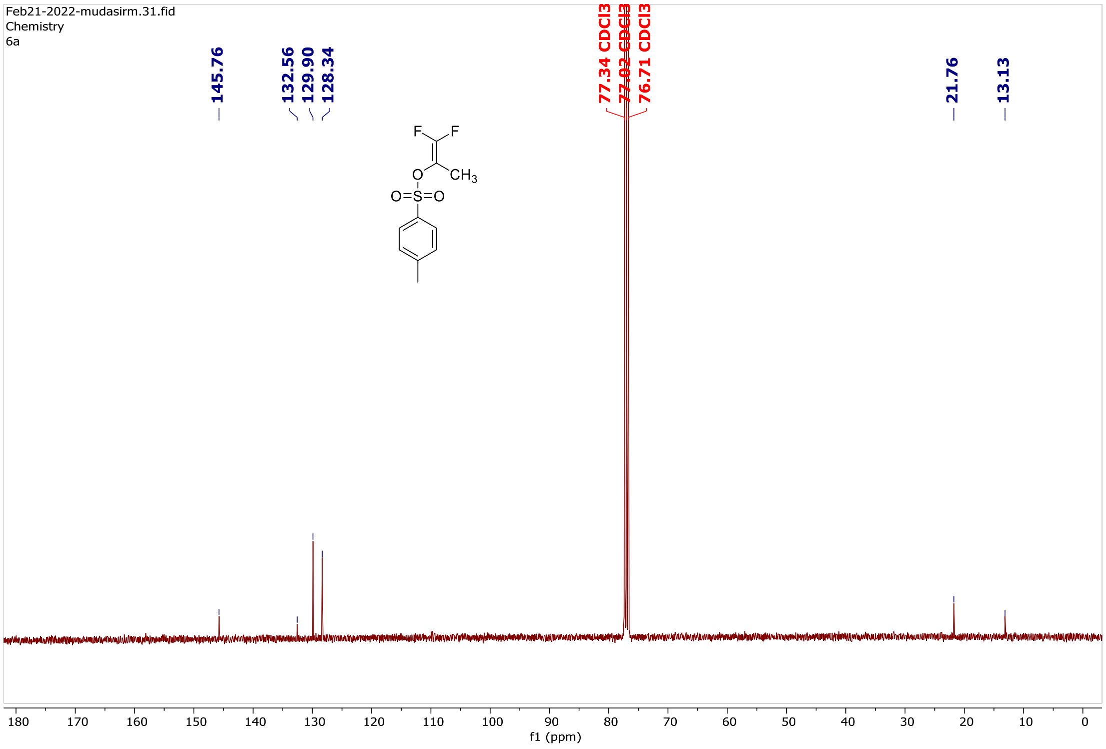

Figure S7: Top: 400 MHz ^{1}H-NMR of 1,1-difluoroprop-1-en-2-yl 4-methylbenzenesulfonate **(2a)**. Bottom: 100 MHz ^{13}C-NMR of 1,1-difluoroprop-1-en-2-yl 4-methylbenzenesulfonate **(2a)**.

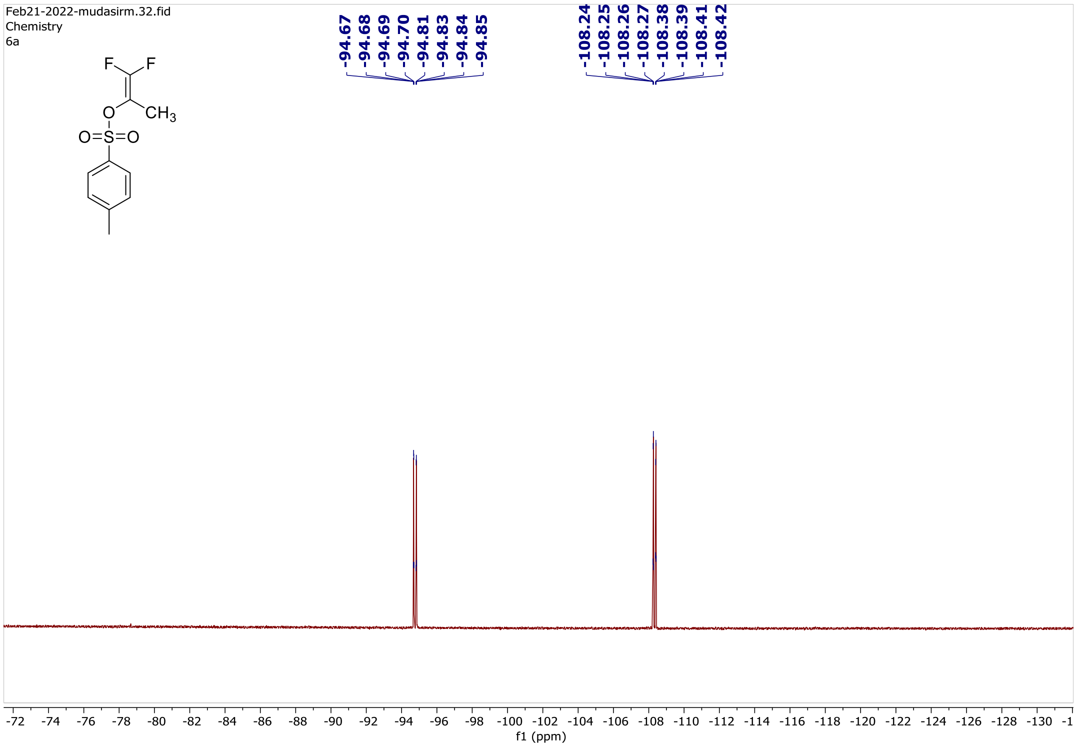

Figure S8: 376 MHz ^{19} F-NMR of 1,1-difluoroprop-1-en-2-yl 4-methylbenzenesulfonate **(2a)**.

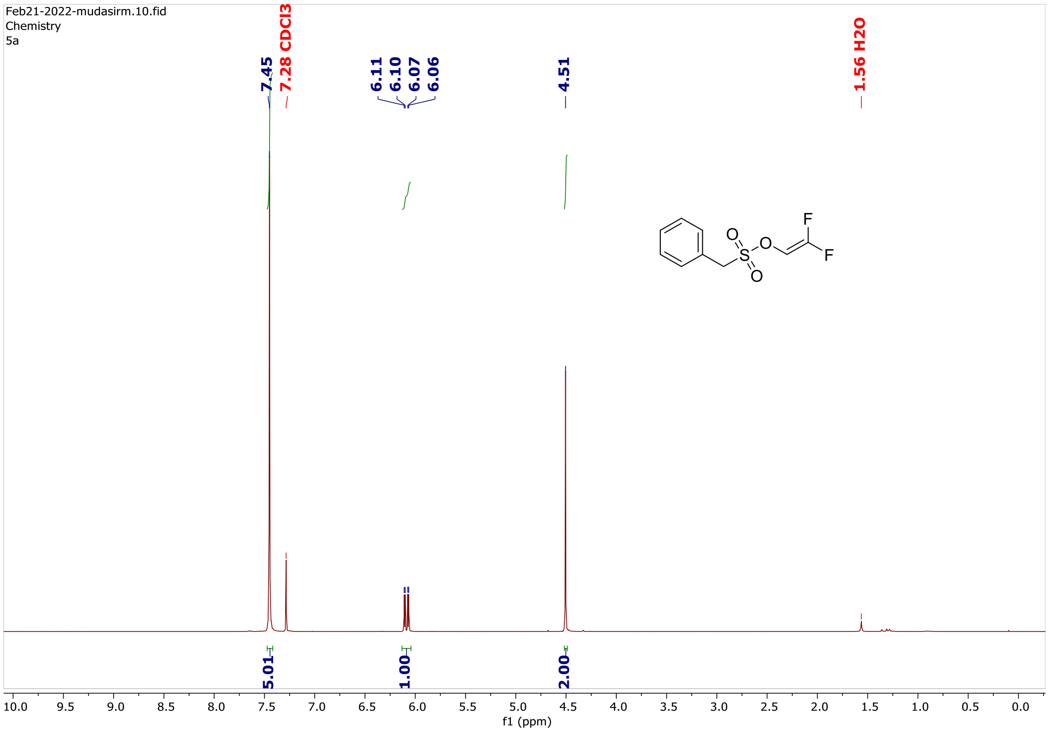

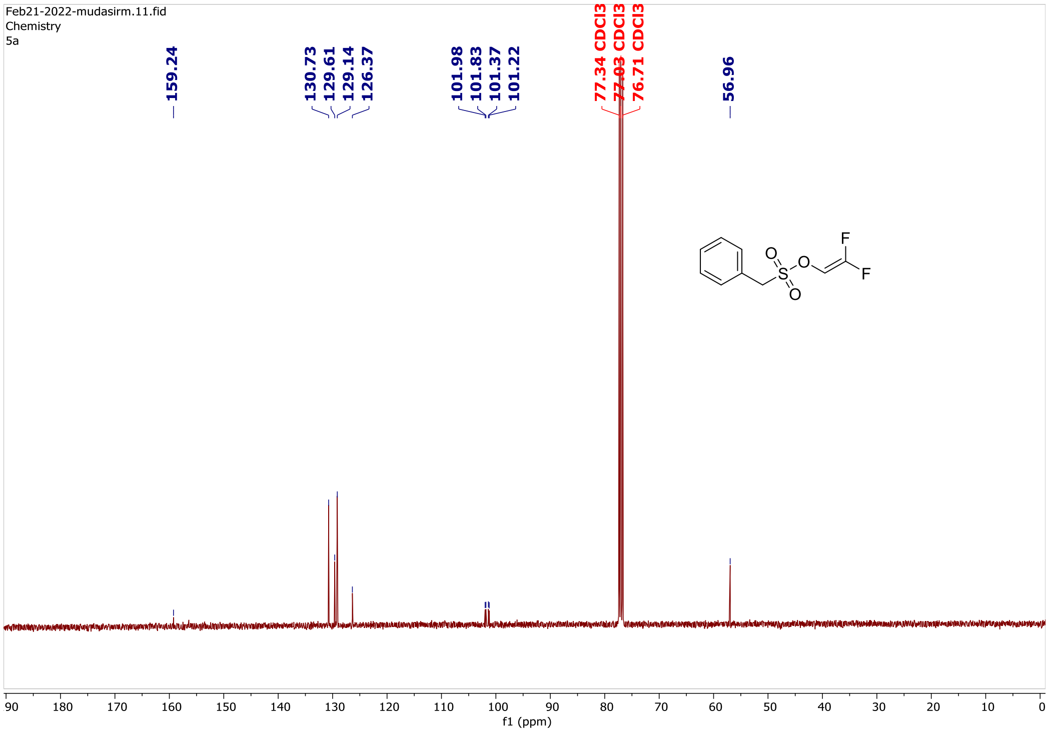

Figure S9: Top: 400 MHz ^{1}H-NMR of 2,2-difluorovinyl phenylmethanesulfonate **(3a)**. Bottom: 100 MHz ^{13}C-NMR of 2,2-difluorovinyl phenylmethanesulfonate **(3a)**.

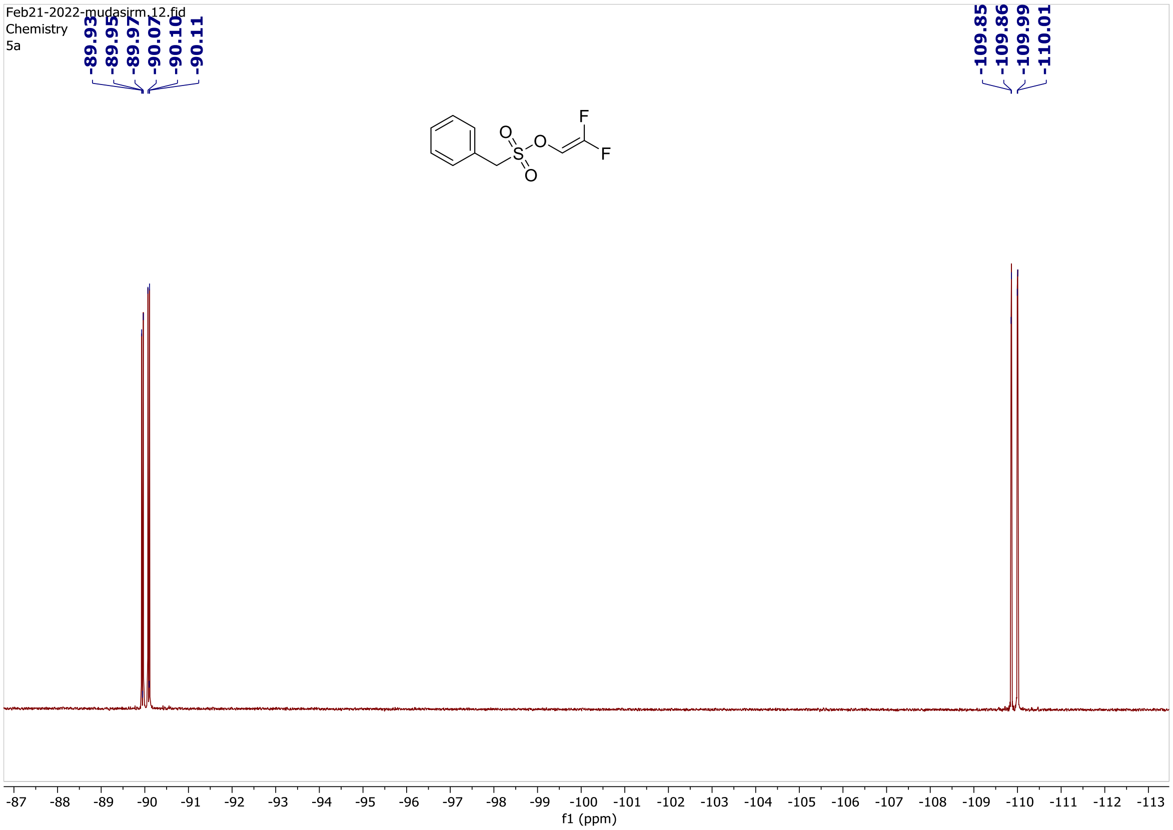

Figure S10: 376 MHz [19] F-NMR of 2,2-difluorovinyl phenylmethanesulfonate **(3a)**.

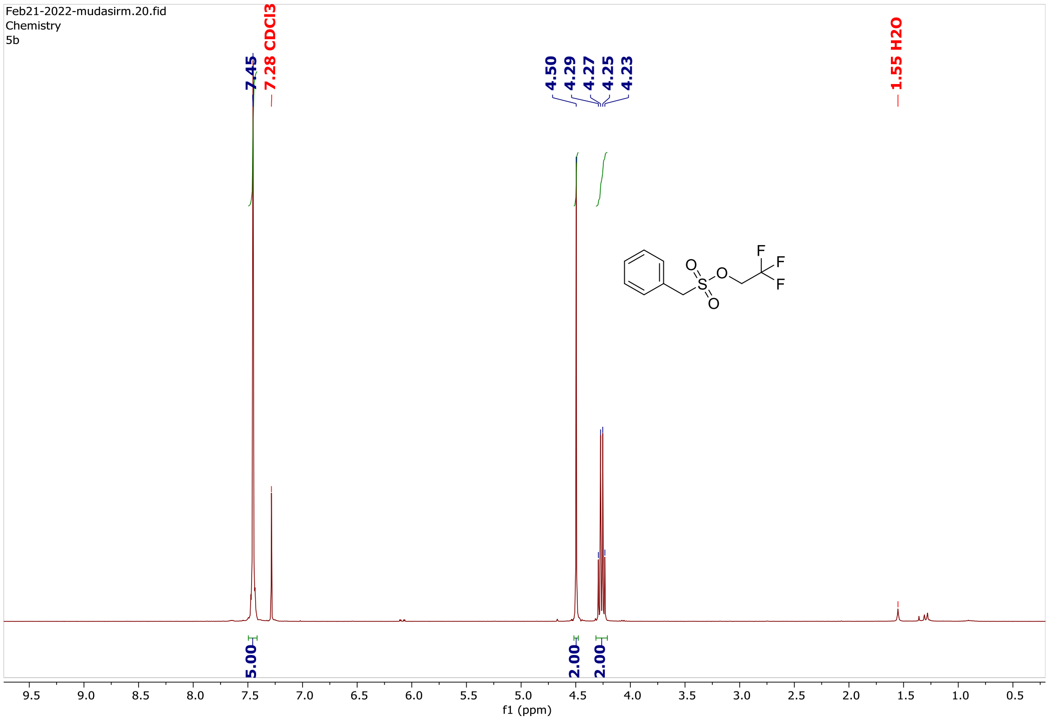

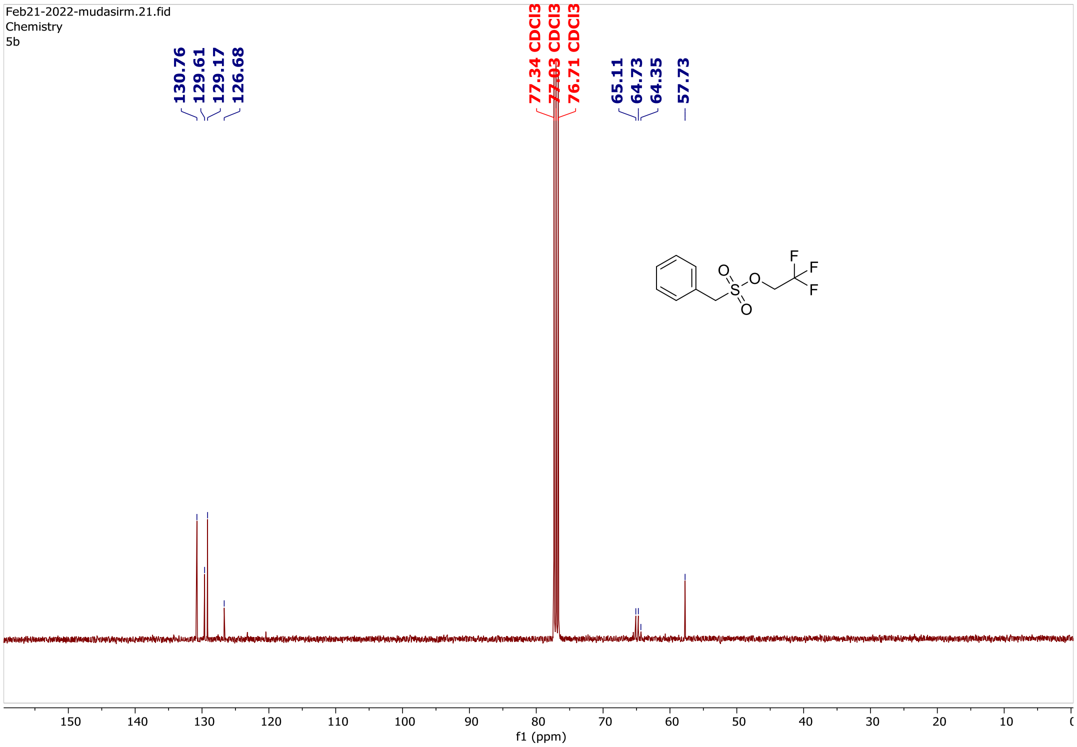

Figure S11: Top: 400 MHz ^{1}H-NMR of 2,2,2-trifluoroethyl phenylmethanesulfonate **(3b)**. Bottom: 100 MHz ^{13}C-NMR of 2,2,2-trifluoroethyl phenylmethanesulfonate **(3b)**.

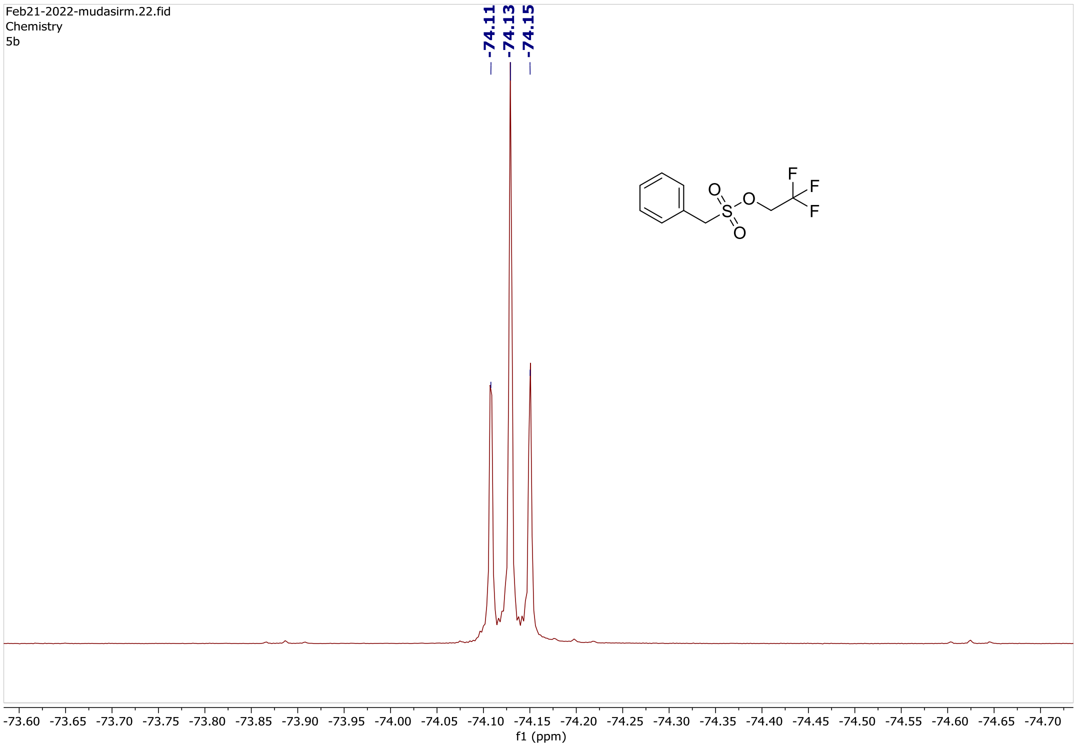

Figure S12: 376 MHz ^{19}F-NMR of 2,2,2-trifluoroethyl phenylmethanesulfonate **(3b)**.

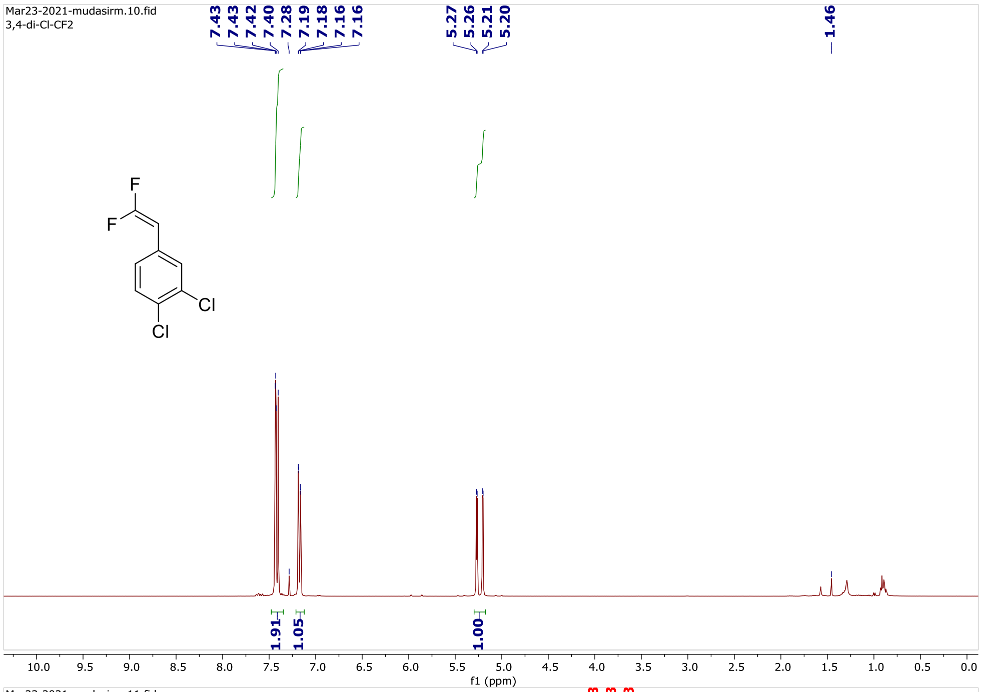

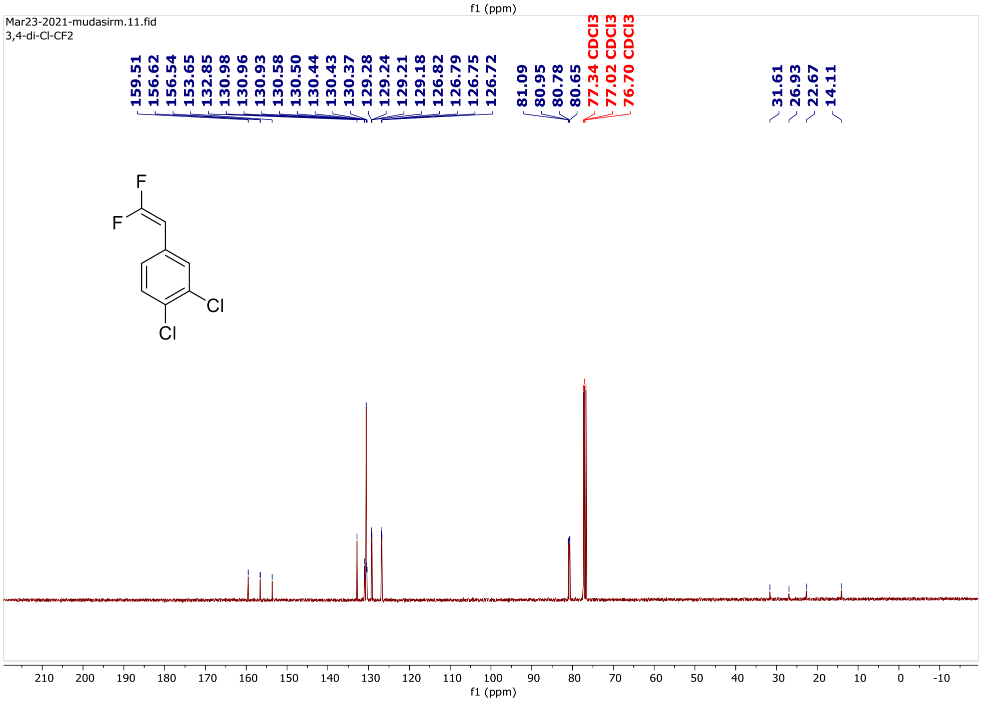

Figure S13: Top: 400 MHz ^{1}H-NMR of 1,2-dichloro-4-(2,2-difluorovinyl)benzene. Bottom: 100 MHz ^{13}C-NMR of 1,2-dichloro-4-(2,2-difluorovinyl)benzene **(4a)**.

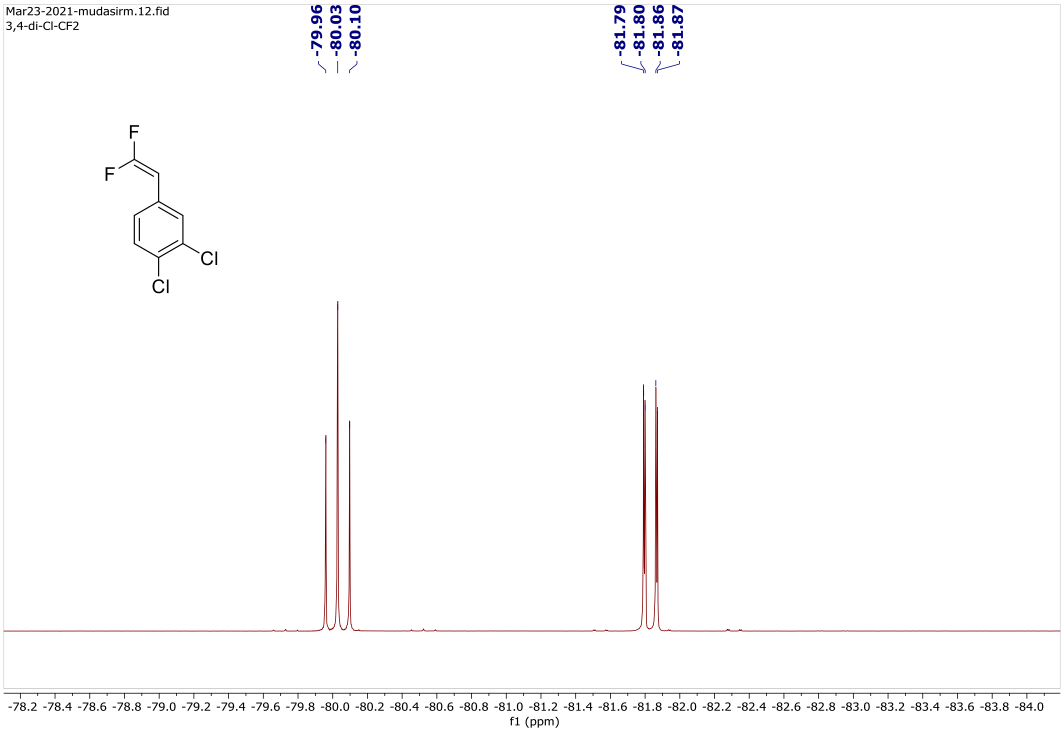

Figure S14: 376 MHz ^{19} F-NMR of 1,2-dichloro-4-(2,2-difluorovinyl)benzene **(4a)**.

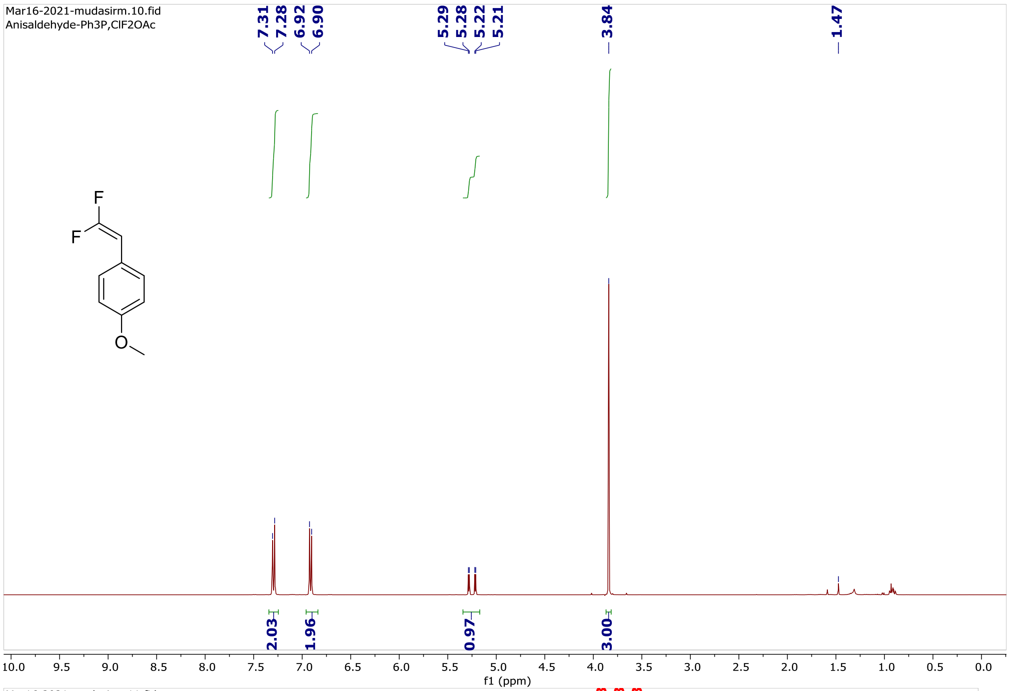

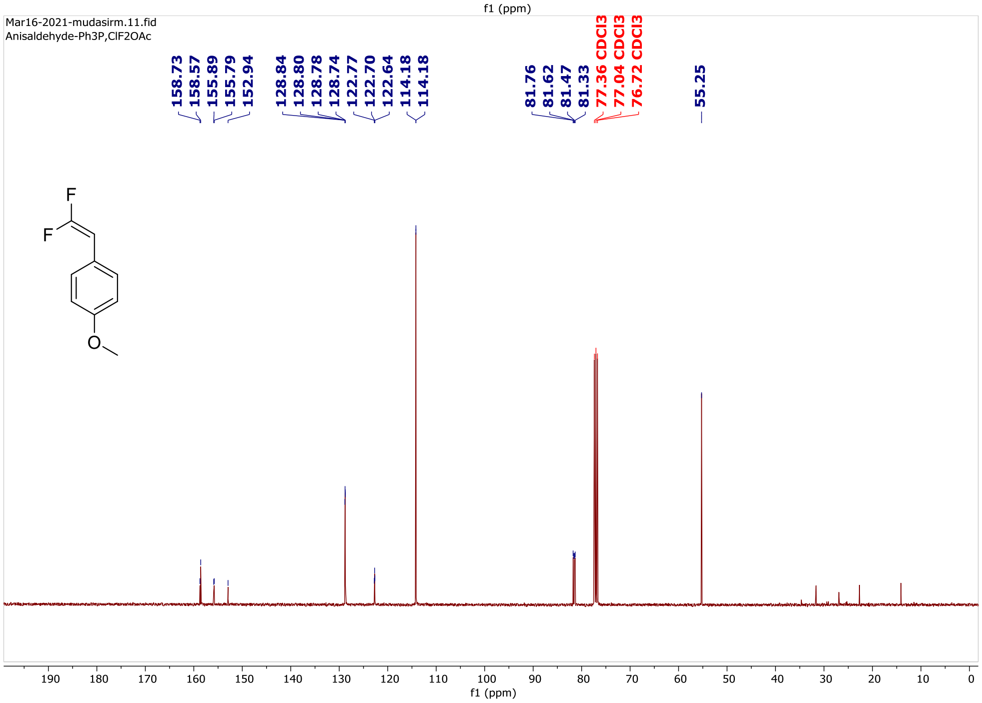

Figure S15: *Top: 400 MHz ^{1}H-NMR of 1-(2,2-difluorovinyl)-4-methoxybenzene* ***(5a)***. *Bottom: 100 MHz ^{13}C-NMR of 1-(2,2-difluorovinyl)-4-methoxybenzene* ***(5a)***.

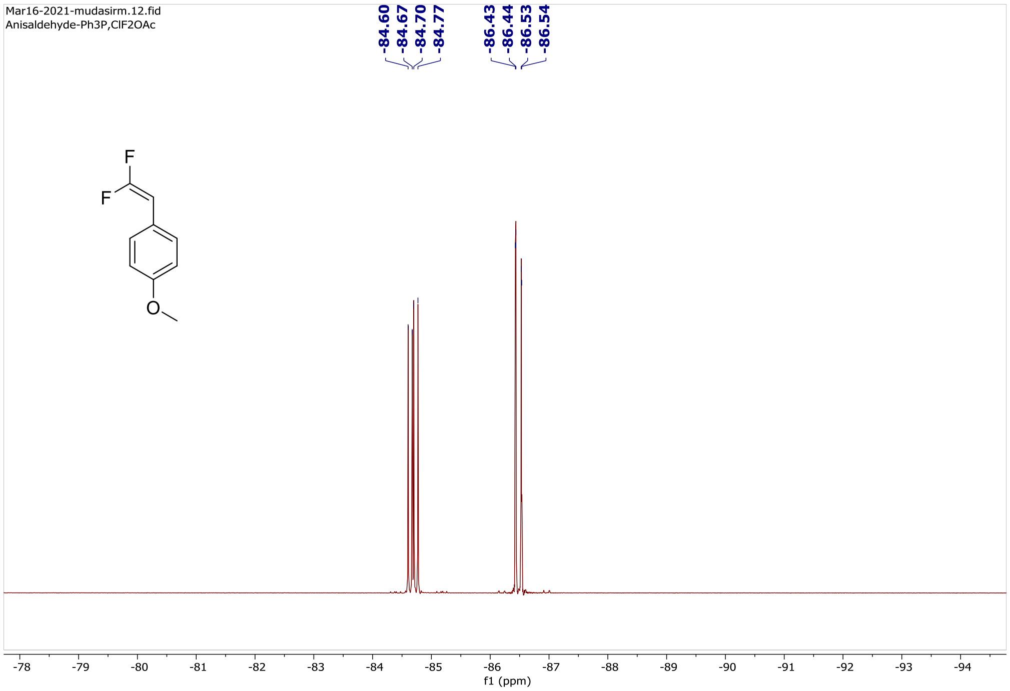

*Figure S16: 376 MHz [19] F-NMR of 1-(2,2-difluorovinyl)-4-methoxybenzene **(5a)**.*

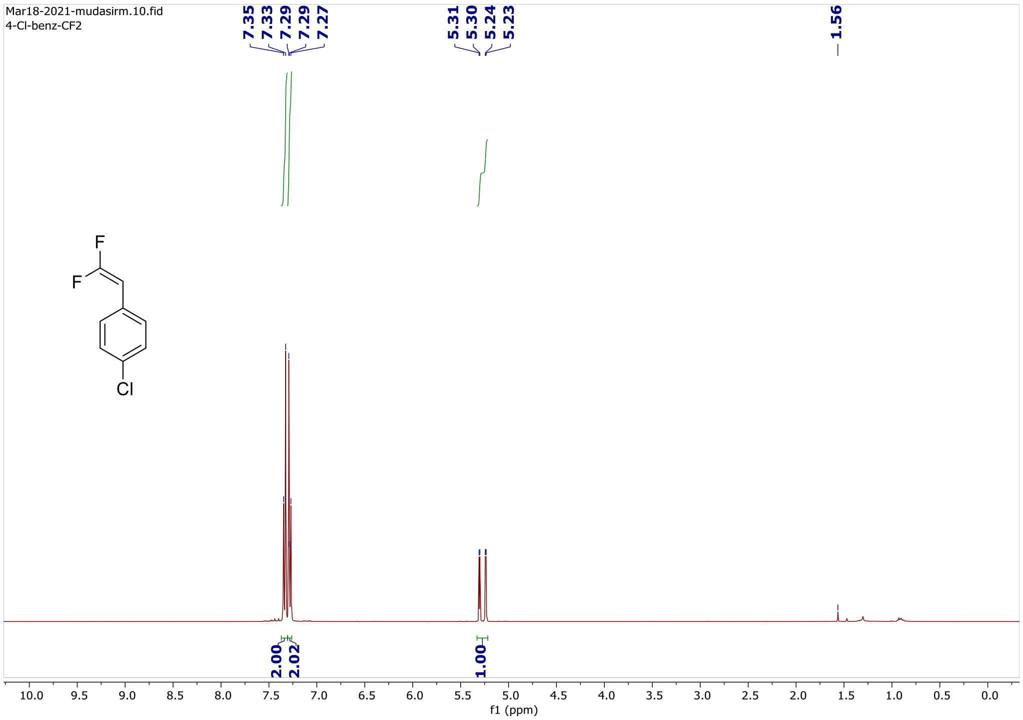

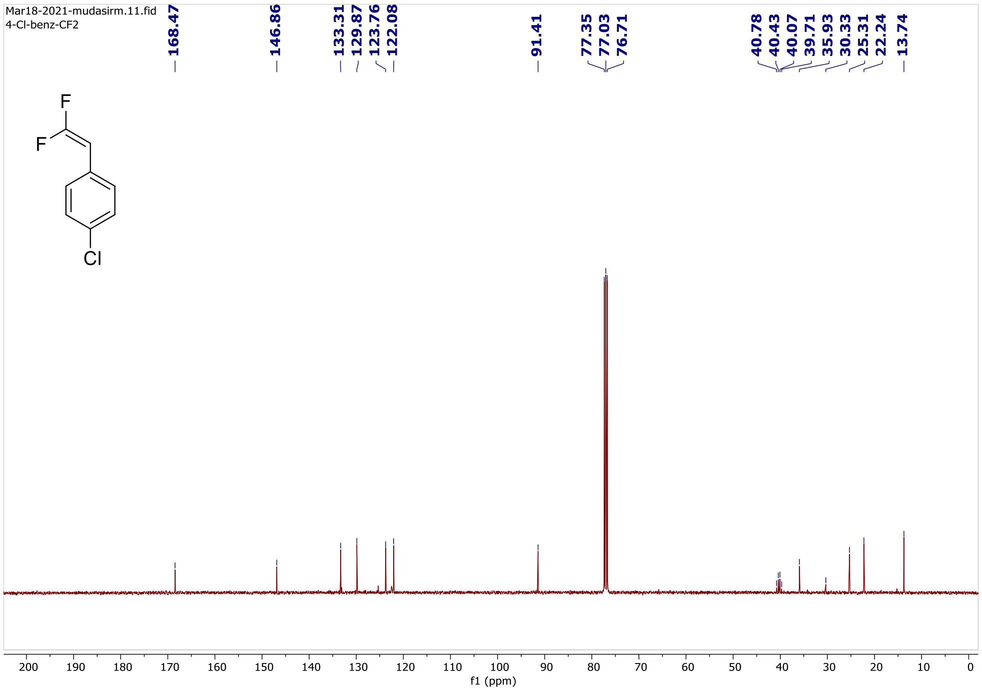

Figure S17: *Top: 400 MHz ^{1}H-NMR of 1-chloro-4-(2,2-difluorovinyl)benzene* ***(6a)****. Bottom: 100 MHz ^{13}C-NMR of 1-chloro-4-(2,2-difluorovinyl)benzene* ***(6a)****.*

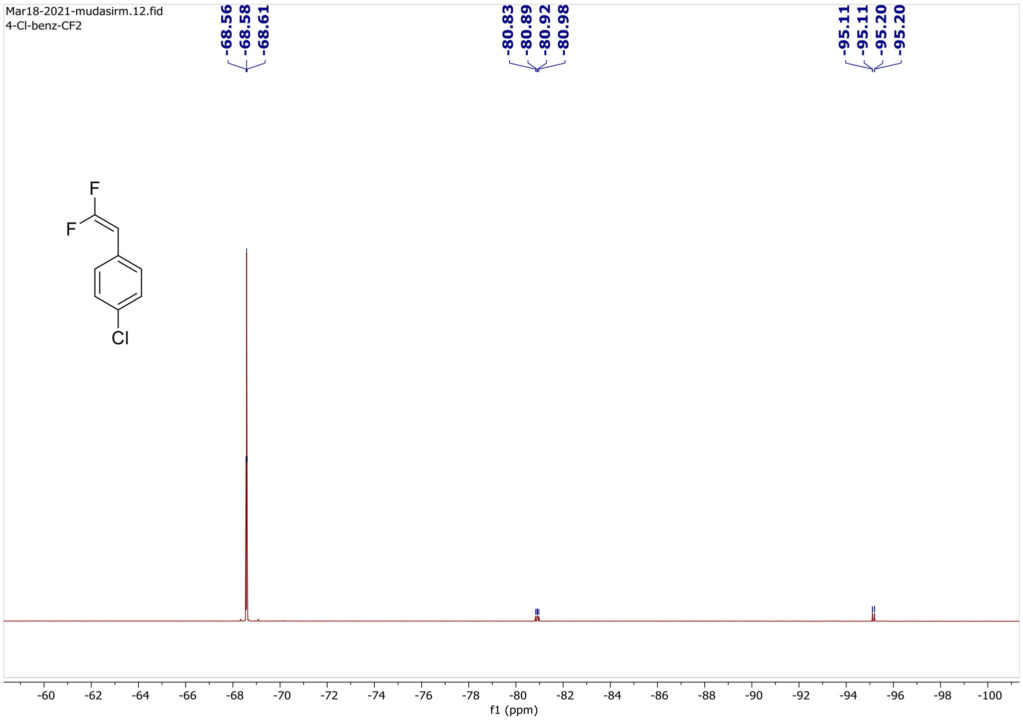

Figure S18: 376 MHz [19] F-NMR of 1-chloro-4-(2,2-difluorovinyl)benzene **(6a)**.

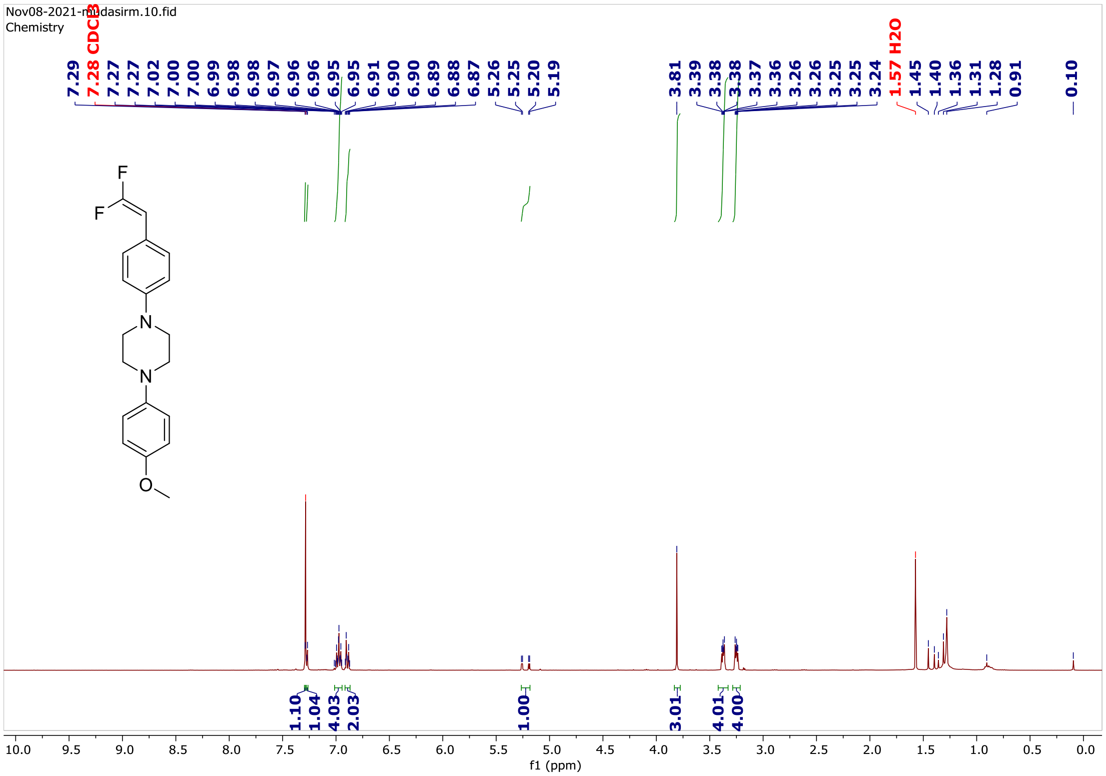

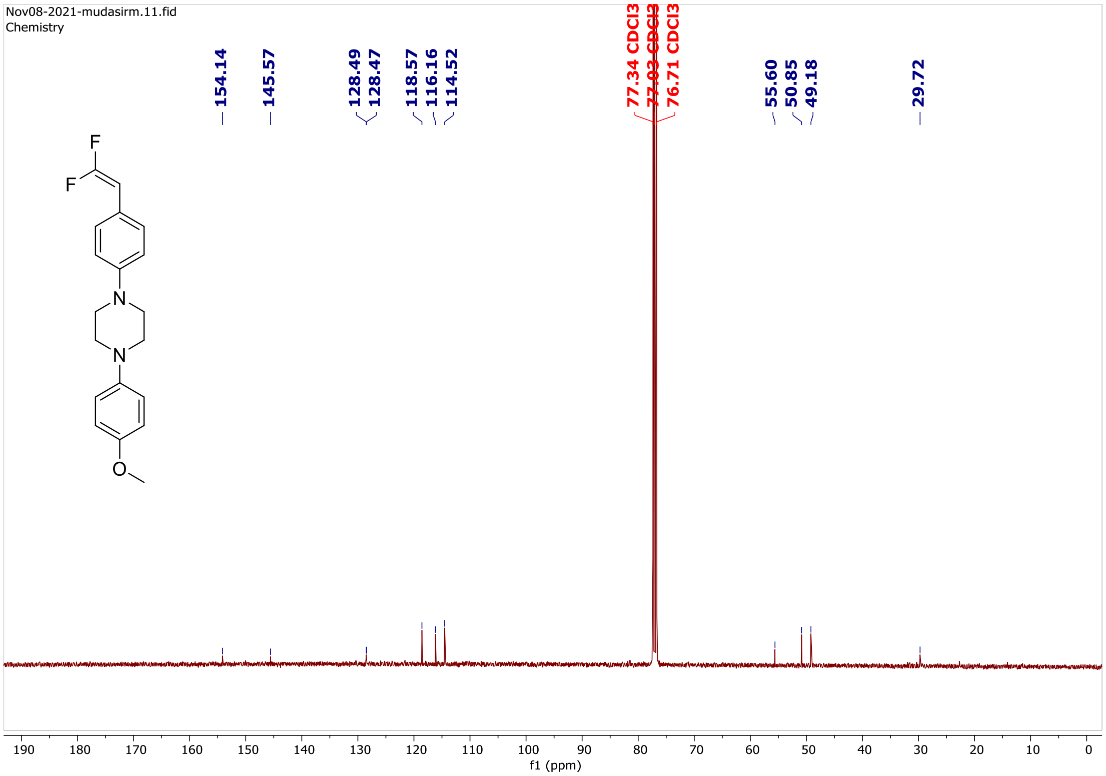

Figure S19: Top: 400 MHz ^{1}H-NMR of 1-(4-(2,2-difluorovinyl)phenyl)-4-(4-methoxyphenyl)piperazine **(7a)**. Bottom: 100 MHz ^{13}C-NMR of 1-(4-(2,2-difluorovinyl)phenyl)-4-(4-methoxyphenyl)piperazine **(7a)**.

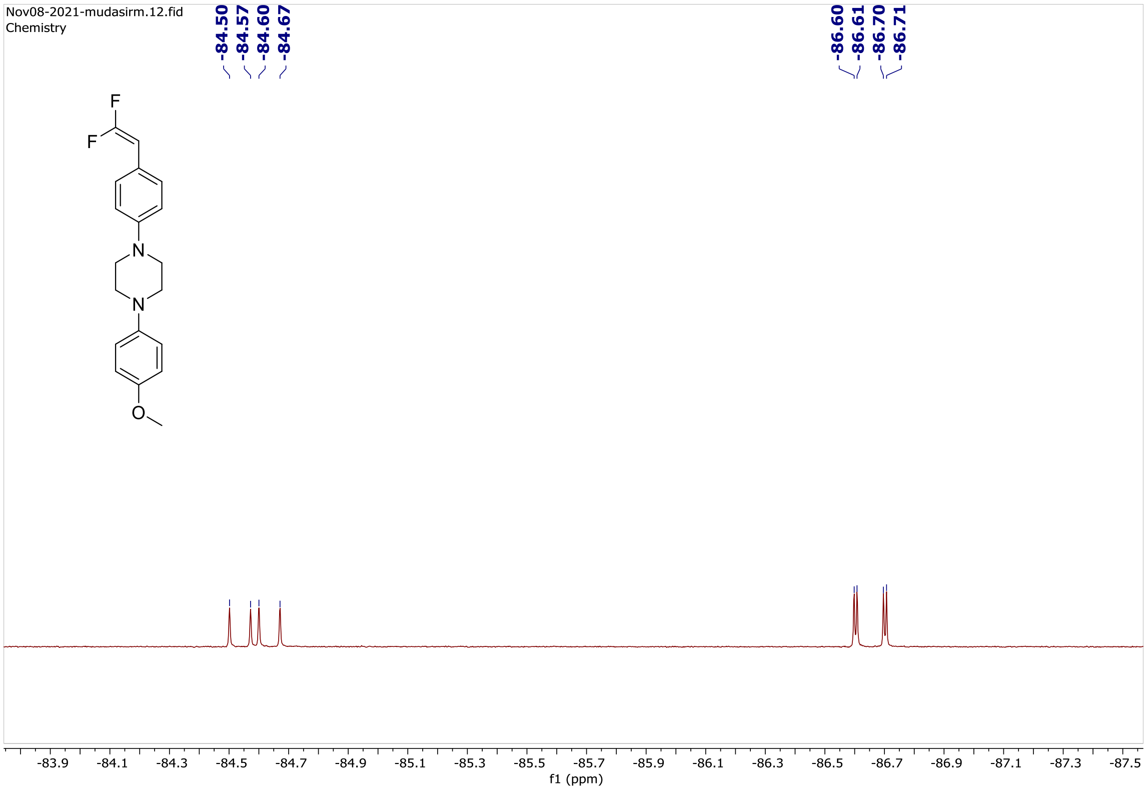

Figure S20: 376 MHz 19 F-NMR of 1-(4-(2,2-difluorovinyl)phenyl)-4-(4-methoxyphenyl)piperazine **(7a)**.

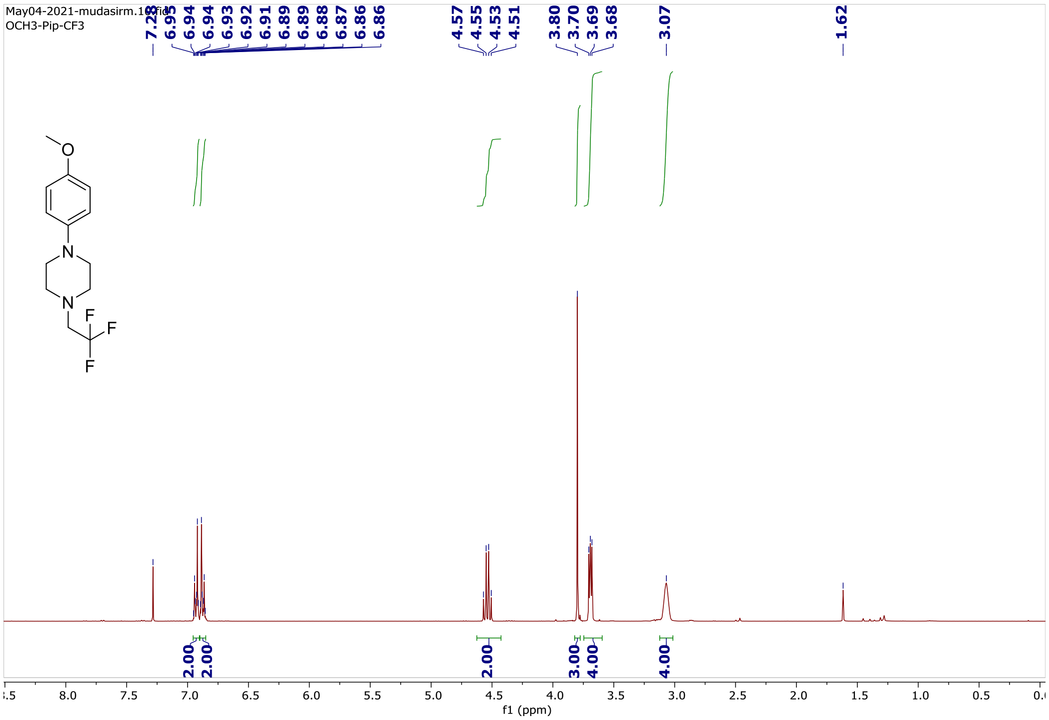

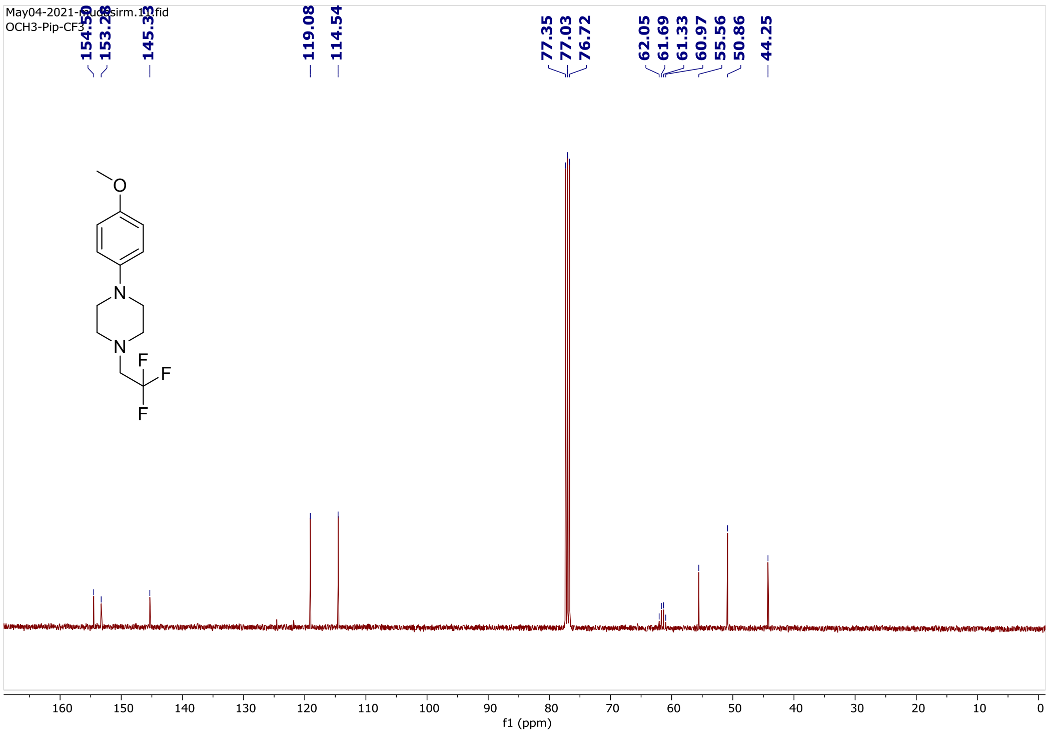

Figure S21: *Top: 400 MHz ^{1}H-NMR of 1-(4-methoxyphenyl)-4-(2,2,2-trifluoroethyl)piperazine. Bottom: 100 MHz ^{13}C-NMR of 1-(4-methoxyphenyl)-4-(2,2,2-trifluoroethyl)piperazine* ***(7b)****.*

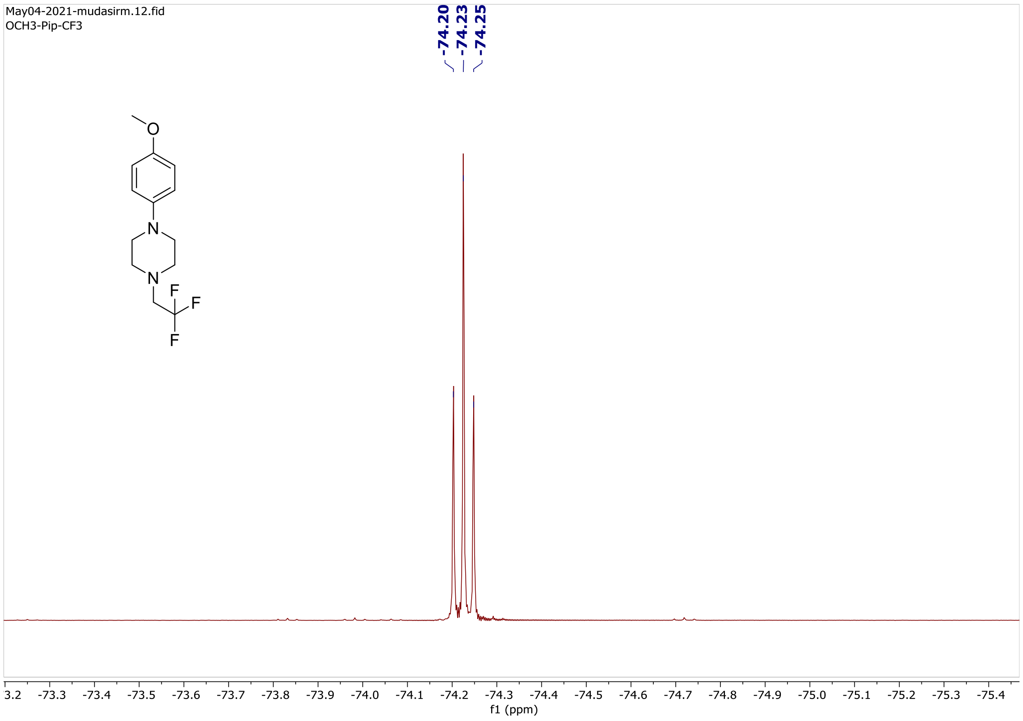

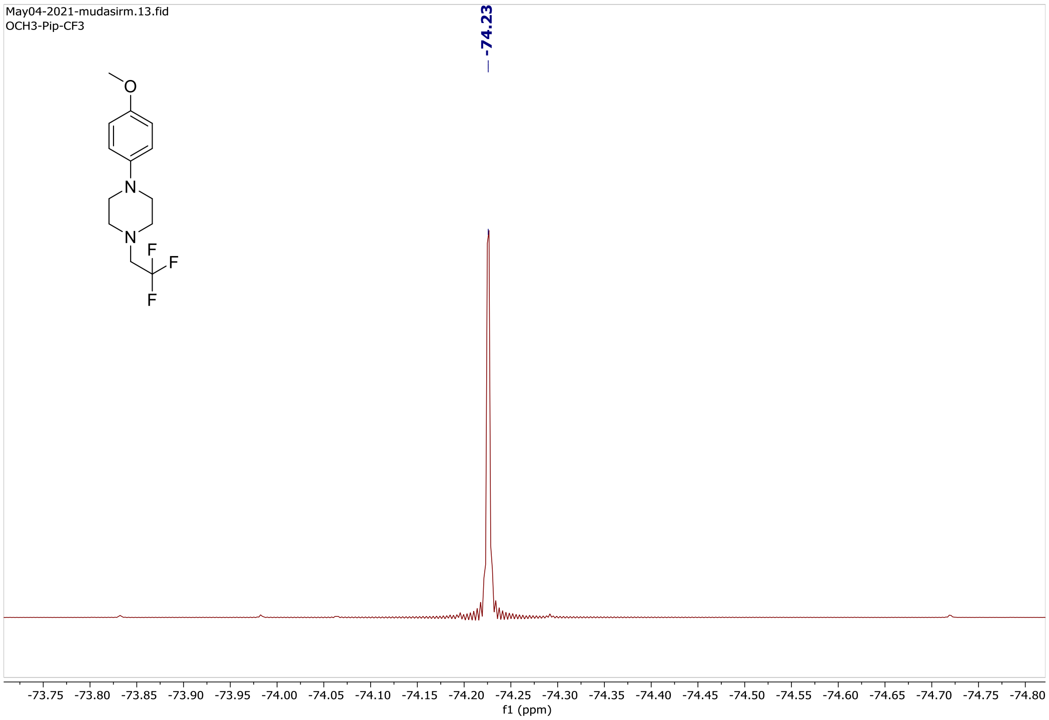

Figure S22: 376 MHz ^{19}F-NMR of 1-(4-methoxyphenyl)-4-(2,2,2-trifluoroethyl)piperazine **(7b)**.

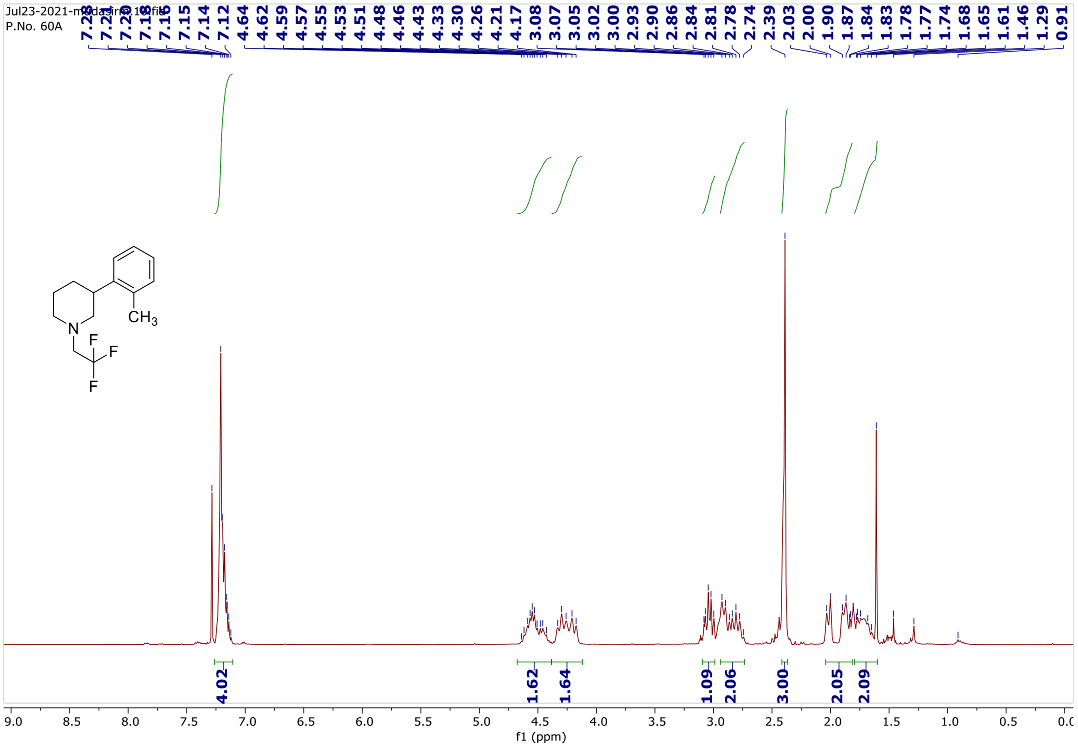

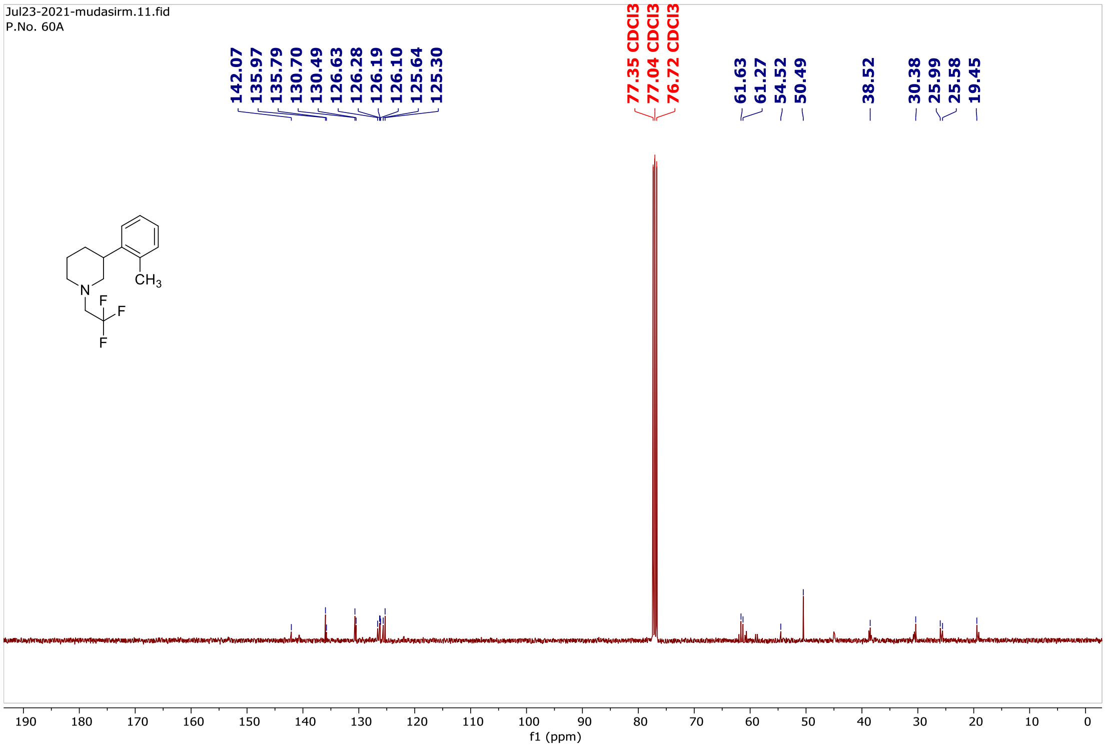

Figure S23: Top: 400 MHz ^{1}H-NMR of 3-(o-tolyl)-1-(2,2,2-trifluoroethyl)piperidine. Bottom: 100 MHz ^{13}C-NMR of 3-(o-tolyl)-1-(2,2,2-trifluoroethyl)piperidines ***(10)***.

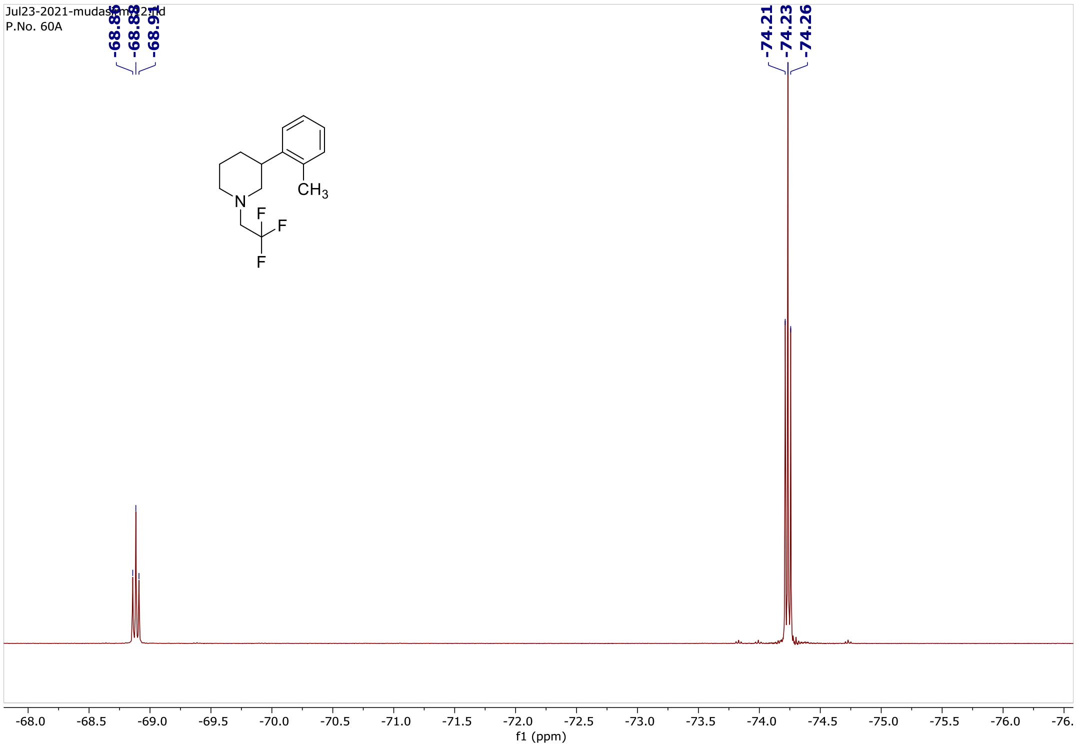

Figure S24: 376 MHz 19 F-NMR of 3-(o-tolyl)-1-(2,2,2-trifluoroethyl)piperidines **(10)**.

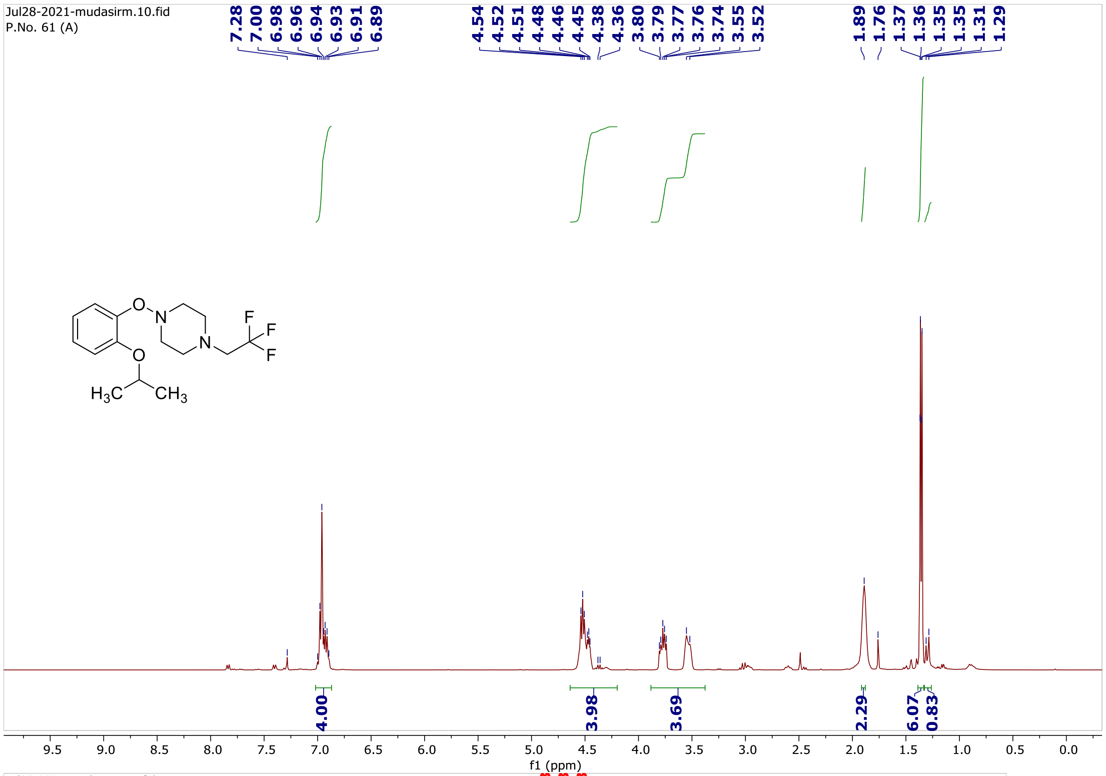

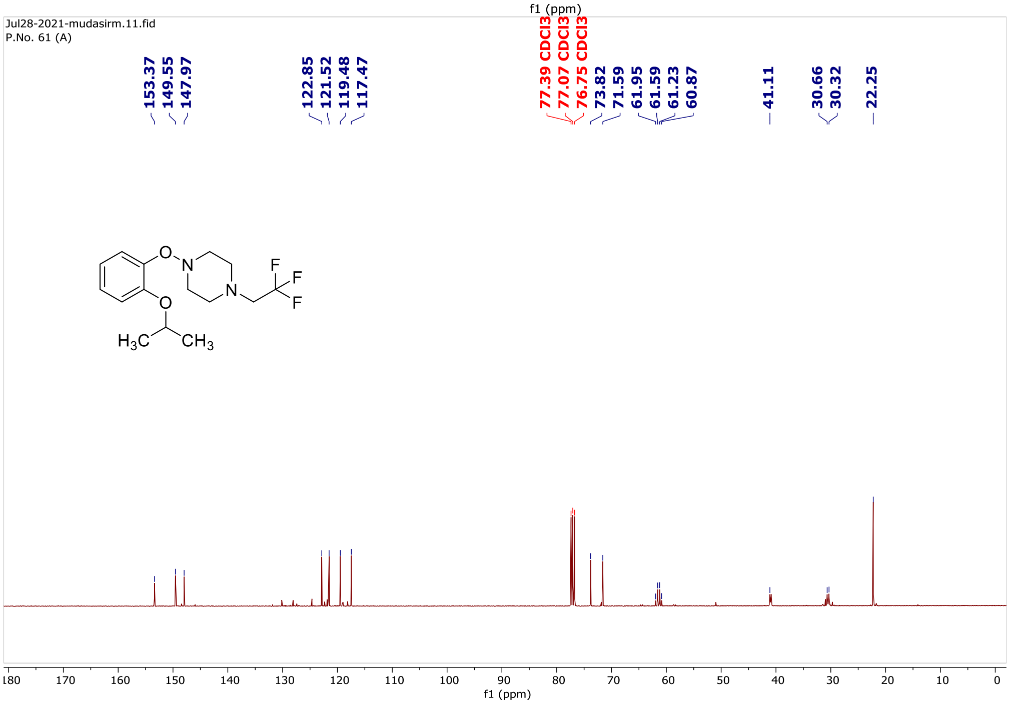

Figure S25: Top: 400 MHz ^{1}H-NMR of 1-(2-isopropoxyphenoxy)-4-(2,2,2-trifluoroethyl)piperazine **(11)**. Bottom: 100 MHz ^{13}C-NMR of 1-(2-isopropoxyphenoxy)-4-(2,2,2-trifluoroethyl)piperazine ***(11)***.

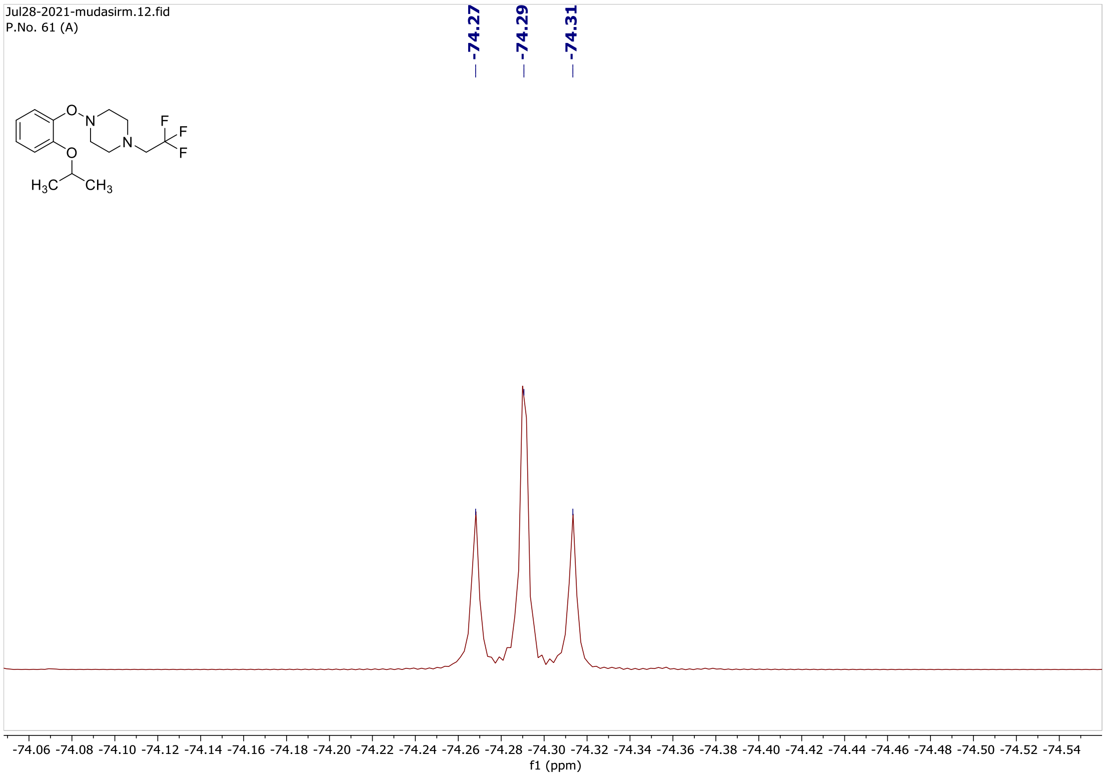

Figure S26: *376 MHz 19 F-NMR of* 1-(2-isopropoxyphenoxy)-4-(2,2,2-trifluoroethyl)piperazine ***(11)***.

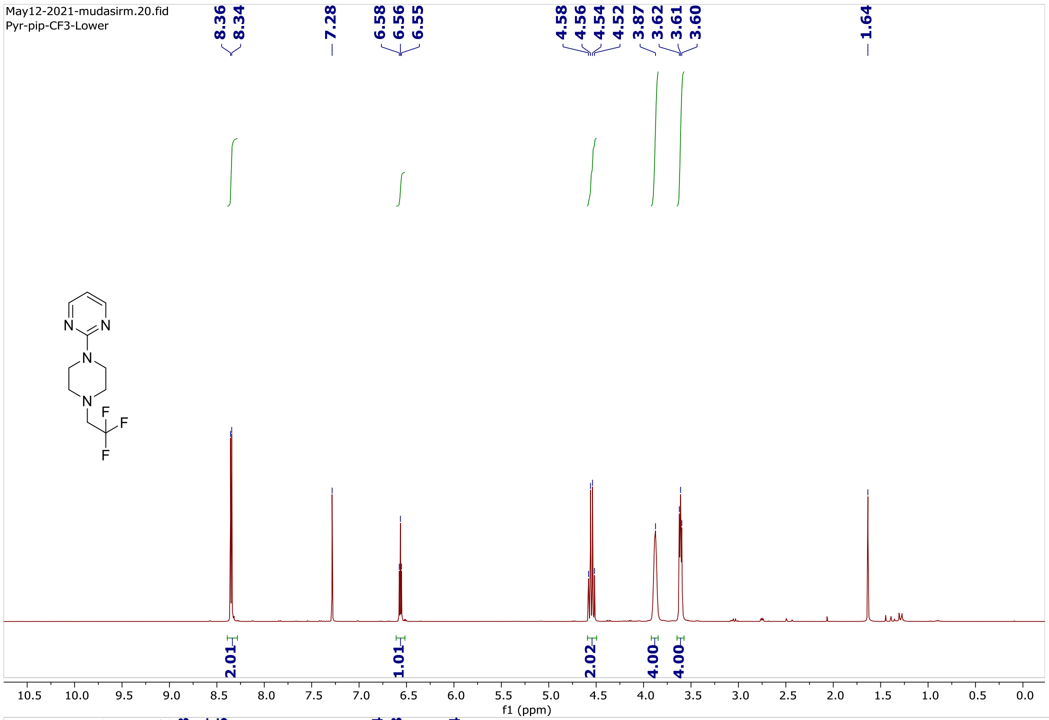

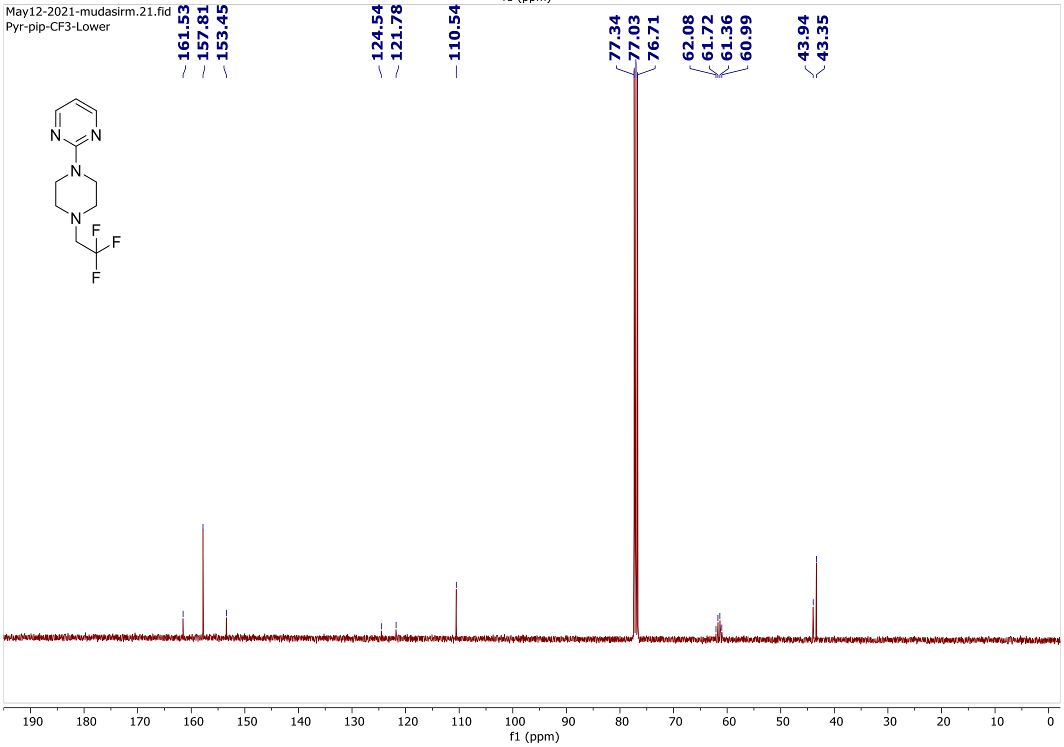

Figure S27: Top: 400 MHz ^{1}H-NMR of 2-(4-(2,2,2-trifluoroethyl)piperazin-1-yl)pyrimidine **(12)**. Bottom: 100 MHz ^{13}C-NMR of 2-(4-(2,2,2-trifluoroethyl)piperazin-1-yl)pyrimidine **(12)**.

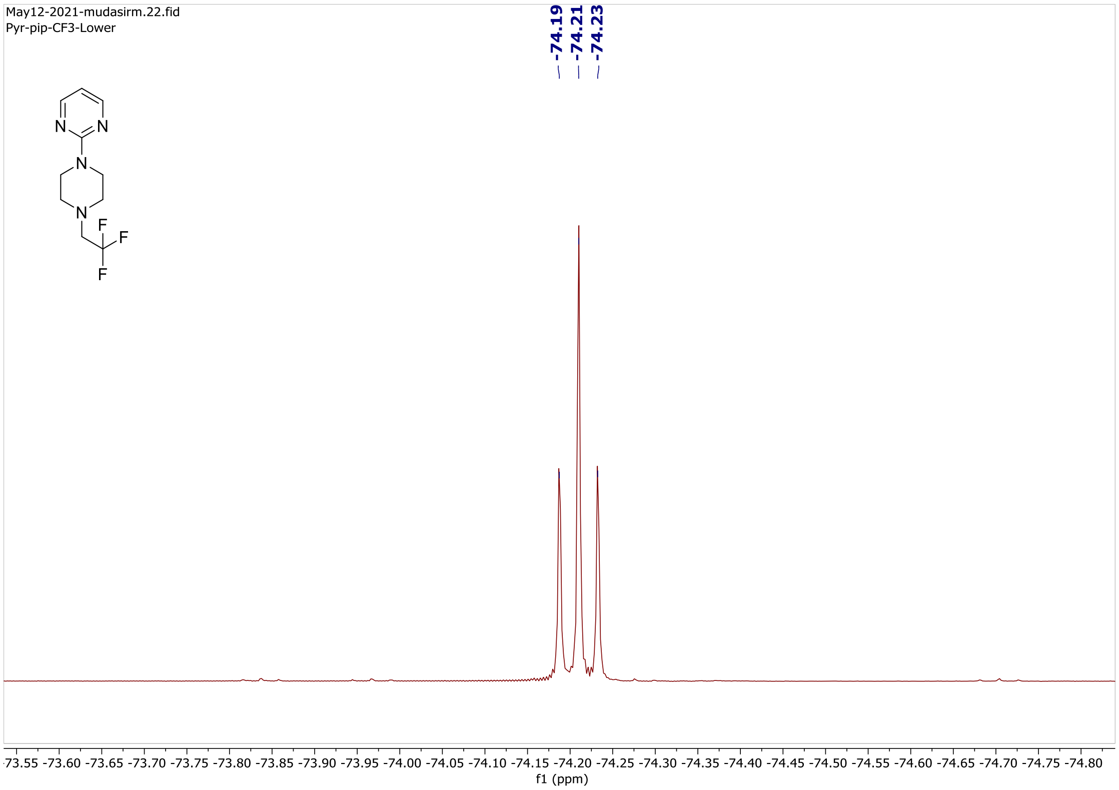

Figure S28: 376 MHz ^{19} F-NMR of2-(4-(2,2,2-trifluoroethyl)piperazin-1-yl)pyrimidine **(12)**.

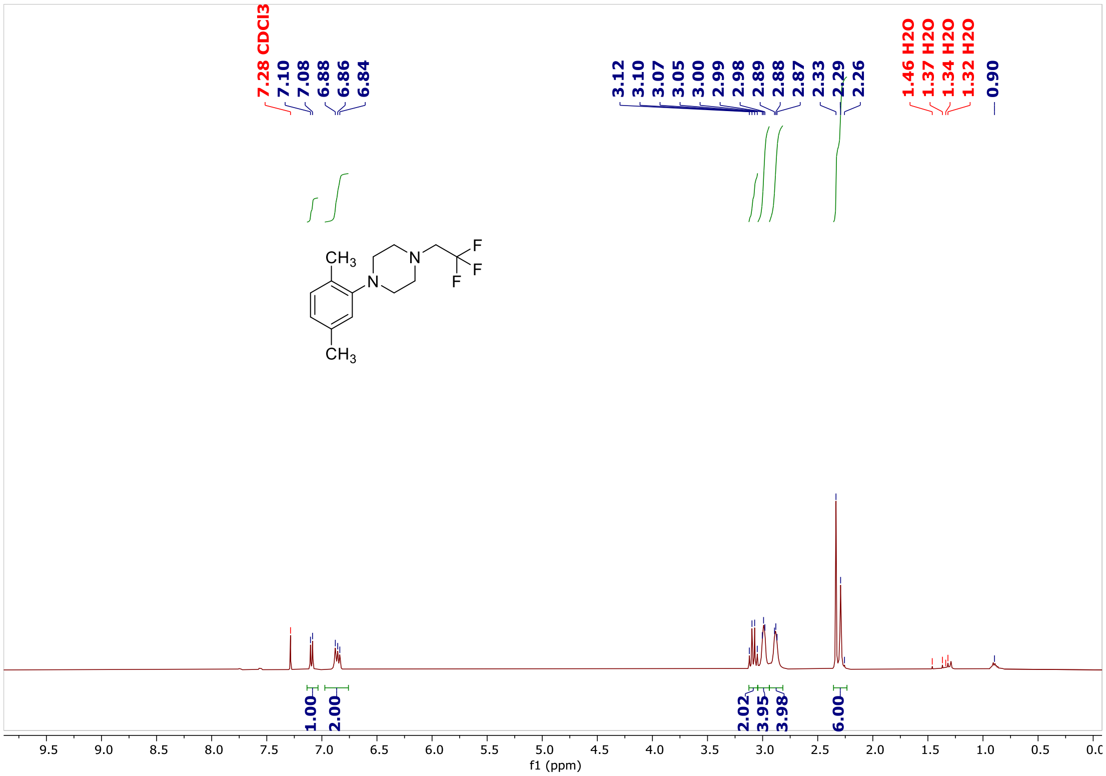

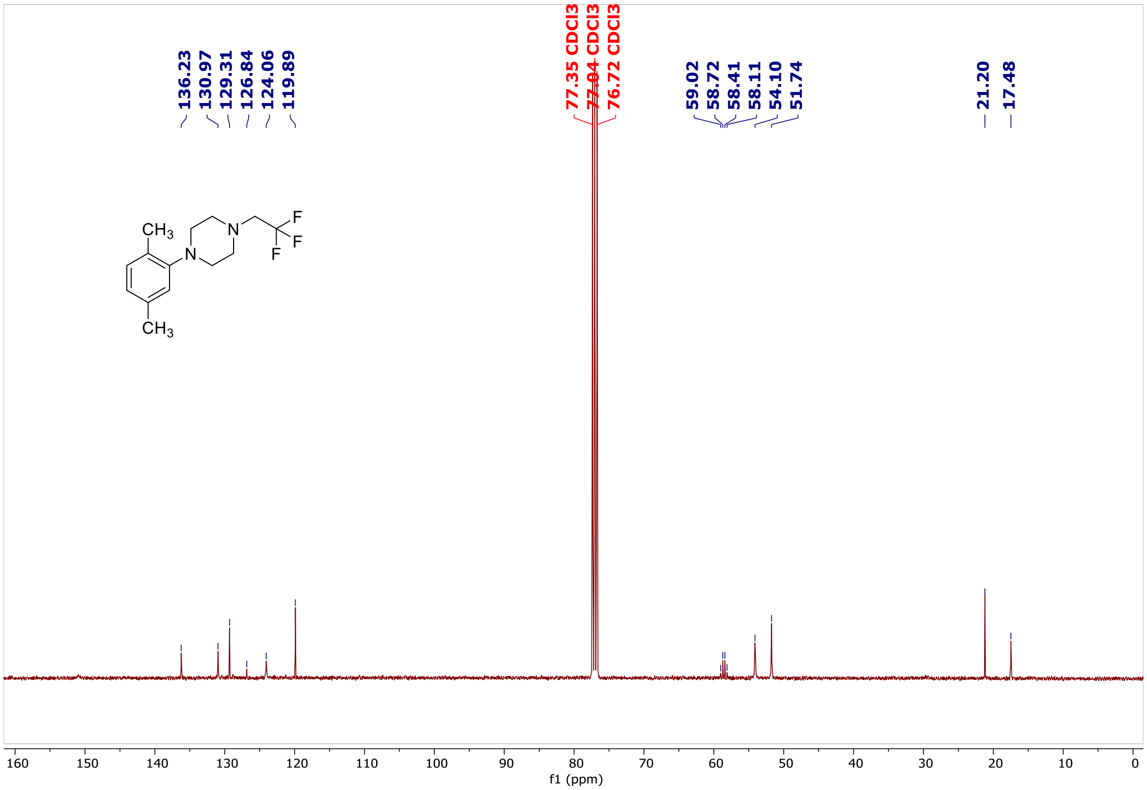

*Figure S29: Top: 400 MHz 1H-NMR of 1-(2,5-dimethylphenyl)-4-(2,2,2-trifluoroethyl)piperazine **(13)**. Bottom: 100 MHz 13C-NMR of 1-(2,5-dimethylphenyl)-4-(2,2,2-trifluoroethyl)piperazine **(13)**.*

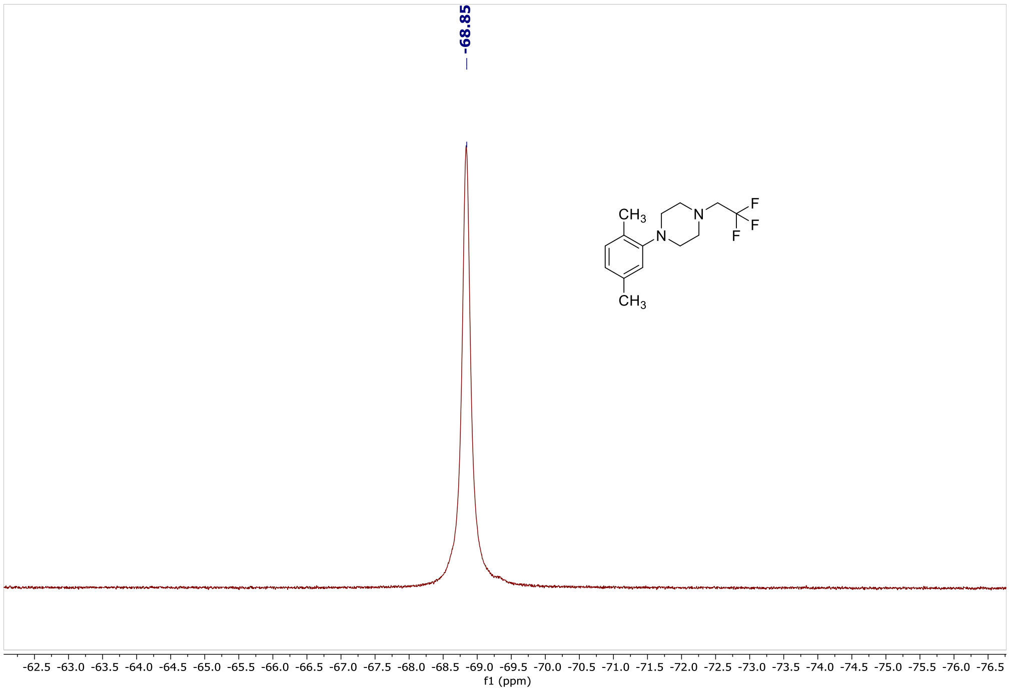

Figure S30: 376 MHz [19] F-NMR of 1-(2,5-dimethylphenyl)-4-(2,2,2-trifluoroethyl)piperazine **(13)**.

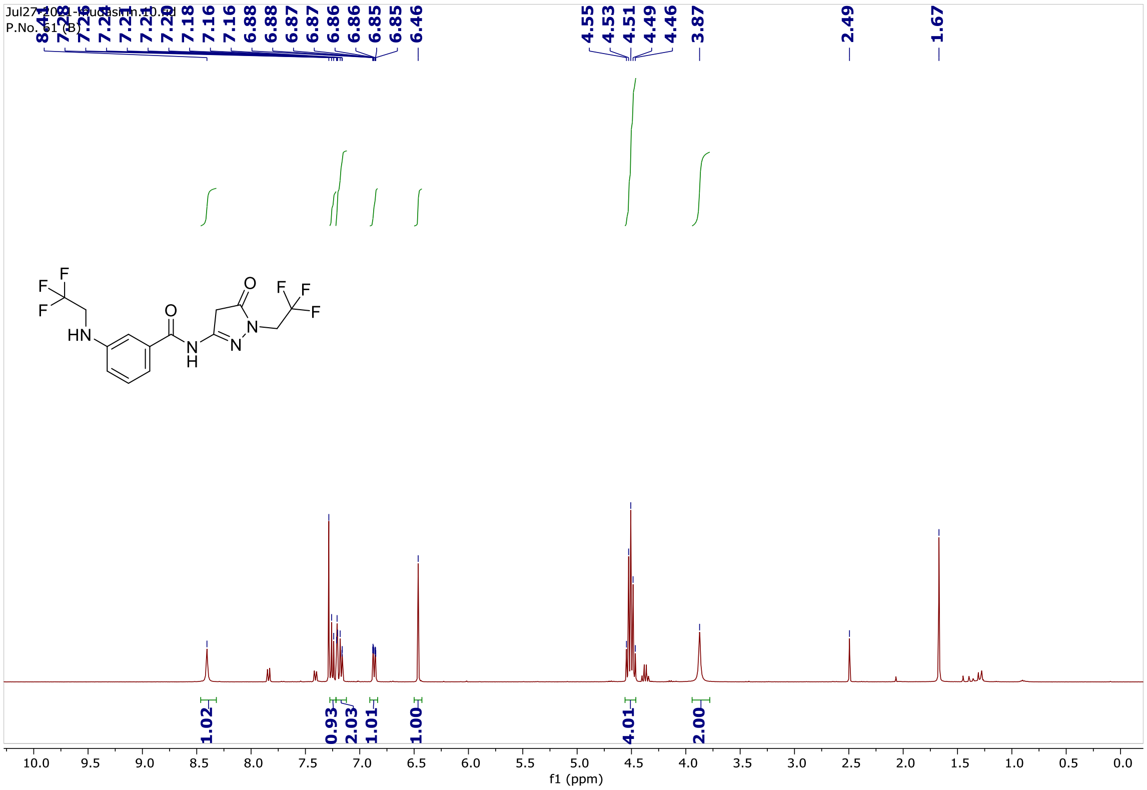

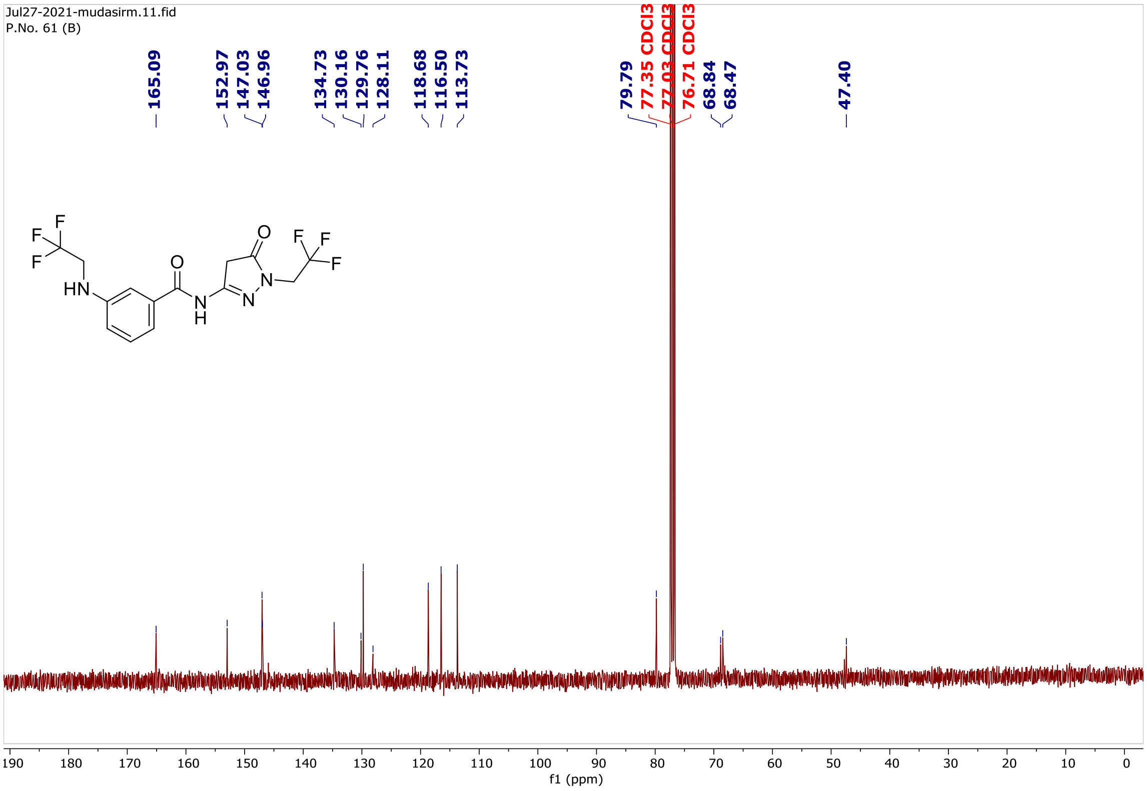

*Figure S31: Top: 400 MHz 1H-NMR of N-(5-oxo-1-(2,2,2-trifluoroethyl)-4,5-dihydro-1H-pyrazol-3-yl)-3-((2,2,2-trifluoroethyl)amino)benzamide **(14)**. Bottom: 100 MHz 13C-NMR of N-(5-oxo-1-(2,2,2-trifluoroethyl)-4,5-dihydro-1H-pyrazol-3-yl)-3-((2,2,2-trifluoroethyl)amino)benzamide **(14)**.*

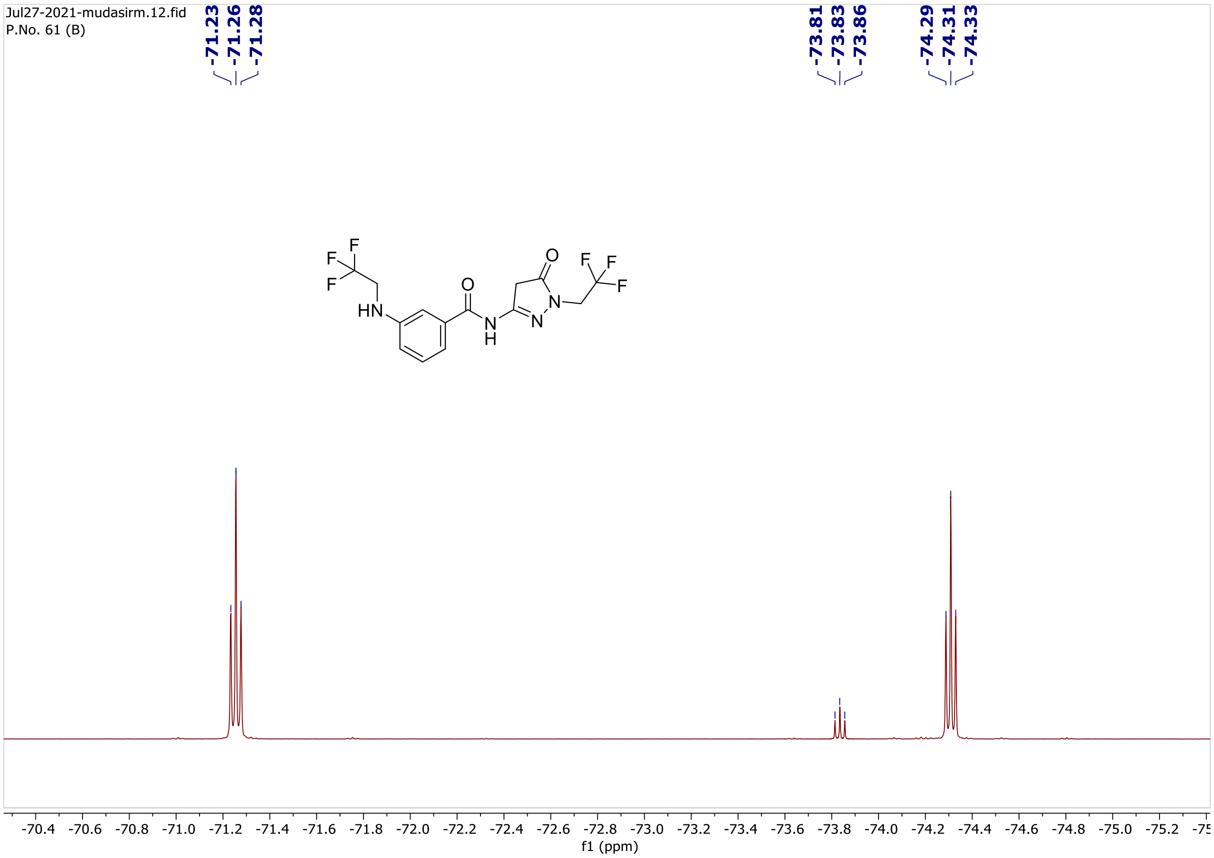

Figure S32: 376 MHz ^{19}F-NMR of N-(5-oxo-1-(2,2,2-trifluoroethyl)-4,5-dihydro-1H-pyrazol-3-yl)-3-((2,2,2-trifluoroethyl)amino)benzamide **(14)**.

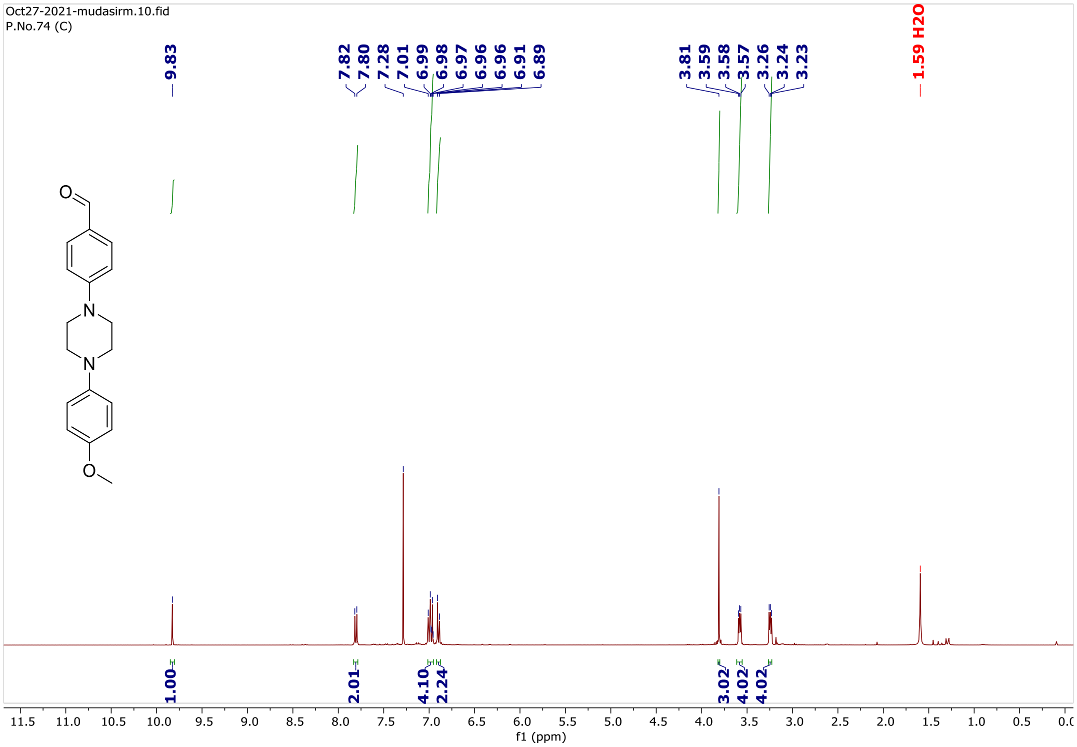

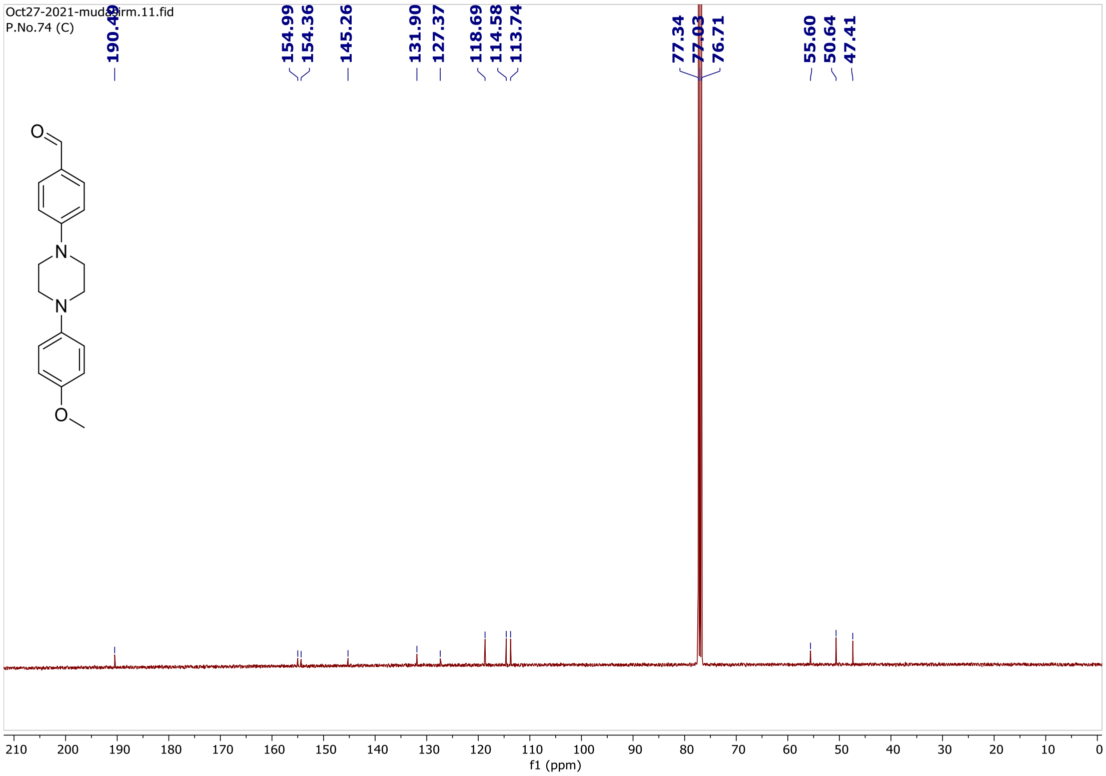

Figure S33: *Top: 400 MHz ^{1}H-NMR of 4-(4-(4-methoxyphenyl)piperazin-1-yl)benzaldehyde. Bottom: 100 MHz ^{13}C-NMR of 4-(4-(4-methoxyphenyl)piperazin-1-yl)benzaldehyde.*
