# Supplementary material for: A protocol for controlled reactivity shift in the 2,2-difluorovinyl motif used for selective S–18F and C–18F bond formation
Source: Commun Chem. 2024 Apr 29;7:97. doi: 10.1038/s42004-024-01132-3 (PMC11058245; doi:10.1038/s42004-024-01132-3)
Supplement: Supplementary file 4 — Supplementary data 2 [file 42004_2024_1132_MOESM4_ESM.pdf]

Supplementary Data 2

Controlled reactivity shift in the 2,2-difluorovinyl motif: A protocol for selective S–^{18}F and C–^{18}F bond formation

Mudasir Maqbool[1,2], Jimmy Erik Jakobsson[1], Santosh Reddy Alluri[1], Vasko Kramer[3] and Patrick Johannes Riss*[1,2,4]
DOI: 10.xxxxxxxx

[1]Department of Clinical Neurocience, OUS-Ullevål, Oslo, Norway.
[2]Department of Chemistry, University of Oslo, Oslo, Norway. Tel: +4795028669; E-mail: patrick.riss@kjemi.uio.no
[3]Positronpharma SA, Rancangua, Santiago de Chile, Chile
[4]Department of Chemistry, Johannes Gutenberg-University, Fritz-Strassmann-Weg 2, 55128 Mainz, Germany. Tel. +49 6131 39 28081; Email: priss@uni-mainz.de

Chromatograms

Measurement 250122-CF2-precursor Uni Oslo Page 1/1
c:\GINA_NT\LUNAPFP Mudasir AcN_H2O 50_50\250122-CF2-precursor

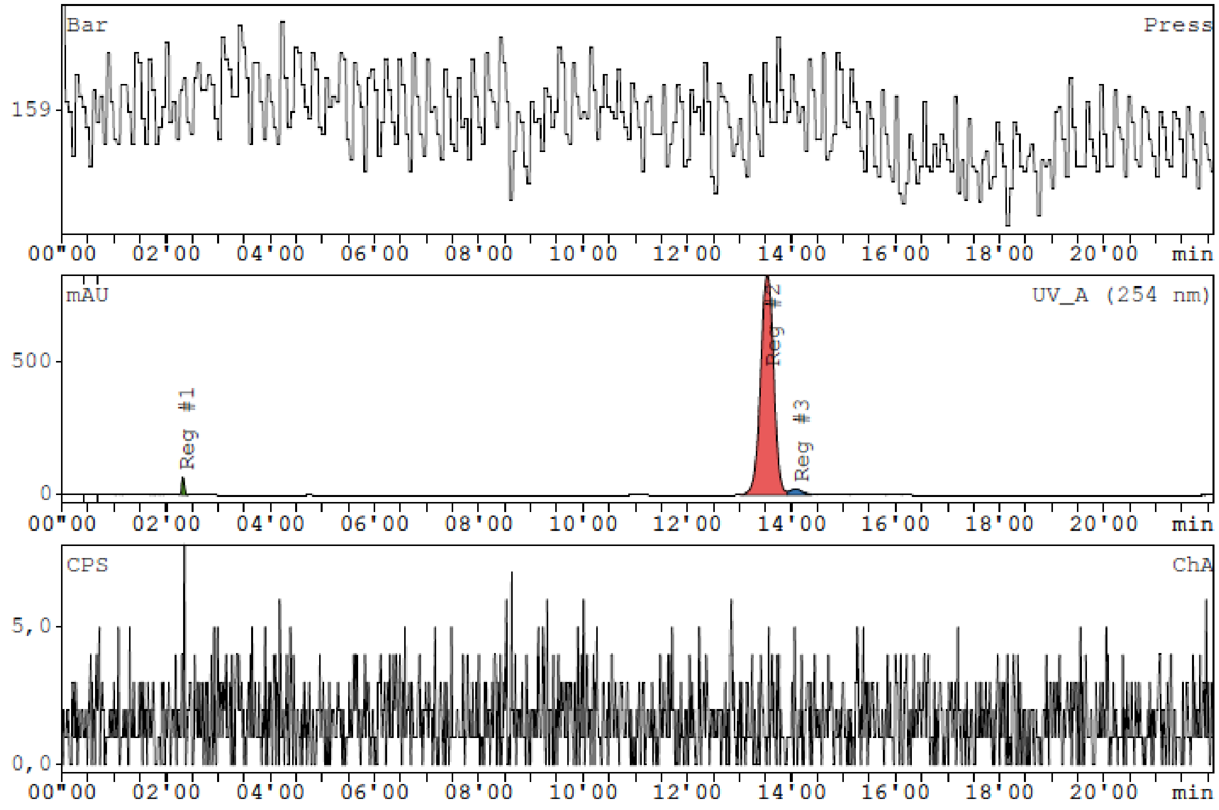

Measurement:	250122-CF2-precursor, injection : 25.01.2022 13:05
Method:	LUNAPFP Mudasir AcN_H2O 50_50 from: 17.12.2020 09:30
CH3CN:H2O=50:50, Flow rate 1,5 mL/min, injection 10 micro L	
Radio detector: raytest Gabi Star	Serial Nr.: #30745 raytest GINA star 20.04.09 Firmware V4.8
Software Version: 5.9, Service Pack 8, Build 5076	

UV_A (254 nm)

**Substance**	**R/T s**	**Type**	**Area mAU*s**	**%Area %**
Reg #1	02'21	DD(M	209,30	1,40
Reg #2	13'32	DD(M	14380,18	96,12
Reg #3	14'09	DD(M	370,86	2,48
Sum in ROI			14960,33	100,00
Area (total)			14861,21	
BKG1			-0,018	

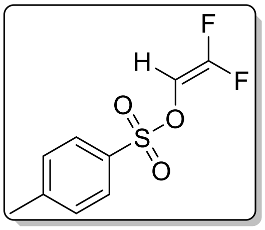

Figure S34: Analytical HPLC chromatogram from screening, top channel = UV, bottom channel = activity. HPLC spectrum of 2,2-difluorovinyl 4-methylbenzenesulfonate **(1a)**. 1 mg precursor dissolved in 1 mL MeCN: H_2O =50:50. **(**MeCN: H_2O =50:50. Flow rate = 1.5 mL/min. Injected volume = 10 uL).

Measurement 250122-CF3-Tosyl-refernce Uni Oslo Page 1/1
c:\GINA_NT\LUNAPFP Mudasir AcN_H2O 50_50\250122-CF3-Tosyl-refernce

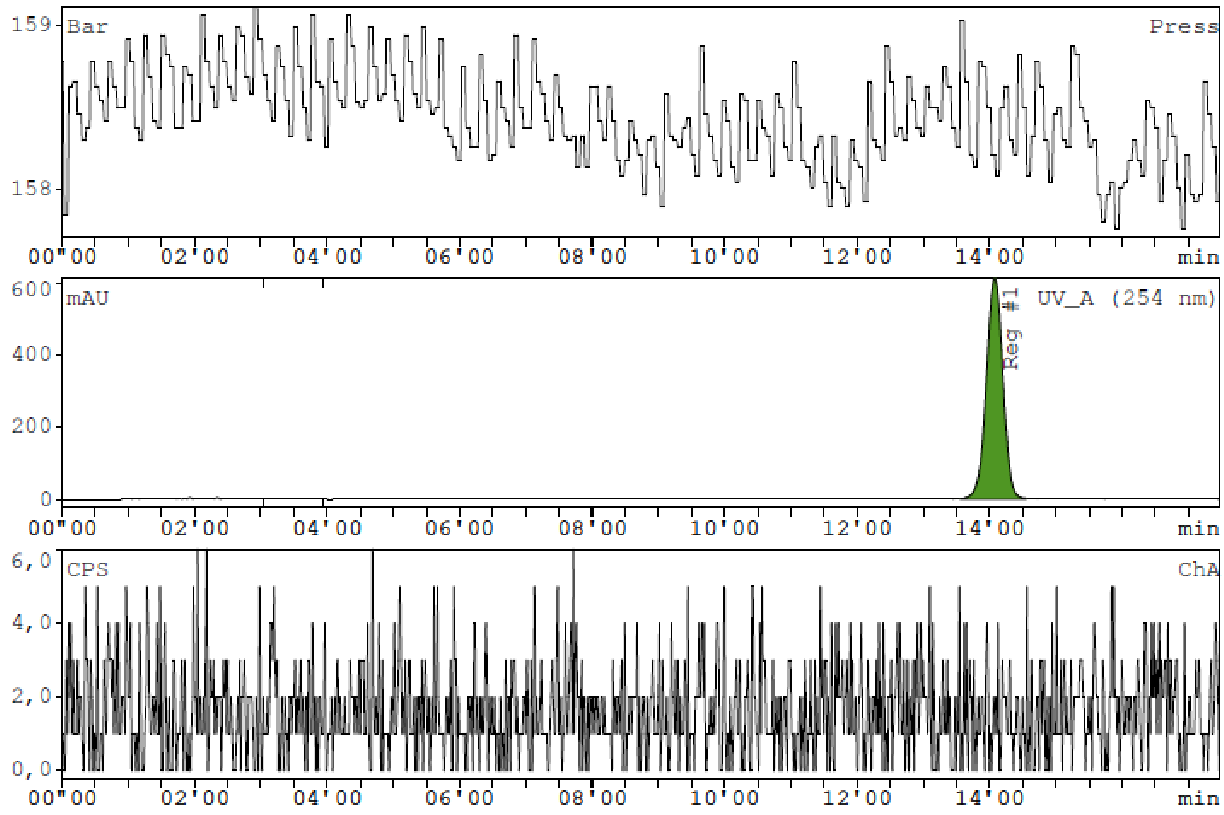

Measurement:	250122-CF3-Tosyl-refernce, injection :	25.01.2022 13:28
Method:	LUNAPFP Mudasir AcN_H2O 50_50 from:	17.12.2020 09:30

CH3CN:H2O=50:50, Flow rate 1,5 mL/min, injection 10 micro L
Radio detector: raytest Gabi Star Serial Nr.: #30745 raytest GINA star 20.04.09 Firmware V4.8
Software Version: 5.9, Service Pack 8, Build 5076

UV_A (254 nm)

**Substance**	**R/T s**	**Type**	**Area mAU*s**	**%Area %**
Reg #1	14'05	DD(M	10626,10	100,00
Sum in ROI			10626,10	100,00
Area (total)			10917,22	
BKG1			0,205	

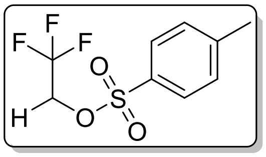

Figure S35: Analytical HPLC chromatogram from screening, top channel = UV, bottom channel = activity. HPLC spectrum of 2,2,2-trifluoroethyl 4-methylbenzenesulfonate **(1b)**. 1 mg precursor dissolved in 1 mL MeCN: H2O=50:50. **(**MeCN: H2O=50:50. Flow rate = 1.5 mL/min. Injected volume = 10 uL).

Measurement 250122-tosylfluoride Uni Oslo Page 1/1
c:\GINA_NT\LUNAPFP Mudasir AcN_H2O 50_50\250122-tosylfluoride

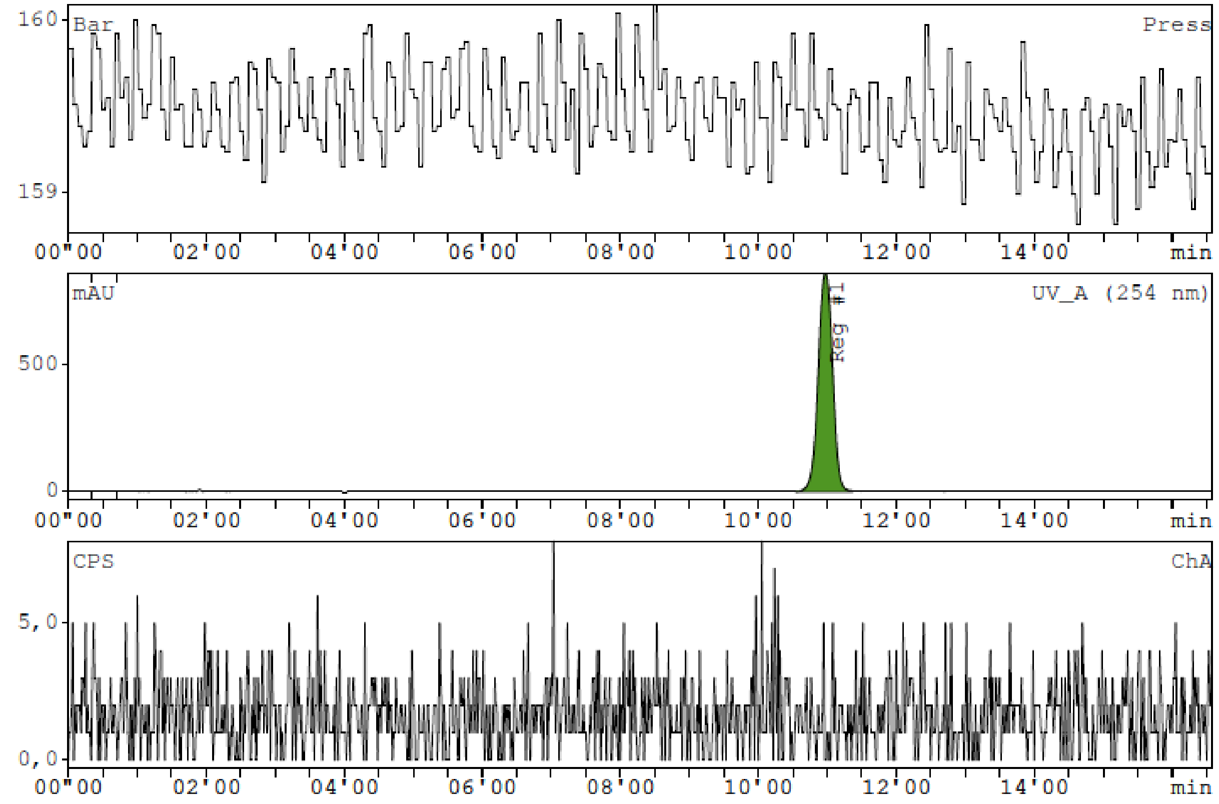

Measurement:	250122-tosylfluoride, injection :	25.01.2022 12:44
Method:	LUNAPFP Mudasir AcN_H2O 50_50 from:	17.12.2020 09:30

CH3CN:H2O=50:50, Flow rate 1,5 mL/min, injection 10 micro L
Radio detector: raytest Gabi Star Serial Nr.: #30745 raytest GINA star 20.04.09 Firmware V4.8
Software Version: 5.9, Service Pack 8, Build 5076

UV_A (254 nm)

**Substance**	**R/T s**	**Type**	**Area mAU*s**	**%Area %**
Reg #1	10'59	DD(M	12346,47	100,00
Sum in ROI			12346,47	100,00
Area (total)			12466,17	
BKG1			-0,026	

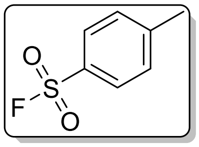

Figure S36: Analytical HPLC chromatogram from screening, top channel = UV, bottom channel = activity. HPLC spectrum of 4-methylbenzenesulfonyl fluoride. 1 mg precursor dissolved in 1 mL MeCN: H2O =50:50. **(**MeCN: H2O=50:50. Flow rate = 1.5 mL/min. Injected volume = 10 uL).

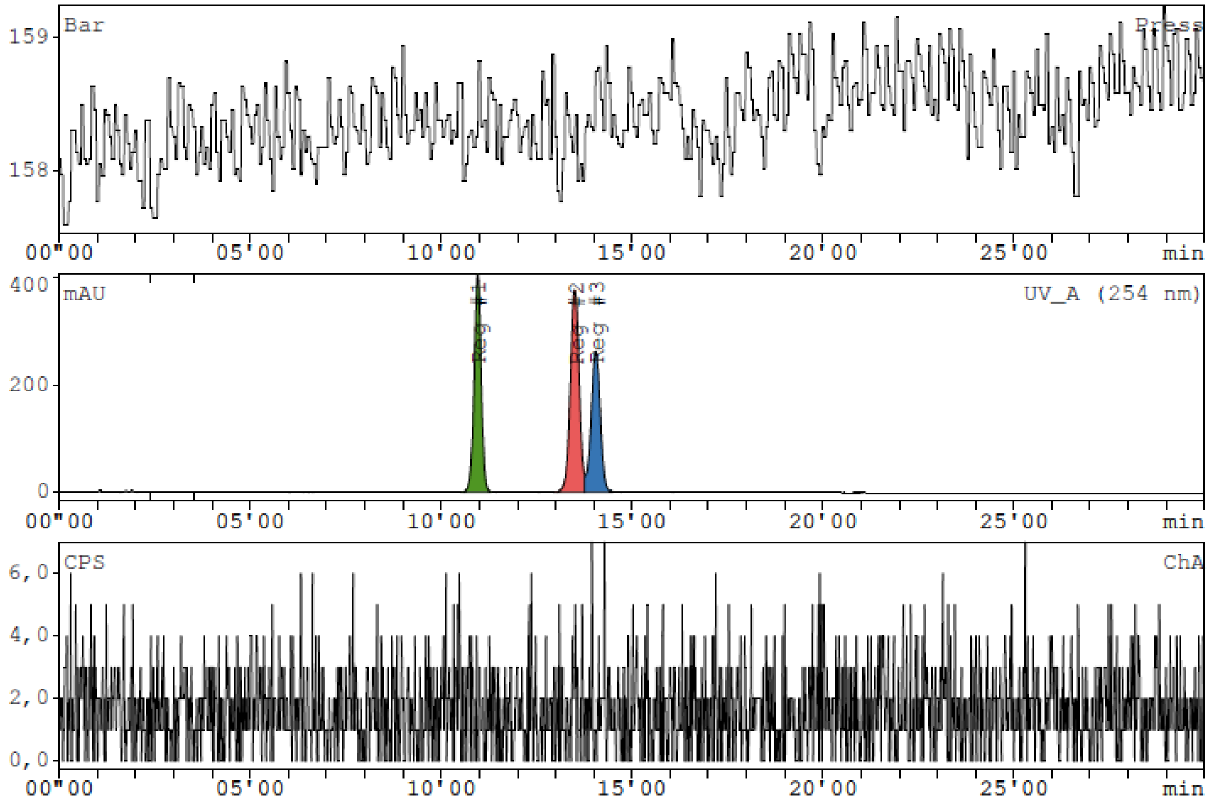

Measurement:	250122-co-injection, injection : 25.01.2022 13:46
Method:	LUNAPFP Mudasir AcN_H2O 50_50 from: 17.12.2020 09:30
CH3CN:H2O=50:50, Flow rate 1,5 mL/min, injection 10 micro L	
Radio detector: raytest Gabi Star	Serial Nr.: #30745 raytest GINA star 20.04.09 Firmware V4.8
Software Version: 5.9, Service Pack 8, Build 5076	

UV_A (254 nm)

Substance	R/T s	Type	Area mAU*s	%Area %
Reg #1	10'58	DD(M	5152,850	33,38
Reg #2	13'30	DD(M	5928,971	38,41
Reg #3	14'03	DD(M	4353,091	28,20
Sum in ROI			15434,912	100,00
Area (total)			14507,892	
BKG1			-0,4972	

Figure S37: Analytical HPLC chromatogram from screening, top channel = UV, bottom channel = activity. HPLC spectrum of 2,2-difluorovinyl 4-methylbenzenesulfonate **(1a),** 4-methylbenzenesulfonyl fluoride and 2,2,2-trifluoroethyl 4-methylbenzenesulfonate **(1b),** together as a co-injection. (MeCN: H2O=50:50. Flow rate = 1.5 mL/min. Injected volume = 10 uL).

Scheme 1, Pathway I:

Measurement CF3CH2WAg Uni Oslo Page 1/2
c:\GINA_NT\18F-Lansoprazole\CF3CH2WAg

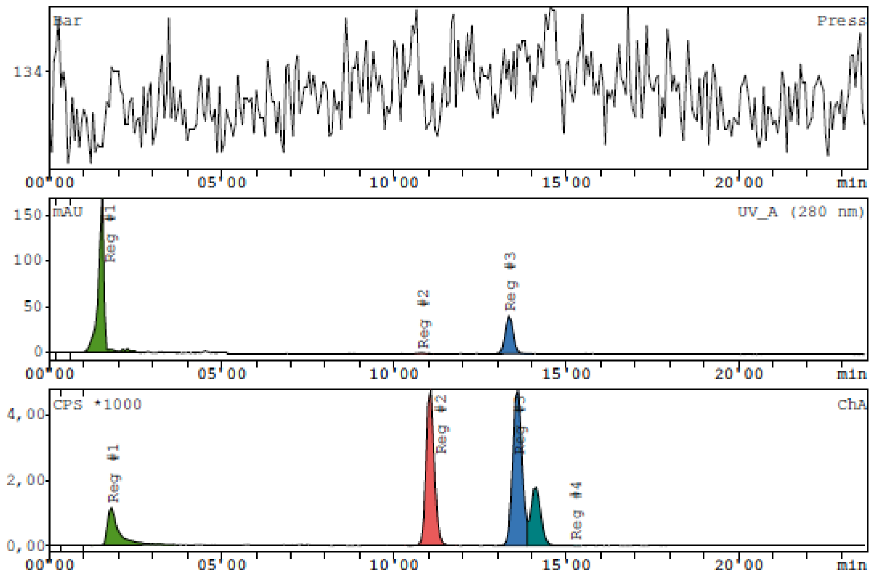

Measurement: CF3CH2WAg, injection : 16.12.2016 16:22
Method: 18F-LANSOPRAZOLE from: 16.12.2016 15:49
Mobile Phase: AcN:Water:30:70
Flow rate: 2 mL/mim
PFP (2) 150 x 4.6 mm
Method changed: 09/12/2016
Mobile Phase: AcN:Water:60:40
Flow rate: 1,5 mL/mim
PFP (2) 250 x 4.6 mm
Method changed: 12/12/2016
Mobile Phase: AcN:Water:50:50
Flow rate: 1,5 mL/mim
PFP (2) 250 x 4.6 mm
Column PFP(2) 150*4.6mm (Used (250*4.6mm) 161209)
Flow rate: 2mL/min
Wavelenght: 280
Radio detector: raytest Gabi Star Serial Nr.: #30745 raytest GINA star 20.04.09 Firmware V4.8
Software Version: 5.9, Service Pack 8, Build 5076

ChA

Substance	R/T s	Type	Area Counts	%Area %
Reg #1	01'48	BB(M	25904,89	11,35
Reg #2	11'04	BB(M	76823,27	33,67
Reg #3	13'36	DD(M	87249,26	38,24
Reg #4	15'16	DD(M	38169,08	16,73
Sum in ROI			228146,51	100,00
Area (total)			236382,47	
BKG1			4,650	

Figure S38: Analytical HPLC chromatogram from screening, top channel = UV, bottom channel = activity. HPLC spectrum of 2,2-difluoro-2-(fluoro-^{18}F)ethyl 4-methylbenzenesulfonate **([^{18}F]1b).** (**1a**), DMSO, 85°C, air, 5 min. 100 µL organic phase in 1 mL MeCN: H_2O=50:50. **(**MeCN: H_2O =30:70. Flow rate = 2.0 mL/min. Injected volume = 10 µL). (*Table 1, entry 1*).

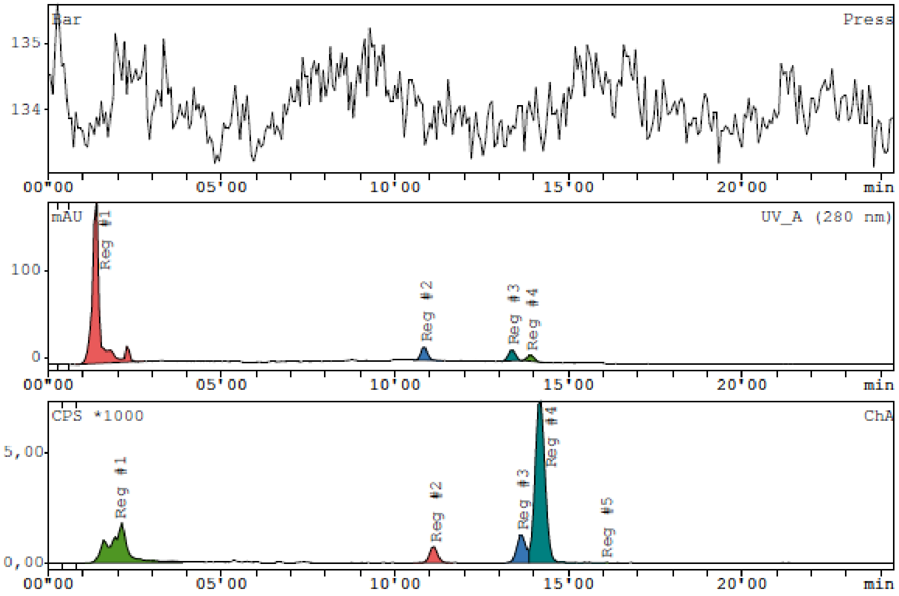

Measurement:	rx_o_Ag, injection :	16.12.2016 15:56
Method:	18F-LANSOPRAZOLE from:	16.12.2016 15:49

Mobile Phase: AcN:Water:30:70
Flow rate: 2 mL/mim
PFP (2) 150 x 4.6 mm
Method changed: 09/12/2016
Mobile Phase: AcN:Water:60:40
Flow rate: 1,5 mL/mim
PFP (2) 250 x 4.6 mm
Method changed: 12/12/2016
Mobile Phase: AcN:Water:50:50
Flow rate: 1,5 mL/mim
PFP (2) 250 x 4.6 mm
Column PFP(2) 150*4.6mm (Used (250*4.6mm) 161209)
Flow rate: 2mL/min
Wavelenght: 280
Radio detector: raytest Gabi Star Serial Nr.: #30745 raytest GINA star 20.04.09 Firmware V4.8
Software Version: 5.9, Service Pack 8, Build 5076

ChA

Substance	R/T s	Type	Area Counts	%Area %
Reg #1	02'04	DD(M	64765,2	26,45
Reg #2	11'08	DD(M	12004,0	4,90
Reg #3	13'40	DD(M	22282,2	9,10
Reg #4	14'12	DD(M	145827,2	59,54
Reg #5	16'04	DD(M	25,2	0,01
Sum in ROI			244903,8	100,00
Area (total)			256545,5	

Figure S39: Analytical HPLC chromatogram from screening, top channel = UV, bottom channel = activity. HPLC spectrum of 2,2-difluoro-2-(fluoro-^{18}F)ethyl 4-methylbenzenesulfonate **([^{18}F]1b).** (**1a**), DMSO, 85°C, N_2, 5 min. 100 µL organic phase in 1 mL MeCN: H_2O=50:50. **(**MeCN: H_2O =30:70. Flow rate = 2.0 mL/min. Injected volume = 10 µL). (*Table 1, entry 2*).

Measurement 161223_rxn10 Uni Oslo Page 1/2
c:\GINA_NT\18F-Lansoprazole_50\161223_rxn10

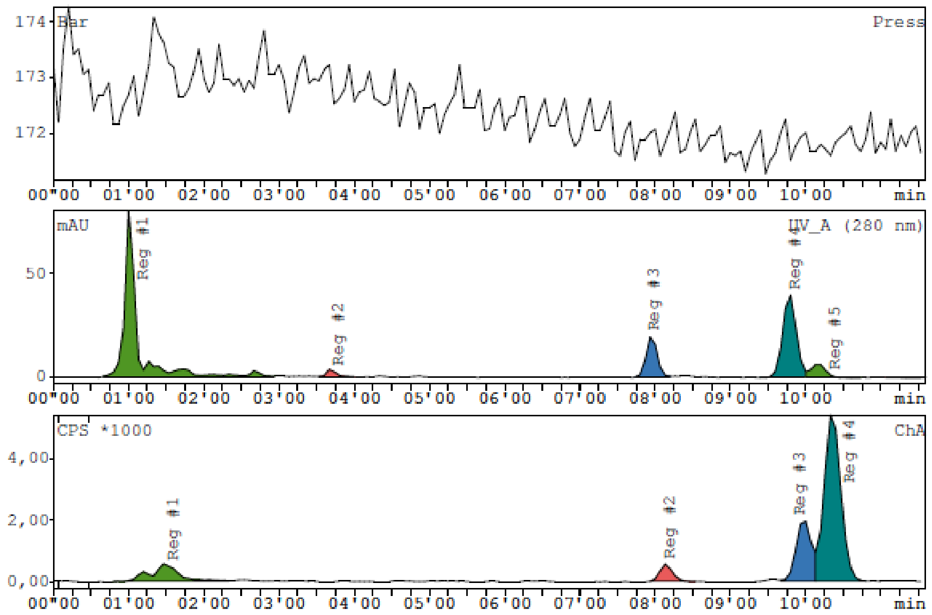

Measurement: 161223_rxn10, injection : 23.12.2016 15:25
Method: 18F-Lansoprazole_50 from: 23.12.2016 14:15
Mobile Phase: AcN:Water:30:70
Flow rate: 2 mL/mim
PFP (2) 150 x 4.6 mm
Method changed: 09/12/2016
Mobile Phase: AcN:Water:60:40
Flow rate: 1,5 mL/mim
PFP (2) 250 x 4.6 mm
Method changed: 12/12/2016
Mobile Phase: AcN:Water:50:50
Flow rate: 1,5 mL/mim
PFP (2) 250 x 4.6 mm
Column PFP(2) 150*4.6mm (Used (250*4.6mm) 161209)
Flow rate: 2mL/min
Wavelenght: 280
Radio detector: raytest Gabi Star Serial Nr.: #30745 raytest GINA star 20.04.09 Firmware V4.8
Software Version: 5.9, Service Pack 8, Build 5076

ChA

Substance	R/T s	Type	Area Counts	%Area %
Reg #1	01'32	DD(M	13758,49	10,50
Reg #2	08'08	DD(M	6178,59	4,72
Reg #3	09'52	DD(M	25691,73	19,61
Reg #4	10'20	DD(M	85411,02	65,18
Sum in ROI			131039,84	100,00
Area (total)			129169,77	
BKG1			26,001	

Figure S40*:* Analytical HPLC chromatogram from screening, top channel = UV, bottom channel = activity. HPLC spectrum of 2,2-difluoro-2-(fluoro-^{18}F)ethyl 4-methylbenzenesulfonate **([^{18}F]1b).** (**1a**), DMSO, 85°C, *i-PrOH,* N_2, 5 min. 100 µL organic phase in 1 mL MeCN: H_2O=50:50. **(**MeCN: H_2O =30:70. Flow rate = 2.0 mL/min. Injected volume = 10 µL). (*Table 1, entry 3*).

Measurement 161223_run4 Uni Oslo Page 1/2
c:\GINA_NT\18F-Lansoprazole_50\161223_run4

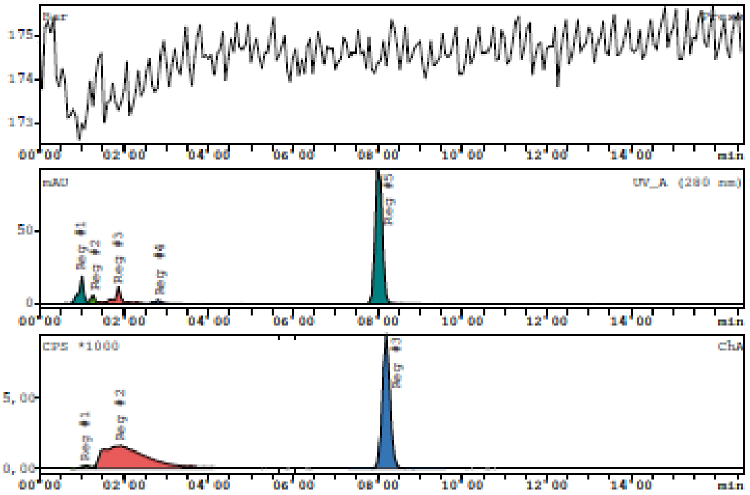

Measurement: 161223_run4, injection : 23.12.2016 13:03
Method: 18F-Lansoprazole_50 from: 23.12.2016 11:24
Mobile Phase: AcN:Water:30:70
Flow rate: 2 mL/mim
PFP (2) 150 x 4.6 mm
Method changed: 09/12/2016
Mobile Phase: AcN:Water:60:40
Flow rate: 1,5 mL/mim
PFP (2) 250 x 4.6 mm
Method changed: 12/12/2016
Mobile Phase: AcN:Water:50:50
Flow rate: 1,5 mL/mim
PFP (2) 250 x 4.6 mm
Column PFP(2) 150*4.6mm (Used (250*4.6mm) 161209)
Flow rate: 2mL/min
Wavelenght: 280
Radio detector: raytest Gabi Star Serial Nr.: #30745 raytest GINA star 20.04.09 Firmware V4.8
Software Version: 5.9, Service Pack 8, Build 5076

ChA

Substance	R/T s	Type	Area Counts	%Area %
Reg #1	01'00	DD/M	2470,3	1,07
Reg #2	01'52	DD/M	111672,8	48,25
Reg #3	08'12	DD/M	117289,7	50,68
Sum in ROI			231432,8	100,00
Area (total)			221867,7	
BKG1			33,21	
Remainder			-9565,10	-4,31

Measurement 161223_run4 Uni Oslo Page 2/2
c:\GINA_NT\18F-Lansoprazole_50\161223_run4

UV_A (280 nm)

Substance	R/T s	Type	Area mAU*s	%Area %
Reg #1	56"00	DD/M	151,152	10,92
Reg #2	01'16	DD/M	41,940	3,03
Reg #3	01'48	DD/M	133,966	9,68
Reg #4	02'48	DD/M	30,448	2,20
Reg #5	08'00	DD/M	1026,827	74,17
Sum in ROI			1384,333	100,00

Figure S41: Tosyl fluoride co-injection with [^{18}F]TsF.

Measurement 161212_Cu21_OAc Uni Oslo Page 1/2
c:\GINA_NT\18F-Lansoprazole\161212_Cu21_OAc

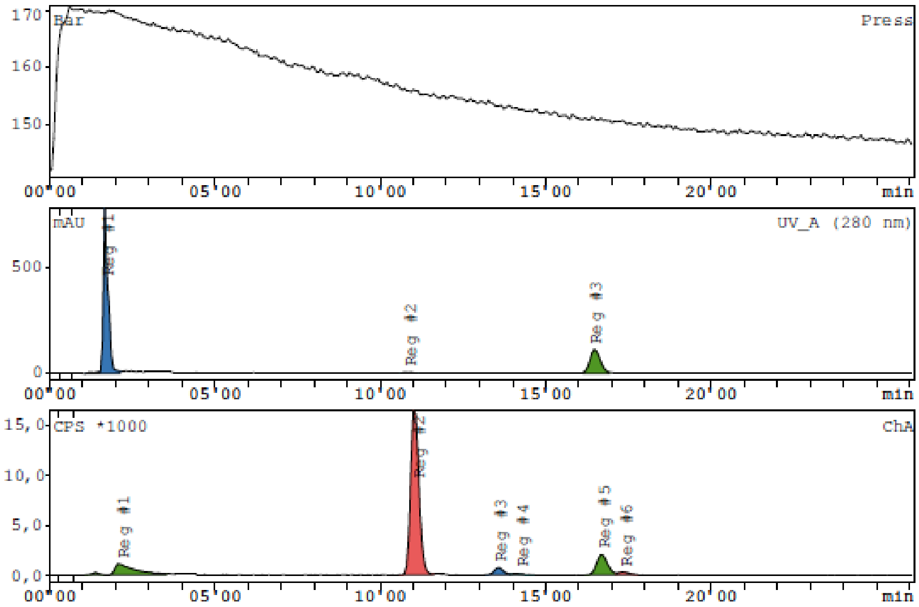

Measurement: 161212_Cu21_OAc, injection : 12.12.2016 16:00
Method: 18F-LANSOPRAZOLE from: 12.12.2016 11:45
Mobile Phase: AcN:Water:30:70
Flow rate: 2 mL/mim
PFP (2) 150 x 4.6 mm
Method changed: 09/12/2016
Mobile Phase: AcN:Water:60:40
Flow rate: 1,5 mL/mim
PFP (2) 250 x 4.6 mm
Column PFP(2) 150*4.6mm (Used (250*4.6mm) 161209)
Flow rate: 2mL/min
Wavelenght: 280
Radio detector: raytest Gabi Star Serial Nr.: #30745 raytest GINA star 20.04.09 Firmware V4.8
Software Version: 5.9, Service Pack 8, Build 5076

ChA

Substance	R/T<br>s	Type	Area<br>Counts	%Area<br>%
Reg #1	02'12	DD(M	49424,3	12,24
Reg #2	11'04	DD(M	284025,7	70,35
Reg #3	13'36	DD(M	13917,0	3,45
Reg #4	14'16	DD(M	3383,1	0,84
Reg #5	16'44	DD(M	45912,4	11,37
Reg #6	17'24	DD(M	7094,1	1,76
Sum in ROI			403756,5	100,00
Area (total)			404450,2	
BKG1			84,98	
Remainder			693,68	0,17

Figure S42: Analytical HPLC chromatogram from screening, top channel = UV, bottom channel = activity. HPLC spectrum of 2,2-difluoro-2-(fluoro-^{18}F)ethyl 4-methylbenzenesulfonate **([^{18}F]1b).** (**1a**), DMSO, 85°C, N_2, CuOAc, TEMPO, 5 min. 100 µL organic phase in 1 mL MeCN: H_2O=50:50. **(**MeCN: H_2O =30:70. Flow rate = 2.0 mL/min. Injected volume = 10 µL). (Table 1, entry 4).

Measurement 161212Cu03 Uni Oslo Page 1/2
c:\GINA_NT\18F-Lansoprazole\161212Cu03

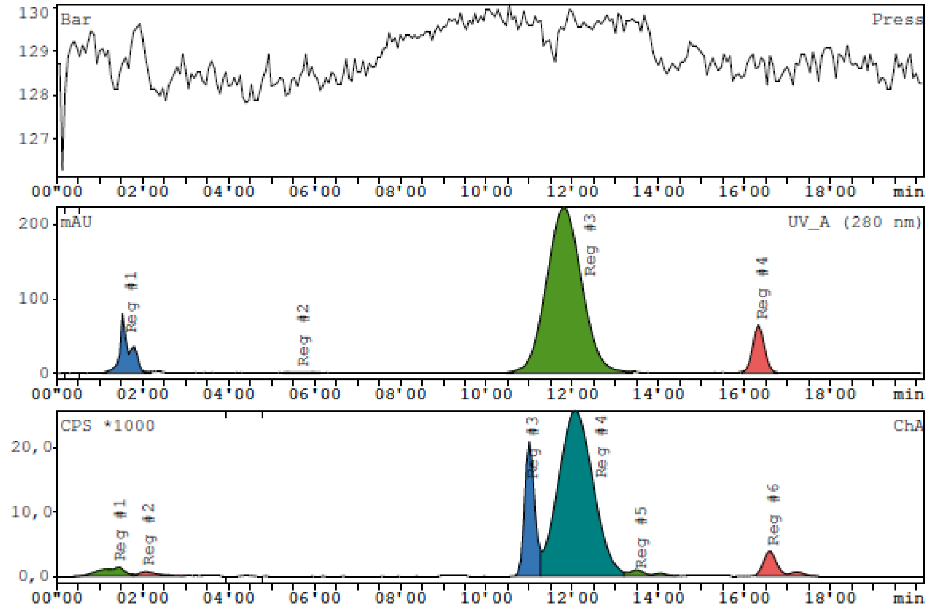

Measurement: 161212Cu03, injection : 12.12.2016 12:41
Method: 18F-LANSOPRAZOLE from: 12.12.2016 11:45
Mobile Phase: AcN:Water:30:70
Flow rate: 2 mL/mim
PFP (2) 150 x 4.6 mm
Method changed: 09/12/2016
Mobile Phase: AcN:Water:60:40
Flow rate: 1,5 mL/mim
PFP (2) 250 x 4.6 mm
Column PFP(2) 150*4.6mm (Used (250*4.6mm) 161209)
Flow rate: 2mL/min
Wavelenght: 280
Radio detector: raytest Gabi Star Serial Nr.: #30745 raytest GINA star 20.04.09 Firmware V4.8
Software Version: 5.9, Service Pack 8, Build 5076

ChA

Substance	R/T s	Type	Area Counts	%Area %
Reg #1	01'24	DD(M	50874	2,59
Reg #2	02'04	DD(M	19951	1,01
Reg #3	11'00	DD(M	326151	16,58
Reg #4	12'04	DD(M	1446011	73,49
Reg #5	13'48	DD(M	31744	1,61
Reg #6	16'36	DD(M	92964	4,72
Sum in ROI			1967695	100,00
Area (total)			1943176	
BKG1			101,3	
Remainder			-24519,45	-1,26

Figure S43: Analytical HPLC chromatogram from screening, top channel = UV, bottom channel = activity. HPLC spectrum of 2,2-difluoro-2-(fluoro-^{18}F)ethyl 4-methylbenzenesulfonate **([^{18}F]1b).** (**1a**), DMSO, 85°C, CuOAc, air, 5 min. 100 µL organic phase in 1 mL MeCN: H_2O=50:50. **(**MeCN: H_2O =30:70. Flow rate = 2.0 mL/min. Injected volume = 10 µL). (Table 1, entry 5).

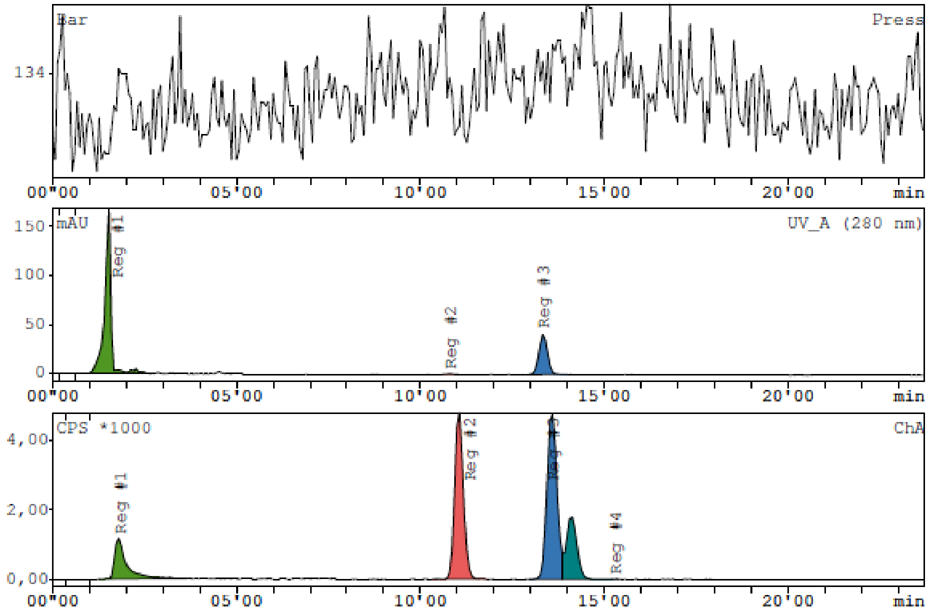

Measurement:	CF3CH2WAg, injection :	16.12.2016 16:22
Method:	18F-LANSOPRAZOLE from:	16.12.2016 15:49

Mobile Phase: AcN:Water:30:70
Flow rate: 2 mL/mim
PFP (2) 150 x 4.6 mm
Method changed: 09/12/2016
Mobile Phase: AcN:Water:60:40
Flow rate: 1,5 mL/mim
PFP (2) 250 x 4.6 mm
Method changed: 12/12/2016
Mobile Phase: AcN:Water:50:50
Flow rate: 1,5 mL/mim
PFP (2) 250 x 4.6 mm
Column PFP(2) 150*4.6mm (Used (250*4.6mm) 161209)
Flow rate: 2mL/min
Wavelenght: 280
Radio detector: raytest Gabi Star Serial Nr.: #30745 raytest GINA star 20.04.09 Firmware V4.8
Software Version: 5.9, Service Pack 8, Build 5076

ChA

Substance	R/T s	Type	Area Counts	%Area %
Reg #1	01'48	BB(M	25904,89	11,35
Reg #2	11'04	BB(M	76823,27	33,67
Reg #3	13'36	DD(M	87249,26	38,24
Reg #4	15'16	DD(M	38169,08	16,73
Sum in ROI			228146,51	100,00
Area (total)			236382,47	
BKG1			4,650	

Figure S44: Analytical HPLC chromatogram from screening, top channel = UV, bottom channel = activity. HPLC spectrum of 2,2-difluoro-2-(fluoro-^{18}F)ethyl 4-methylbenzenesulfonate **([^{18}F]1b).** (**1a**), DMSO, 85°C, CuCN, air, 5 min. 100 µL organic phase in 1 mL MeCN: H_2O=50:50. **(**MeCN: H_2O =30:70. Flow rate = 2.0 mL/min. Injected volume = 10 µL). (Table 1, entry 6).

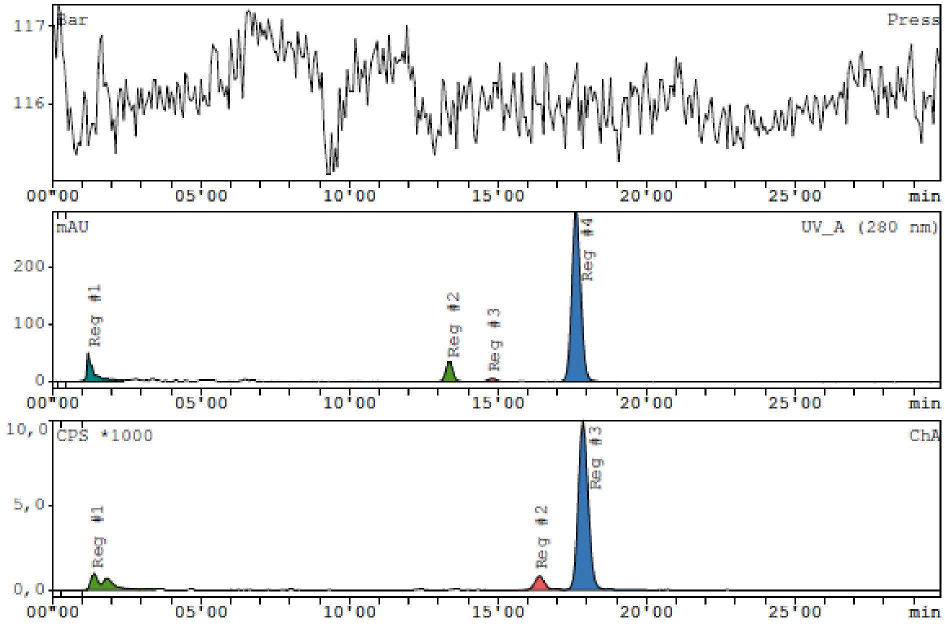

Measurement:	control_styr, injection :	15.12.2016 17:47
Method:	18F-LANSOPRAZOLE from:	15.12.2016 14:50

Mobile Phase: AcN:Water:30:70
Flow rate: 2 mL/mim
PFP (2) 150 x 4.6 mm
Method changed: 09/12/2016
Mobile Phase: AcN:Water:60:40
Flow rate: 1,5 mL/mim
PFP (2) 250 x 4.6 mm
Method changed: 12/12/2016
Mobile Phase: AcN:Water:50:50
Flow rate: 1,5 mL/mim
PFP (2) 250 x 4.6 mm
Column PFP(2) 150*4.6mm (Used (250*4.6mm) 161209)
Flow rate: 2mL/min
Wavelenght: 280
Radio detector: raytest Gabi Star Serial Nr.: #30745 raytest GINA star 20.04.09 Firmware V4.8
Software Version: 5.9, Service Pack 8, Build 5076

ChA

Substance	R/T s	Type	Area Counts	%Area %
Reg #1	01'24	DD(M	36105,4	12,65
Reg #2	16'24	DD(M	19137,8	6,70
Reg #3	17'52	DD(M	230223,6	80,65
Sum in ROI			285466,7	100,00
Area (total)			295233,1	
BKG1			6,18 CPS	
Remainder			9766,39	3,31

Figure S45: Analytical HPLC chromatogram from screening, top channel = UV, bottom channel = activity. HPLC spectrum of 2,2-difluoro-2-(fluoro-^{18}F)ethyl 4-methylbenzenesulfonate **([^{18}F]1b).** (**1a**), DMSO, 85°C, $CuBH_4$, 5 min. 100 µL organic phase in 1 mL MeCN: H_2O=50:50. **(**MeCN: H_2O =30:70. Flow rate = 2.0 mL/min. Injected volume = 10 µL). (Table 1, entry 7).

Measurement 161209_CuCN_02 Uni Oslo Page 1/2
c:\GINA_NT\18F-Lansoprazole\161209_CuCN_02

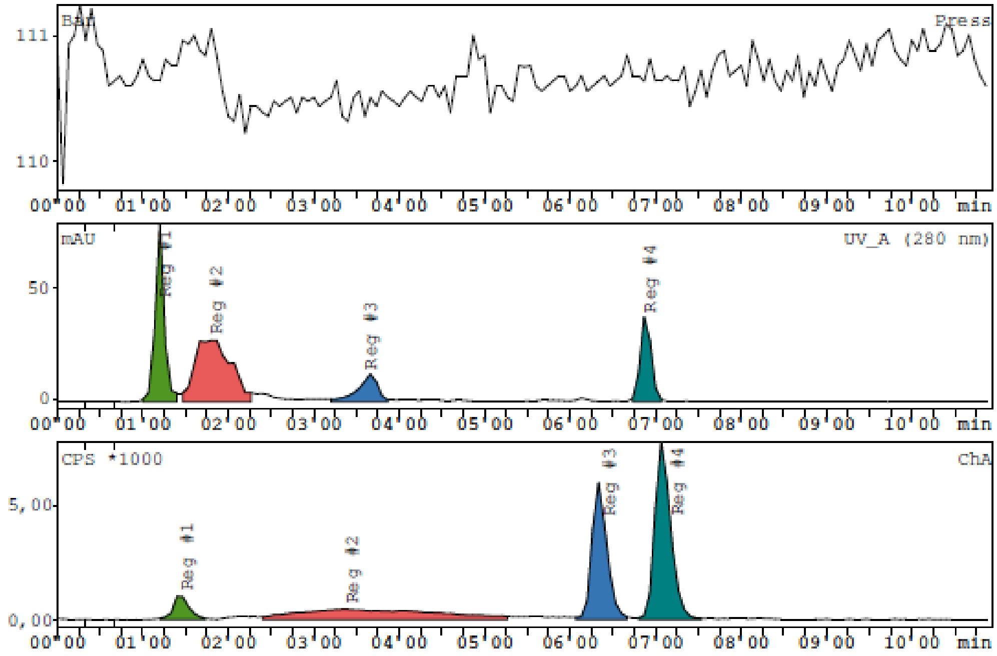

Measurement:	161209_CuCN_02, injection :	09.12.2016 15:00	
Method:	18F-LANSOPRAZOLE from:	09.12.2016 13:31	

Mobile Phase: AcN:Water:30:70
Column PFP(2) 150*4.6mm (Used (250*4.6mm) 161209)
Flow rate: 2mL/min
Wavelenght: 280
Radio detector: raytest Gabi Star Serial Nr.: #30745 raytest GINA star 20.04.09 Firmware V4.8
Software Version: 5.9, Service Pack 8, Build 5076

ChA

Substance	R/T s	Type	Area Counts	%Area %
Reg #1	01'28	DD(M	13343,74	5,68
Reg #2	03'24	DD(M	50826,15	21,63
Reg #3	06'20	DD(M	71665,26	30,50
Reg #4	07'08	DD(M	99129,73	42,19
Sum in ROI			234964,88	100,00
Area (total)			248348,45	
BKG1			44,703	
Remainder			13383,58	5,39

Figure S46: Analytical HPLC chromatogram from screening, top channel = UV, bottom channel = activity. HPLC spectrum of 2,2-difluoro-2-(fluoro-^{18}F)ethyl 4-methylbenzenesulfonate **([^{18}F]1b).** (**1a**), DMSO, 85°C, CuOAc, N_2, TEMPO, phosphite, 5 min. 100 µL organic phase in 1 mL MeCN: H_2O=50:50. **(**MeCN: H_2O =30:70. Flow rate = 2.0 mL/min. Injected volume = 10 µL). (Table 1, entry 8).

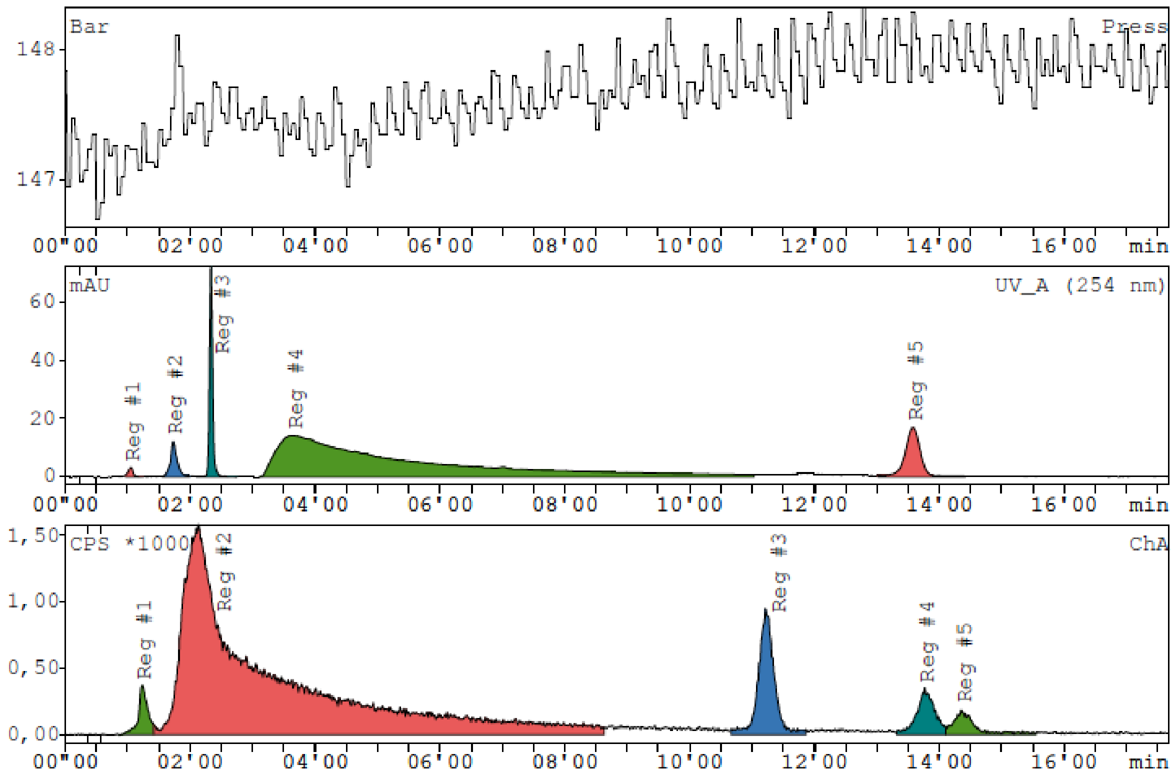

Sample description

Measurement:	181220-G, injection :	18.12.2020 13:39
Method:	LUNAPFP Mudasir AcN_H2O 50_50 from:	17.12.2020 09:30
Actonitrile:H20 =50:50, Flow 1,5 mL/min, injection 10 micro L.		
Radio detector: raytest Gabi Star	Serial Nr.: #30745 raytest GINA star 20.04.09 Firmware V4.8	
Software Version: 5.9, Service Pack 8, Build 5076		

Integration ChA

Substance	R/T s	Type	Area Counts	%Area %
Reg #1	01'14	DD(M	3681,0	2,27
Reg #2	02'07	DD(M	131600,0	81,10
Reg #3	11'14	DD(M	16107,6	9,93
Reg #4	13'46	DD(M	6647,0	4,10
Reg #5	14'23	DD(M	4235,3	2,61
Sum in ROI			162270,8	100,00
Area (total)			171900,1	
BKG1			6,50 CPS	
Remainder			9629,32	5,60

Figure S47: Analytical HPLC chromatogram from screening, top channel = UV, bottom channel = activity. HPLC spectrum of 2,2-difluoro-2-(fluoro-^{18}F)ethyl 4-methylbenzenesulfonate **([^{18}F]1b). 1a**, $(Bu)_4SnH$, DMSO, 90̊C, 6 min. 100 µL organic phase in 1 mL MeCN: H_2O =50:50. **(**MeCN: H_2O =50:50. Flow rate = 1.5 mL/min. Injected volume = 10 µL) (Table 1, entry 9).

Measurement 181220-A Uni Oslo Page 1/2
c:\GINA_NT\LUNAPFP Mudasir AcN_H2O 50_50\181220-A

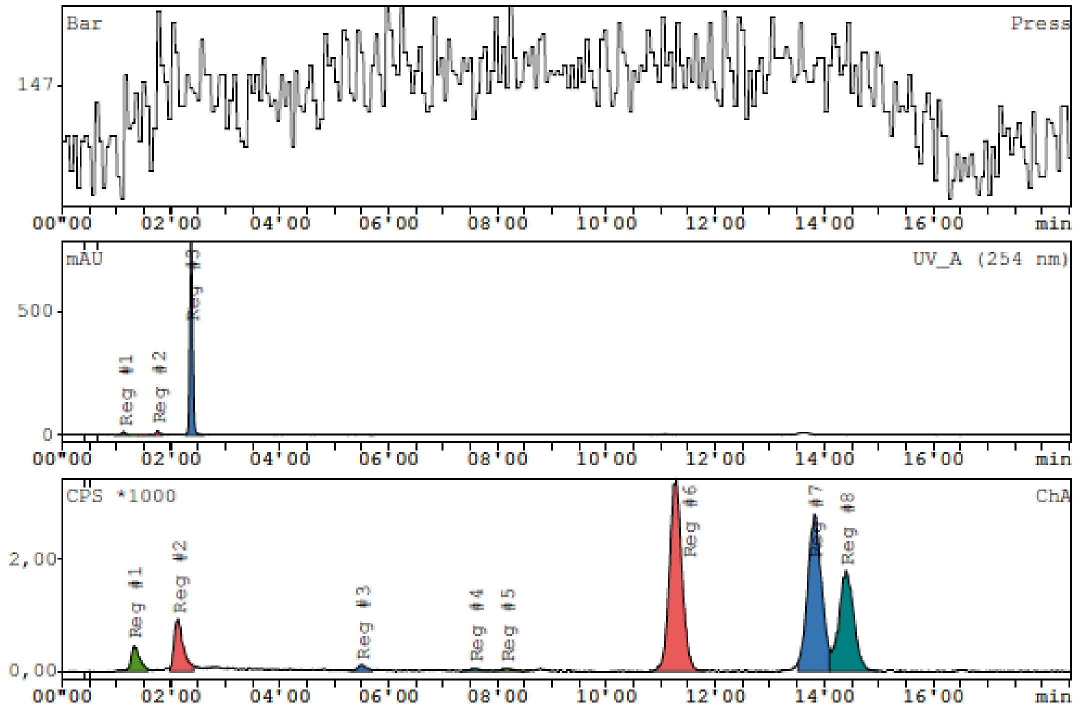

Sample description

Measurement:	181220-A, injection : 18.12.2020 10:50
Method:	LUNAPFP Mudasir AcN_H2O 50_50 from: 17.12.2020 09:30
Actonitrile:H20 =50:50, Flow 1,5 mL/min, injection 10 micro L.	
Radio detector: raytest Gabi Star	Serial Nr.: #30745 raytest GINA star 20.04.09 Firmware V4.8
Software Version: 5.9, Service Pack 8, Build 5076	

Integration ChA

Substance	R/T s	Type	Area Counts	%Area %
Reg #1	01'20	DD(M	5003,58	3,20
Reg #2	02'07	DD(M	10934,51	7,00
Reg #3	05'30	DD(M	1388,18	0,89
Reg #4	07'35	DD(M	747,67	0,48
Reg #5	08'10	DD(M	1024,81	0,66
Reg #6	11'16	DD(M	53520,57	34,25
Reg #7	13'49	DD(M	49088,80	31,42
Reg #8	14'24	DD(M	34550,02	22,11
Sum in ROI			156258,15	100,00
Area (total)			168852,06	
BKG1			2,875	
Remainder			12593,91	7,46

Figure S48: Analytical HPLC chromatogram from screening, top channel = UV, bottom channel = activity. HPLC spectrum of 2,2-difluoro-2-(fluoro-^{18}F)ethyl 4-methylbenzenesulfonate **([^{18}F]1b). 1a**, DMSO, 90°C, 6 min. 100 µL organic phase in 1 mL MeCN: H_2O =50:50. **(**MeCN: H_2O =50:50. Flow rate = 1.5 mL/min. Injected volume = 10 µL) (Table 1, entry 10).

Measurement 070121-B Uni Oslo Page 1/2
c:\GINA_NT\LUNAPFP Mudasir AcN_H2O 50_50\070121-B

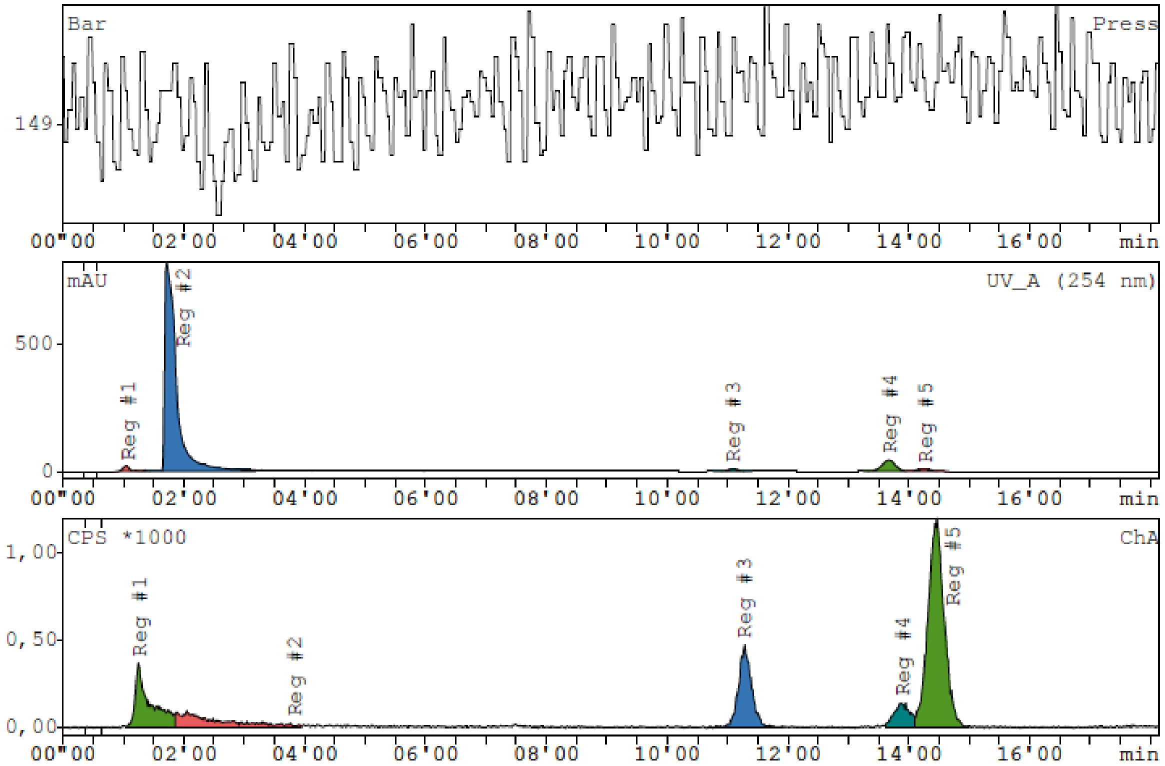

Sample description

Measurement:	070121-B, injection :	07.01.2021 13:17
Method:	LUNAPFP Mudasir AcN_H2O 50_50 from:	17.12.2020 09:30
MeCN: H2O=50:50. Flow rate = 1,5 mL/min. Injected volume = 10 uL.		
Radio detector: raytest Gabi Star	Serial Nr.: #30745 raytest GINA star 20.04.09 Firmware V4.8	
Software Version: 5.9, Service Pack 8, Build 5076		

Integration ChA

Substance	R/T s	Type	Area Counts	%Area %
Reg #1	01'15	DD(M	6178,76	14,76
Reg #2	03'50	DD(M	4265,55	10,19
Reg #3	11'16	DD(M	6897,11	16,48
Reg #4	13'53	DD(M	2493,78	5,96
Reg #5	14'27	DD(M	22018,73	52,61
Sum in ROI			41853,92	100,00
Area (total)			44411,32	
BKG1			2,471	
Remainder			2557,40	5,76

Figure S49: Analytical HPLC chromatogram from screening, top channel = UV, bottom channel = activity. HPLC spectrum of 2,2-difluoro-2-(fluoro-^{18}F)ethyl 4-methylbenzenesulfonate **([^{18}F]1b). 1a**, DMSO, 90°C, 2-propanol, 6 min. 100 µL organic phase in 1 mL MeCN: H_2O =50:50. **(**MeCN: H_2O =50:50. Flow rate = 1.5 mL/min. Injected volume = 10 µL) (Table 1, entry 11).

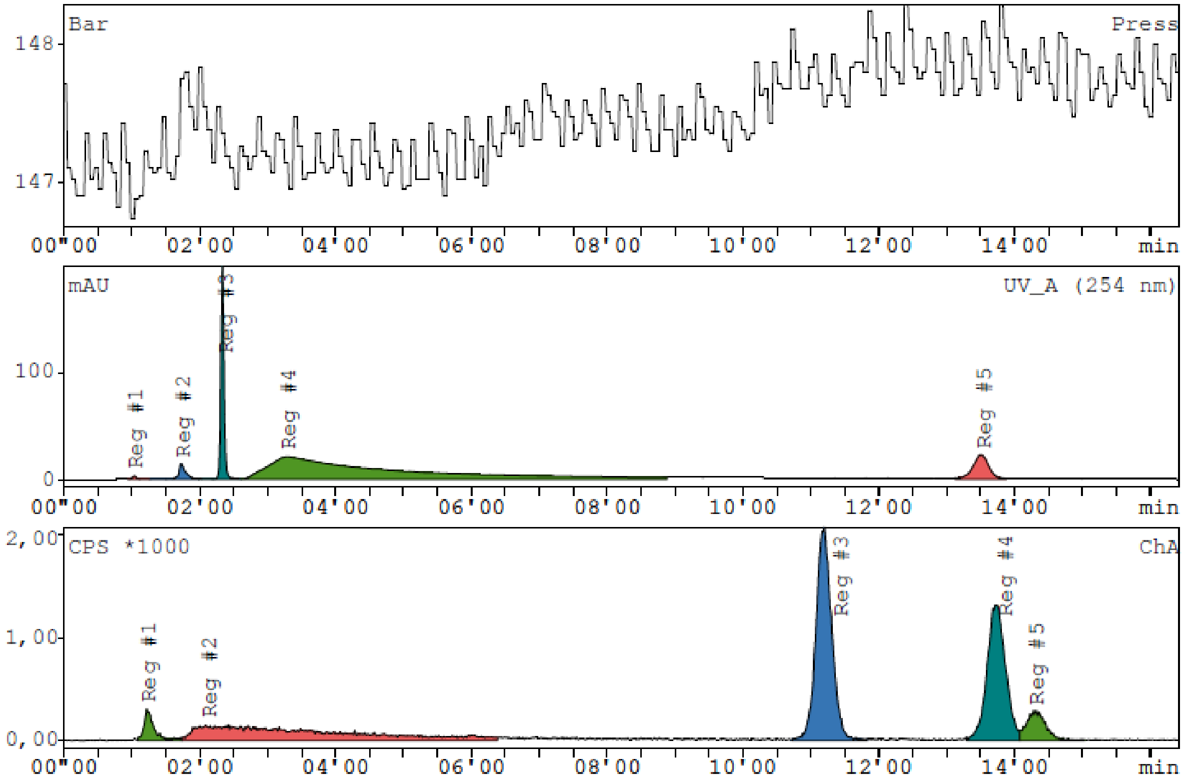

Sample description

Measurement:	181220-F, injection :	18.12.2020 13:22	
Method:	LUNAPFP Mudasir AcN_H2O 50_50 from:		17.12.2020 09:30
Actonitrile:H20 =50:50, Flow 1,5 mL/min, injection 10 micro L.			
Radio detector: raytest Gabi Star	Serial Nr.: #30745 raytest GINA star 20.04.09 Firmware V4.8		
Software Version: 5.9, Service Pack 8, Build 5076			

Integration ChA

Substance	R/T s	Type	Area Counts	%Area %
Reg #1	01'14	DD(M	3090,86	3,52
Reg #2	02'07	DD(M	20727,11	23,60
Reg #3	11'11	DD(M	33288,06	37,91
Reg #4	13'43	DD(M	24962,57	28,43
Reg #5	14'18	DD(M	5739,83	6,54
Sum in ROI			87808,43	100,00
Area (total)			94336,62	
Ext. BKG			0,00 CPS	

Figure S50: Analytical HPLC chromatogram from screening, top channel = UV, bottom channel = activity. HPLC spectrum of 2,2-difluoro-2-(fluoro-^{18}F)ethyl 4-methylbenzenesulfonate **([^{18}F]1b). 1a**, Et_3SiH, DMSO, 90°C, Ag (II), 6 min. 100 µL organic phase in 1 mL MeCN: H_2O =50:50. **(**MeCN: H_2O =50:50. Flow rate = 1.5 mL/min. Injected volume = 10 µL) (Table 1, entry 12).

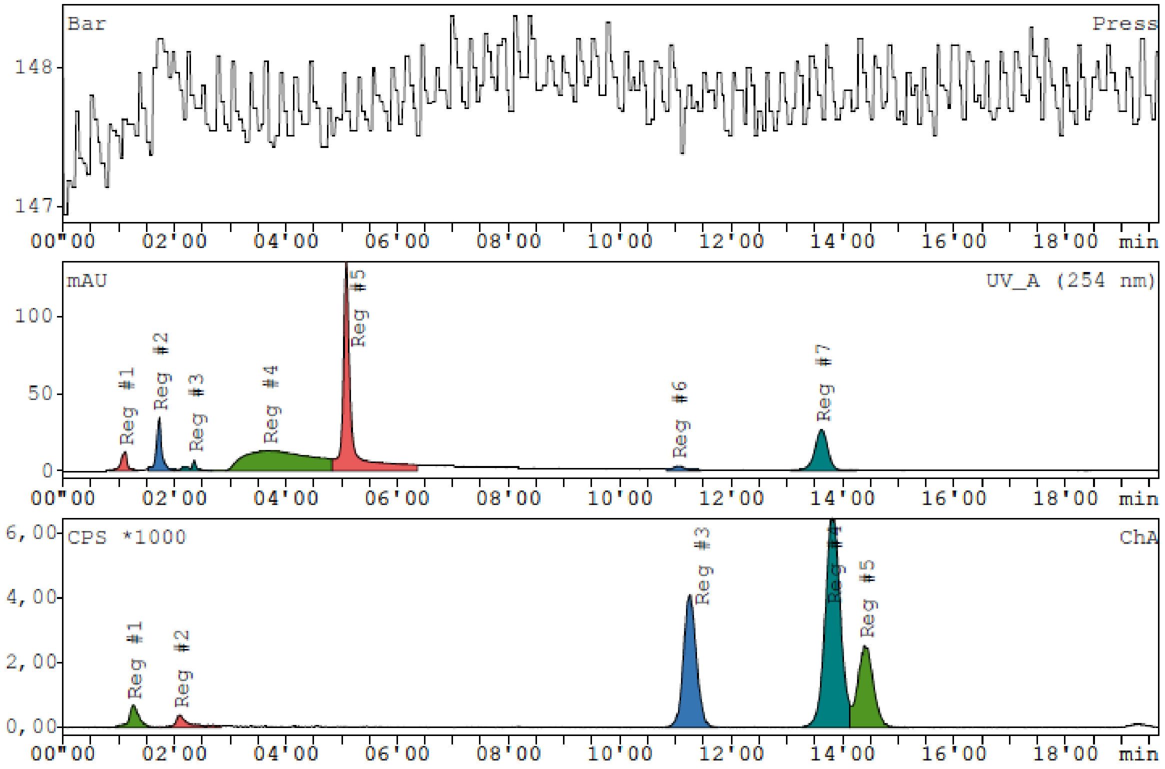

Sample description

Measurement:	181220-H, injection :	18.12.2020 14:00
Method:	LUNAPFP Mudasir AcN_H2O 50_50 from:	17.12.2020 09:30
Actonitrile:H20 =50:50, Flow 1,5 mL/min, injection 10 micro L.		
Radio detector: raytest Gabi Star	Serial Nr.: #30745 raytest GINA star 20.04.09 Firmware V4.8	
Software Version: 5.9, Service Pack 8, Build 5076		

Integration ChA

Substance	R/T s	Type	Area Counts	%Area %
Reg #1	01'16	DD(M	8878,0	3,50
Reg #2	02'06	DD(M	6900,1	2,72
Reg #3	11'15	DD(M	65466,3	25,81
Reg #4	13'49	DD(M	122068,5	48,13
Reg #5	14'25	DD(M	50298,9	19,83
Sum in ROI			253611,8	100,00
Area (total)			269428,6	
Ext. BKG			0,00 CPS	

Figure S51: Analytical HPLC chromatogram from screening, top channel = UV, bottom channel = activity. HPLC spectrum of 2,2-difluoro-2-(fluoro-^{18}F)ethyl 4-methylbenzenesulfonate **([^{18}F]1b). 1a**, TEMPO, Et_3SiH, DMSO, 90°C, Ag (II), 6 min. 100 µL organic phase in 1 mL MeCN: H_2O =50:50. **(**MeCN: H_2O =50:50. Flow rate = 1.5 mL/min. Injected volume = 10 µL) (Table 1, entry 13).

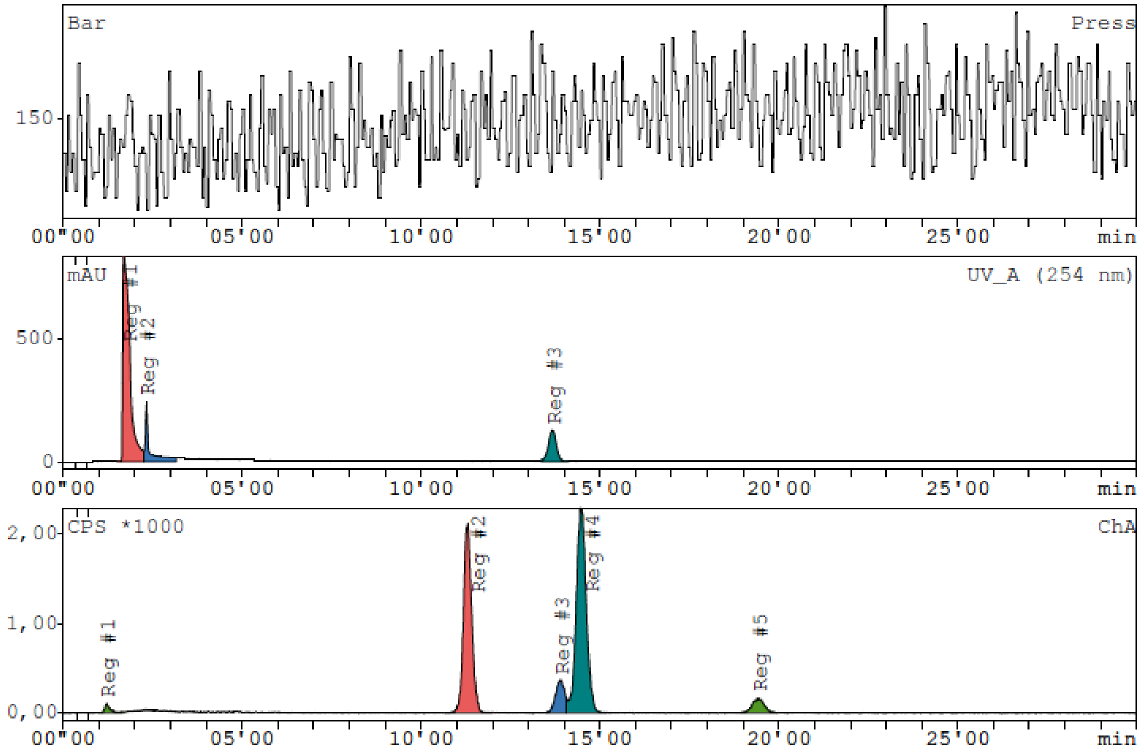

Sample description

Measurement:	070121-F, injection :	07.01.2021 15:40
Method:	LUNAPFP Mudasir AcN_H2O 50_50 from:	17.12.2020 09:30
MeCN: H2O=50:50. Flow rate = 1,5 mL/min. Injected volume = 10 uL.		
Radio detector: raytest Gabi Star	Serial Nr.: #30745 raytest GINA star 20.04.09 Firmware V4.8	
Software Version: 5.9, Service Pack 8, Build 5076		

Integration ChA

Substance	R/T s	Type	Area Counts	%Area %
Reg #1	01'13	DD(M	1083,61	1,23
Reg #2	11'18	DD(M	32807,95	37,30
Reg #3	13'54	DD(M	6042,22	6,87
Reg #4	14'29	DD(M	44270,90	50,34
Reg #5	19'27	DD(M	3747,20	4,26
Sum in ROI			87951,88	100,00
Area (total)			92497,04	
BKG1			2,500	
Remainder			4545,16	4,91

Figure S52: Analytical HPLC chromatogram from screening, top channel = UV, bottom channel = activity. HPLC spectrum of 2,2-difluoro-2-(fluoro-^{18}F)ethyl 4-methylbenzenesulfonate **([^{18}F]1b). 1a**, Et_3SiH, DMSO, 90°C, 2-propanol, Ag (II), 6 min. 100 µL organic phase in 1 mL MeCN: H_2O =50:50. **(**MeCN: H_2O =50:50. Flow rate = 1.5 mL/min. Injected volume = 10 µL) (Table 1, entry 14).

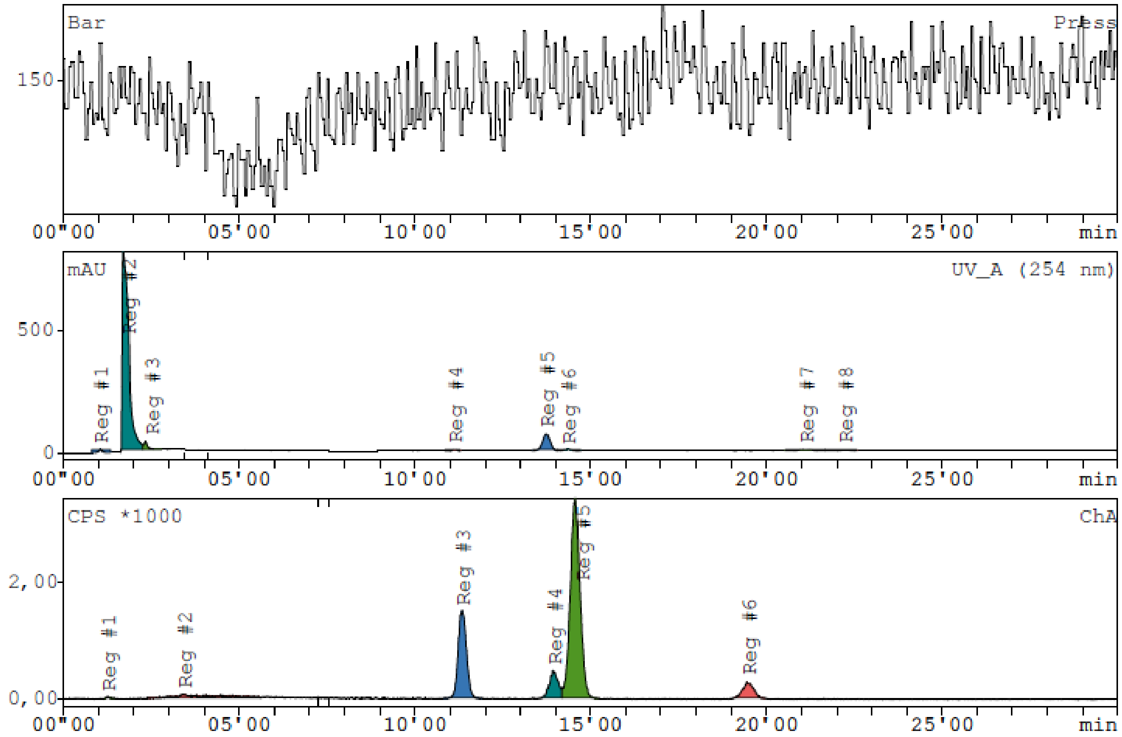

Sample description

Measurement:	080121-C, injection :	08.01.2021 12:54
Method:	LUNAPFP Mudasir AcN_H2O 50_50 from:	17.12.2020 09:30
MeCN:H2O =50:50. Flow rate = 1,5 mL/min. Injected volume = 10 uL.		
Radio detector: raytest Gabi Star	Serial Nr.: #30745 raytest GINA star 20.04.09 Firmware V4.8	
Software Version: 5.9, Service Pack 8, Build 5076		

Integration ChA

Substance	R/T s	Type	Area Counts	%Area %
Reg #1	01'15	DD(M	-20,35	-0,02
Reg #2	03'25	DD(M	4602,58	4,33
Reg #3	11'20	DD(M	23434,65	22,06
Reg #4	13'57	DD(M	7691,27	7,24
Reg #5	14'33	DD(M	65105,27	61,30
Reg #6	19'29	DD(M	5398,03	5,08
Sum in ROI			106211,46	100,00
Area (total)			85901,06	
BKG1			26,551	
Remainder			-20310,40	-23,64

Figure S53: Analytical HPLC chromatogram from screening, top channel = UV, bottom channel = activity. HPLC spectrum of 2,2-difluoro-2-(fluoro-^{18}F)ethyl 4-methylbenzenesulfonate **([^{18}F]1b). 1a**, TEMPO, Et_3SiH, DMSO, 90°C, 2-propanol, Ag (II), 6 min. 100 µL organic phase in 1 mL MeCN: H_2O =50:50. **(**MeCN: H_2O =50:50. Flow rate = 1.5 mL/min. Injected volume = 10 µL) (Table 1, entry 15).

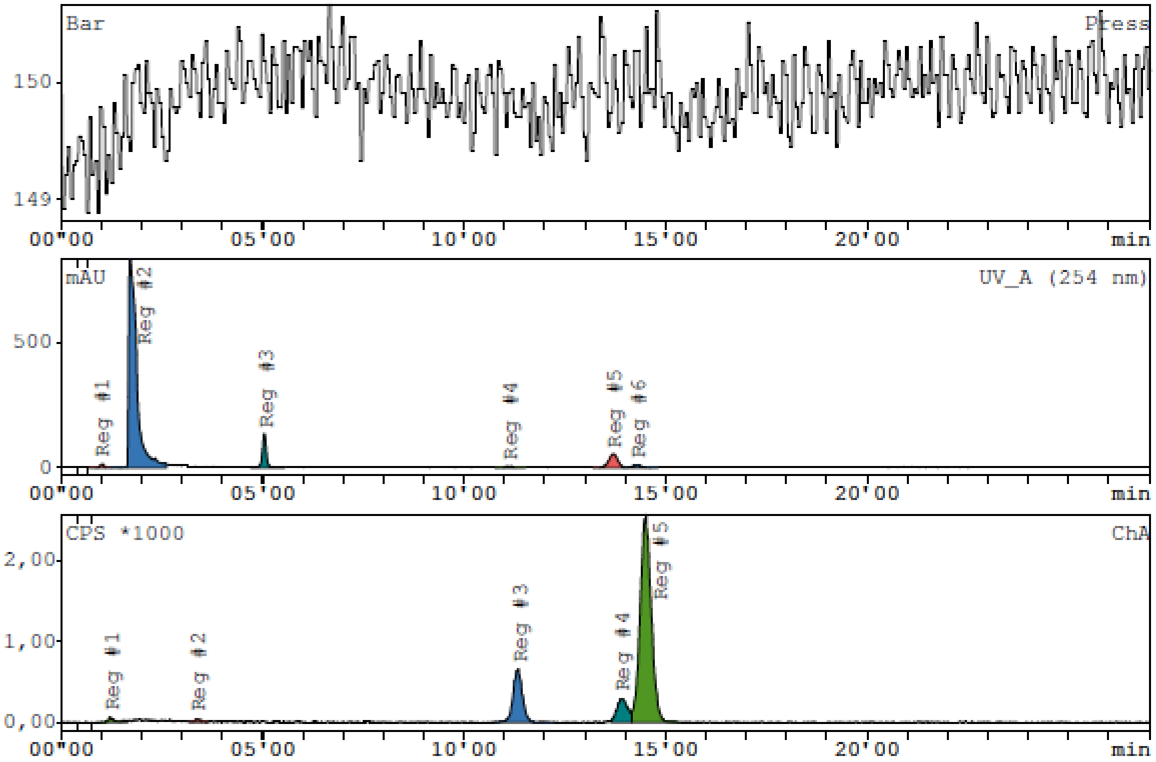

Sample description

Measurement:	070121-E, injection : 07.01.2021 15:12
Method:	LUNAPFP Mudasir AcN_H2O 50_50 from: 17.12.2020 09:30
MeCN: H2O=50:50. Flow rate = 1,5 mL/min. Injected volume = 10 uL.	
Radio detector: raytest Gabi Star	Serial Nr.: #30745 raytest GINA star 20.04.09 Firmware V4.8
Software Version: 5.9, Service Pack 8, Build 5076	

Integration ChA

Substance	R/T s	Type	Area Counts	%Area %
Reg #1	01'13	DD(M	743,31	1,14
Reg #2	03'23	DD(M	512,39	0,79
Reg #3	11'20	DD(M	10278,42	15,80
Reg #4	13'55	DD(M	5348,08	8,22
Reg #5	14'30	DD(M	48161,14	74,04
Sum in ROI			65043,34	100,00
Area (total)			68986,94	
BKG1			2,737	
Remainder			3943,60	5,72

Figure S54. Analytical HPLC chromatogram from screening, top channel = UV, bottom channel = activity. HPLC spectrum of 2,2-difluoro-2-(fluoro-^{18}F)ethyl 4-methylbenzenesulfonate **([^{18}F]1b). 1a**, TEMPO, Et_3SiH, DMSO, 90°C, 2-propanol , 6 min. 100 µL organic phase in 1 mL MeCN: H_2O =50:50. **(**MeCN: H_2O =50:50. Flow rate = 1.5 mL/min. Injected volume = 10 µL). (*Table 1, entry 16*).

Measurement 090621-H Uni Oslo Page 1/1
c:\GINA_NT\LUNAPFP Mudasir AcN_H2O 50_50\090621-H

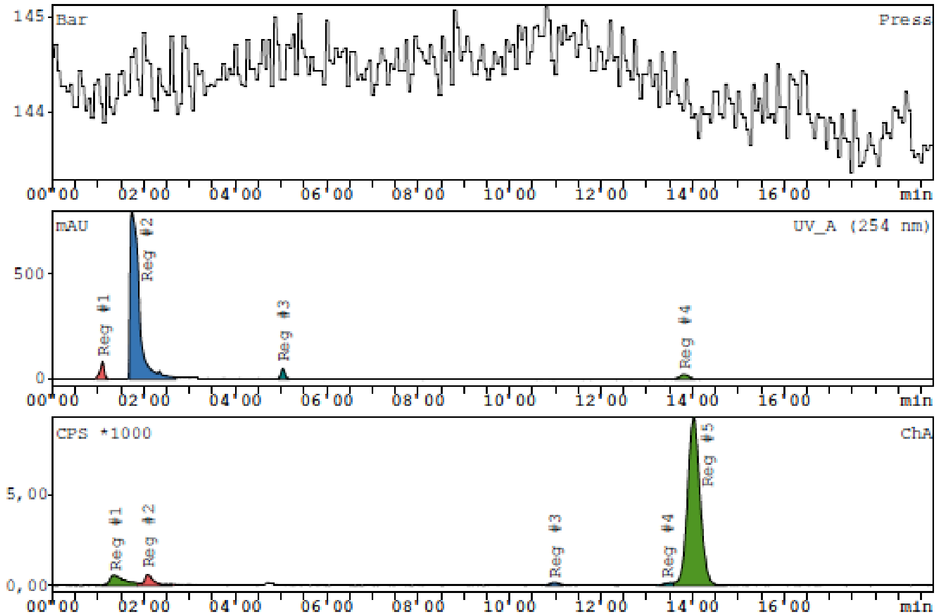

Sample description

Measurement:	090621-H, injection : 09.06.2021 14:11
Method:	LUNAPFP Mudasir AcN_H2O 50_50 from: 17.12.2020 09:30
CH3CN:H2O=50:50, Flow rate 1,5 mL/min, injection 10 micro L	
Radio detector: raytest Gabi Star	Serial Nr.: #30745 raytest GINA star 20.04.09 Firmware V4.8
Software Version: 5.9, Service Pack 8, Build 5076	

Integration ChA

Substance	R/T s	Type	Area Counts	%Area %
Reg #1	01'21	DD(M	12934,9	6,52
Reg #2	02'06	DD(M	9178,2	4,63
Reg #3	10'58	DD(M	2313,5	1,17
Reg #4	13'25	DD(M	1913,4	0,97
Reg #5	14'01	DD(M	171904,7	86,71
Sum in ROI			198244,6	100,00
Area (total)			210831,3	
Ext. BKG			0,00 CPS	

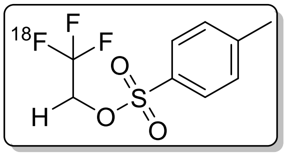

Integration UV_A (254 nm)

Substance	R/T s	Type	Area mAU*s	%Area %
Reg #1	01'05	DD(M	508,07	4,20
Reg #2	01'45	DD(M	10971,51	90,62
Reg #3	05'03	DD(M	316,22	2,61
Reg #4	13'49	DD(M	311,95	2,58
Sum in ROI			12107,75	100,00

Figure S55: Analytical HPLC chromatogram from screening, top channel = UV, bottom channel = activity. HPLC spectrum of 2,2-difluoro-2-(fluoro-^{18}F)ethyl 4-methylbenzenesulfonate **([^{18}F]1b).** (**1a**), TEMPO, Et_3SiH, DMSO, 90̊C, 2-methyl-2-butanol, 6 min. 100 µL organic phase in 1 mL MeCN: H2O=50:50. **(**MeCN: H2O=50:50. Flow rate = 1.5 mL/min. Injected volume = 10 µL). (*Table 1, entry 17*).

Measurement 310821-I Uni Oslo Page 1/2
c:\GINA_NT\LUNAPFP Mudasir AcN_H2O 50_50\310821-I

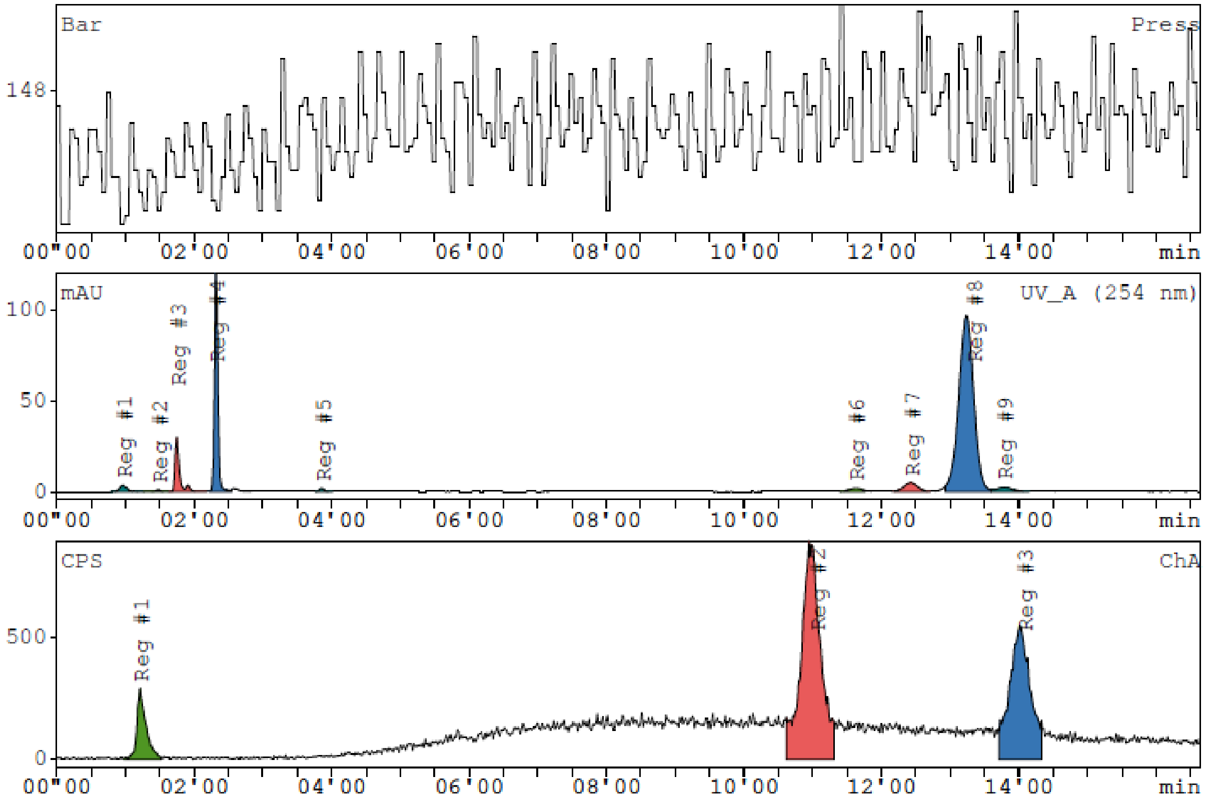

Measurement:	310821-I, injection :	31.08.2021 13:41
Method:	LUNAPFP Mudasir AcN_H2O 50_50 from:	17.12.2020 09:30
CH3CN:H2O=50:50, Flow rate 1,5 mL/min, injection 10 micro L		
Radio detector: raytest Gabi Star	Serial Nr.: #30745 raytest GINA star 20.04.09 Firmware V4.8	
Software Version: 5.9, Service Pack 8, Build 5076		

ChA

**Substance**	**R/T s**	**Type**	**Area Counts**	**%Area %**
Reg #1	01'13	DD(M	2642,74	8,36
Reg #2	10'59	DD(M	17502,56	55,40
Reg #3	14'01	DD(M	11448,01	36,24
Sum in ROI			31593,30	100,00
Area (total)			107802,62	
Ext. BKG			0,00 CPS	

Figure S56: Analytical HPLC chromatogram from screening, top channel = UV, bottom channel = activity. HPLC spectrum of 2,2-difluoro-2-(fluoro-^{18}F)ethyl 4-methylbenzenesulfonate **([^{18}F]1b). 1a**, TEMPO, Et_3SiH, without DMSO, 90̊ C, 2-methyl-2-butanol (1 mL), 6 min. 100 µL organic phase in 1 mL MeCN: H_2O =50:50. **(**MeCN: H_2O =50:50. Flow rate = 1.5 mL/min. Injected volume = 10 µL) (Table 1, entry 18).

Measurement 161212_controlMe Uni Oslo Page 1/2
c:\GINA_NT\18F-Lansoprazole\161212_controlMe

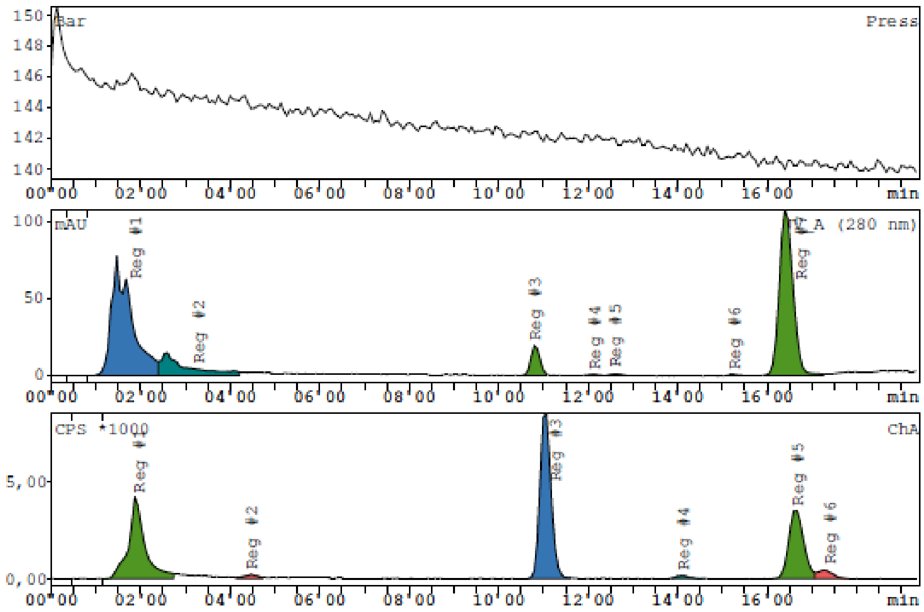

Measurement:	161212_controlMe, injection :	12.12.2016 16:27
Method:	18F-LANSOPRAZOLE from:	12.12.2016 11:45

Mobile Phase: AcN:Water:30:70
Flow rate: 2 mL/mim
PFP (2) 150 x 4.6 mm
Method changed: 09/12/2016
Mobile Phase: AcN:Water:60:40
Flow rate: 1,5 mL/mim
PFP (2) 250 x 4.6 mm
Column PFP(2) 150*4.6mm (Used (250*4.6mm) 161209)
Flow rate: 2mL/min
Wavelenght: 280
Radio detector: raytest Gabi Star Serial Nr.: #30745 raytest GINA star 20.04.09 Firmware V4.8
Software Version: 5.9, Service Pack 8, Build 5076

ChA

Substance	R/T s	Type	Area Counts	%Area %
Reg #1	01'56	DD(M	103055,7	29,85
Reg #2	04'28	DD(M	4393,2	1,27
Reg #3	11'04	DD(M	145204,8	42,05
Reg #4	14'08	DD(M	2703,3	0,78
Reg #5	16'40	DD(M	79629,1	23,06
Reg #6	17'24	DD(M	10312,3	2,99
Sum in ROI			345298,3	100,00
Area (total)			364543,3	
BKG1			47,40	
Remainder			19244,99	5,28

Figure S57: *Analytical HPLC chromatogram from screening, top channel = UV, bottom channel = activity. HPLC spectrum of 1,1-difluoro-1-(fluoro-*[18]*F)propan-2-yl benzenesulfonate* ***(***[^{18}F]***2b). (2a****), DMSO, 85°C, air, 5 min. 100 µL organic phase in 1 mL MeCN: H_2O=50:50.* ***(****MeCN: H_2O =30:70. Flow rate = 2.0 mL/min. Injected volume = 10 µL). (Table* S2, *entry 1).*

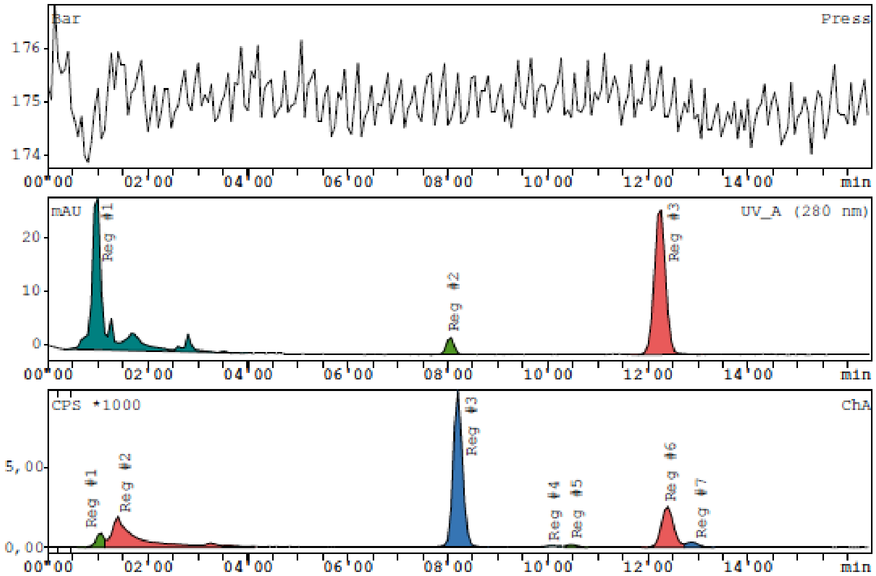

Measurement: 161223_rxn3, injection : 23.12.2016 12:40
Method: 18F-Lansoprazole_50 from: 23.12.2016 11:24
Mobile Phase: AcN:Water:30:70
Flow rate: 2 mL/mim
PFP (2) 150 x 4.6 mm
Method changed: 09/12/2016
Mobile Phase: AcN:Water:60:40
Flow rate: 1,5 mL/mim
PFP (2) 250 x 4.6 mm
Method changed: 12/12/2016
Mobile Phase: AcN:Water:50:50
Flow rate: 1,5 mL/mim
PFP (2) 250 x 4.6 mm
Column PFP(2) 150*4.6mm (Used (250*4.6mm) 161209)
Flow rate: 2mL/min
Wavelenght: 280
Radio detector: raytest Gabi Star Serial Nr.: #30745 raytest GINA star 20.04.09 Firmware V4.8
Software Version: 5.9, Service Pack 8, Build 5076

ChA

Substance	R/T s	Type	Area Counts	%Area %
Reg #1	48"00	BD(M	7836,5	3,15
Reg #2	01'28	DD(M	66882,9	26,91
Reg #3	08'12	BB(M	122736,3	49,38
Reg #4	10'04	BB(M	44,5	0,02
Reg #5	10'32	DD(M	2556,8	1,03
Reg #6	12'24	DD(M	42722,4	17,19
Reg #7	13'00	DD(M	5799,3	2,33

Figure 58: *Analytical HPLC chromatogram from screening, top channel = UV, bottom channel = activity. HPLC spectrum of 2,2-difluoro-2-(fluoro-^{18}F)ethyl 4-methylbenzenesulfonate* ***([^{18}F]1b).*** *(**1a**), DMSO, 85°C, AgNO$_3$, N$_2$, 5 min. 100 µL organic phase in 1 mL MeCN: H$_2$O=50:50.* ***(****MeCN: H$_2$O =30:70. Flow rate = 2.0 mL/min. Injected volume = 10 µL). (Table S5, entry 34).*

Measurement 161212_control Uni Oslo Page 1/2
c:\GINA_NT\18F-Lansoprazole\161212_control

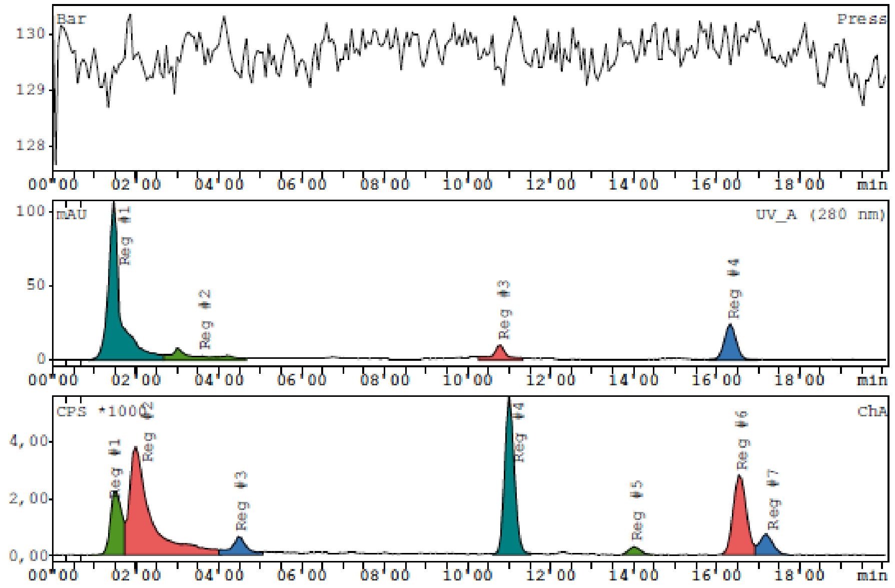

Measurement:	161212_control, injection :	12.12.2016 13:30
Method:	18F-LANSOPRAZOLE from:	12.12.2016 11:45

Mobile Phase: AcN:Water:30:70
Flow rate: 2 mL/mim
PFP (2) 150 x 4.6 mm
Method changed: 09/12/2016
Mobile Phase: AcN:Water:60:40
Flow rate: 1,5 mL/mim
PFP (2) 250 x 4.6 mm
Column PFP(2) 150*4.6mm (Used (250*4.6mm) 161209)
Flow rate: 2mL/min
Wavelenght: 280
Radio detector: raytest Gabi Star Serial Nr.: #30745 raytest GINA star 20.04.09 Firmware V4.8
Software Version: 5.9, Service Pack 8, Build 5076

ChA

Substance	R/T s	Type	Area Counts	%Area %
Reg #1	01'32	DD(M	39432,1	10,76
Reg #2	02'00	DD(M	137463,1	37,52
Reg #3	04'28	DD(M	16807,0	4,59
Reg #4	11'00	DD(M	89326,5	24,38
Reg #5	14'00	DD(M	5609,2	1,53
Reg #6	16'32	DD(M	60936,5	16,63
Reg #7	17'16	DD(M	16813,2	4,59
Sum in ROI			366387,7	100,00
Area (total)			390190,6	
BKG1			32,30	
Remainder			23802,90	6,10

Figure S59: Analytical HPLC chromatogram from screening, top channel = UV, bottom channel = activity. HPLC spectrum of 1,1-difluoro-1-(fluoro-^{18}F)propan-2-yl benzenesulfonate **(**[^{18}F]**2b). (2a**), DMSO, 85°C, N_2, 5 min. 100 µL organic phase in 1 mL MeCN: H_2O=50:50. **(**MeCN: H_2O =30:70. Flow rate = 2.0 mL/min. Injected volume = 10 µL). (Table S6, entry 2).

Measurement 161223_rxn14 Uni Oslo Page 1/2
c:\GINA_NT\18F-Lansoprazole_50\161223_rxn14

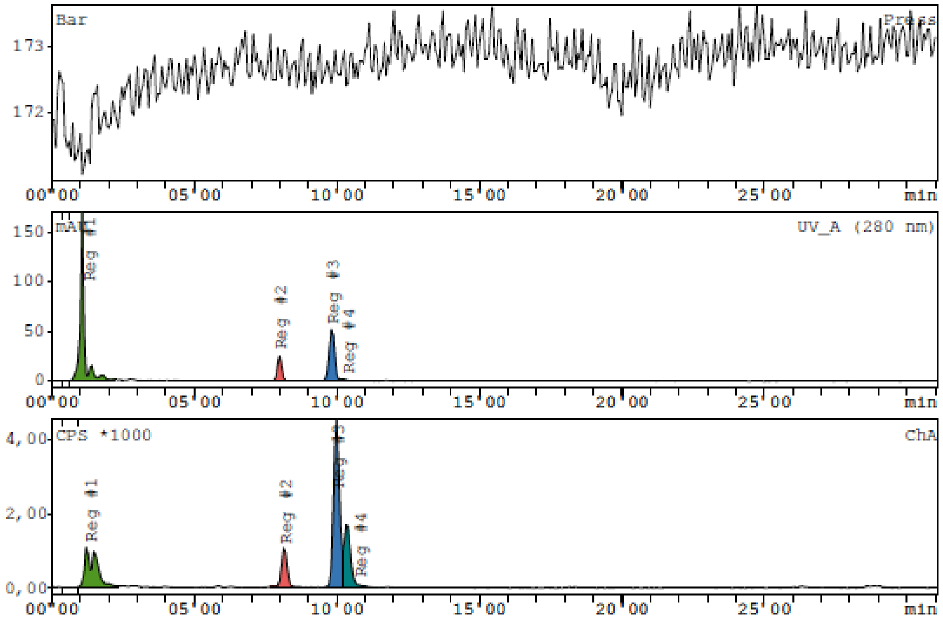

Measurement: 161223_rxn14, injection : 23.12.2016 15:56
Method: 18F-Lansoprazole_50 from: 23.12.2016 14:15
Mobile Phase: AcN:Water:30:70
Flow rate: 2 mL/mim
PFP (2) 150 x 4.6 mm
Method changed: 09/12/2016
Mobile Phase: AcN:Water:60:40
Flow rate: 1,5 mL/mim
PFP (2) 250 x 4.6 mm
Method changed: 12/12/2016
Mobile Phase: AcN:Water:50:50
Flow rate: 1,5 mL/mim
PFP (2) 250 x 4.6 mm
Column PFP(2) 150*4.6mm (Used (250*4.6mm) 161209)
Flow rate: 2mL/min
Wavelenght: 280
Radio detector: raytest Gabi Star Serial Nr.: #30745 raytest GINA star 20.04.09 Firmware V4.8
Software Version: 5.9, Service Pack 8, Build 5076

ChA

Substance	R/T s	Type	Area Counts	%Area %
Reg #1	01'20	DD(M	29859,02	21,33
Reg #2	08'08	DD(M	15158,83	10,83
Reg #3	10'00	DD(M	63938,45	45,67
Reg #4	10'48	DD(M	31044,61	22,17
Sum in ROI			140000,91	100,00
Area (total)			143879,42	
BKG1			12,917	

Figure S60: Analytical HPLC chromatogram from screening, top channel = UV, bottom channel = activity. HPLC spectrum of 1,1-difluoro-1-(fluoro-^{18}F)propan-2-yl benzenesulfonate (*[^{18}F]***2b).** (**2a**), DMSO, 85°C, N_2, i-PrOH, 5 min. 100 µL organic phase in 1 mL MeCN: H_2O=50:50. **(**MeCN: H_2O =30:70. Flow rate = 2.0 mL/min. Injected volume = 10 µL). (Table *S6*, entry 3).

Measurement CF3CH2WAg Uni Oslo Page 1/2
c:\GINA_NT\18F-Lansoprazole\CF3CH2WAg

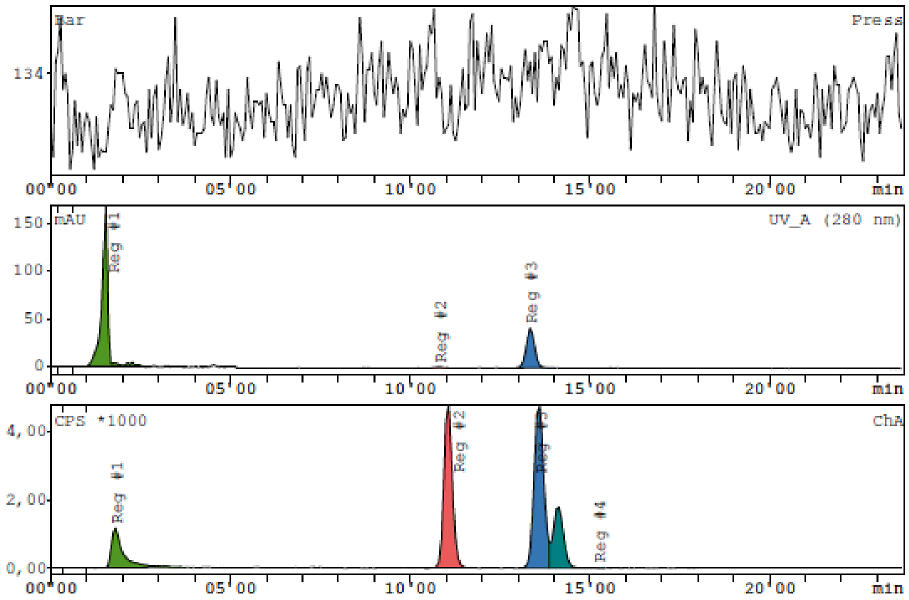

Measurement:	CF3CH2WAg, injection :	16.12.2016 16:22
Method:	18F-LANSOPRAZOLE from:	16.12.2016 15:49

Mobile Phase: AcN:Water:30:70
Flow rate: 2 mL/mim
PFP (2) 150 x 4.6 mm
Method changed: 09/12/2016
Mobile Phase: AcN:Water:60:40
Flow rate: 1,5 mL/mim
PFP (2) 250 x 4.6 mm
Method changed: 12/12/2016
Mobile Phase: AcN:Water:50:50
Flow rate: 1,5 mL/mim
PFP (2) 250 x 4.6 mm
Column PFP(2) 150*4.6mm (Used (250*4.6mm) 161209)
Flow rate: 2mL/min
Wavelenght: 280
Radio detector: raytest Gabi Star Serial Nr.: #30745 raytest GINA star 20.04.09 Firmware V4.8
Software Version: 5.9, Service Pack 8, Build 5076

ChA

Substance	R/T s	Type	Area Counts	%Area %
Reg #1	01'48	BB(M	25904,89	11,35
Reg #2	11'04	BB(M	76823,27	33,67
Reg #3	13'36	DD(M	87249,26	38,24
Reg #4	15'16	DD(M	38169,08	16,73
Sum in ROI			228146,51	100,00
Area (total)			236382,47	
BKG1			4,650	

Figure S61: Analytical HPLC chromatogram from screening, top channel = UV, bottom channel = activity. HPLC spectrum of 1,1-difluoro-1-(fluoro-^{18}F)propan-2-yl benzenesulfonate **([^{18}F]2b).** (**2a**), DMSO, 85°C, N_2, $AgNO_3$, 5 min. 100 µL organic phase in 1 mL MeCN: H_2O=50:50. **(**MeCN: H_2O =30:70. Flow rate = 2.0 mL/min. Injected volume = 10 µL). (Table S6, entry 4).

Measurement 161209_CuCN_1 Uni Oslo Page 1/2

c:\GINA_NT\18F-Lansoprazole\161209_CuCN_1

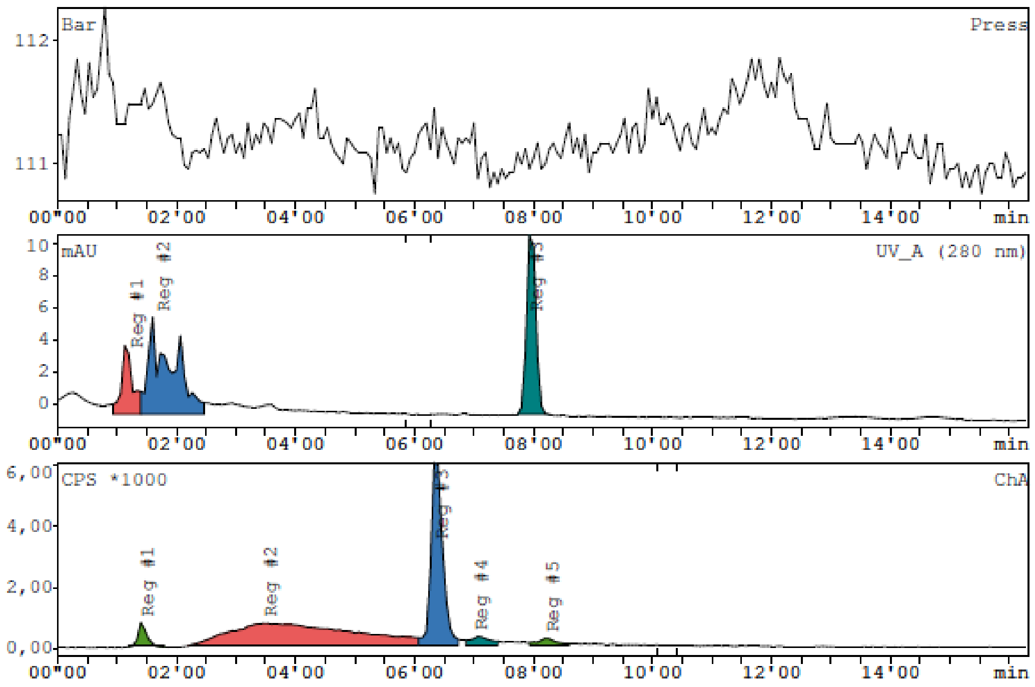

Measurement: 161209_CuCN_1, injection :	09.12.2016 14:14
Method: 18F-LANSOPRAZOLE from:	09.12.2016 13:31
Mobile Phase: AcN:Water:30:70	
Column PFP(2) 150*4.6mm (Used (250*4.6mm) 161209)	
Flow rate: 2mL/min	
Wavelenght: 280	
Radio detector: raytest Gabi Star Serial Nr.: #30745 raytest GINA star 20.04.09 Firmware V4.8	
Software Version: 5.9, Service Pack 8, Build 5076	

ChA

Substance	R/T s	Type	Area Counts	%Area %
Reg #1	01'28	DD(M	5878,1	2,92
Reg #2	03'32	DD(M	101788,7	50,56
Reg #3	06'24	DD(M	82815,7	41,14
Reg #4	07'04	DD(M	6203,3	3,08
Reg #5	08'16	DD(M	4628,8	2,30
Sum in ROI			201314,6	100,00
Area (total)			185780,9	
BKG1			110,62	
Remainder			-15533,64	-8,36

Figure S62: Analytical HPLC chromatogram from screening, top channel = UV, bottom channel = activity. HPLC spectrum of 1,1-difluoro-1-(fluoro-^{18}F)propan-2-yl benzenesulfonate **([^{18}F]2b).** (**2a**), DMSO, 85°C, CuCN, air, 5 min. 100 µL organic phase in 1 mL MeCN: H_2O=50:50. **(**MeCN: H_2O =30:70. Flow rate = 2.0 mL/min. Injected volume = 10 µL). (Table S6, entry 5).

Measurement 161213_dmsotest_ipropoh Uni Oslo Page 1/2
c:\GINA_NT\18F-Lansoprazole\161213_dmsotest_ipropoh

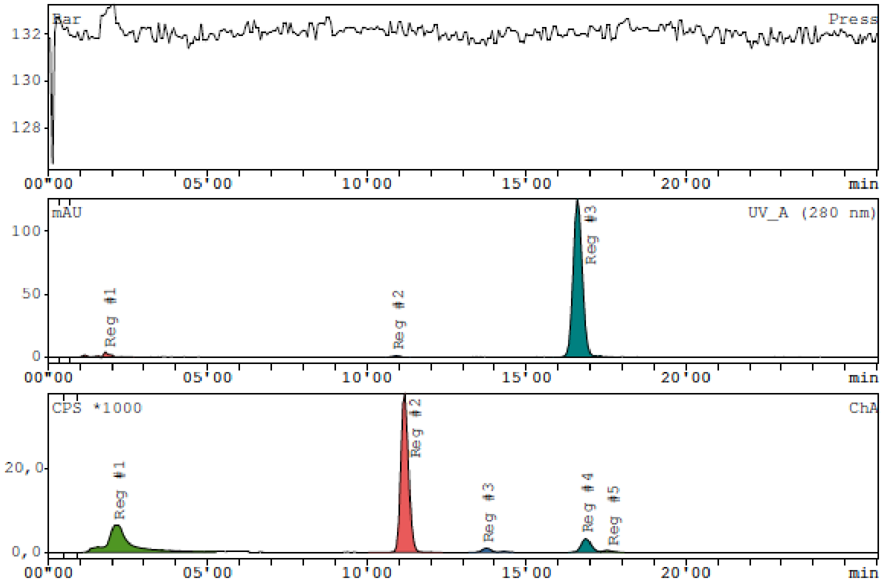

Measurement: 161213_dmsotest_ipropoh, injection : 13.12.2016 13:46
Method: 18F-LANSOPRAZOLE from: 13.12.2016 08:33
Mobile Phase: AcN:Water:30:70
Flow rate: 2 mL/mim
PFP (2) 150 x 4.6 mm
Method changed: 09/12/2016
Mobile Phase: AcN:Water:60:40
Flow rate: 1,5 mL/mim
PFP (2) 250 x 4.6 mm
Method changed: 12/12/2016
Mobile Phase: AcN:Water:50:50
Flow rate: 1,5 mL/mim
PFP (2) 250 x 4.6 mm
Column PFP(2) 150*4.6mm (Used (250*4.6mm) 161209)
Flow rate: 2mL/min
Wavelenght: 280
Radio detector: raytest Gabi Star Serial Nr.: #30745 raytest GINA star 20.04.09 Firmware V4.8
Software Version: 5.9, Service Pack 8, Build 5076

ChA

Substance	R/T s	Type	Area Counts	%Area %
Reg #1	02'08	DD(M	320006,0	30,44
Reg #2	11'12	DD(M	626377,6	59,59
Reg #3	13'44	DD(M	24179,7	2,30
Reg #4	16'52	DD(M	70051,2	6,66
Reg #5	17'40	DD(M	10518,6	1,00
Sum in ROI			1051133,3	100,00
Area (total)			1089392,8	

Figure S63: Analytical HPLC chromatogram from screening, top channel = UV, bottom channel = activity. HPLC spectrum of 1,1-difluoro-1-(fluoro-^{18}F)propan-2-yl benzenesulfonate **([^{18}F]2b).** (**2a**), DMSO, 85°C, CuOAC, i-PrOH, air, 5 min. 100 µL organic phase in 1 mL MeCN: H_2O=50:50. **(**MeCN: H_2O =30:70. Flow rate = 2.0 mL/min. Injected volume = 10 µL). (Table S6, entry 6).

Measurement 161212Cu03 Uni Oslo Page 1/2
c:\GINA_NT\18F-Lansoprazole\161212Cu03

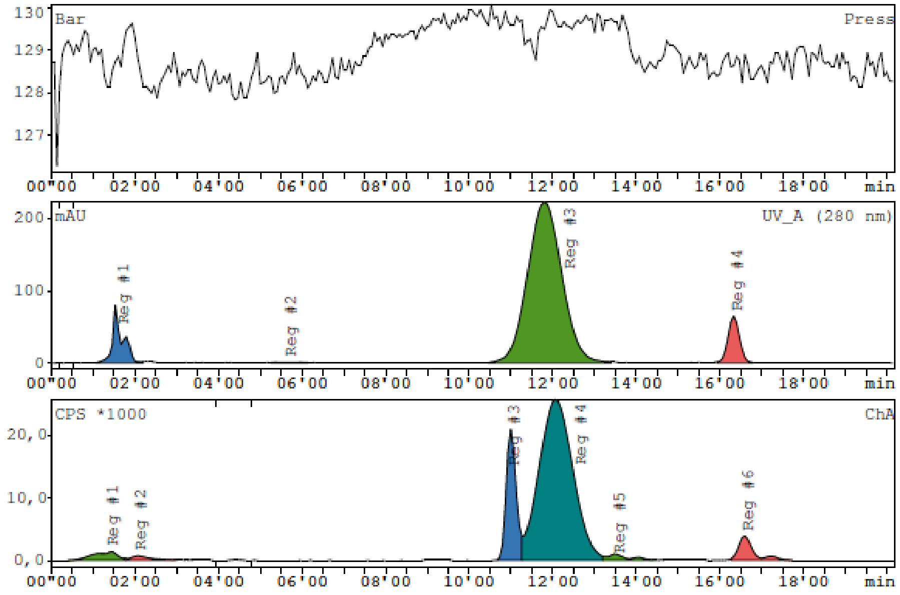

Measurement:	161212Cu03, injection :	12.12.2016 12:41
Method:	18F-LANSOPRAZOLE from:	12.12.2016 11:45

Mobile Phase: AcN:Water:30:70
Flow rate: 2 mL/mim
PFP (2) 150 x 4.6 mm
Method changed: 09/12/2016
Mobile Phase: AcN:Water:60:40
Flow rate: 1,5 mL/mim
PFP (2) 250 x 4.6 mm
Column PFP(2) 150*4.6mm (Used (250*4.6mm) 161209)
Flow rate: 2mL/min
Wavelenght: 280
Radio detector: raytest Gabi Star Serial Nr.: #30745 raytest GINA star 20.04.09 Firmware V4.8
Software Version: 5.9, Service Pack 8, Build 5076

ChA

Substance	R/T s	Type	Area Counts	%Area %
Reg #1	01'24	DD(M	50874	2,59
Reg #2	02'04	DD(M	19951	1,01
Reg #3	11'00	DD(M	326151	16,58
Reg #4	12'04	DD(M	1446011	73,49
Reg #5	13'48	DD(M	31744	1,61
Reg #6	16'36	DD(M	92964	4,72
Sum in ROI			1967695	100,00
Area (total)			1943176	
BKG1			101,3	
Remainder			-24519,45	-1,26

Figure S64: Analytical HPLC chromatogram from screening, top channel = UV, bottom channel = activity. HPLC spectrum of 1,1-difluoro-1-(fluoro-^{18}F)propan-2-yl benzenesulfonate **([^{18}F]2b).** (**2a**), DMSO, 85°C, CuOAC, air, 5 min. 100 µL organic phase in 1 mL MeCN: H_2O=50:50. **(**MeCN: H_2O =30:70. Flow rate = 2.0 mL/min. Injected volume = 10 µL). (Table S6, entry 7).

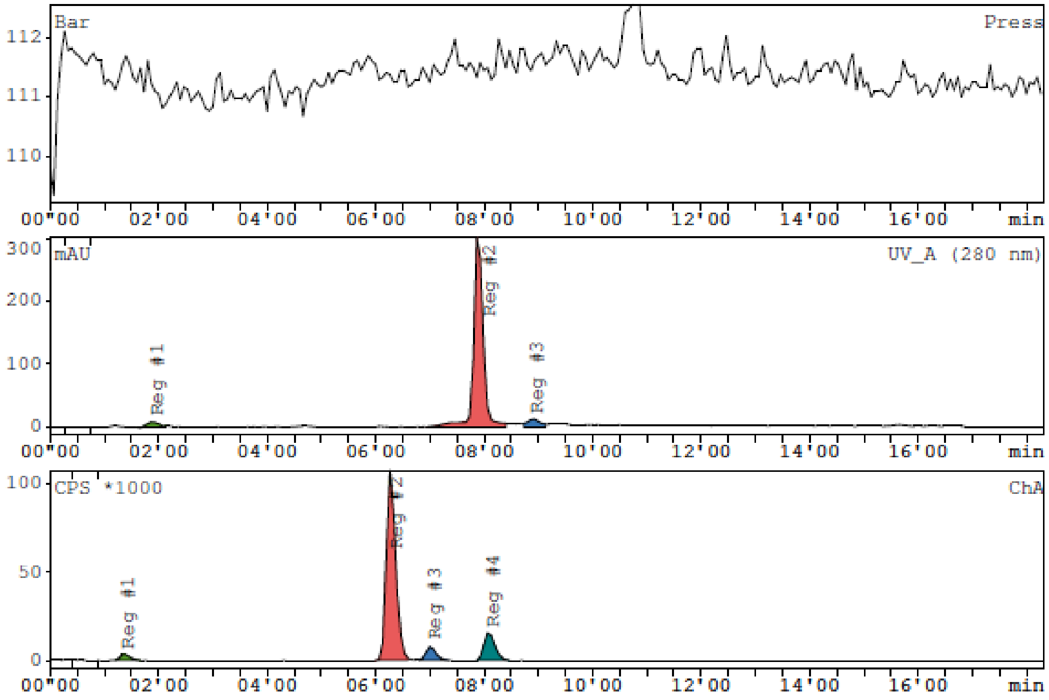

Measurement: 161209_2_CuOAc, injection :	09.12.2016 13:54
Method: 18F-LANSOPRAZOLE from:	09.12.2016 13:31
Mobile Phase: AcN:Water:30:70	
Column PFP(2) 150*4.6mm (Used (250*4.6mm) 161209)	
Flow rate: 2mL/min	
Wavelenght: 280	
Radio detector: raytest Gabi Star Serial Nr.: #30745 raytest GINA star 20.04.09 Firmware V4.8	
Software Version: 5.9, Service Pack 8, Build 5076	

ChA

Substance	R/T s	Type	Area Counts	%Area %
Reg #1	01'24	DD(M	37996	2,38
Reg #2	06'20	DD(M	1253235	78,64
Reg #3	07'04	DD(M	92786	5,82
Reg #4	08'08	DD(M	209548	13,15
Sum in ROI			1593566	100,00
Area (total)			1353115	
BKG1			377,8	
Remainder			-240450,59	-17,77

Figure S65: Analytical HPLC chromatogram from screening, top channel = UV, bottom channel = activity. HPLC spectrum of 1,1-difluoro-1-(fluoro-^{18}F)propan-2-yl benzenesulfonate **([^{18}F]2b).** (**2a**), DMSO, 85°C, Cu(OAC)$_2$, air, 5 min. 100 µL organic phase in 1 mL MeCN: H_2O=50:50. **(**MeCN: H_2O =30:70. Flow rate = 2.0 mL/min. Injected volume = 10 µL). (Table S6, entry 8).

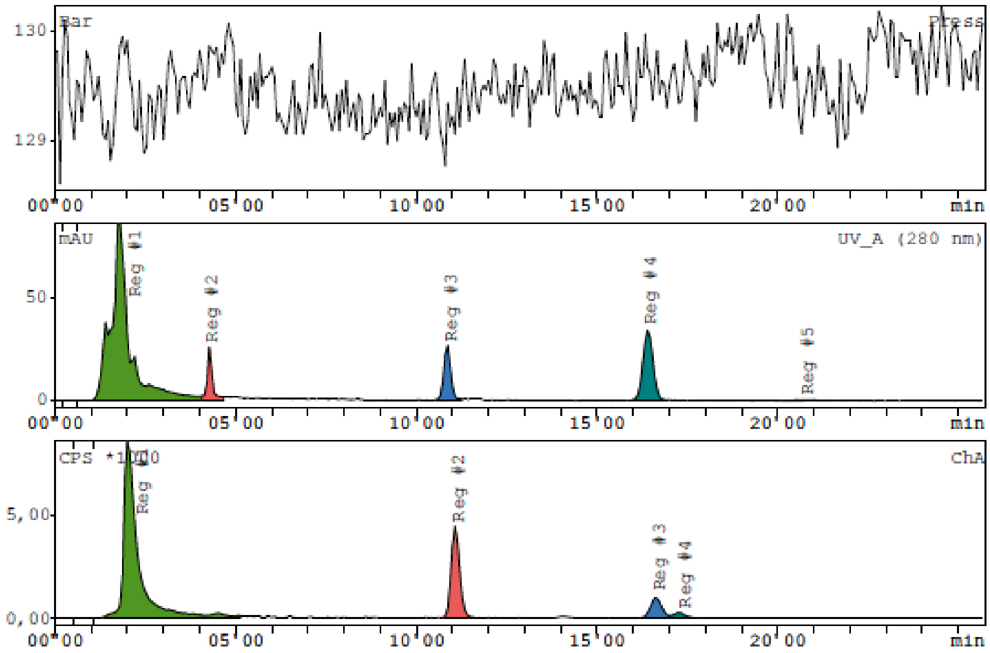

Measurement:	161212_Cu8, injection : 12.12.2016 14:12
Method:	18F-LANSOPRAZOLE from: 12.12.2016 11:45

Mobile Phase: AcN:Water:30:70
Flow rate: 2 mL/mim
PFP (2) 150 x 4.6 mm
Method changed: 09/12/2016
Mobile Phase: AcN:Water:60:40
Flow rate: 1,5 mL/mim
PFP (2) 250 x 4.6 mm
Column PFP(2) 150*4.6mm (Used (250*4.6mm) 161209)
Flow rate: 2mL/min
Wavelenght: 280
Radio detector: raytest Gabi Star Serial Nr.: #30745 raytest GINA star 20.04.09 Firmware V4.8
Software Version: 5.9, Service Pack 8, Build 5076

ChA

Substance	R/T s	Type	Area Counts	%Area %
Reg #1	02'04	DD(M	236387,6	70,64
Reg #2	11'04	DD(M	71408,8	21,34
Reg #3	16'40	DD(M	21046,4	6,29
Reg #4	17'20	DD(M	5817,2	1,74
Sum in ROI			334660,1	100,00
Area (total)			348752,3	
BKG1			24,31	
Remainder			14092,26	4,04

Figure S66: Analytical HPLC chromatogram from screening, top channel = UV, bottom channel = activity. HPLC spectrum of 1,1-difluoro-1-(fluoro-^{18}F)propan-2-yl benzenesulfonate **([^{18}F]2b).** (**2a**), DMSO, 85°C, CuOAC, N_2, TEMPO, 5 min. 100 µL organic phase in 1 mL MeCN: H_2O=50:50. **(**MeCN: H_2O =30:70. Flow rate = 2.0 mL/min. Injected volume = 10 µL). (Table S6, entry 9).

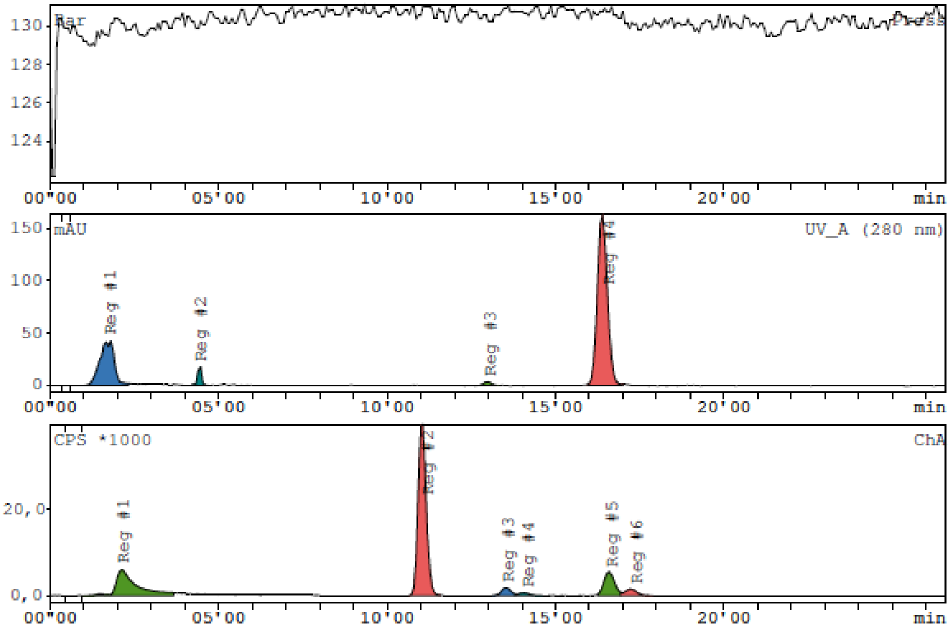

Measurement: 161212Cu5, injection :	12.12.2016 13:02
Method: 18F-LANSOPRAZOLE from:	12.12.2016 11:45

Mobile Phase: AcN:Water:30:70
Flow rate: 2 mL/mim
PFP (2) 150 x 4.6 mm
Method changed: 09/12/2016
Mobile Phase: AcN:Water:60:40
Flow rate: 1,5 mL/mim
PFP (2) 250 x 4.6 mm
Column PFP(2) 150*4.6mm (Used (250*4.6mm) 161209)
Flow rate: 2mL/min
Wavelenght: 280
Radio detector: raytest Gabi Star Serial Nr.: #30745 raytest GINA star 20.04.09 Firmware V4.8
Software Version: 5.9, Service Pack 8, Build 5076

ChA

Substance	R/T s	Type	Area Counts	%Area %
Reg #1	02'08	DD(M	243504,3	21,97
Reg #2	11'04	DD(M	665304,9	60,02
Reg #3	13'32	DD(M	32814,2	2,96
Reg #4	14'08	DD(M	13690,7	1,24
Reg #5	16'36	DD(M	116096,0	10,47
Reg #6	17'20	DD(M	37138,5	3,35
Sum in ROI			1108548,6	100,00
Area (total)			1166489,2	
BKG1			55,29	
Remainder			57940,57	4,97

*Figure S67: Analytical HPLC chromatogram from screening, top channel = UV, bottom channel = activity. HPLC spectrum of 1,1-difluoro-1-(fluoro-^{18}F)propan-2-yl benzenesulfonate **([^{18}F]2b)**. (**2a**), DMSO, 85°C, CuOTf, air, 5 min. 100 µL organic phase in 1 mL MeCN: H_2O=50:50. **(**MeCN: H_2O =30:70. Flow rate = 2.0 mL/min. Injected volume = 10 µL). (Table S6, entry 10).*

Measurement cf3ots_rx ohne Ag Uni Oslo Page 1/2
c:\GINA_NT\18F-Lansoprazole\cf3ots_rx ohne Ag

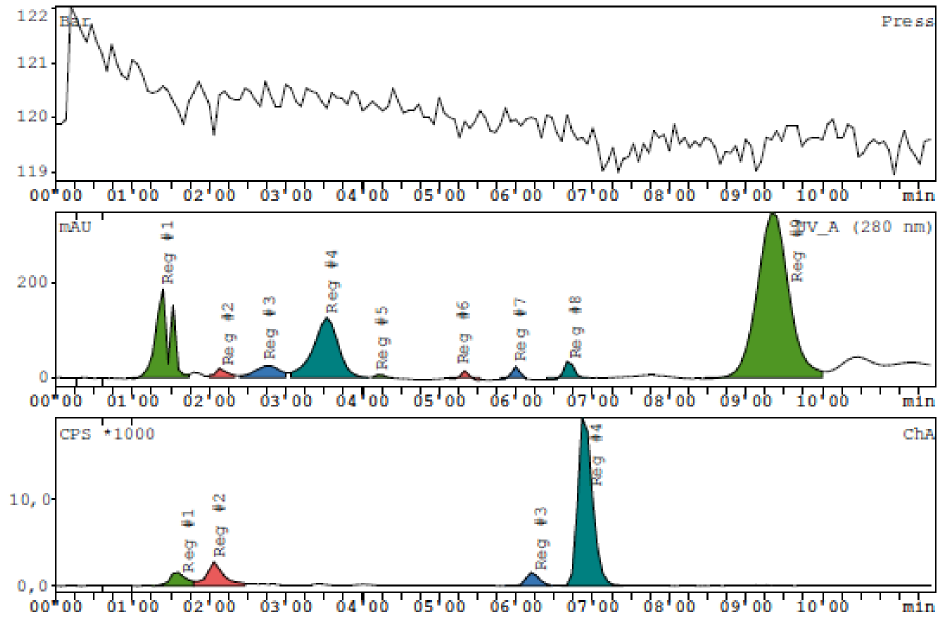

Measurement: cf3ots_rx ohne Ag, injection : 16.12.2016 15:07
Method: 18F-LANSOPRAZOLE from: 15.12.2016 14:50
Mobile Phase: AcN:Water:30:70
Flow rate: 2 mL/mim
PFP (2) 150 x 4.6 mm
Method changed: 09/12/2016
Mobile Phase: AcN:Water:60:40
Flow rate: 1,5 mL/mim
PFP (2) 250 x 4.6 mm
Method changed: 12/12/2016
Mobile Phase: AcN:Water:50:50
Flow rate: 1,5 mL/mim
PFP (2) 250 x 4.6 mm
Column PFP(2) 150*4.6mm (Used (250*4.6mm) 161209)
Flow rate: 2mL/min
Wavelenght: 280
Radio detector: raytest Gabi Star Serial Nr.: #30745 raytest GINA star 20.04.09 Firmware V4.8
Software Version: 5.9, Service Pack 8, Build 5076

ChA

Substance	R/T s	Type	Area Counts	%Area %
Reg #1	01'40	DD(M	21828,1	6,41
Reg #2	02'04	DD(M	43423,7	12,74
Reg #3	06'16	DD(M	18318,3	5,38
Reg #4	06'56	DD(M	257207,0	75,48
Sum in ROI			340777,0	100,00
Area (total)			370279,3	
BKG1			4,33 CPS	

Figure S68: Analytical HPLC chromatogram from screening, top channel = UV, bottom channel = activity. HPLC spectrum of 1,1-difluoro-1-(fluoro-^{18}F)propan-2-yl benzenesulfonate **([^{18}F]2b).** (**2a**), DMSO, 85°C, $CuBH_4$, 5 min. 100 µL organic phase in 1 mL MeCN: H_2O=50:50. **(**MeCN: H_2O =30:70. Flow rate = 2.0 mL/min. Injected volume = 10 µL). (Table S6, entry 11).

Scheme 1, Pathway II:

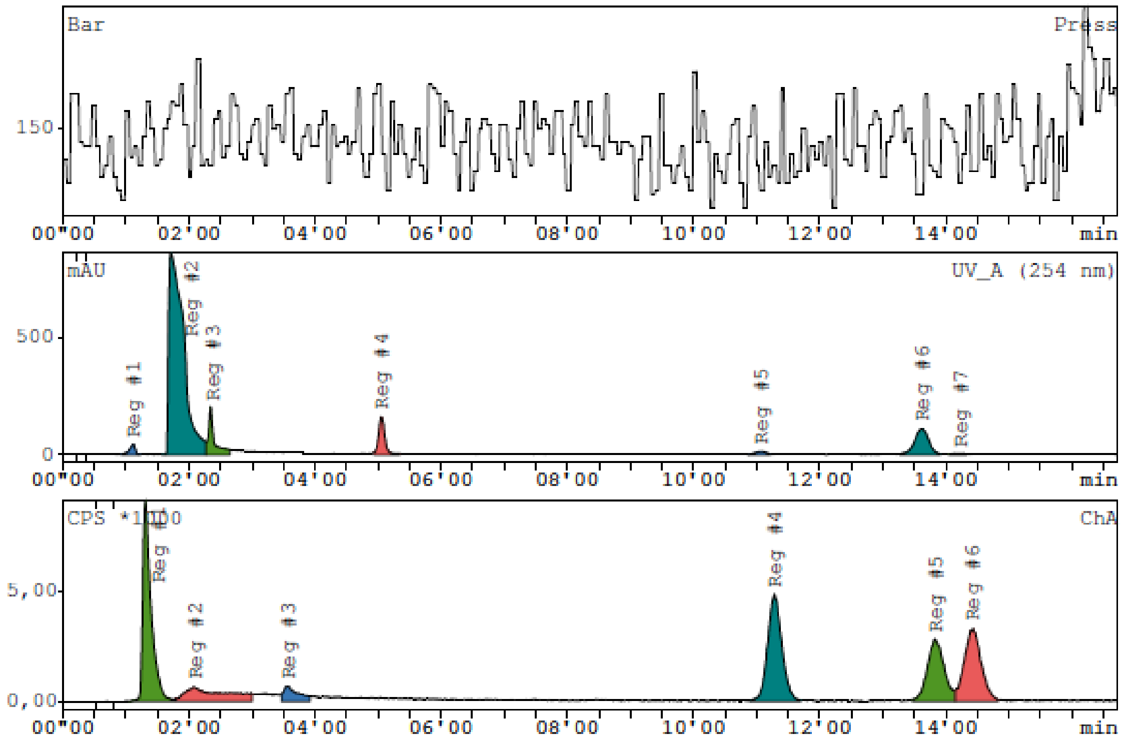

Sample description

Measurement:	200121-D, injection :	20.01.2021 11:45	
Method:	LUNAPFP Mudasir AcN_H2O 50_50 from:		17.12.2020 09:30
1 minute, CH3CN:H2O=50:50, Flow rate 1,5 mL/min, injection 10 micro L			
Radio detector: raytest Gabi Star	Serial Nr.: #30745 raytest GINA star 20.04.09 Firmware V4.8		
Software Version: 5.9, Service Pack 8, Build 5076			

Integration ChA

Substance	R/T s	Type	Area Counts	%Area %
Reg #1	01'19	DD(M	83053,98	26,66
Reg #2	02'06	DD(M	28241,35	9,07
Reg #3	03'34	DD(M	11348,01	3,64
Reg #4	11'17	DD(M	75393,13	24,20
Reg #5	13'50	DD(M	51696,40	16,60
Reg #6	14'25	DD(M	61745,95	19,82
Sum in ROI			311478,83	100,00
Area (total)			364254,06	
BKG1			4,882	
Remainder			52775,24	14,49

Figure S69: Analytical HPLC chromatogram from screening, top channel = UV, bottom channel = activity. HPLC spectrum of 2,2-difluoro-2-(fluoro-^{18}F)ethyl 4-methylbenzenesulfonate **([^{18}F]1b).** (**1a**), TEMPO, Et_3SiH, DMSO, 90°C, 2-methyl-2-butanol, 1 min. 100 µL organic phase in 1 mL MeCN: H_2O=50:50. **(**MeCN: H_2O =50:50. Flow rate = 1.5 mL/min. Injected volume = 10 µL). (Table S5, entry 1).

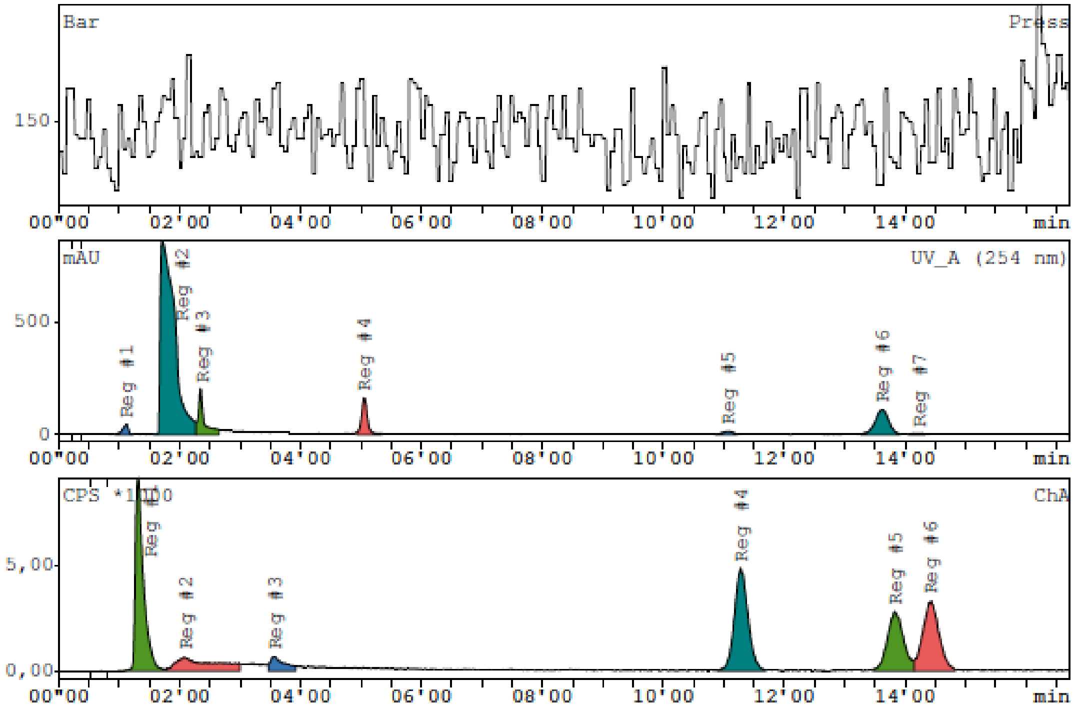

Sample description

Measurement:	200121-D, injection :	20.01.2021 11:45	
Method:	LUNAPFP Mudasir AcN_H2O 50_50 from:		17.12.2020 09:30
1 minute, CH3CN:H2O=50:50, Flow rate 1,5 mL/min, injection 10 micro L			
Radio detector: raytest Gabi Star	Serial Nr.: #30745 raytest GINA star 20.04.09 Firmware V4.8		
Software Version: 5.9, Service Pack 8, Build 5076			

Integration ChA

Substance	R/T s	Type	Area Counts	%Area %
Reg #1	01'19	DD(M	83053,98	26,66
Reg #2	02'06	DD(M	28241,35	9,07
Reg #3	03'34	DD(M	11348,01	3,64
Reg #4	11'17	DD(M	75393,13	24,20
Reg #5	13'50	DD(M	51696,40	16,60
Reg #6	14'25	DD(M	61745,95	19,82
Sum in ROI			311478,83	100,00
Area (total)			364254,06	
BKG1			4,882	
Remainder			52775,24	14,49

Figure S70: Analytical HPLC chromatogram from screening, top channel = UV, bottom channel = activity. HPLC spectrum of 2,2-difluoro-2-(fluoro-^{18}F)ethyl 4-methylbenzenesulfonate **([^{18}F]1b).** (**1a**), TEMPO, Et_3SiH, DMSO, 90°C, 2-methyl-2-butanol, 2 min. 100 µL organic phase in 1 mL MeCN: H_2O =50:50. **(**MeCN: H_2O =50:50. Flow rate = 1.5 mL/min. Injected volume = 10 µL). (Table S5, entry 2).

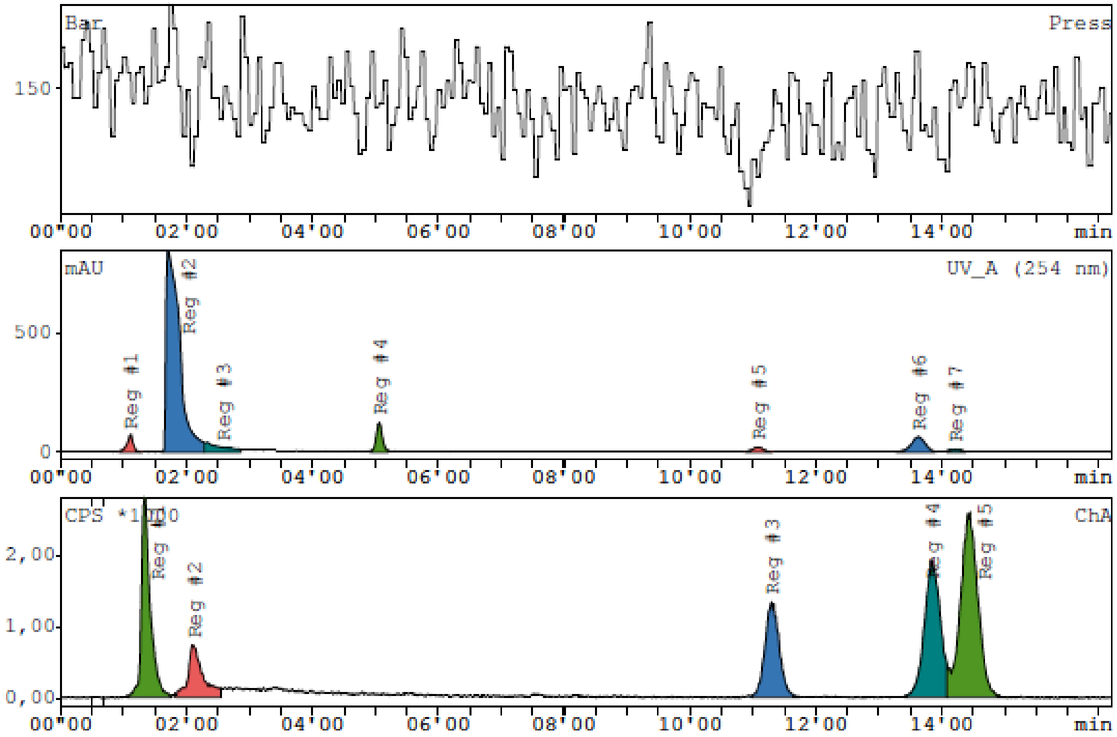

Sample description

Measurement:	200121-E, injection :	20.01.2021 12:03
Method:	LUNAPFP Mudasir AcN_H2O 50_50 from:	17.12.2020 09:30
2 minutes, CH3CN:H2O=50:50, Flow rate 1,5 mL/min, injection 10 micro L		
Radio detector: raytest Gabi Star	Serial Nr.: #30745 raytest GINA star 20.04.09 Firmware V4.8	
Software Version: 5.9, Service Pack 8, Build 5076		

Integration ChA

Substance	R/T s	Type	Area Counts	%Area %
Reg #1	01'20	DD(M	27340,63	19,20
Reg #2	02'07	DD(M	11800,88	8,29
Reg #3	11'18	DD(M	20713,83	14,55
Reg #4	13'51	DD(M	32733,52	22,99
Reg #5	14'26	DD(M	49815,88	34,98
Sum in ROI			142404,75	100,00
Area (total)			160479,09	
BKG1			17,091	
Remainder			18074,34	11,26

Figure S71. Analytical HPLC chromatogram from screening, top channel = UV, bottom channel = activity. HPLC spectrum of 2,2-difluoro-2-(fluoro-^{18}F)ethyl 4-methylbenzenesulfonate **([^{18}F]1b).** (**1a**), TEMPO, Et_3SiH, DMSO, 90°C, 2-methyl-2-butanol, 4 min. 100 µL organic phase in 1 mL MeCN: H_2O =50:50. **(**MeCN: H_2O =50:50. Flow rate = 1.5 mL/min. Injected volume = 10 µL) (Table S5, entry 3).

Measurement 310821-L Uni Oslo Page 1/2
c:\GINA_NT\LUNAPFP Mudasir AcN_H2O 50_50\310821-L

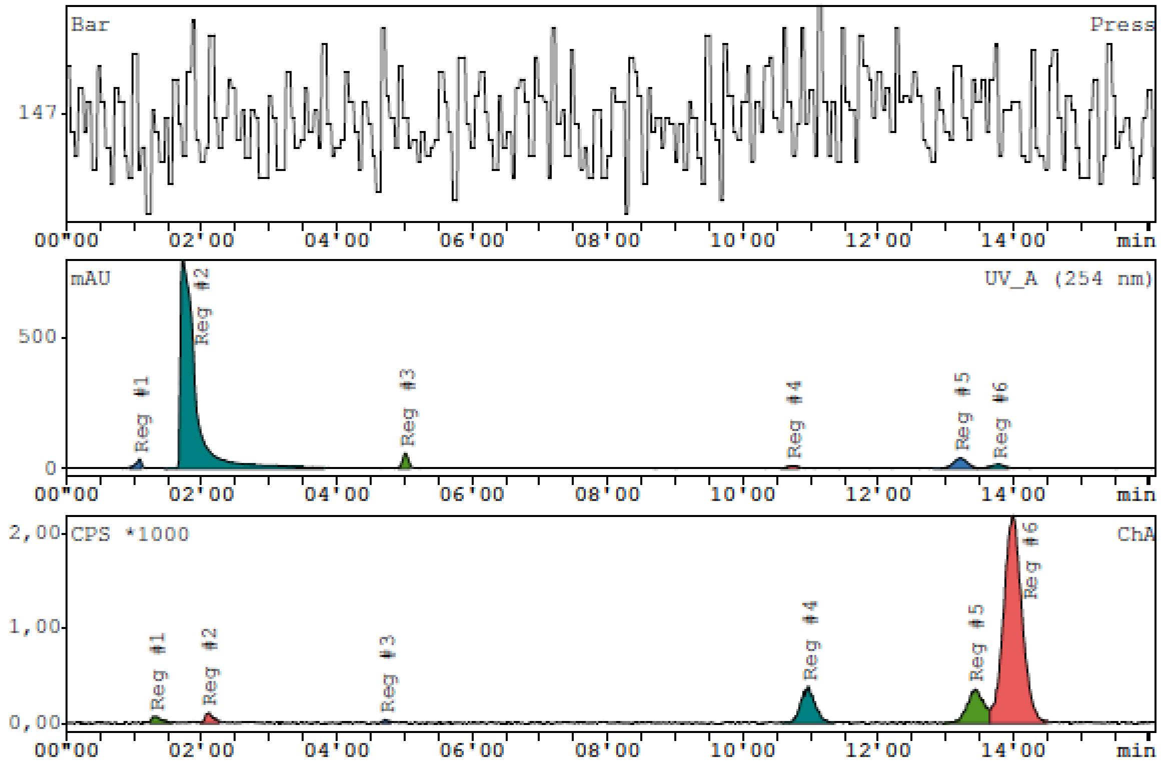

Measurement:	310821-L, injection :	31.08.2021 15:48	
Method:	LUNAPFP Mudasir AcN_H2O 50_50 from:	17.12.2020 09:30	
CH3CN:H2O=50:50, Flow rate 1,5 mL/min, injection 10 micro L			
Radio detector: raytest Gabi Star	Serial Nr.: #30745 raytest GINA star 20.04.09 Firmware V4.8		
Software Version: 5.9, Service Pack 8, Build 5076			

ChA

Substance	R/T s	Type	Area Counts	%Area %
Reg #1	01'19	DD(M	793,07	1,45
Reg #2	02'06	DD(M	950,12	1,73
Reg #3	04'43	DD(M	221,01	0,40
Reg #4	10'58	DD(M	5718,32	10,44
Reg #5	13'26	DD(M	6074,50	11,09
Reg #6	14'00	DD(M	41031,73	74,89
Sum in ROI			54788,75	100,00
Area (total)			61097,85	
Ext. BKG			0,00 CPS	

Figure S72. Analytical HPLC chromatogram from screening, top channel = UV, bottom channel = activity. HPLC spectrum of 2,2-difluoro-2-(fluoro-^{18}F)ethyl 4-methylbenzenesulfonate **([^{18}F]1b).** (**1a**), TEMPO, Et_3SiH, DMSO, 90°C, 2-methyl-2-butanol, 10 min. 100 µL organic phase in 1 mL MeCN: H_2O =50:50. **(**MeCN: H_2O =50:50. Flow rate = 1.5 mL/min. Injected volume = 10 µL) (Table S5, entry 5).

Measurement 310821-M Uni Oslo Page 1/2
c:\GINA_NT\LUNAPFP Mudasir AcN_H2O 50_50\310821-M

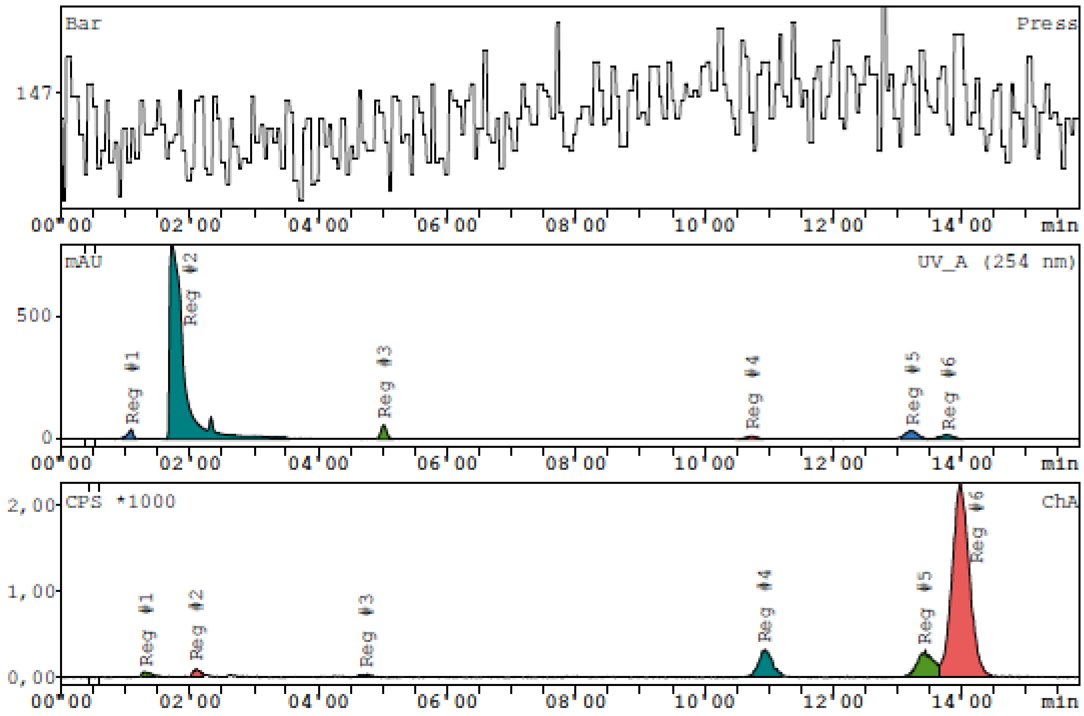

Measurement:	310821-M, injection : 31.08.2021 16:05
Method:	LUNAPFP Mudasir AcN_H2O 50_50 from: 17.12.2020 09:30
CH3CN:H2O=50:50, Flow rate 1,5 mL/min, injection 10 micro L	
Radio detector: raytest Gabi Star	Serial Nr.: #30745 raytest GINA star 20.04.09 Firmware V4.8
Software Version: 5.9, Service Pack 8, Build 5076	

ChA

Substance	R/T s	Type	Area Counts	%Area %
Reg #1	01'18	DD(M	539,80	1,02
Reg #2	02'06	DD(M	730,71	1,38
Reg #3	04'44	DD(M	298,76	0,56
Reg #4	10'56	DD(M	4690,23	8,85
Reg #5	13'24	DD(M	5157,84	9,73
Reg #6	13'58	DD(M	41579,08	78,46
Sum in ROI			52996,42	100,00
Area (total)			54518,97	
BKG1			4,875	
Remainder			1522,56	2,79

Figure S73. Analytical HPLC chromatogram from screening, top channel = UV, bottom channel = activity. HPLC spectrum of 2,2-difluoro-2-(fluoro-^{18}F)ethyl 4-methylbenzenesulfonate **([^{18}F]1b).** (**1a**), TEMPO, Et_3SiH, DMSO, 90°C, 2-methyl-2-butanol, 20 min. 100 µL organic phase in 1 mL MeCN: H_2O=50:50. **(**MeCN: H_2O =50:50. Flow rate = 1.5 mL/min. Injected volume = 10 µL) (Table S5, entry 6).

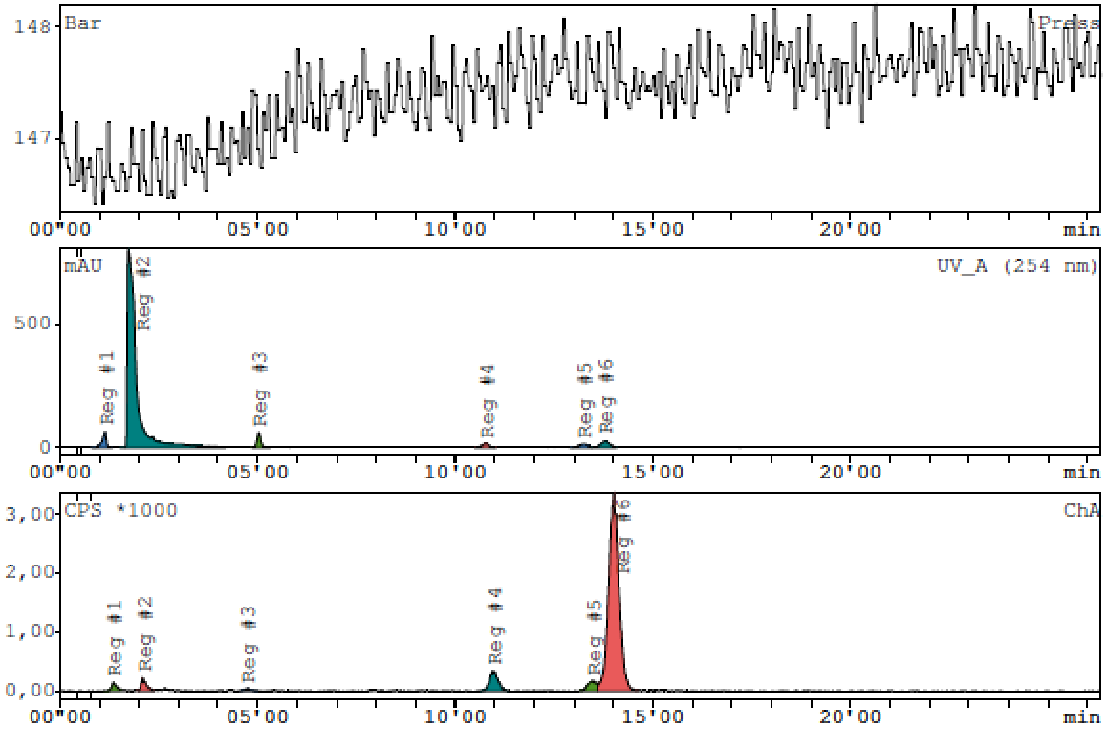

Measurement:	310821-N, injection : 31.08.2021 16:21
Method:	LUNAPFP Mudasir AcN_H2O 50_50 from: 17.12.2020 09:30
CH3CN:H2O=50:50, Flow rate 1,5 mL/min, injection 10 micro L	
Radio detector: raytest Gabi Star	Serial Nr.: #30745 raytest GINA star 20.04.09 Firmware V4.8
Software Version: 5.9, Service Pack 8, Build 5076	

ChA

Substance	R/T s	Type	Area Counts	%Area %
Reg #1	01'22	DD(M	1187,18	1,67
Reg #2	02'07	DD(M	1869,40	2,63
Reg #3	04'44	DD(M	431,02	0,61
Reg #4	10'59	DD(M	4987,91	7,00
Reg #5	13'29	DD(M	2443,54	3,43
Reg #6	14'01	DD(M	60292,55	84,67
Sum in ROI			71211,61	100,00
Area (total)			74172,70	
BKG1			4,000	
Remainder			2961,09	3,99

Figure S74. Analytical HPLC chromatogram from screening, top channel = UV, bottom channel = activity. HPLC spectrum of 2,2-difluoro-2-(fluoro-^{18}F)ethyl 4-methylbenzenesulfonate **([^{18}F]1b). 1a**, TEMPO, Et_3SiH, DMSO, 90°C, 2-methyl-2-butanol, 30 min. 100 µL organic phase in 1 mL MeCN: H_2O =50:50. **(**MeCN: H_2O =50:50. Flow rate = 1.5 mL/min. Injected volume = 10 µL) (Table S5, entry 7).

Measurement 260121-D Uni Oslo Page 1/1
c:\GINA_NT\LUNAPFP Mudasir AcN_H2O 50_50\260121-D

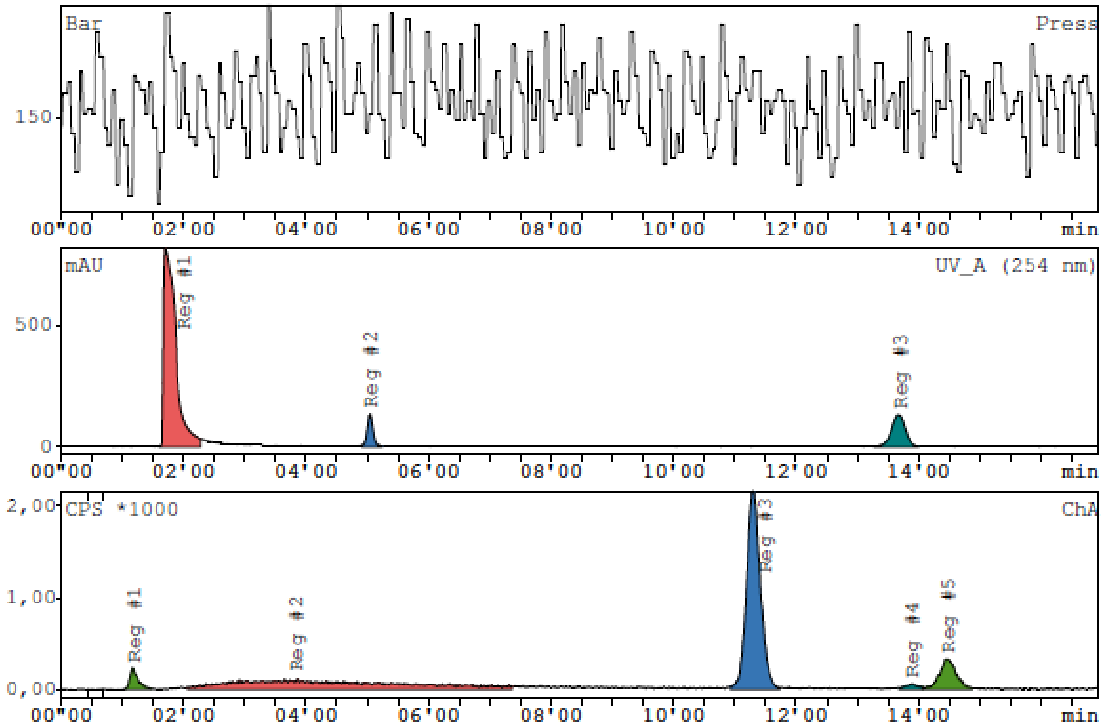

Sample description

Measurement:	260121-D, injection : 26.01.2021 12:29
Method:	LUNAPFP Mudasir AcN_H2O 50_50 from: 17.12.2020 09:30
2-propanol, 1 minute rxn, CH3CN:H2O=50:50, Flow rate=1,5 ml/min, injection volume=10 micro L	
Radio detector: raytest Gabi Star Serial Nr.: #30745 raytest GINA star 20.04.09 Firmware V4.8	
Software Version: 5.9, Service Pack 8, Build 5076	

Integration ChA

Substance	R/T s	Type	Area Counts	%Area %
Reg #1	01'11	DD(M	2118,43	3,37
Reg #2	03'50	DD(M	19592,18	31,21
Reg #3	11'18	DD(M	33829,39	53,89
Reg #4	13'53	DD(M	862,20	1,37
Reg #5	14'28	DD(M	6374,40	10,15
Sum in ROI			62776,61	100,00
Area (total)			70115,38	
BKG1			2,733	
Remainder			7338,77	10,47

Figure S75. Analytical HPLC chromatogram from screening, top channel = UV, bottom channel = activity. HPLC spectrum of 2,2-difluoro-2-(fluoro-^{18}F)ethyl 4-methylbenzenesulfonate ***([^{18}F]1b). 1a****, TEMPO, Et_3SiH, DMSO, 90°C, 2-propanol , 1 min. 100 µL organic phase in 1 mL MeCN: H_2O =50:50. (MeCN: H_2O =50:50. Flow rate = 1.5 mL/min. Injected volume = 10 µL)* (Table S5, entry 8).

Measurement 260121-E Uni Oslo Page 1/1

c:\GINA_NT\LUNAPFP Mudasir AcN_H2O 50_50\260121-E

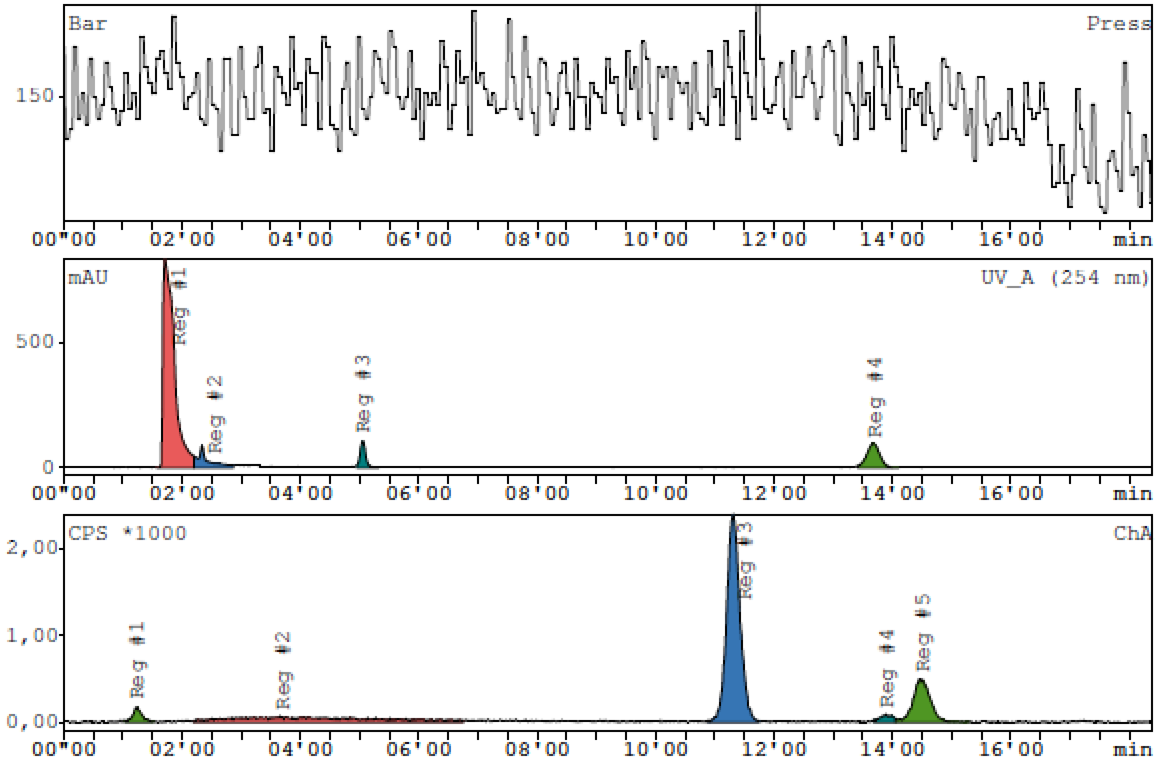

Sample description

Measurement: 260121-E, injection : 26.01.2021 12:47	
Method: LUNAPFP Mudasir AcN_H2O 50_50 from: 17.12.2020 09:30	
2-propanol, 2 minute rxn, CH3CN:H2O=50:50, Flow rate=1,5 ml/min, injection volume=10 micro L	
Radio detector: raytest Gabi Star Serial Nr.: #30745 raytest GINA star 20.04.09 Firmware V4.8	
Software Version: 5.9, Service Pack 8, Build 5076	

Integration ChA

Substance	R/T s	Type	Area Counts	%Area %
Reg #1	01'14	DD(M	1979,34	3,17
Reg #2	03'40	DD(M	11106,79	17,80
Reg #3	11'18	DD(M	37595,83	60,24
Reg #4	13'52	DD(M	1332,14	2,13
Reg #5	14'29	DD(M	10392,66	16,65
Sum in ROI			62406,76	100,00
Area (total)			70559,96	
Ext. BKG			0,00 CPS	

Figure S76. Analytical HPLC chromatogram from screening, top channel = UV, bottom channel = activity. HPLC spectrum of 2,2-difluoro-2-(fluoro-^{18}F)ethyl 4-methylbenzenesulfonate **([^{18}F]1b). 1a**, TEMPO, Et_3SiH, DMSO, 90°C, 2-propanol , 2 min. 100 µL organic phase in 1 mL MeCN: H_2O =50:50. **(**MeCN: H_2O =50:50. Flow rate = 1.5 mL/min. Injected volume = 10 µL) (Table S5, entry 9).

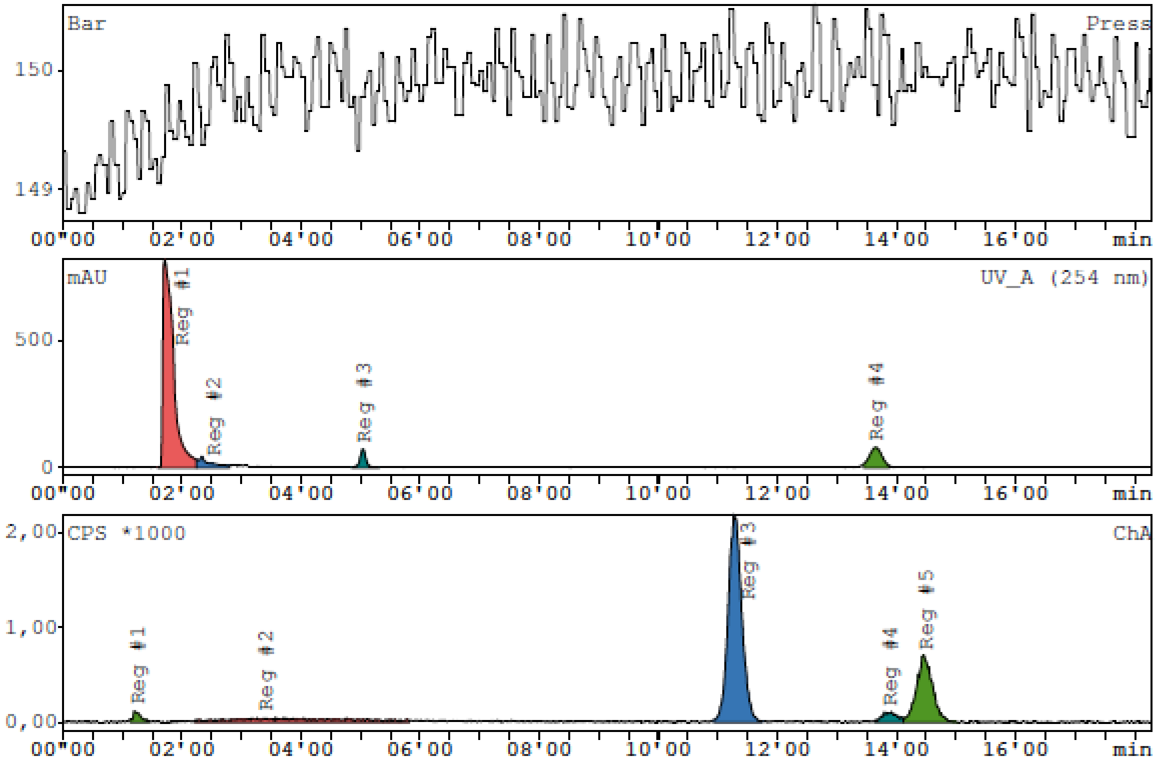

Sample description

Measurement:	260121-F, injection : 26.01.2021 13:06
Method:	LUNAPFP Mudasir AcN_H2O 50_50 from: 17.12.2020 09:30
2-propanol, 4 minute rxn, CH3CN:H2O=50:50, Flow rate=1,5 ml/min, injection volume=10 micro L	
Radio detector: raytest Gabi Star	Serial Nr.: #30745 raytest GINA star 20.04.09 Firmware V4.8
Software Version: 5.9, Service Pack 8, Build 5076	

Integration ChA

Substance	R/T s	Type	Area Counts	%Area %
Reg #1	01'14	DD(M	1093,14	1,92
Reg #2	03'24	DD(M	6245,31	10,97
Reg #3	11'17	DD(M	34516,78	60,65
Reg #4	13'53	DD(M	1920,24	3,37
Reg #5	14'28	DD(M	13137,91	23,08
Sum in ROI			56913,39	100,00
Area (total)			63880,52	
Ext. BKG			0,00 CPS	

Figure S77. Analytical HPLC chromatogram from screening, top channel = UV, bottom channel = activity. HPLC spectrum of 2,2-difluoro-2-(fluoro-^{18}F)ethyl 4-methylbenzenesulfonate **([^{18}F]1b). 1a**, TEMPO, Et_3SiH, DMSO, 90°C, 2-propanol, 4 min. 100 µL organic phase in 1 mL MeCN: H_2O =50:50. **(**MeCN: H_2O =50:50. Flow rate = 1.5 mL/min. Injected volume = 10 µL) (Table S5, entry 10).

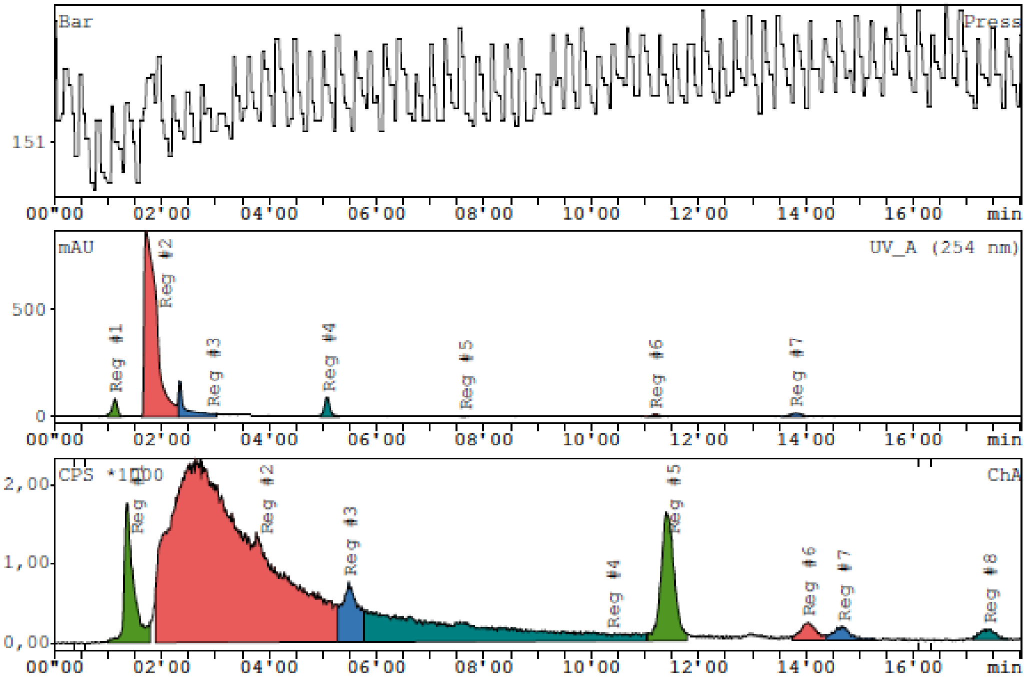

Sample description

Measurement:	200121-B, injection : 20.01.2021 10:26
Method:	LUNAPFP Mudasir AcN_H2O 50_50 from: 17.12.2020 09:30
hexafluoro-propanol, CH3CN:H2O=50:50, Flow rate 1,5 mL/min, injection 10 micro L	
Radio detector: raytest Gabi Star	Serial Nr.: #30745 raytest GINA star 20.04.09 Firmware V4.8
Software Version: 5.9, Service Pack 8, Build 5076	

Integration ChA

Substance	R/T s	Type	Area Counts	%Area %
Reg #1	01'22	DD(M	20982,0	5,40
Reg #2	02'40	DD(M	259260,7	66,67
Reg #3	05'30	DD(M	15180,7	3,90
Reg #4	10'23	DD(M	55186,9	14,19
Reg #5	11'25	DD(M	27295,3	7,02
Reg #6	14'02	DD(M	4540,7	1,17
Reg #7	14'41	DD(M	3686,1	0,95
Reg #8	17'24	DD(M	2713,4	0,70
Sum in ROI			388845,8	100,00
Area (total)			397000,4	
BKG1			6,33 CPS	
BKG2			43,86	
Remainder			8154,63	2,05

Figure S78. Analytical HPLC chromatogram from screening, top channel = UV, bottom channel = activity. HPLC spectrum of 2,2-difluoro-2-(fluoro-^{18}F)ethyl 4-methylbenzenesulfonate **([^{18}F]1b). 1a**, TEMPO, Et_3SiH, DMSO, 90°C, 1,1,1,3,3,3-hexafluoro-2-propanol**,** 6 min. 100 µL organic phase in 1 mL MeCN: H_2O =50:50. **(**MeCN: H_2O =50:50. Flow rate = 1.5 mL/min. Injected volume = 10 µL) (Table S5, entry 12).

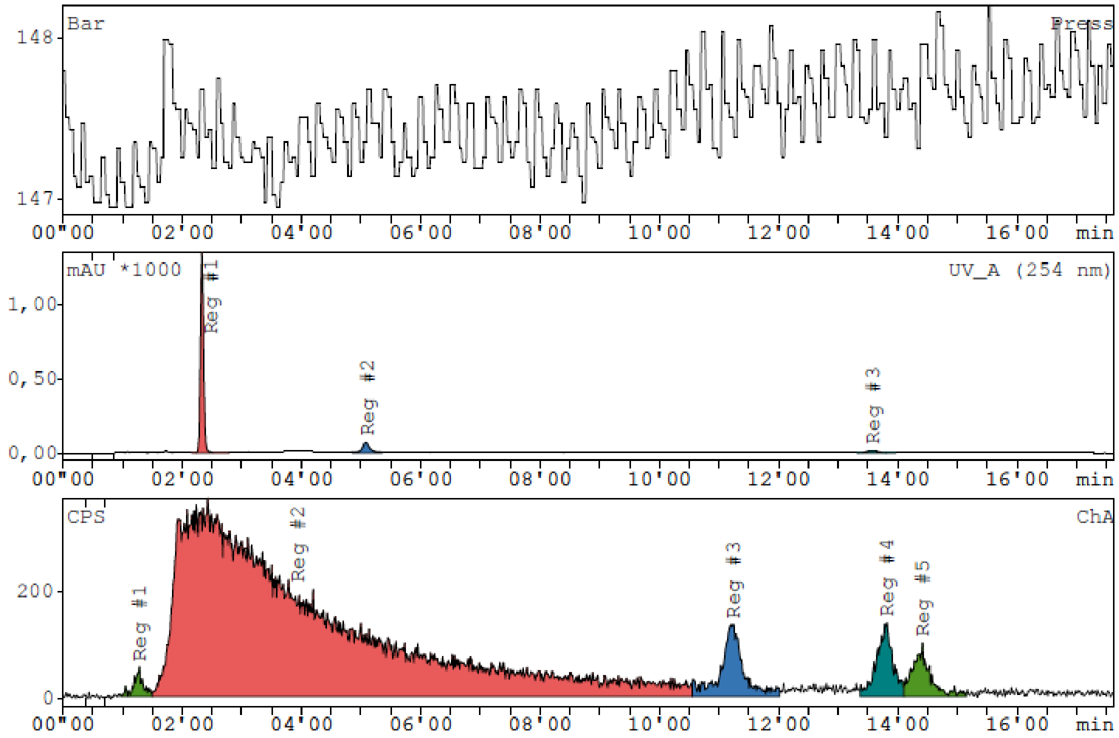

Sample description

Measurement:	181220-I, injection :	18.12.2020 14:22
Method:	LUNAPFP Mudasir AcN_H2O 50_50 from:	17.12.2020 09:30
Actonitrile:H20 =50:50, Flow 1,5 mL/min, injection 10 micro L.		
Radio detector: raytest Gabi Star	Serial Nr.: #30745 raytest GINA star 20.04.09 Firmware V4.8	
Software Version: 5.9, Service Pack 8, Build 5076		

Integration ChA

Substance	R/T s	Type	Area Counts	%Area %
Reg #1	01'15	DD(M	543,98	0,79
Reg #2	02'25	DD(M	60671,11	87,88
Reg #3	11'13	DD(M	3427,58	4,96
Reg #4	13'48	DD(M	2494,43	3,61
Reg #5	14'23	DD(M	1902,24	2,76
Sum in ROI			69039,34	100,00
Area (total)			71239,95	
BKG1			5,158	
Remainder			2200,60	3,09

Figure S79: Analytical HPLC chromatogram from screening, top channel = UV, bottom channel = activity. HPLC spectrum of 2,2-difluoro-2-(fluoro-^{18}F)ethyl 4-methylbenzenesulfonate **([^{18}F]1b). 1a**, $(Bu)_4SnH$, TEMPO, DMSO, 90°C, Ag (II), 6 min. 100 µL organic phase in 1 mL MeCN: H_2O =50:50. **(**MeCN: H_2O =50:50. Flow rate = 1.5 mL/min. Injected volume = 10 µL).

Measurement 181220-B Uni Oslo Page 1/2

c:\GINA_NT\LUNAPFP Mudasir AcN_H2O 50_50\181220-B

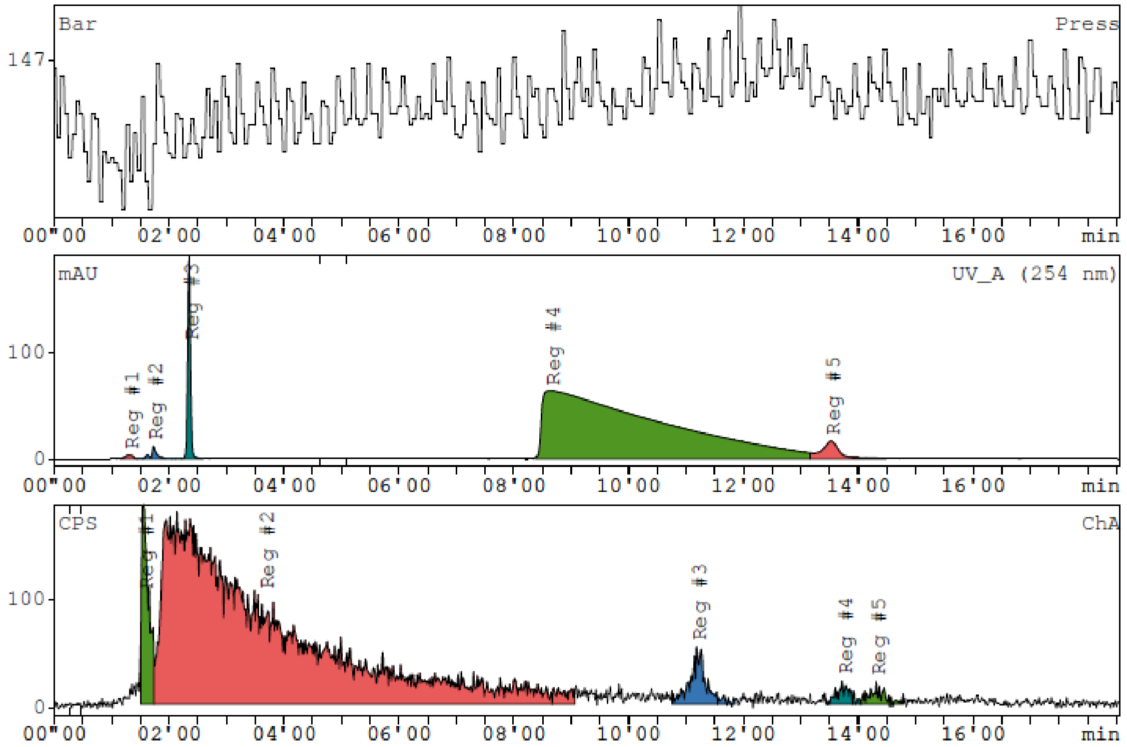

Sample description

Measurement:	181220-B, injection : 18.12.2020 11:10
Method:	LUNAPFP Mudasir AcN_H2O 50_50 from: 17.12.2020 09:30
Actonitrile:H20 =50:50, Flow 1,5 mL/min, injection 10 micro L.	
Radio detector: raytest Gabi Star	Serial Nr.: #30745 raytest GINA star 20.04.09 Firmware V4.8
Software Version: 5.9, Service Pack 8, Build 5076	

Integration ChA

Substance	R/T s	Type	Area Counts	%Area %
Reg #1	01'34	DD(M	1591,91	6,04
Reg #2	01'56	DD(M	23254,83	88,22
Reg #3	11'13	DD(M	939,23	3,56
Reg #4	13'45	DD(M	293,55	1,11
Reg #5	14'20	DD(M	279,19	1,06
Sum in ROI			26358,71	100,00
Area (total)			28227,85	
BKG1			3,818	
Remainder			1869,14	6,62

Figure S80: Analytical HPLC chromatogram from screening, top channel = UV, bottom channel = activity. HPLC spectrum of 2,2-difluoro-2-(fluoro-^{18}F)ethyl 4-methylbenzenesulfonate **([^{18}F]1b). 1a**, Et_3SiH, DMSO, Cu (II)$(Py)_4$OTf , 6 min. 100 µL organic phase in 1 mL MeCN: H_2O =50:50. **(**MeCN: H_2O =50:50. Flow rate = 1.5 mL/min. Injected volume = 10 µL).

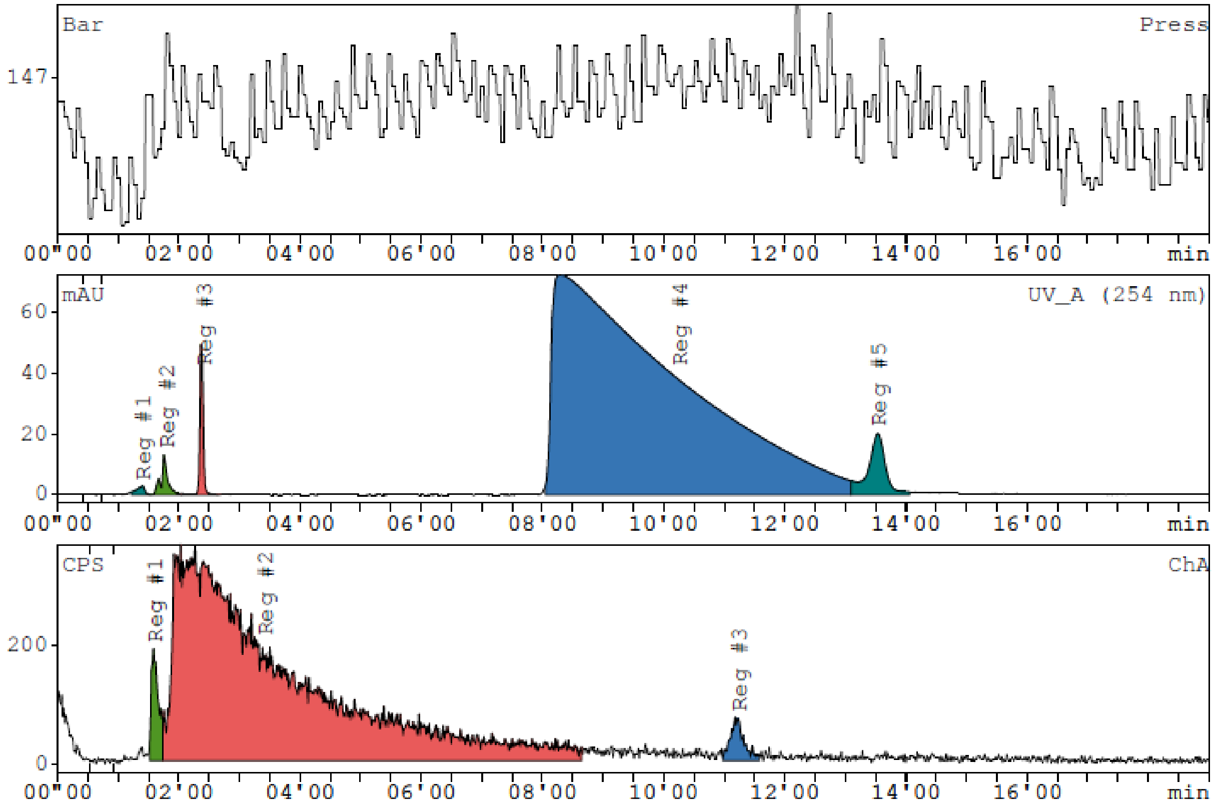

Sample description

Measurement:	181220-C, injection :	18.12.2020 11:30	
Method:	LUNAPFP Mudasir AcN_H2O 50_50 from:		17.12.2020 09:30
Actonitrile:H20 =50:50, Flow 1,5 mL/min, injection 10 micro L.			
Radio detector: raytest Gabi Star	Serial Nr.: #30745 raytest GINA star 20.04.09 Firmware V4.8		
Software Version: 5.9, Service Pack 8, Build 5076			

Integration ChA

Substance	R/T s	Type	Area Counts	%Area %
Reg #1	01'35	DD(M	1466,10	3,05
Reg #2	02'14	DD(M	45575,88	94,70
Reg #3	11'13	DD(M	1087,11	2,26
Sum in ROI			48129,09	100,00
Area (total)			52732,16	
BKG1			7,250	
Remainder			4603,08	8,73

Figure S81: Analytical HPLC chromatogram from screening, top channel = UV, bottom channel = activity. HPLC spectrum of 2,2-difluoro-2-(fluoro-^{18}F)ethyl 4-methylbenzenesulfonate **([^{18}F]1b). 1a**, $(Bu)_4SnH$, DMSO, Cu (II)$(Py)_4$OTf , 6 min. 100 µL organic phase in 1 mL MeCN: H_2O =50:50. **(**MeCN: H_2O =50:50. Flow rate = 1.5 mL/min. Injected volume = 10 µL).

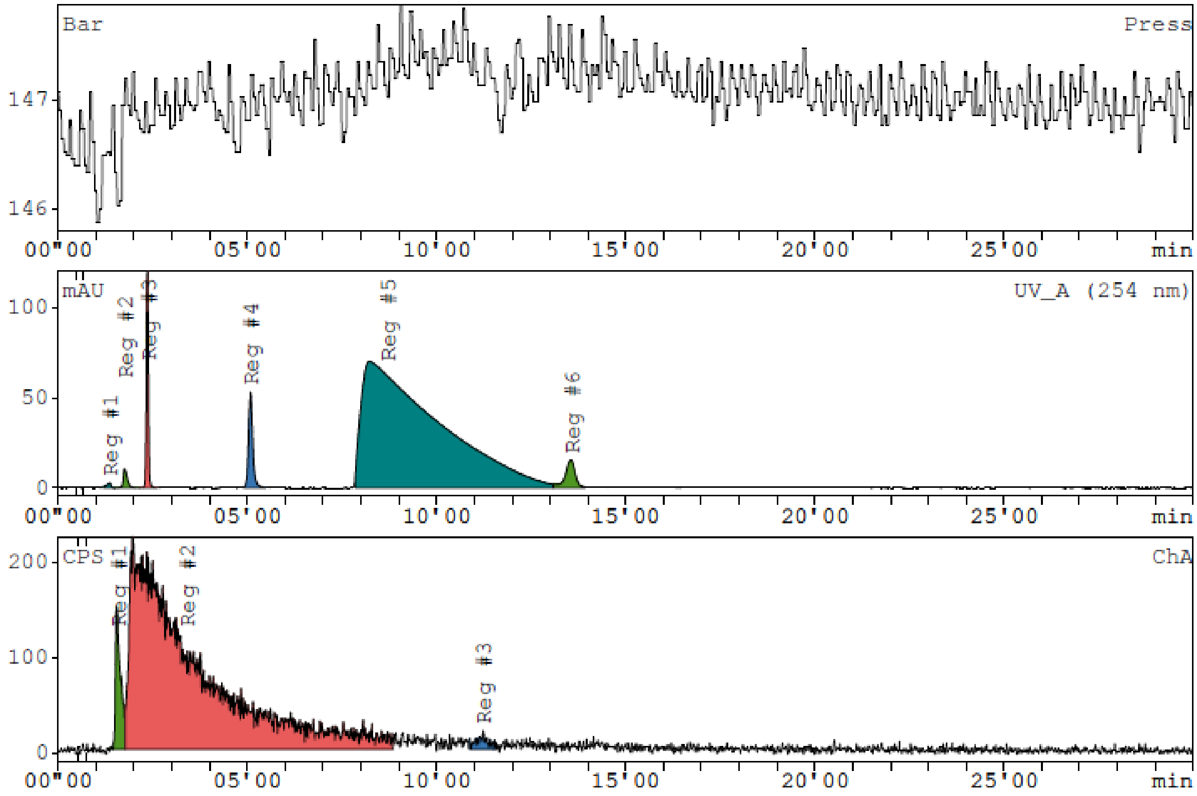

Sample description

Measurement:	181220-D, injection : 18.12.2020 11:59
Method:	LUNAPFP Mudasir AcN_H2O 50_50 from: 17.12.2020 09:30
Actonitrile:H20 =50:50, Flow 1,5 mL/min, injection 10 micro L.	
Radio detector: raytest Gabi Star	Serial Nr.: #30745 raytest GINA star 20.04.09 Firmware V4.8
Software Version: 5.9, Service Pack 8, Build 5076	

Integration ChA

Substance	R/T s	Type	Area Counts	%Area %
Reg #1	01'34	DD(M	1368,24	4,98
Reg #2	01'58	DD(M	25786,21	93,80
Reg #3	11'13	DD(M	336,51	1,22
Sum in ROI			27490,97	100,00
Area (total)			29285,43	
BKG1			4,583	
Remainder			1794,46	6,13

Figure S82: Analytical HPLC chromatogram from screening, top channel = UV, bottom channel = activity. HPLC spectrum of 2,2-difluoro-2-(fluoro-^{18}F)ethyl 4-methylbenzenesulfonate **([^{18}F]1b)**. **1a**, TEMPO, Et_3SiH, DMSO, Cu (II)$(Py)_4$OTf , 6 min. 100 µL organic phase in 1 mL MeCN: H_2O =50:50. **(**MeCN: H_2O =50:50. Flow rate = 1.5 mL/min. Injected volume = 10 µL).

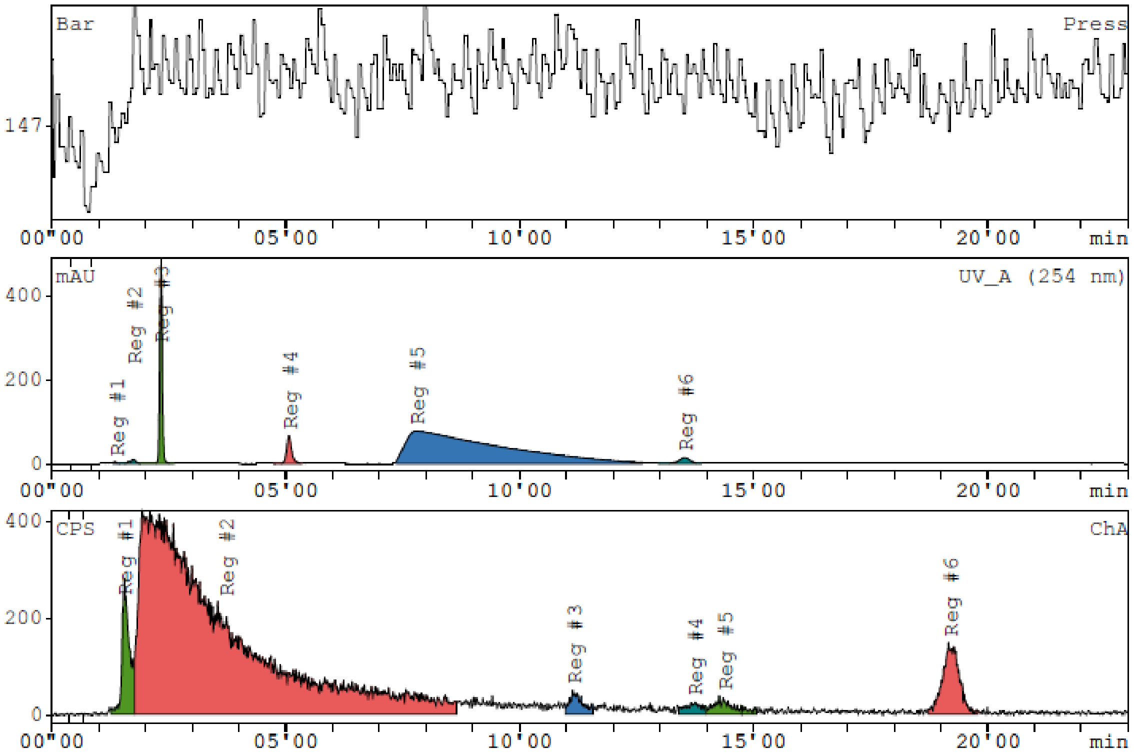

Sample description

Measurement:	181220-E, injection :	18.12.2020 12:34
Method:	LUNAPFP Mudasir AcN_H2O 50_50 from:	17.12.2020 09:30
Actonitrile:H20 =50:50, Flow 1,5 mL/min, injection 10 micro L.		
Radio detector: raytest Gabi Star	Serial Nr.: #30745 raytest GINA star 20.04.09 Firmware V4.8	
Software Version: 5.9, Service Pack 8, Build 5076		

Integration ChA

Substance	R/T s	Type	Area Counts	%Area %
Reg #1	01'33	DD(M	2885,10	4,55
Reg #2	01'58	DD(M	54546,50	86,02
Reg #3	11'10	DD(M	869,52	1,37
Reg #4	13'44	DD(M	573,50	0,90
Reg #5	14'22	DD(M	966,78	1,52
Reg #6	19'12	DD(M	3571,49	5,63
Sum in ROI			63412,89	100,00
Area (total)			69418,41	
BKG1			3,158	
Remainder			6005,52	8,65

Figure S83: Analytical HPLC chromatogram from screening, top channel = UV, bottom channel = activity. HPLC spectrum of 2,2-difluoro-2-(fluoro-^{18}F)ethyl 4-methylbenzenesulfonate **([^{18}F]1b). 1a**, TEMPO, $(Bu)_4SnH$, DMSO, Cu (II)$(Py)_4$OTf , 6 min. 100 µL organic phase in 1 mL MeCN: H_2O =50:50. **(**MeCN: H_2O =50:50. Flow rate = 1.5 mL/min. Injected volume = 10 µL).

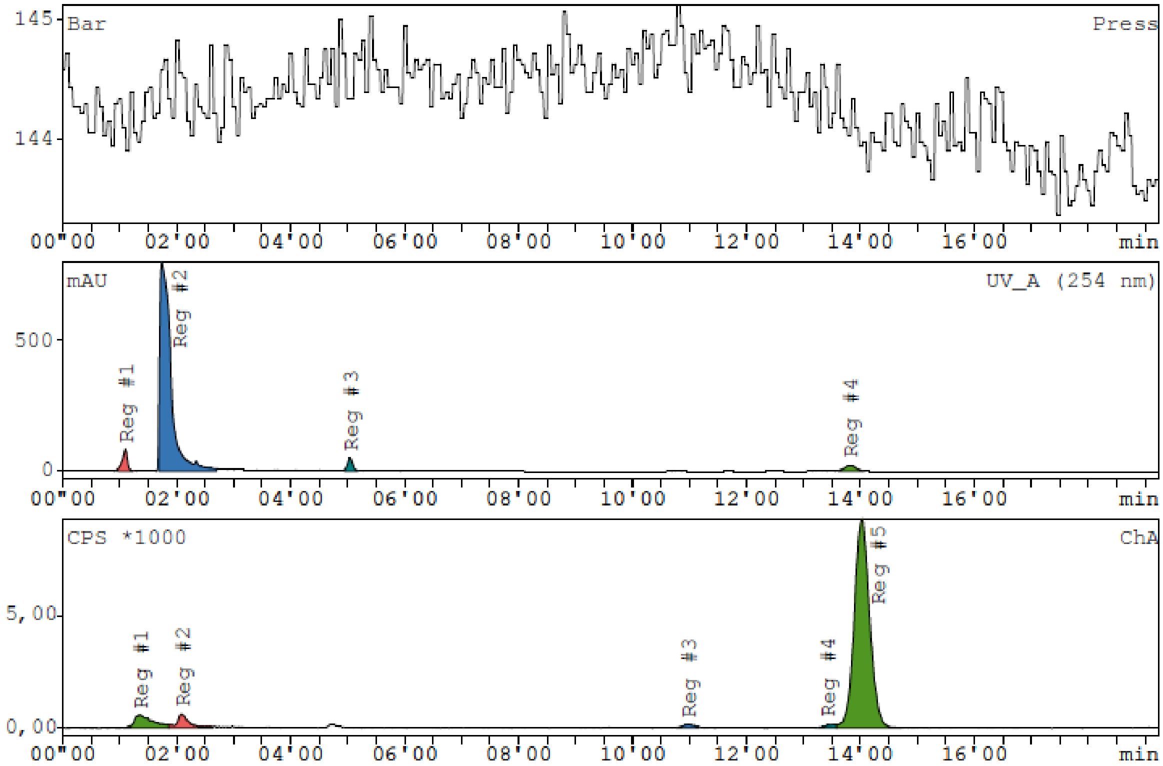

Sample description

Measurement:	090621-H, injection : 09.06.2021 14:11
Method:	LUNAPFP Mudasir AcN_H2O 50_50 from: 17.12.2020 09:30
CH3CN:H2O=50:50, Flow rate 1,5 mL/min, injection 10 micro L	
Radio detector: raytest Gabi Star	Serial Nr.: #30745 raytest GINA star 20.04.09 Firmware V4.8
Software Version: 5.9, Service Pack 8, Build 5076	

Integration ChA

Substance	R/T s	Type	Area Counts	%Area %
Reg #1	01'21	DD(M	12934,9	6,52
Reg #2	02'06	DD(M	9178,2	4,63
Reg #3	10'58	DD(M	2313,5	1,17
Reg #4	13'25	DD(M	1913,4	0,97
Reg #5	14'01	DD(M	171904,7	86,71
Sum in ROI			198244,6	100,00
Area (total)			210831,3	
Ext. BKG			0,00 CPS	

Figure S84: Analytical HPLC chromatogram from screening, top channel = UV, bottom channel = activity. HPLC spectrum of 2,2-difluoro-2-(fluoro-^{18}F)ethyl 4-methylbenzenesulfonate **([^{18}F]1b).** K_2CO_3 in the eluent for [^{18}F], **1a**, TEMPO, Et_3SiH, DMSO, 90°C, 2-methyl-2-butanol, 6 min. 100 µL organic phase in 1 mL MeCN: H_2O =50:50. **(**MeCN: H_2O =50:50. Flow rate = 1.5 mL/min. Injected volume = 10 µL).

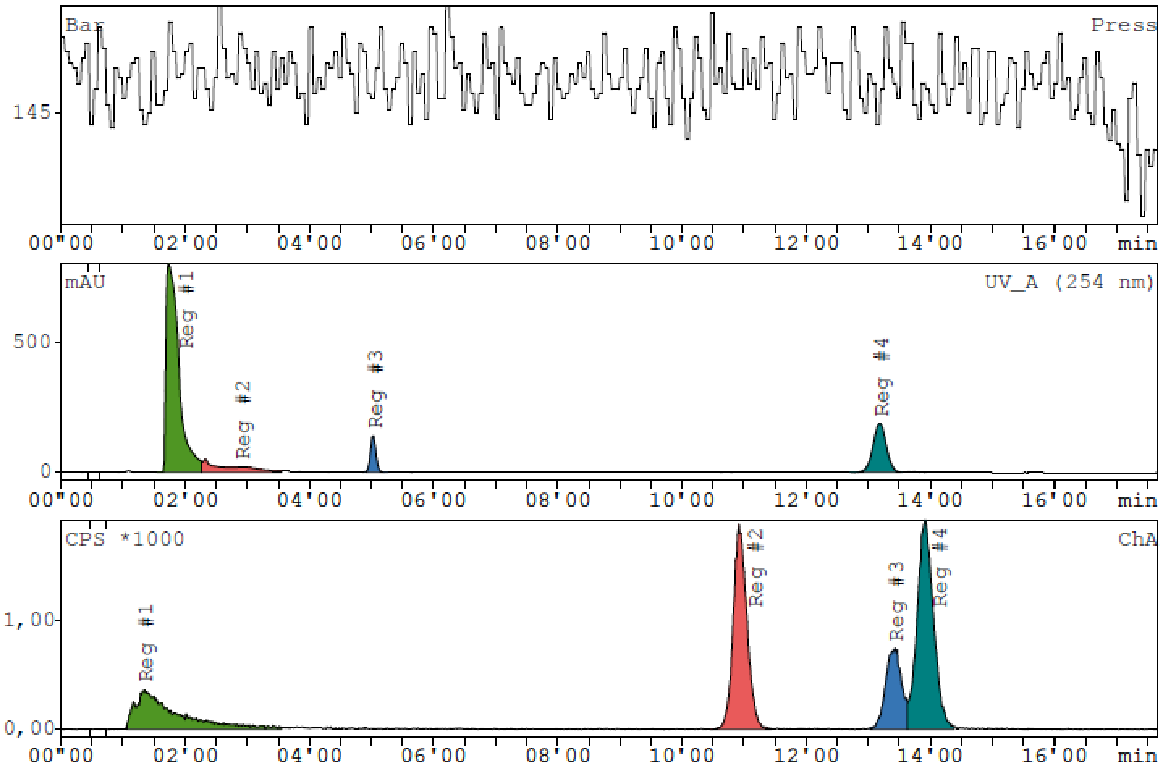

Measurement: 210721-A2, injection : 21.07.2021 13:25	
Method: LUNAPFP Mudasir AcN_H2O 50_50 from: 17.12.2020 09:30	
CH3CN:H2O=50:50, Flow rate 1,5 mL/min, injection 10 micro L	
Radio detector: raytest Gabi Star	Serial Nr.: #30745 raytest GINA star 20.04.09 Firmware V4.8
Software Version: 5.9, Service Pack 8, Build 5076	

ChA

Substance	R/T s	Type	Area Counts	%Area %
Reg #1	01'21	DD(M	16479,51	17,73
Reg #2	10'56	DD(M	28365,92	30,52
Reg #3	13'26	DD(M	13068,95	14,06
Reg #4	13'55	DD(M	35029,07	37,69
Sum in ROI			92943,45	100,00
Area (total)			95953,58	
BKG1			2,133	
Remainder			3010,13	3,14

Figure S85: Analytical HPLC chromatogram from screening, top channel = UV, bottom channel = activity. HPLC spectrum of 2,2-difluoro-2-(fluoro-^{18}F)ethyl 4-methylbenzenesulfonate **([^{18}F]1b).** KH_2PO_4 in the eluent for [^{18}F], **1a**, TEMPO, Et_3SiH, DMSO, 90°C, 2-methyl-2-butanol, 6 min. 100 µL organic phase in 1 mL MeCN: H_2O =50:50. **(**MeCN: H_2O =50:50. Flow rate = 1.5 mL/min. Injected volume = 10 µL).

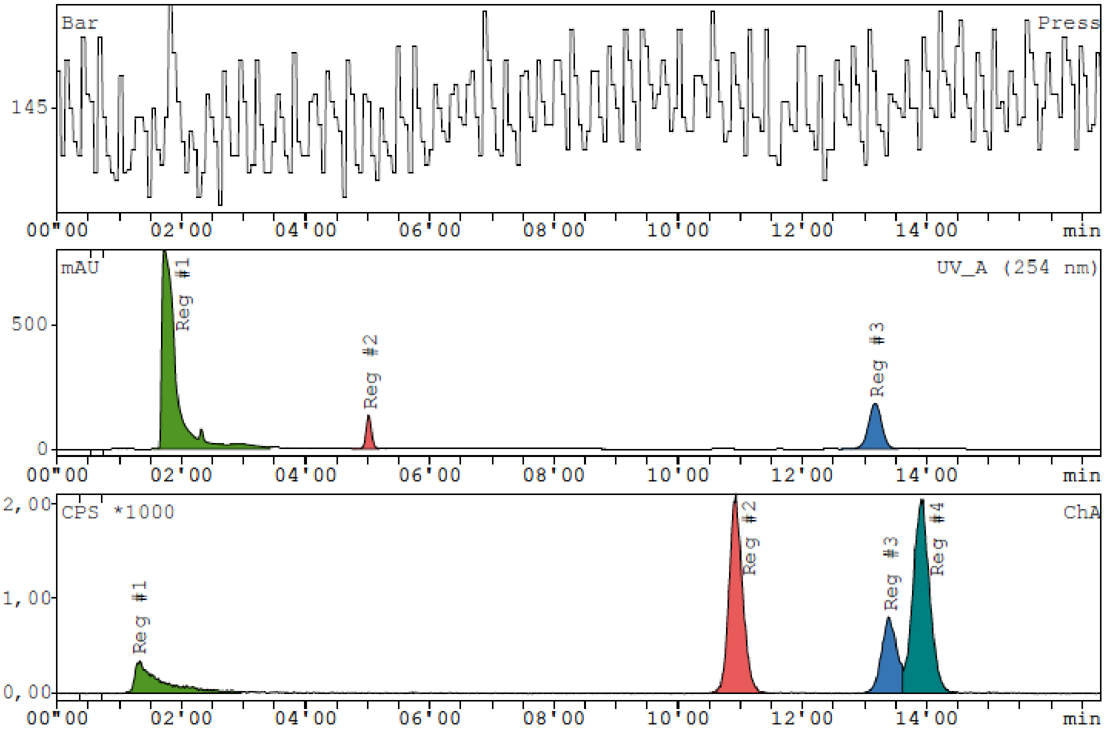

Measurement:	210721-B2, injection :	21.07.2021 14:04
Method:	LUNAPFP Mudasir AcN_H2O 50_50 from:	17.12.2020 09:30
CH3CN:H2O=50:50, Flow rate 1,5 mL/min, injection 10 micro L		
Radio detector: raytest Gabi Star	Serial Nr.: #30745 raytest GINA star 20.04.09 Firmware V4.8	
Software Version: 5.9, Service Pack 8, Build 5076		

ChA

**Substance**	**R/T s**	**Type**	**Area Counts**	**%Area %**
Reg #1	01'19	DD(M	9550,23	10,43
Reg #2	10'55	DD(M	30902,64	33,74
Reg #3	13'24	DD(M	13326,34	14,55
Reg #4	13'55	DD(M	37818,59	41,29
Sum in ROI			91597,80	100,00
Area (total)			93495,27	
BKG1			3,238	
Remainder			1897,47	2,03

Figure S86: Analytical HPLC chromatogram from screening, top channel = UV, bottom channel = activity. HPLC spectrum of 2,2-difluoro-2-(fluoro-^{18}F)ethyl 4-methylbenzenesulfonate **([^{18}F]1b).** KOTf in the eluent for [^{18}F], **1a**, TEMPO, Et_3SiH, DMSO, 90°C, 2-methyl-2-butanol, 6 min. 100 µL organic phase in 1 mL MeCN: H_2O =50:50. **(**MeCN: H_2O =50:50. Flow rate = 1.5 mL/min. Injected volume = 10 µL).

Measurement 210721-C2 Uni Oslo Page 1/1
c:\GINA_NT\LUNAPFP Mudasir AcN_H2O 50_50\210721-C2

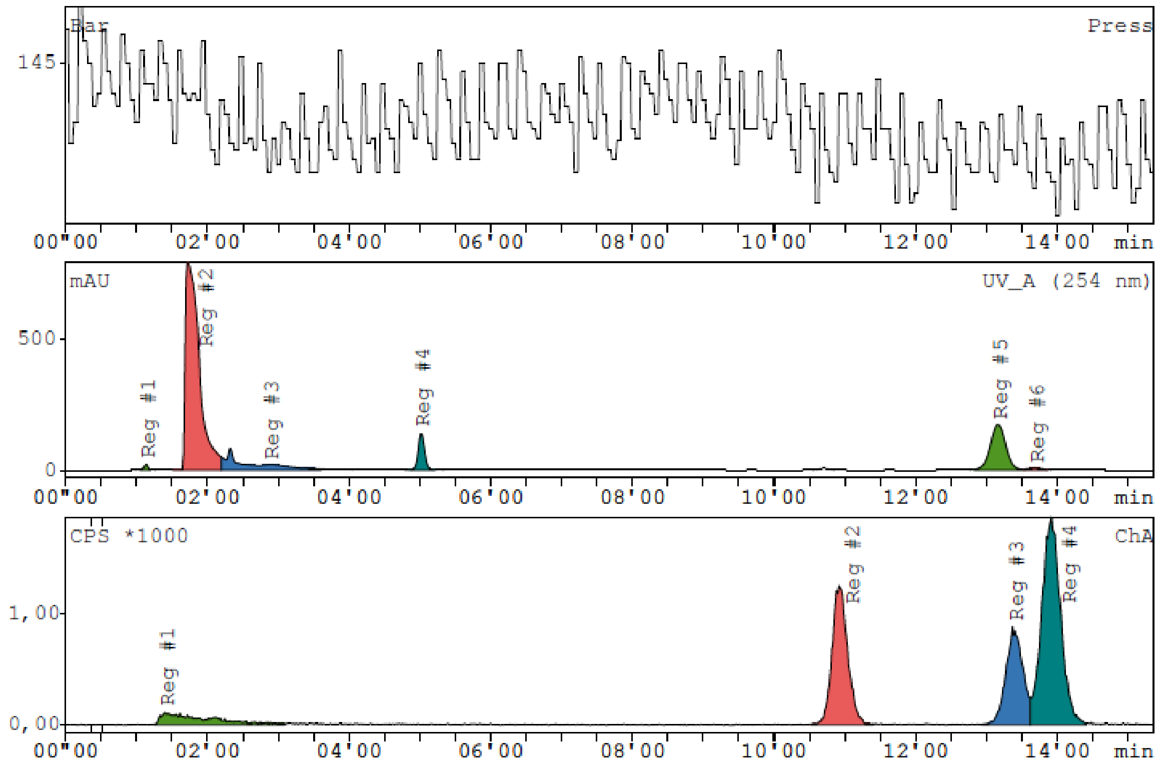

Measurement:	210721-C2, injection :	21.07.2021 14:43	
Method:	LUNAPFP Mudasir AcN_H2O 50_50 from:		17.12.2020 09:30

CH3CN:H2O=50:50, Flow rate 1,5 mL/min, injection 10 micro L
Radio detector: raytest Gabi Star Serial Nr.: #30745 raytest GINA star 20.04.09 Firmware V4.8
Software Version: 5.9, Service Pack 8, Build 5076

ChA

Substance	R/T s	Type	Area Counts	%Area %
Reg #1	01'25	DD(M	4744,97	6,56
Reg #2	10'55	DD(M	18936,66	26,17
Reg #3	13'24	DD(M	14187,25	19,61
Reg #4	13'55	DD(M	34483,51	47,66
Sum in ROI			72352,39	100,00
Area (total)			73865,31	
BKG1			2,778	
Remainder			1512,92	2,05

Figure S87: Analytical HPLC chromatogram from screening, top channel = UV, bottom channel = activity. HPLC spectrum of 2,2-difluoro-2-(fluoro-^{18}F)ethyl 4-methylbenzenesulfonate **([^{18}F]1b).** NaOTf in the eluent for [^{18}F], **1a**, TEMPO, Et_3SiH, DMSO, 90°C, 2-methyl-2-butanol, 6 min. 100 µL organic phase in 1 mL MeCN: H_2O =50:50. **(**MeCN: H_2O =50:50. Flow rate = 1.5 mL/min. Injected volume = 10 µL).

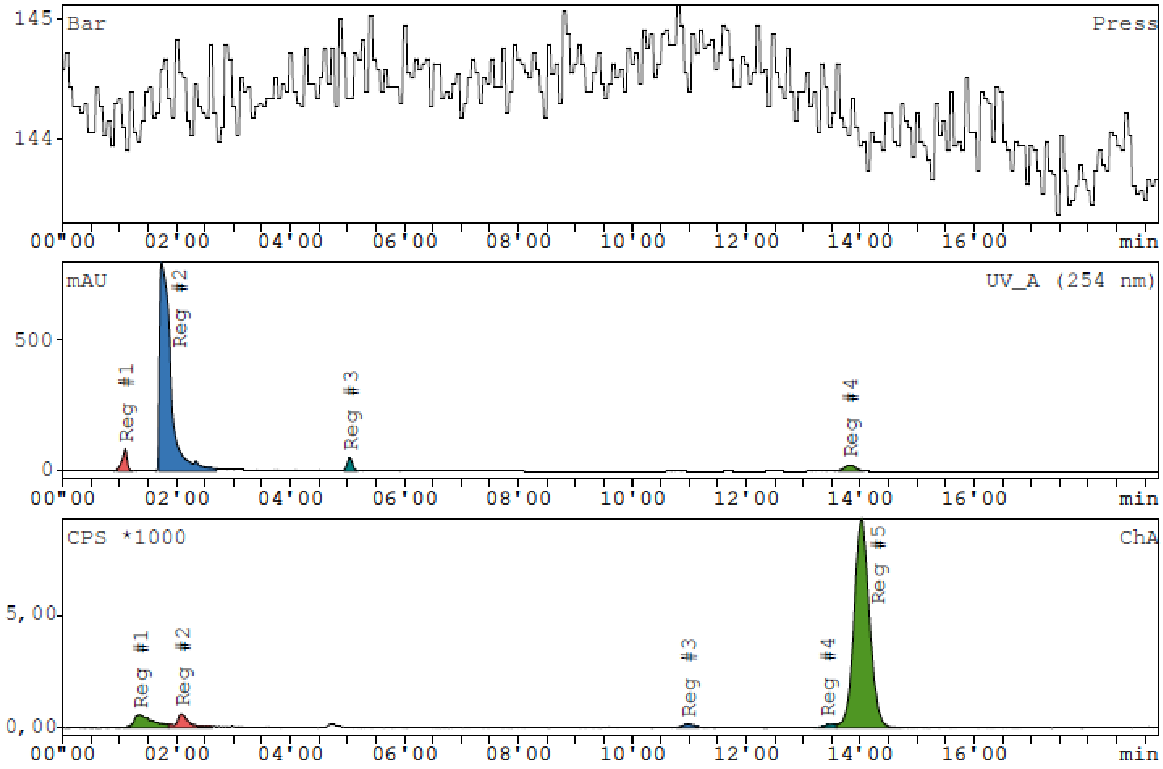

Sample description

Measurement:	090621-H, injection :	09.06.2021 14:11	
Method:	LUNAPFP Mudasir AcN_H2O 50_50 from:		17.12.2020 09:30
CH3CN:H2O=50:50, Flow rate 1,5 mL/min, injection 10 micro L			
Radio detector: raytest Gabi Star	Serial Nr.: #30745 raytest GINA star 20.04.09 Firmware V4.8		
Software Version: 5.9, Service Pack 8, Build 5076			

Integration ChA

Substance	R/T s	Type	Area Counts	%Area %
Reg #1	01'21	DD(M	12934,9	6,52
Reg #2	02'06	DD(M	9178,2	4,63
Reg #3	10'58	DD(M	2313,5	1,17
Reg #4	13'25	DD(M	1913,4	0,97
Reg #5	14'01	DD(M	171904,7	86,71
Sum in ROI			198244,6	100,00
Area (total)			210831,3	
Ext. BKG			0,00 CPS	

Figure S88: Analytical HPLC chromatogram from screening, top channel = UV, bottom channel = activity. HPLC spectrum of 2,2-difluoro-2-(fluoro-^{18}F)ethyl 4-methylbenzenesulfonate **([^{18}F]1b). 1a**, TEMPO, Et_3SiH, DMSO, 90°C, 2-methyl-2-butanol (0.1 mL), 6 min. 100 µL organic phase in 1 mL MeCN: H_2O =50:50. **(**MeCN: H_2O =50:50. Flow rate = 1.5 mL/min. Injected volume = 10 µL).

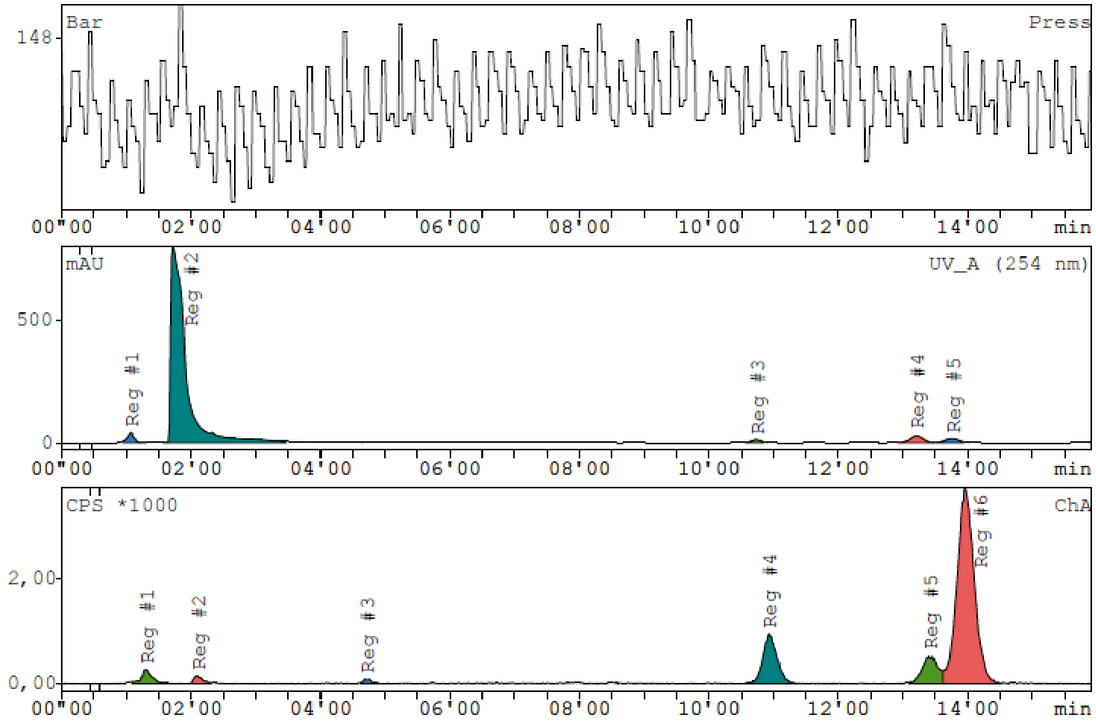

Measurement:	310821-A, injection : 31.08.2021 11:56
Method:	LUNAPFP Mudasir AcN_H2O 50_50 from: 17.12.2020 09:30
CH3CN:H2O=50:50, Flow rate 1,5 mL/min, injection 10 micro L	
Radio detector: raytest Gabi Star	Serial Nr.: #30745 raytest GINA star 20.04.09 Firmware V4.8
Software Version: 5.9, Service Pack 8, Build 5076	

ChA

**Substance**	**R/T s**	**Type**	**Area Counts**	**%Area %**
Reg #1	01'18	DD(M	3061,46	3,16
Reg #2	02'06	DD(M	1427,72	1,47
Reg #3	04'43	DD(M	790,88	0,82
Reg #4	10'56	DD(M	14128,05	14,58
Reg #5	13'25	DD(M	8703,20	8,98
Reg #6	13'58	DD(M	68771,35	70,98
Sum in ROI			96882,66	100,00
Area (total)			101630,42	
BKG1			7,889	
Remainder			4747,76	4,67

Figure S89: Analytical HPLC chromatogram from screening, top channel = UV, bottom channel = activity. HPLC spectrum of 2,2-difluoro-2-(fluoro-^{18}F)ethyl 4-methylbenzenesulfonate **([^{18}F]1b). 1a**, TEMPO, Et_3SiH, DMSO, 90°C, 2-methyl-2-butanol (0.2 mL), 6 min. 100 µL organic phase in 1 mL MeCN: H_2O =50:50. **(**MeCN: H_2O =50:50. Flow rate = 1.5 mL/min. Injected volume = 10 µL).

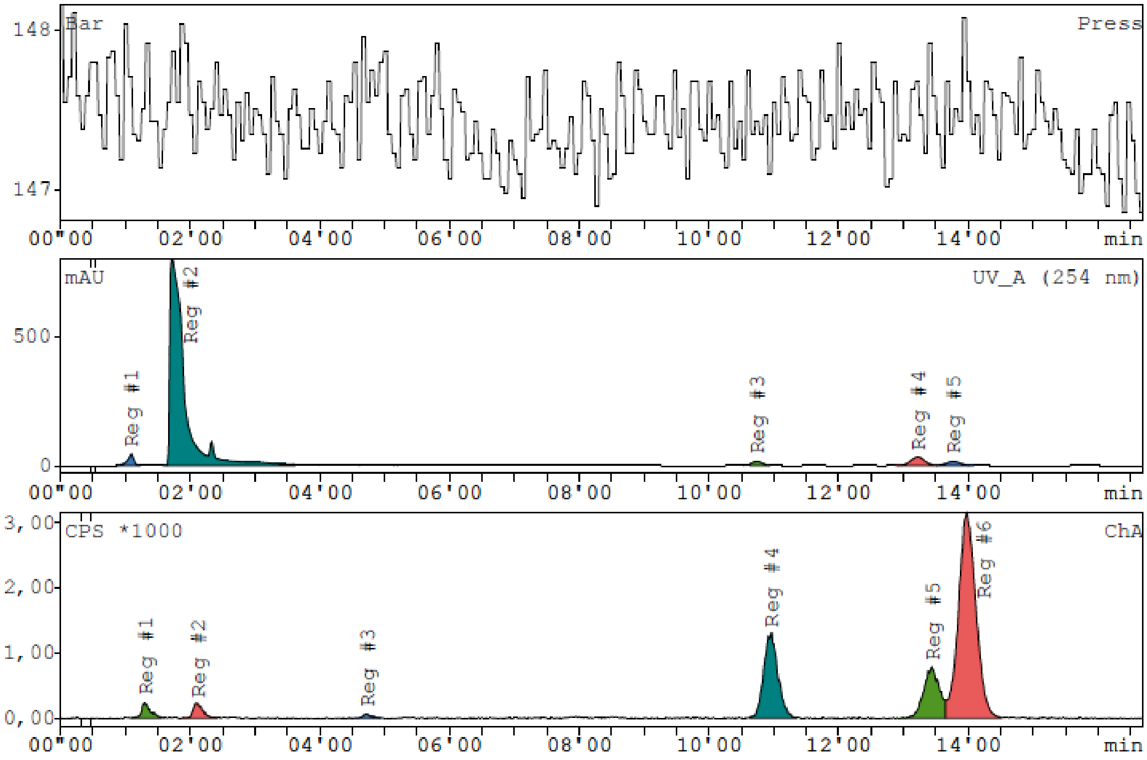

Measurement:	310821-F, injection : 31.08.2021 14:19
Method:	LUNAPFP Mudasir AcN_H2O 50_50 from: 17.12.2020 09:30
CH3CN:H2O=50:50, Flow rate 1,5 mL/min, injection 10 micro L	
Radio detector: raytest Gabi Star	Serial Nr.: #30745 raytest GINA star 20.04.09 Firmware V4.8
Software Version: 5.9, Service Pack 8, Build 5076	

ChA

Substance	R/T s	Type	Area Counts	%Area %
Reg #1	01'18	DD(M	2301,07	2,39
Reg #2	02'06	DD(M	2464,93	2,56
Reg #3	04'43	DD(M	661,96	0,69
Reg #4	10'56	DD(M	19619,09	20,34
Reg #5	13'26	DD(M	12935,05	13,41
Reg #6	13'58	DD(M	58471,59	60,62
Sum in ROI			96453,69	100,00
Area (total)			100414,80	
BKG1			12,556	
Remainder			3961,11	3,94

Figure S90: Analytical HPLC chromatogram from screening, top channel = UV, bottom channel = activity. HPLC spectrum of 2,2-difluoro-2-(fluoro-^{18}F)ethyl 4-methylbenzenesulfonate **([^{18}F]1b). 1a**, TEMPO, Et_3SiH, DMSO, 90°C, 2-methyl-2-butanol (0.3 mL), 6 min. 100 µL organic phase in 1 mL MeCN: H_2O =50:50. **(**MeCN: H_2O =50:50. Flow rate = 1.5 mL/min. Injected volume = 10 µL).

Measurement 310821-G Uni Oslo Page 1/1
c:\GINA_NT\LUNAPFP Mudasir AcN_H2O 50_50\310821-G

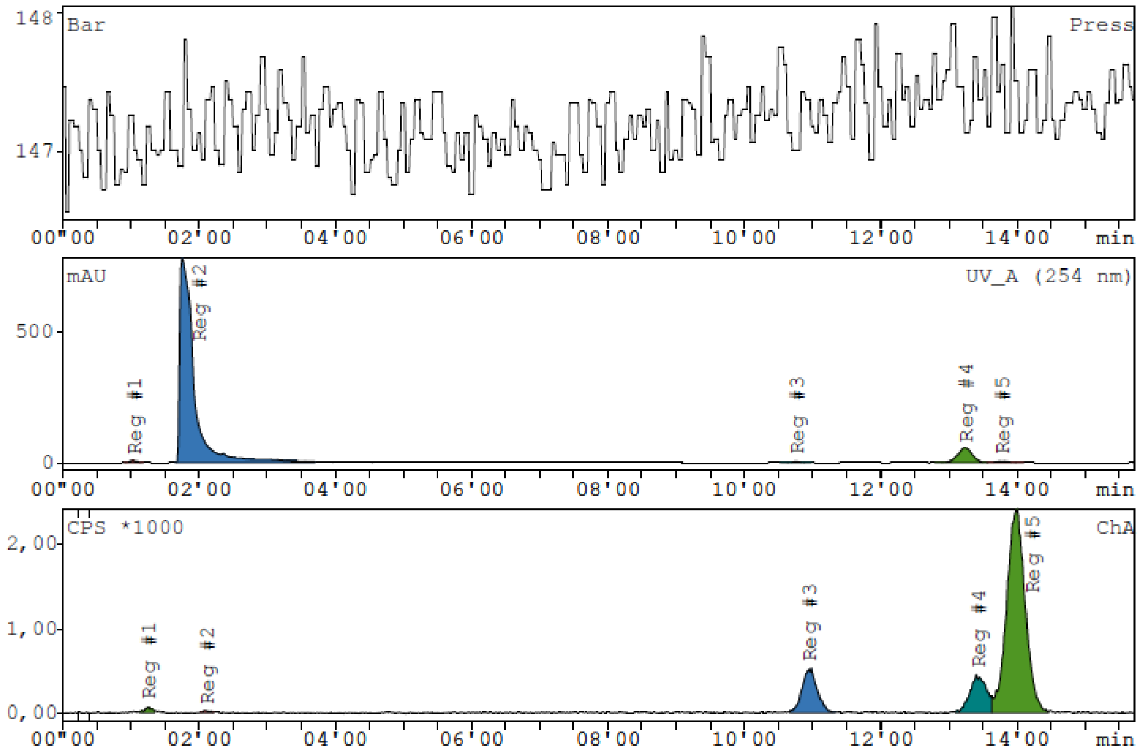

Measurement:	310821-G, injection : 31.08.2021 14:36
Method:	LUNAPFP Mudasir AcN_H2O 50_50 from: 17.12.2020 09:30
CH3CN:H2O=50:50, Flow rate 1,5 mL/min, injection 10 micro L	
Radio detector: raytest Gabi Star	Serial Nr.: #30745 raytest GINA star 20.04.09 Firmware V4.8
Software Version: 5.9, Service Pack 8, Build 5076	

ChA

Substance	R/T s	Type	Area Counts	%Area %
Reg #1	01'15	DD(M	493,26	0,81
Reg #2	02'06	DD(M	177,81	0,29
Reg #3	10'57	DD(M	7959,92	12,99
Reg #4	13'26	DD(M	7367,92	12,03
Reg #5	13'59	DD(M	45268,29	73,89
Sum in ROI			61267,20	100,00
Area (total)			62551,04	
BKG1			11,600	
Remainder			1283,84	2,05

Figure S91: Analytical HPLC chromatogram from screening, top channel = UV, bottom channel = activity. HPLC spectrum of 2,2-difluoro-2-(fluoro-^{18}F)ethyl 4-methylbenzenesulfonate **([^{18}F]1b). 1a**, TEMPO, Et_3SiH, DMSO, 90°C, 2-methyl-2-butanol (0.4 mL), 6 min. 100 µL organic phase in 1 mL MeCN: H_2O =50:50. **(**MeCN: H_2O =50:50. Flow rate = 1.5 mL/min. Injected volume = 10 µL).

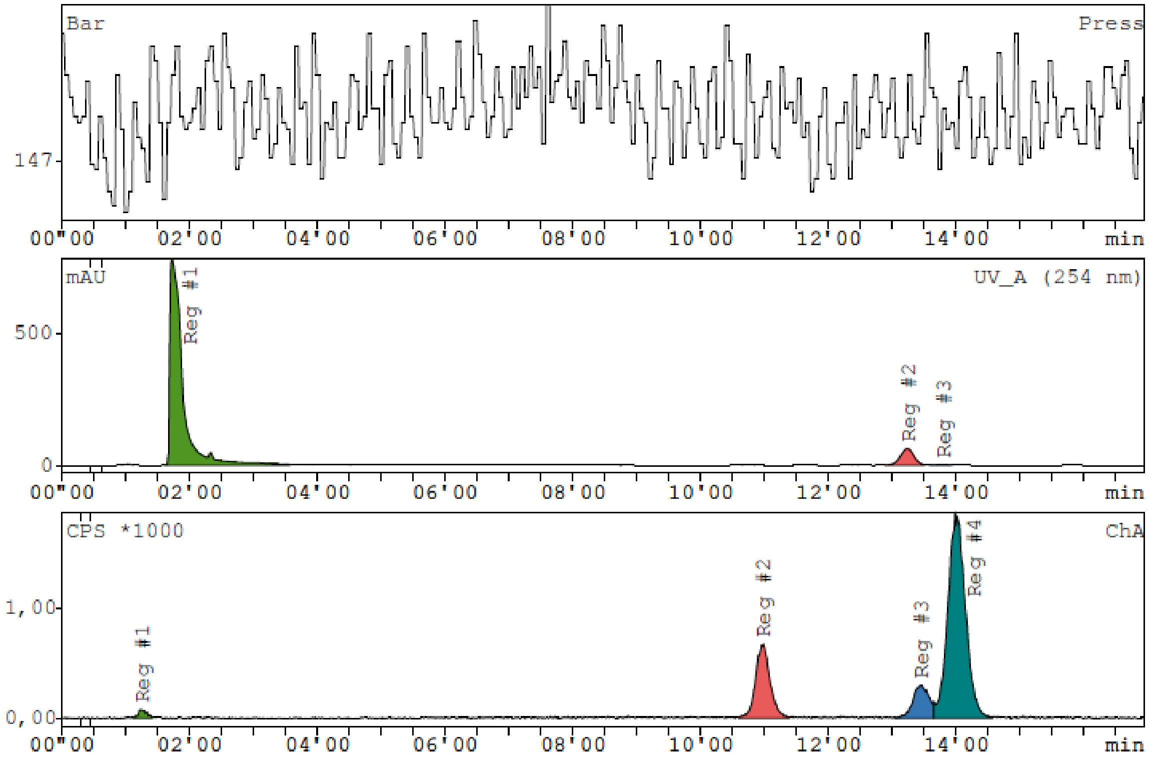

Measurement:	310821-H, injection : 31.08.2021 14:56
Method:	LUNAPFP Mudasir AcN_H2O 50_50 from: 17.12.2020 09:30
CH3CN:H2O=50:50, Flow rate 1,5 mL/min, injection 10 micro L	
Radio detector: raytest Gabi Star	Serial Nr.: #30745 raytest GINA star 20.04.09 Firmware V4.8
Software Version: 5.9, Service Pack 8, Build 5076	

ChA

Substance	R/T s	Type	Area Counts	%Area %
Reg #1	01'15	DD(M	685,08	1,33
Reg #2	10'58	DD(M	10436,31	20,24
Reg #3	13'26	DD(M	5155,65	10,00
Reg #4	14'01	DD(M	35289,03	68,43
Sum in ROI			51566,07	100,00
Area (total)			55270,53	
BKG1			7,333	
Remainder			3704,46	6,70

Figure S92: Analytical HPLC chromatogram from screening, top channel = UV, bottom channel = activity. HPLC spectrum of 2,2-difluoro-2-(fluoro-^{18}F)ethyl 4-methylbenzenesulfonate **([^{18}F]1b). 1a**, TEMPO, Et_3SiH, DMSO, 90°C, 2-methyl-2-butanol (0.5 mL), 6 min. 100 µL organic phase in 1 mL MeCN: H_2O =50:50. **(**MeCN: H_2O =50:50. Flow rate = 1.5 mL/min. Injected volume = 10 µL).

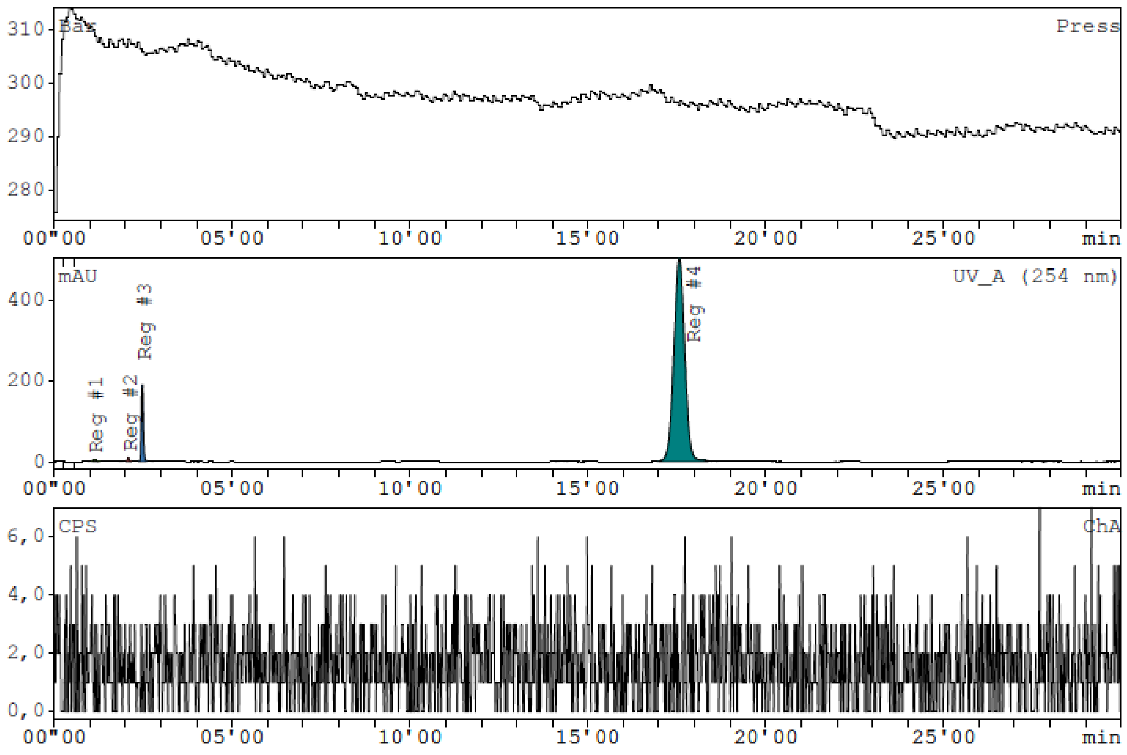

Measurement:	220222-6a, injection : 22.02.2022 11:31
Method:	LUNAPFP Mudasir AcN_H2O 50_50 from: 17.12.2020 09:30
MeCN:H2O =50:50. Flow rate = 1,5 mL/min. Injected volume = 10 uL.	
Radio detector: raytest Gabi Star	Serial Nr.: #30745 raytest GINA star 20.04.09 Firmware V4.8
Software Version: 5.9, Service Pack 8, Build 5076	

UV_A (254 nm)

Substance	R/T s	Type	Area mAU*s	%Area %
Reg #1	01'09	DD(M	35,45	0,31
Reg #2	02'05	DD(M	37,63	0,33
Reg #3	02'29	DD(M	668,19	5,86
Reg #4	17'35	DD(M	10664,18	93,50
Sum in ROI			11405,45	100,00
Area (total)			11581,31	
BKG1			0,046	

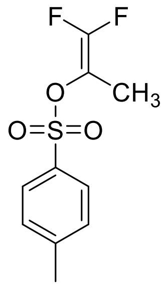

Figure S93: Analytical HPLC chromatogram from screening, top channel = UV, bottom channel = activity. HPLC spectrum of 1,1-difluoroprop-1-en-2-yl 4-methylbenzenesulfonate **(2a)**. 1 mg precursor dissolved in 1 mL MeCN: H_2O =50:50. **(**MeCN: H_2O =50:50. Flow rate = 1.5 mL/min. Injected volume = 10 uL).

Measurement 090621-G Uni Oslo Page 1/1
c:\GINA_NT\LUNAPFP Mudasir AcN_H2O 50_50\090621-G

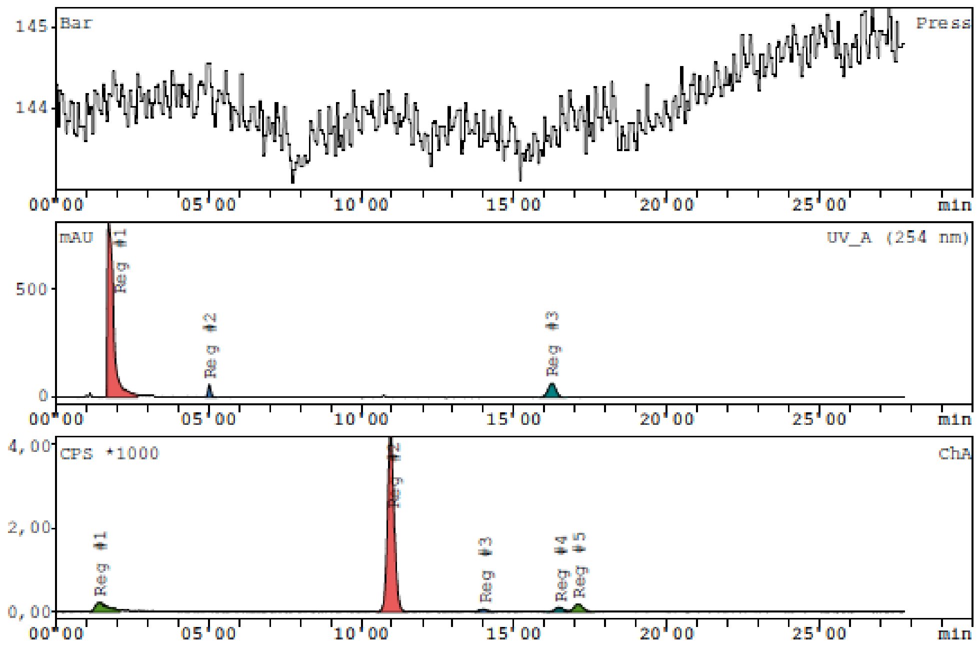

Sample description

Measurement:	090621-G, injection : 09.06.2021 14:53
Method:	LUNAPFP Mudasir AcN_H2O 50_50 from: 17.12.2020 09:30
CH3CN:H2O=50:50, Flow rate 1,5 mL/min, injection 10 micro L	
Radio detector: raytest Gabi Star	Serial Nr.: #30745 raytest GINA star 20.04.09 Firmware V4.8
Software Version: 5.9, Service Pack 8, Build 5076	

Integration ChA

Substance	R/T s	Type	Area Counts	%Area %
Reg #1	01'26	DD(M	6566,54	8,50
Reg #2	10'58	DD(M	63326,86	81,97
Reg #3	14'02	DD(M	1316,10	1,70
Reg #4	16'30	DD(M	2017,24	2,61
Reg #5	17'05	DD(M	4027,80	5,21
Sum in ROI			77254,55	100,00
Area (total)			85745,74	
Ext. BKG			0,00 CPS	

Integration UV_A (254 nm)

Substance	R/T s	Type	Area mAU*s	%Area %
Reg #1	01'44	DD(M	11308,46	88,14
Reg #2	05'02	DD(M	375,37	2,93
Reg #3	16'15	DD(M	1145,75	8,93
Sum in ROI			12829,58	100,00

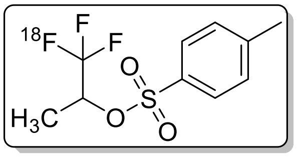

Figure S94: Analytical HPLC chromatogram from screening, top channel = UV, bottom channel = activity. HPLC spectrum of 1,1-difluoro-1-(fluoro-^{18}F)propan-2-yl 4-methylbenzenesulfonate **([^{18}F]2b).** 100 µL organic phase in 1 mL MeCN: H_2O =50:50. **(**MeCN: H_2O =50:50. Flow rate = 1.5 mL/min. Injected volume = 10 uL).

Measurement 060721-J Uni Oslo Page 1/2
c:\GINA_NT\LUNAPFP Mudasir AcN_H2O 50_50\060721-J

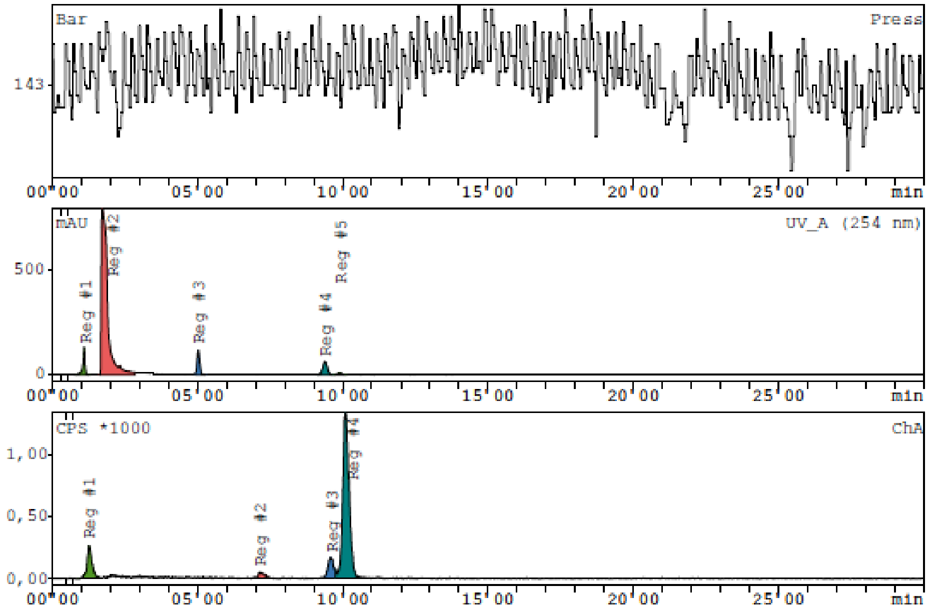

Measurement:	060721-J, injection : 06.07.2021 15:28
Method:	LUNAPFP Mudasir AcN_H2O 50_50 from: 17.12.2020 09:30
CH3CN:H2O=50:50, Flow rate 1,5 mL/min, injection 10 micro L	
Radio detector: raytest Gabi Star	Serial Nr.: #30745 raytest GINA star 20.04.09 Firmware V4.8
Software Version: 5.9, Service Pack 8, Build 5076	

ChA

Substance	R/T s	Type	Area Counts	%Area %
Reg #1	01'16	DD(M	3009,54	11,53
Reg #2	07'10	DD(M	681,47	2,61
Reg #3	09'36	DD(M	2185,74	8,37
Reg #4	10'06	DD(M	20236,19	77,49
Sum in ROI			26112,94	100,00
Area (total)			30081,74	
BKG1			2,286	
Remainder			3968,80	13,19

UV_A (254 nm)

Substance	R/T s	Type	Area mAU*s	%Area %
Reg #1	01'06	DD(M	632,65	4,60
Reg #2	01'45	DD(M	11625,31	84,56
Reg #3	05'02	DD(M	726,32	5,28
Reg #4	09'23	DD(M	671,93	4,89
Reg #5	09'54	DD(M	92,54	0,67
Sum in ROI			13748,75	100,00
Area (total)			14542,58	
BKG1			-0,060	

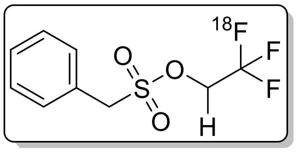

Figure S95: Analytical HPLC chromatogram from screening, top channel = UV, bottom channel = activity. HPLC spectrum of 2,2-difluoro-2-(fluoro-^{18}F)ethyl phenylmethanesulfonate **([^{18}F]3b)**. 100 µL organic phase in 1 mL MeCN: H_2O =50:50. **(**MeCN: H_2O =50:50. Flow rate = 1.5 mL/min. Injected volume = 10 uL).

Measurement 250122-benzyl-sul-CF2-CF3 Uni Oslo Page 1/1

c:\GINA_NT\LUNAPFP Mudasir AcN_H2O 50_50\250122-benzyl-sul-CF2-CF3

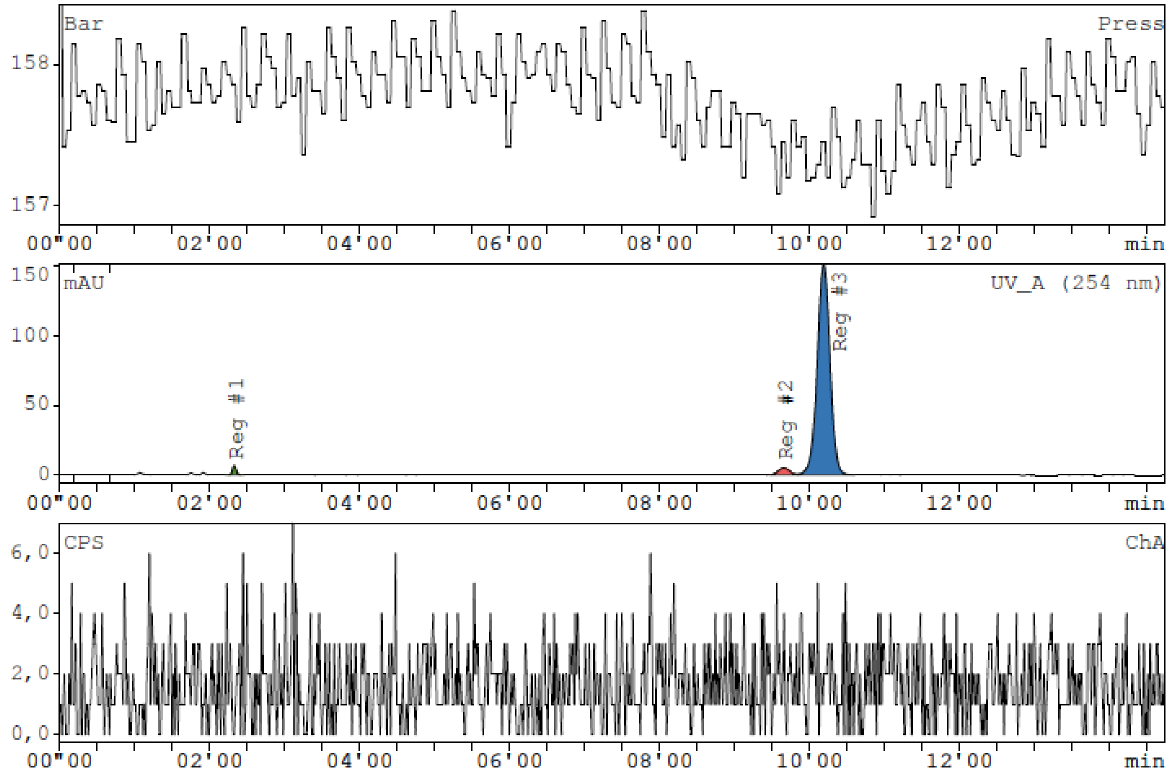

Measurement:	250122-benzyl-sul-CF2-CF3, injection : 25.01.2022 14:55
Method:	LUNAPFP Mudasir AcN_H2O 50_50 from: 17.12.2020 09:30
CH3CN:H2O=50:50, Flow rate 1,5 mL/min, injection 10 micro L	
Radio detector: raytest Gabi Star Serial Nr.: #30745 raytest GINA star 20.04.09 Firmware V4.8	
Software Version: 5.9, Service Pack 8, Build 5076	

UV_A (254 nm)

Substance	R/T s	Type	Area mAU*s	%Area %
Reg #1	02'20	DD(M	22,746	1,23
Reg #2	09'40	DD(M	52,948	2,87
Reg #3	10'12	DD(M	1769,838	95,90
Sum in ROI			1845,532	100,00
Area (total)			1782,581	
BKG1			-0,0167	

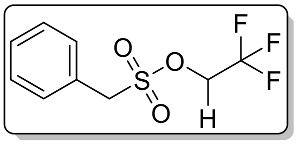

Figure S96: Analytical HPLC chromatogram from screening, top channel = UV, bottom channel = activity. HPLC spectrum of 2,2,2-trifluoroethyl phenylmethanesulfonate **(3b)**. 1 mg precursor dissolved in 1 mL MeCN: H_2O=50:50. (MeCN: H_2O=50:50. Flow rate = 1.5 mL/min. Injected volume = 10 uL).

Measurement 110821-P6 Uni Oslo Page 1/1

c:\GINA_NT\LUNAPFP Mudasir AcN_H2O 50_50\110821-P6

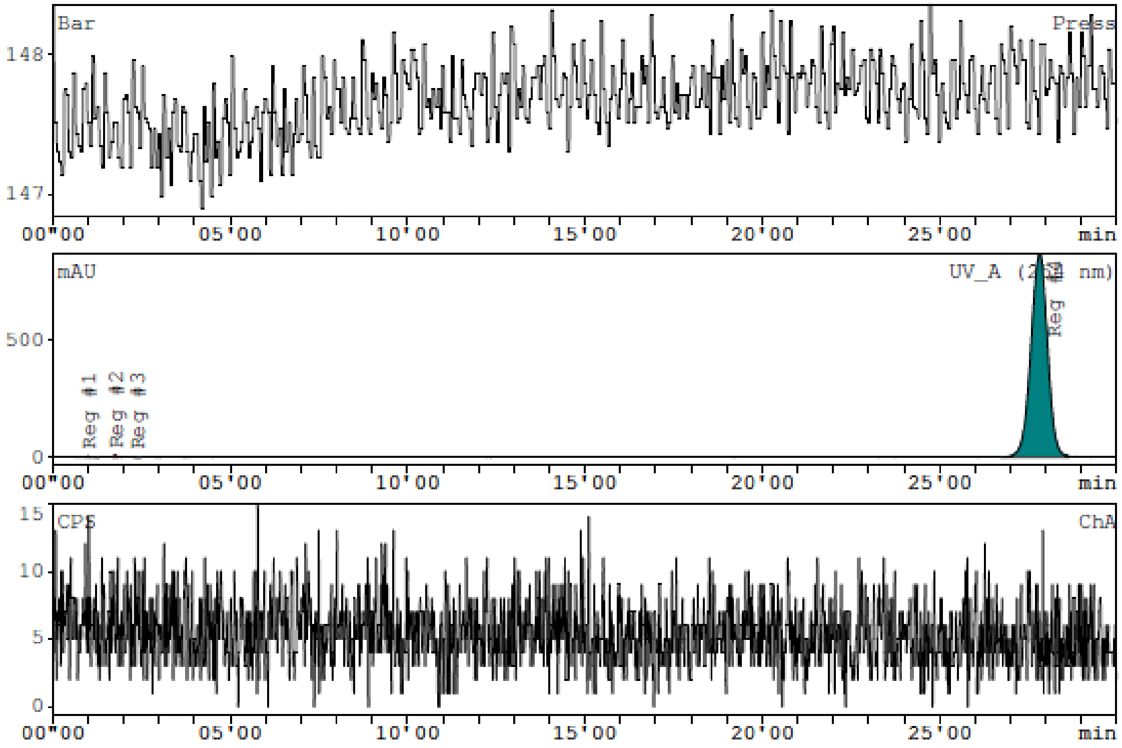

Measurement:	110821-P6, injection : 11.08.2021 17:13
Method:	LUNAPFP Mudasir AcN_H2O 50_50 from: 17.12.2020 09:30
CH3CN:H2O=50:50, Flow rate 1,5 mL/min, injection 10 micro L	
Radio detector: raytest Gabi Star	Serial Nr.: #30745 raytest GINA star 20.04.09 Firmware V4.8
Software Version: 5.9, Service Pack 8, Build 5076	

UV_A (254 nm)

Substance	R/T s	Type	Area mAU*s	%Area %
Reg #1	01'00	DD(M	37,64	0,13
Reg #2	01'46	DD(M	34,35	0,12
Reg #3	02'23	DD(M	7,30	0,03
Reg #4	27'49	DD(M	28128,09	99,72
Sum in ROI			28207,39	100,00

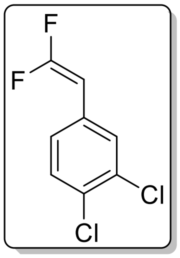

Figure S97: Analytical HPLC chromatogram from screening, top channel = UV, bottom channel = activity. HPLC spectrum of pure 1,2-dichloro-4-(2,2-difluorovinyl)benzene **(4a)**. 1 mg dissolved in 1 mL MeCN: H_2O =50:50. **(**MeCN: H_2O =50:50. Flow rate = 1.5 mL/min. Injected volume = 10 uL).

Measurement 210521-P6-repeat Uni Oslo Page 1/2
c:\GINA_NT\LUNAPFP Mudasir AcN_H2O 50_50\210521-P6-repeat

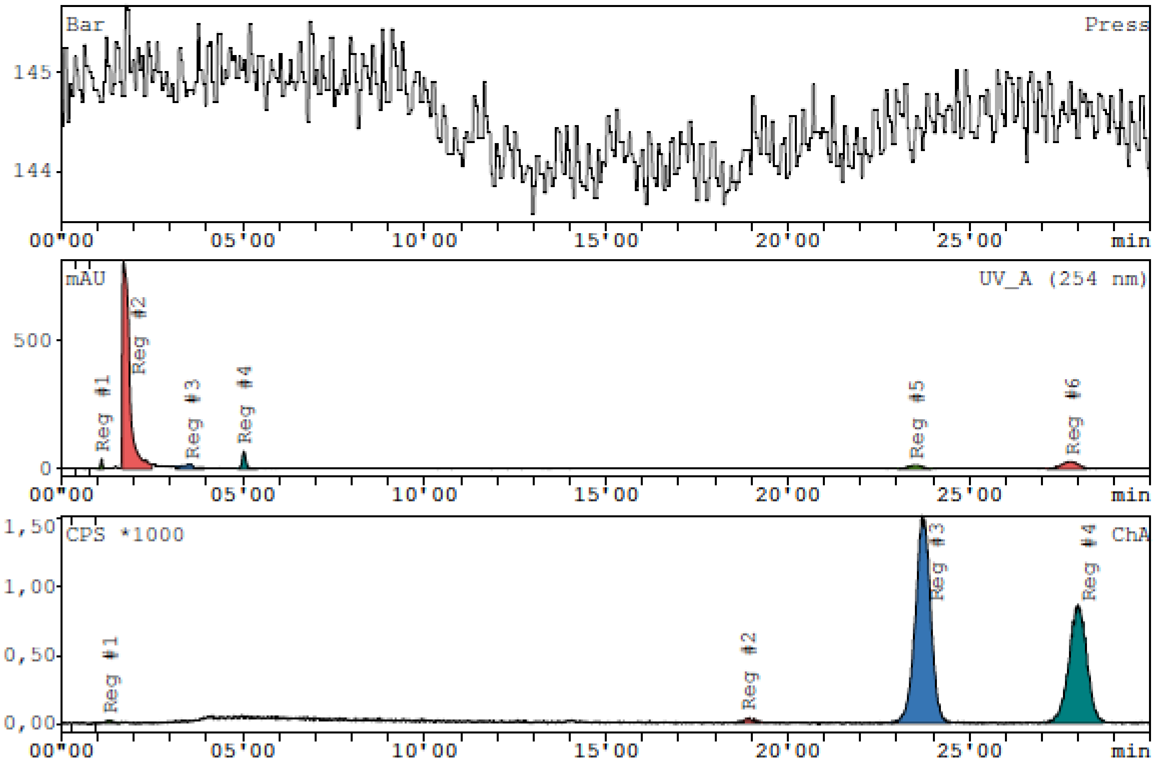

Sample description

Measurement:	210521-P6-repeat, injection :	21.05.2021 15:19	
Method:	LUNAPFP Mudasir AcN_H2O 50_50 from:		17.12.2020 09:30
CH3:CN= 50:50, Flow rate= 1,5 mL/min, Injection vol. 10 microL			
Radio detector: raytest Gabi Star	Serial Nr.: #30745 raytest GINA star 20.04.09 Firmware V4.8		
Software Version: 5.9, Service Pack 8, Build 5076			

Integration ChA

Substance	R/T s	Type	Area Counts	%Area %
Reg #1	01'18	DD(M	241,13	0,33
Reg #2	18'55	DD(M	699,14	0,95
Reg #3	23'44	DD(M	43730,57	59,15
Reg #4	28'00	DD(M	29258,64	39,58
Sum in ROI			73929,48	100,00
Area (total)			91771,57	
BKG1			5,951	
Remainder			17842,10	19,44

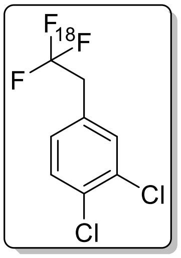

Figure S98: Analytical HPLC chromatogram from screening, top channel = UV, bottom channel = activity. HPLC spectrum of1,2-dichloro-4-(2,2-difluoro-2-(fluoro-^{18}F)ethyl)benzene **([^{18}F]4b)**. 100 µL organic phase in 1 mL MeCN: H_2O =50:50. **(**MeCN: H_2O =50:50. Flow rate = 1.5 mL/min. Injected volume = 10 uL).

Measurement 070621-P7-2 Uni Oslo Page 1/1
c:\GINA_NT\LUNAPFP Mudasir AcN_H2O 50_50\070621-P7-2

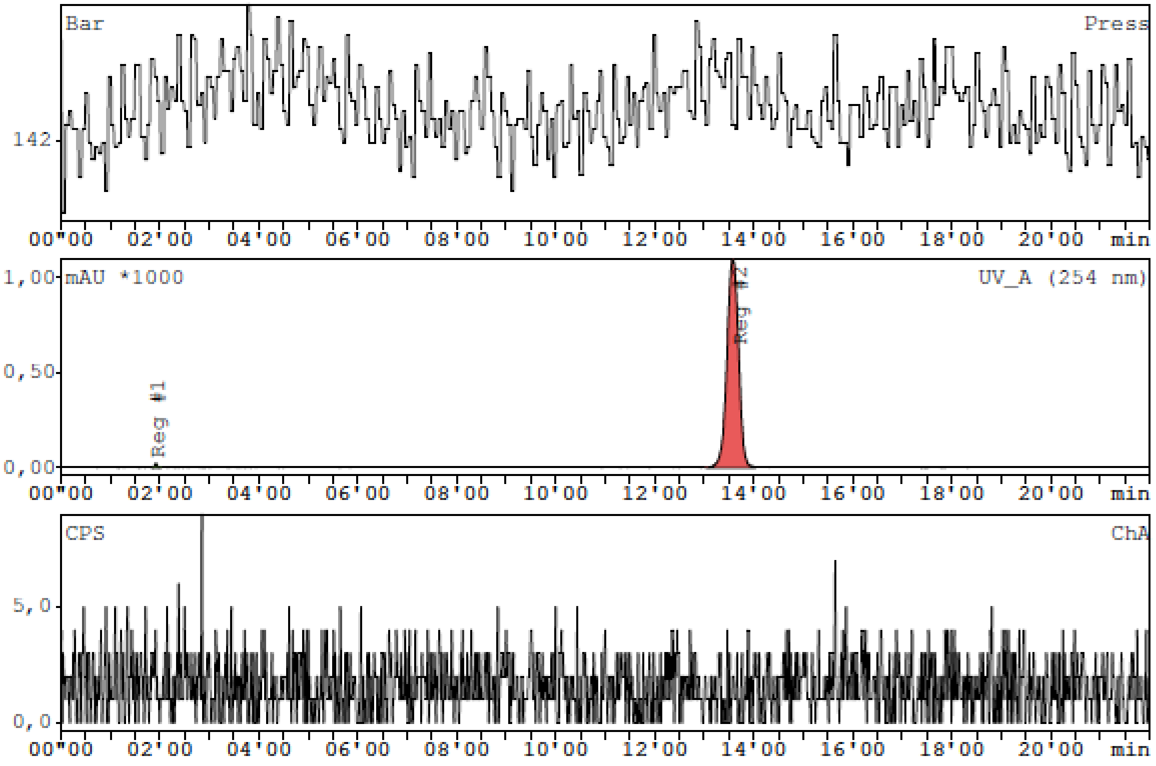

Measurement:	070621-P7-2, injection : 07.06.2021 16:34
Method:	LUNAPFP Mudasir AcN_H2O 50_50 from: 17.12.2020 09:30
CH3CN:H2O =50:50, Flow rate= 1,5 mL/min, injection vol. 10 microL	
Radio detector: raytest Gabi Star	Serial Nr.: #30745 raytest GINA star 20.04.09 Firmware V4.8
Software Version: 5.9, Service Pack 8, Build 5076	

UV_A (254 nm)

Substance	R/T s	Type	Area mAU*s	%Area %
Reg #1	01'56	DD(M	68,74	0,39
Reg #2	13'35	DD(M	17537,28	99,61
Sum in ROI			17606,03	100,00

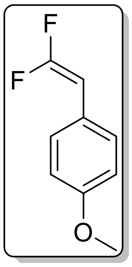

Figure S99: Analytical HPLC chromatogram from screening, top channel = UV, bottom channel = activity. HPLC spectrum of 1-(2,2-difluorovinyl)-4-methoxybenzene **(5a)**. 100 µL organic phase in 1 mL MeCN: H_2O =50:50. **(**MeCN: H_2O =50:50. Flow rate = 1.5 mL/min. Injected volume = 10 uL).

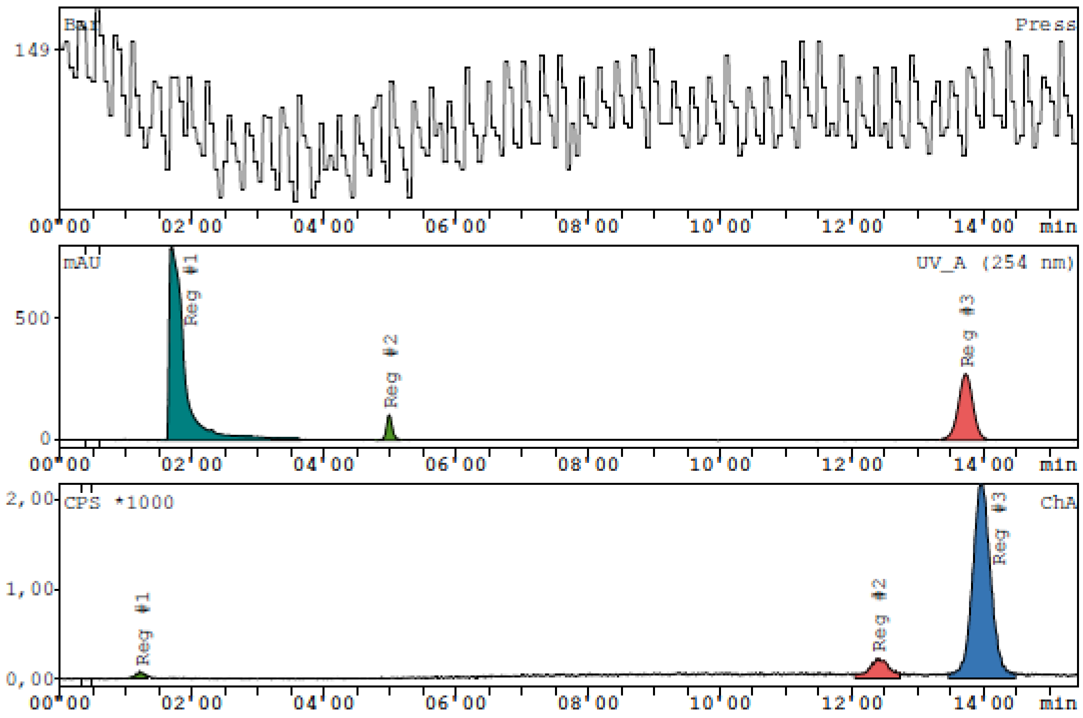

Measurement:	270821-C2, injection : 27.08.2021 14:41
Method:	LUNAPFP Mudasir AcN_H2O 50_50 from: 17.12.2020 09:30
CH3CN:H2O=50:50, Flow rate 1,5 mL/min, injection 10 micro L	
Radio detector: raytest Gabi Star	Serial Nr.: #30745 raytest GINA star 20.04.09 Firmware V4.8
Software Version: 5.9, Service Pack 8, Build 5076	

ChA

Substance	R/T s	Type	Area Counts	%Area %
Reg #1	01'14	DD(M	629,28	1,33
Reg #2	12'24	DD(M	4789,67	10,16
Reg #3	13'58	DD(M	41734,47	88,51
Sum in ROI			47153,42	100,00
Area (total)			68177,61	
BKG1			5,111	
Remainder			21024,19	30,84

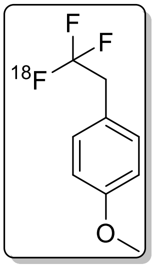

Figure S100: Analytical HPLC chromatogram from screening, top channel = UV, bottom channel = activity. HPLC spectrum of 1-(2,2-difluoro-2-(fluoro-^{18}F)ethyl)-4-methoxybenzene **([^{18}F]5b)**. 100 µL organic phase in 1 mL MeCN: H_2O =50:50. **(**MeCN: H_2O =50:50. Flow rate = 1.5 mL/min. Injected volume = 10 uL).

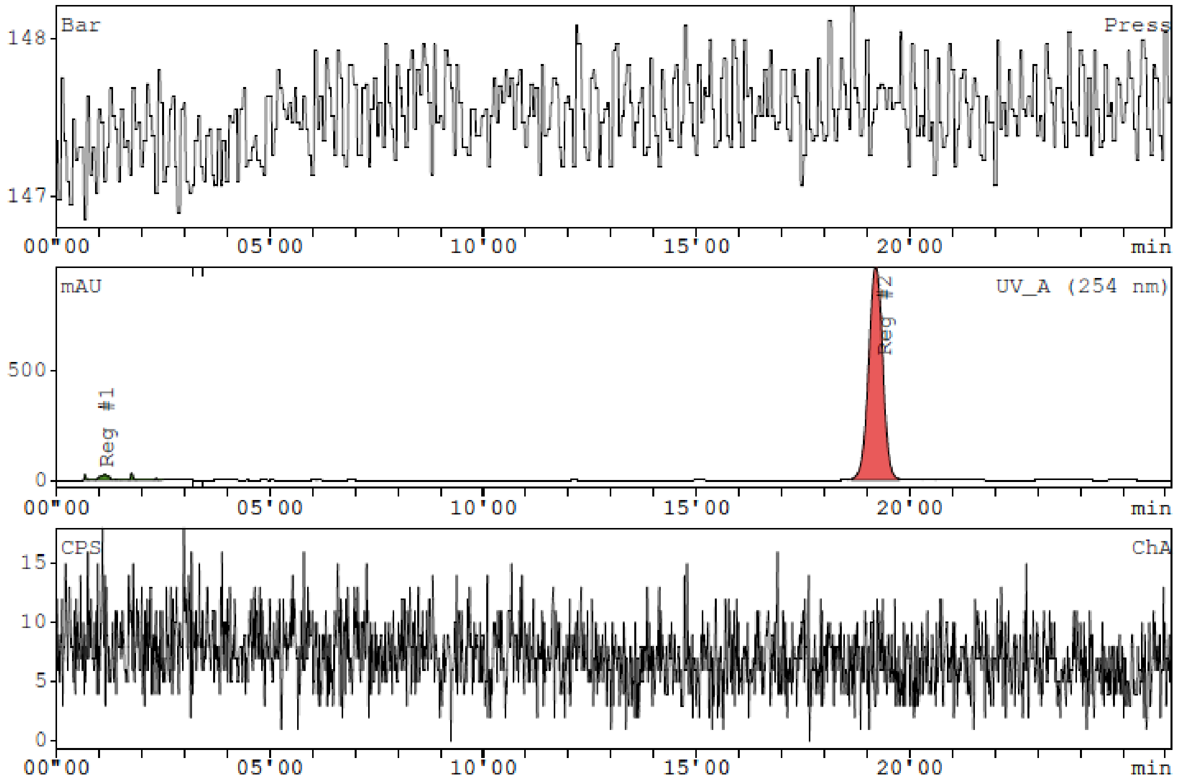

Measurement:	110821-P8, injection : 11.08.2021 16:45
Method:	LUNAPFP Mudasir AcN_H2O 50_50 from: 17.12.2020 09:30
CH3CN:H2O=50:50, Flow rate 1,5 mL/min, injection 10 micro L	
Radio detector: raytest Gabi Star	Serial Nr.: #30745 raytest GINA star 20.04.09 Firmware V4.8
Software Version: 5.9, Service Pack 8, Build 5076	

UV_A (254 nm)

Substance	R/T s	Type	Area mAU*s	%Area %
Reg #1	01'08	DD(M	608,24	2,67
Reg #2	19'12	DD(M	22132,47	97,33
Sum in ROI			22740,72	100,00
Area (total)			22909,73	
BKG1			0,151	

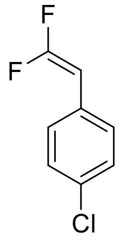

Figure S101: Analytical HPLC chromatogram from screening, top channel = UV, bottom channel = activity. HPLC spectrum of 1-chloro-4-(2,2-difluorovinyl)benzene **(6a)**. 1 mg precursor dissolved in 1 mL MeCN: H_2O =50:50. **(**MeCN: H_2O =50:50. Flow rate = 1.5 mL/min. Injected volume = 10 uL).

Measurement 270821-G Uni Oslo Page 1/1

c:\GINA_NT\LUNAPFP Mudasir AcN_H2O 50_50\270821-G

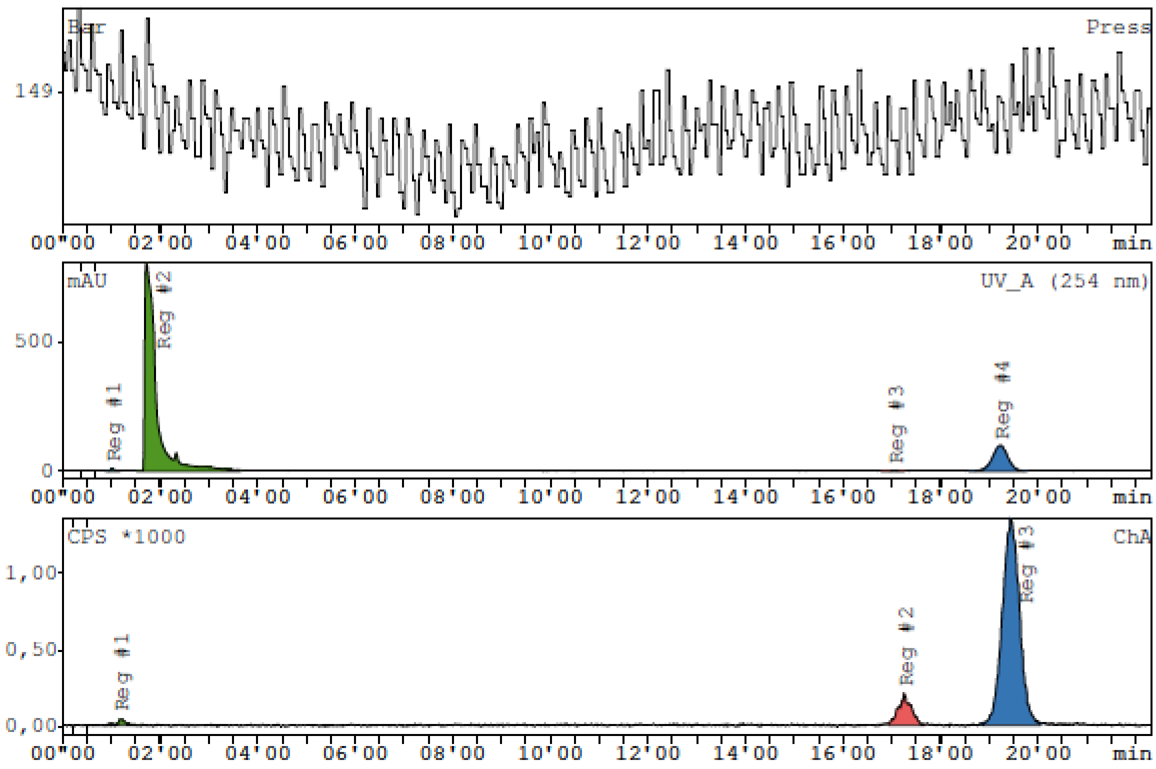

Measurement:	270821-G, injection :	27.08.2021 16:56	
Method:	LUNAPFP Mudasir AcN_H2O 50_50 from:		17.12.2020 09:30
CH3CN:H2O=50:50, Flow rate 1,5 mL/min, injection 10 micro L			
Radio detector: raytest Gabi Star	Serial Nr.: #30745 raytest GINA star 20.04.09 Firmware V4.8		
Software Version: 5.9, Service Pack 8, Build 5076			

ChA

Substance	R/T s	Type	Area Counts	%Area %
Reg #1	01'12	DD(M	419,97	1,15
Reg #2	17'15	DD(M	3614,89	9,87
Reg #3	19'27	DD(M	32606,67	88,99
Sum in ROI			36641,53	100,00
Area (total)			37239,62	
BKG1			9,177	
Remainder			598,09	1,61

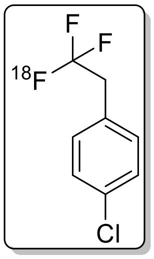

Figure S102: Analytical HPLC chromatogram from screening, top channel = UV, bottom channel = activity. HPLC spectrum of 1,2-dichloro-4-(2,2-difluoro-2-(fluoro-^{18}F)ethyl)benzene **([^{18}F]6b)**. 100 µL organic phase in 1 mL MeCN: H_2O =50:50. **(**MeCN: H_2O =50:50. Flow rate = 1.5 mL/min. Injected volume = 10 uL).

Measurement 200521-P1 Uni Oslo Page 1/1
c:\GINA_NT\LUNAPFP Mudasir AcN_H2O 50_50\200521-P1

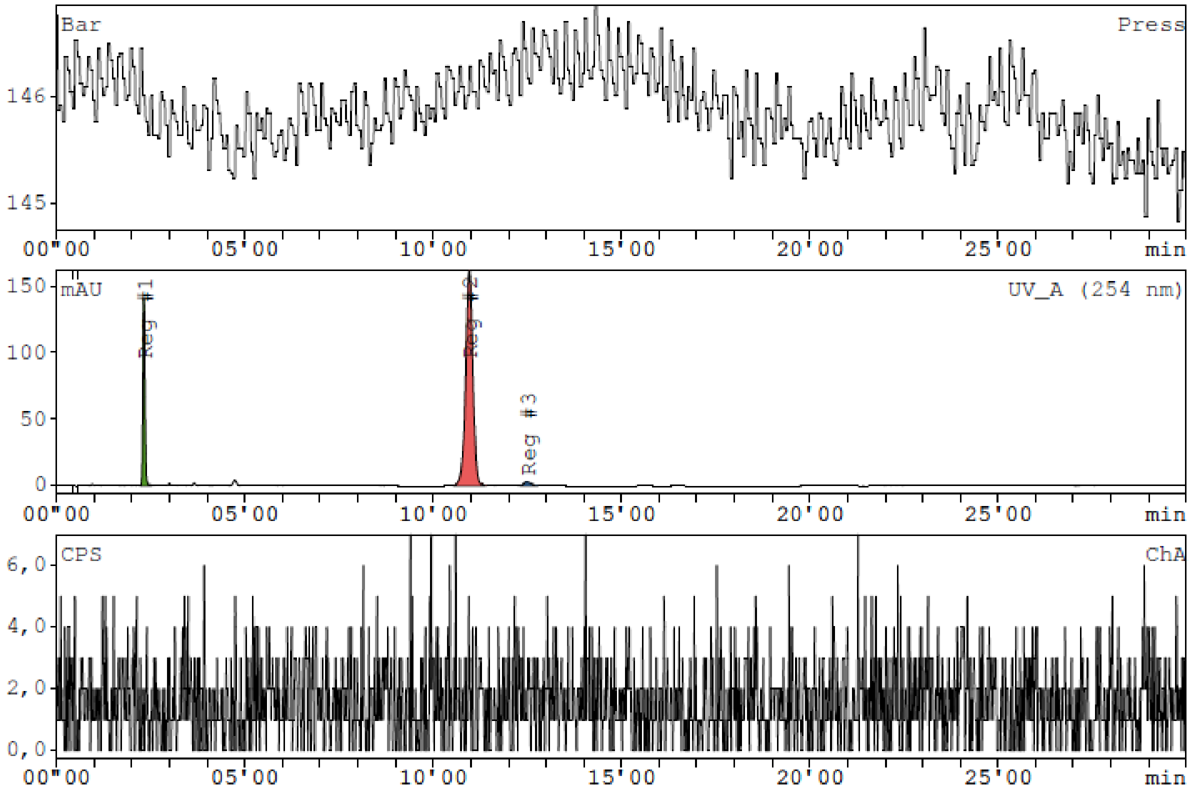

Sample description

Measurement:	200521-P1, injection :	20.05.2021 13:30
Method:	LUNAPFP Mudasir AcN_H2O 50_50 from:	17.12.2020 09:30
CH3:CN=50:50, Flow 1,5 mL/Min, injection vol. 10 microL		
Radio detector: raytest Gabi Star	Serial Nr.: #30745 raytest GINA star 20.04.09 Firmware V4.8	
Software Version: 5.9, Service Pack 8, Build 5076		

Integration UV_A (254 nm)

Substance	R/T s	Type	Area mAU*s	%Area %
Reg #1	02'20	DD(M	478,325	18,19
Reg #2	10'58	DD(M	2110,523	80,25
Reg #3	12'30	DD(M	41,017	1,56
Sum in ROI			2629,865	100,00
Area (total)			2840,243	
BKG1			-0,0247	

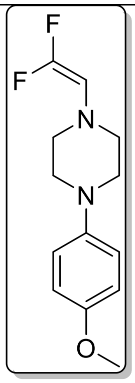

Figure S103: Analytical HPLC chromatogram from screening, top channel = UV, bottom channel = activity. HPLC spectrum of 1-(2,2-difluorovinyl)-4-(4-methoxyphenyl)piperazine **(7a)** (precursor). 1 mg of the sample dissolved in 1 mL MeCN: H_2O =50:50. **(**MeCN: H_2O =50:50. Flow rate = 1.5 mL/min. Injected volume = 10 uL).

Measurement 200521-R1 Uni Oslo Page 1/1

c:\GINA_NT\LUNAPFP Mudasir AcN_H2O 50_50\200521-R1

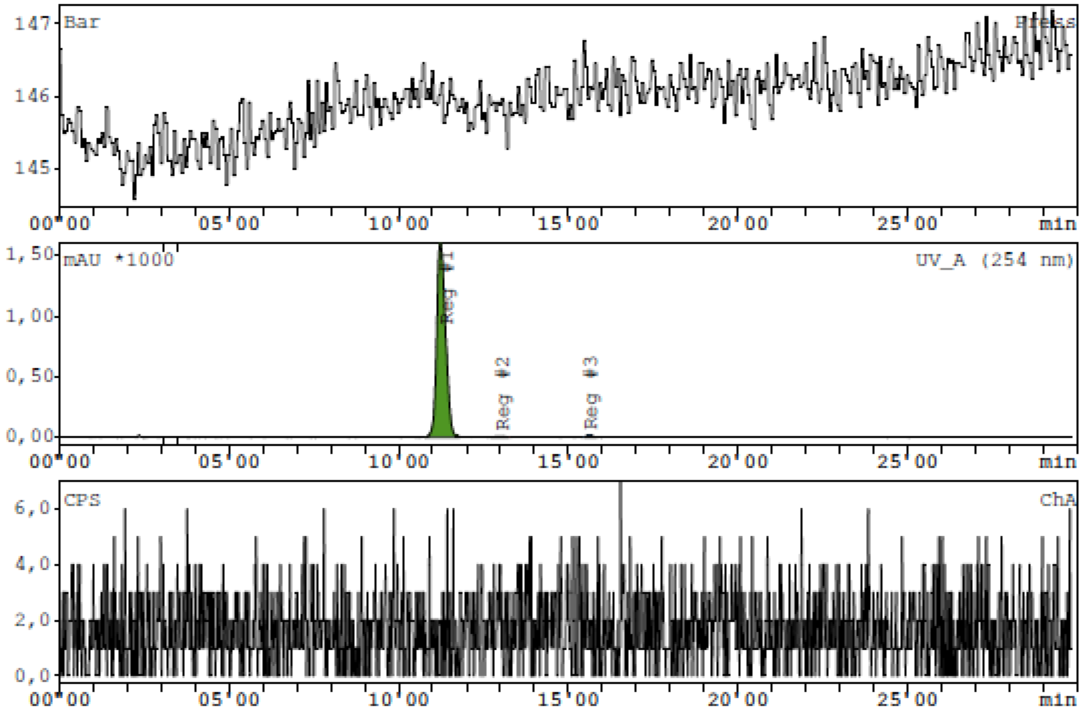

Sample description

Measurement:	200521-R1, injection :	20.05.2021 14:03	
Method:	LUNAPFP Mudasir AcN_H2O 50_50 from:		17.12.2020 09:30
CH3:CN=50:50, Flow 1,5 mL/Min, injection vol. 10 microL			
Radio detector: raytest Gabi Star	Serial Nr.: #30745 raytest GINA star 20.04.09 Firmware V4.8		
Software Version: 5.9, Service Pack 8, Build 5076			

Integration UV_A (254 nm)

Substance	R/T s	Type	Area mAU*s	%Area %
Reg #1	11'14	DD(M	28606,09	98,65
Reg #2	13'01	DD(M	198,34	0,68
Reg #3	15'38	DD(M	191,78	0,66
Sum in ROI			28996,20	100,00
Area (total)			28422,91	
BKG1			0,583	

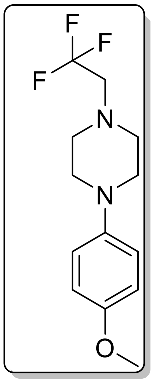

Figure S104: Analytical HPLC chromatogram from screening, top channel = UV, bottom channel = activity. HPLC spectrum of 1-(4-methoxyphenyl)-4-(2,2,2-trifluoroethyl)piperazine **(7b)** (reference). 1 mg of the sample dissolved in 1 mL MeCN: H_2O =50:50. **(**MeCN: H_2O =50:50. Flow rate = 1.5 mL/min. Injected volume = 10 uL).

Measurement 160721-B2 Uni Oslo Page 1/2
c:\GINA_NT\LUNAPFP Mudasir AcN_H2O 50_50\160721-B2

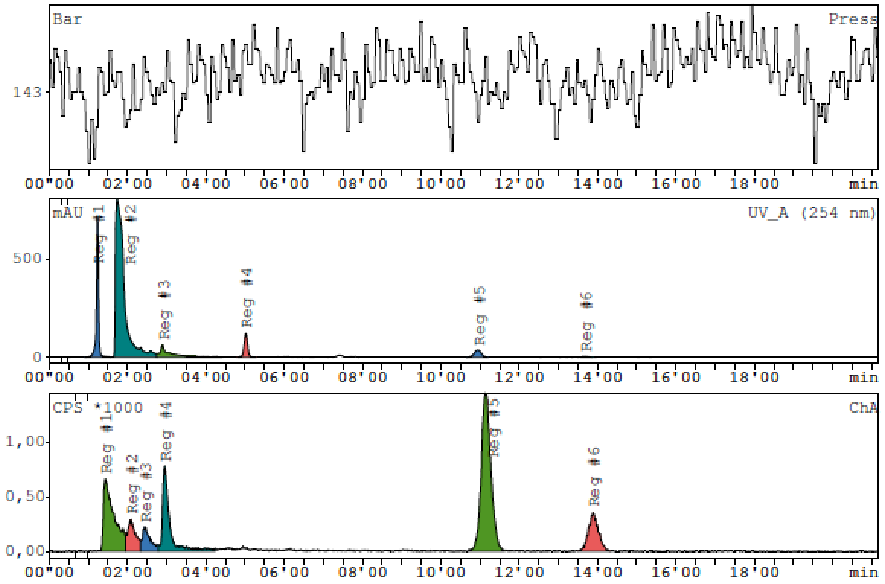

Measurement:	160721-B2, injection : 16.07.2021 13:28
Method:	LUNAPFP Mudasir AcN_H2O 50_50 from: 17.12.2020 09:30
CH3CN:H2O=50:50, Flow rate 1,5 mL/min, injection 10 micro L	
Radio detector: raytest Gabi Star	Serial Nr.: #30745 raytest GINA star 20.04.09 Firmware V4.8
Software Version: 5.9, Service Pack 8, Build 5076	

ChA

Substance	R/T s	Type	Area Counts	%Area %
Reg #1	01'26	DD(M	12030,65	20,43
Reg #2	02'06	DD(M	4151,59	7,05
Reg #3	02'26	DD(M	3112,28	5,29
Reg #4	02'57	DD(M	9275,07	15,75
Reg #5	11'09	DD(M	24226,54	41,15
Reg #6	13'53	DD(M	6084,22	10,33
Sum in ROI			58880,34	100,00
Area (total)			61586,30	
BKG1			5,167	
Remainder			2705,95	4,39

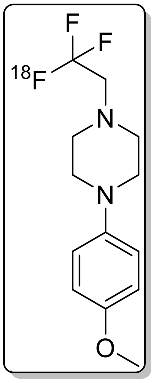

UV_A (254 nm)

Substance	R/T s	Type	Area mAU*s	%Area %
Reg #1	01'14	DD(M	2525,71	14,37
Reg #2	01'45	DD(M	12512,96	71,20
Reg #3	02'54	DD(M	1223,01	6,96
Reg #4	05'01	DD(M	765,82	4,36
Reg #5	10'57	DD(M	485,93	2,76
Reg #6	13'41	DD(M	61,41	0,35

Figure S105: Analytical HPLC chromatogram from screening, top channel = UV, bottom channel = activity. HPLC spectrum of 1-(2,2-difluoro-2-(fluoro-^{18}F)ethyl)-4-(4-methoxyphenyl)piperazine **([^{18}F]7b)** before purification (Direct labeling method). 100 µL organic phase in 1 mL MeCN: H_2O =50:50. **(**MeCN: H_2O =50:50. Flow rate = 1.5 mL/min. Injected volume = 10 uL).

Measurement 160721-B3 Uni Oslo Page 1/2

c:\GINA_NT\LUNAPFP Mudasir AcN_H2O 50_50\160721-B3

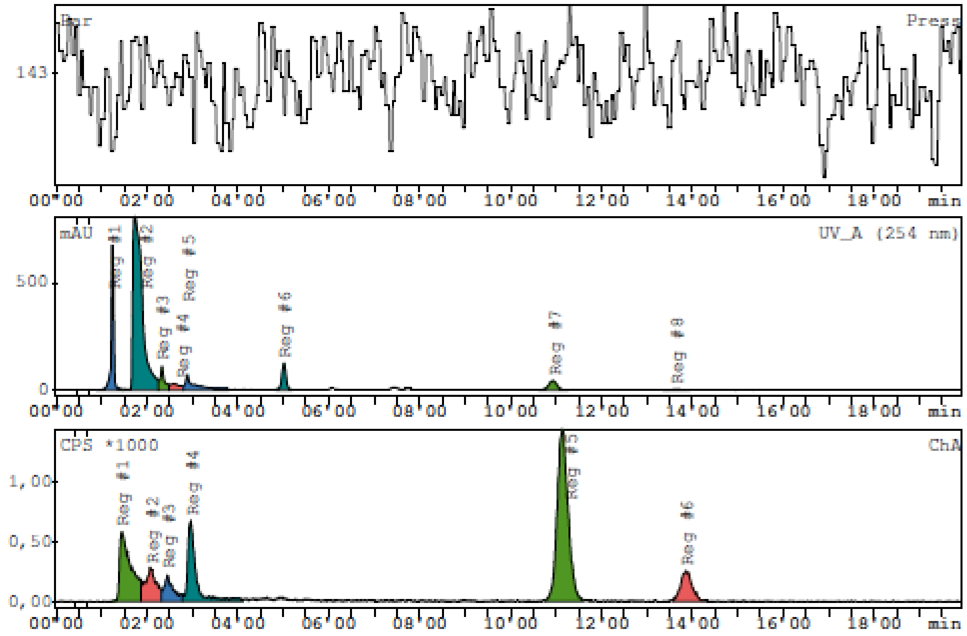

Measurement:	160721-B3, injection :	16.07.2021 13:51
Method:	LUNAPFP Mudasir AcN_H2O 50_50 from:	17.12.2020 09:30
CH3CN:H2O=50:50, Flow rate 1,5 mL/min, injection 10 micro L		
Radio detector: raytest Gabi Star	Serial Nr.: #30745 raytest GINA star 20.04.09 Firmware V4.8	
Software Version: 5.9, Service Pack 8, Build 5076		

ChA

Substance	R/T s	Type	Area Counts	%Area %
Reg #1	01'27	DD(M	9849,67	18,21
Reg #2	02'05	DD(M	4808,13	8,89
Reg #3	02'27	DD(M	3034,68	5,61
Reg #4	02'58	DD(M	8340,34	15,42
Reg #5	11'08	DD(M	23622,13	43,67
Reg #6	13'51	DD(M	4437,86	8,20
Sum in ROI			54092,81	100,00
Area (total)			58745,79	
BKG1			2,571	
Remainder			4652,99	7,92

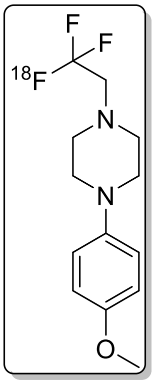

Figure S106: Analytical HPLC chromatogram from screening, top channel = UV, bottom channel = activity. HPLC spectrum of 1-(2,2-difluoro-2-(fluoro-^{18}F)ethyl)-4-(4-methoxyphenyl)piperazine **([^{18}F]7b)** before purification (Indirect labeling method). 100 µL organic phase in 1 mL MeCN: H_2O =50:50. **(**MeCN: H_2O =50:50. Flow rate = 1.5 mL/min. Injected volume = 10 uL).

Measurement 150721-I-pure-ref-co Uni Oslo Page 1/1
c:\GINA_NT\LUNAPFP Mudasir AcN_H2O 50_50\150721-I-pure-ref-co

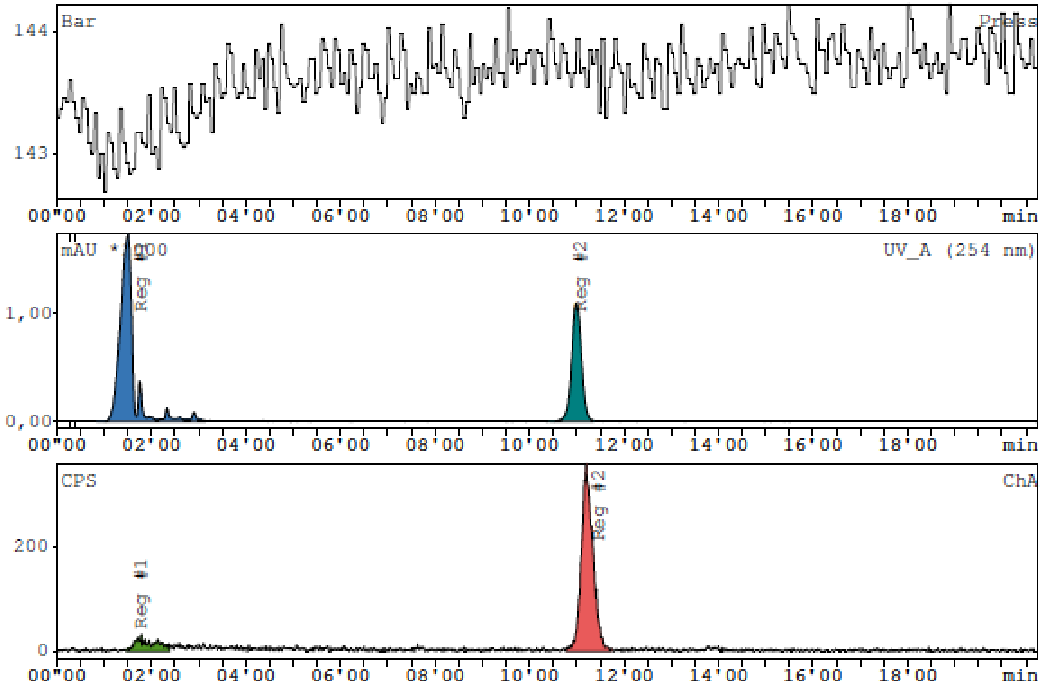

Measurement:	150721-I-pure-ref-co, injection :	15.07.2021 16:32
Method:	LUNAPFP Mudasir AcN_H2O 50_50 from:	17.12.2020 09:30
CH3CN:H2O=50:50, Flow rate 1,5 mL/min, injection 10 micro L		
Radio detector: raytest Gabi Star	Serial Nr.: #30745 raytest GINA star 20.04.09 Firmware V4.8	
Software Version: 5.9, Service Pack 8, Build 5076		

ChA

Substance	R/T s	Type	Area Counts	%Area %
Reg #1	01'45	DD(M	762,020	11,30
Reg #2	11'12	DD(M	5979,233	88,70
Sum in ROI			6741,253	100,00
Area (total)			10683,288	
Ext. BKG			0,00 CPS	

UV_A (254 nm)

Substance	R/T s	Type	Area mAU*s	%Area %
Reg #1	01'30	DD(M	30703,88	66,33
Reg #2	11'00	DD(M	15585,84	33,67
Sum in ROI			46289,72	100,00
Area (total)			46908,72	
BKG1			-0,026	

Figure S107: Analytical HPLC chromatogram from screening, top channel = UV, bottom channel = activity. HPLC chromatogram of 1-(2,2-difluoro-2-(fluoro-^{18}F)ethyl)-4-(4-methoxyphenyl)piperazine **([^{18}F]7b),** purified sample and 1-(4-methoxyphenyl)-4-(2,2,2-trifluoroethyl)piperazine (reference) together as a co-injection (5 uL + 5 uL). Flow rate = 1.5 mL/min. Injected volume = 10 uL).

Measurement 160721-A1-pure Uni Oslo Page 1/1

c:\GINA_NT\LUNAPFP Mudasir AcN_H2O 50_50\160721-A1-pure

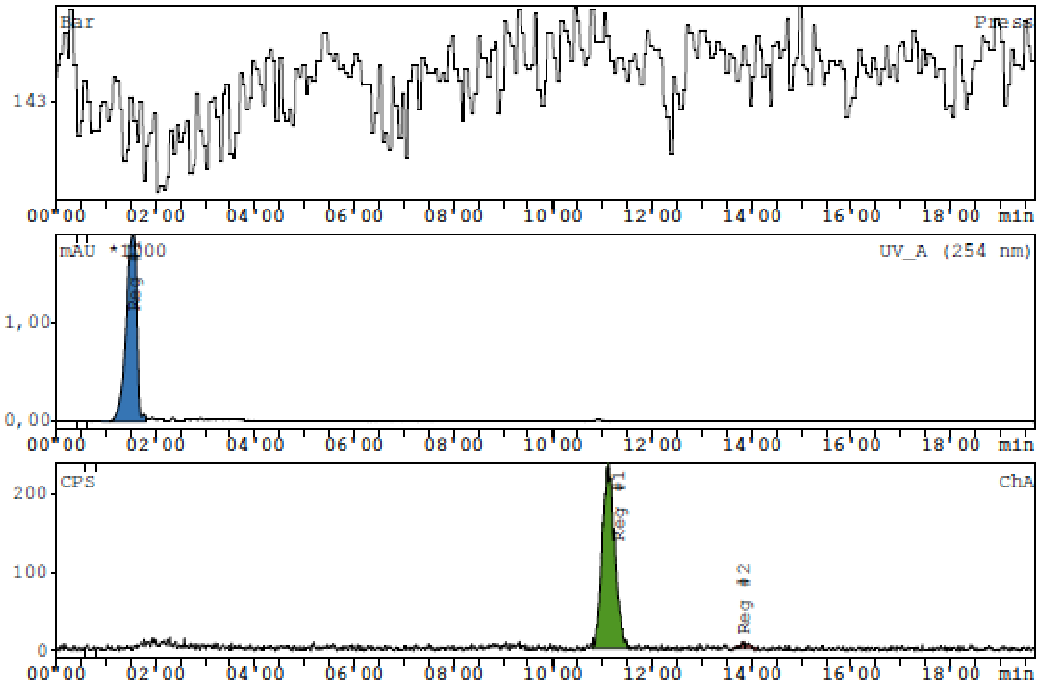

Measurement:	160721-A1-pure, injection :	16.07.2021 14:16	
Method:	LUNAPFP Mudasir AcN_H2O 50_50 from:		17.12.2020 09:30
CH3CN:H2O=50:50, Flow rate 1,5 mL/min, injection 10 micro L			
Radio detector: raytest Gabi Star	Serial Nr.: #30745 raytest GINA star 20.04.09 Firmware V4.8		
Software Version: 5.9, Service Pack 8, Build 5076			

ChA

Substance	R/T s	Type	Area Counts	%Area %
Reg #1	11'07	DD(M	3996,931	98,23
Reg #2	13'50	DD(M	72,001	1,77
Sum in ROI			4068,932	100,00
Area (total)			4565,796	
BKG1			2,8572	
Remainder			496,86	10,88

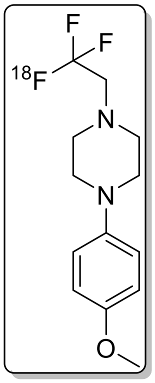

UV_A (254 nm)

Substance	R/T s	Type	Area mAU*s	%Area %
Reg #1	01'33	DD(M	27007,26	100,00
Sum in ROI			27007,26	100,00
Area (total)			31923,21	
BKG1			-0,041	

Figure S108: Analytical HPLC chromatogram from screening, top channel = UV, bottom channel = activity. HPLC chromatogram of pure 1-(2,2-difluoro-2-(fluoro-^{18}F)ethyl)-4-(4-methoxyphenyl)piperazine **([^{18}F]7b)**. 100 µL organic phase in 1 mL MeCN: H_2O =50:50. **(**MeCN: H_2O =50:50. Flow rate = 1.5 mL/min. Injected volume = 10 uL).

Measurement 240622-Sel-precursor Uni Oslo Page 1/1
c:\GINA_NT\LUNAPFP MUDASIR 70_30-H2O_CH3CN\240622-Sel-precursor

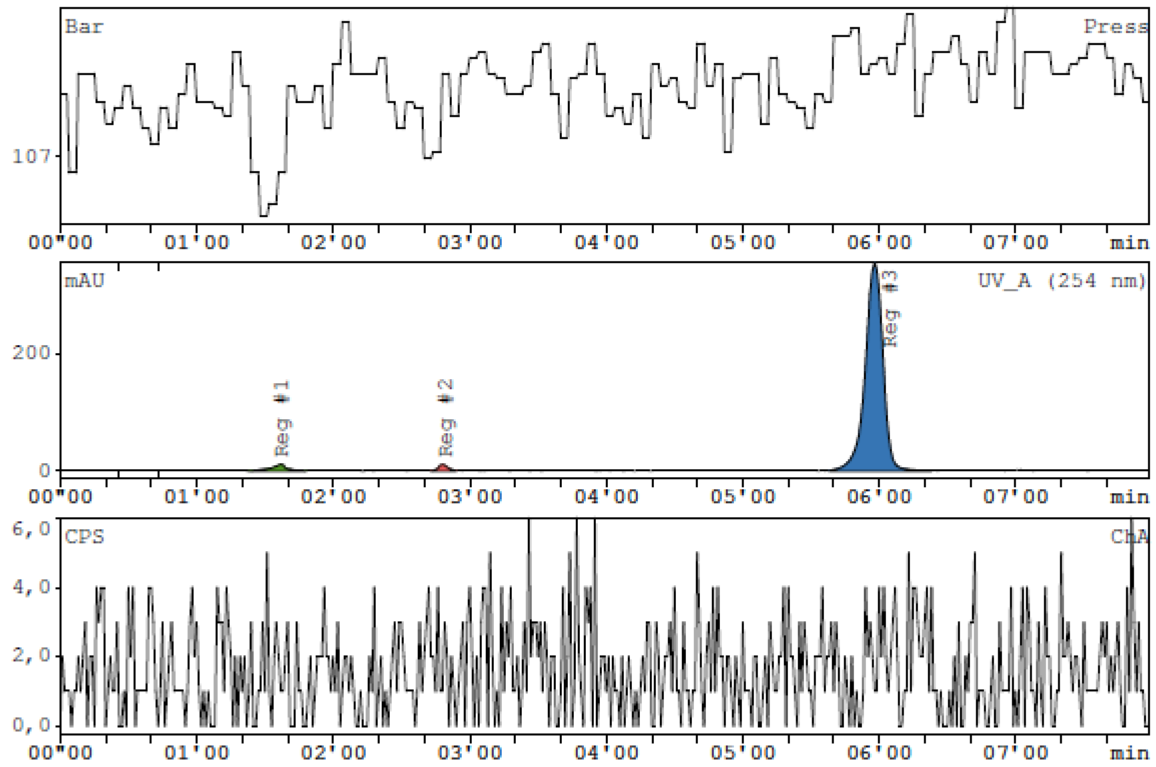

Measurement:	240622-Sel-precursor, injection : 24.06.2022 16:26
Method:	LUNAPFP MUDASIR 70_30-H2O_CH3CN from: 11.04.2022 11:26
MeCN:H2O =70:30. Flow rate = 1,0 mL/min. Injected volume = 10 uL.	
Radio detector: raytest Gabi Star	Serial Nr.: #30745 raytest GINA star 20.04.09 Firmware V4.8
Software Version: 5.9, Service Pack 8, Build 5076	

UV_A (254 nm)

Substance	R/T s	Type	Area mAU*s	%Area %
Reg #1	01'36	DD(M	86,262	2,58
Reg #2	02'48	DD(M	51,552	1,54
Reg #3	05'58	DD(M	3200,904	95,87
Sum in ROI			3338,718	100,00
Area (total)			3402,895	
BKG1			-0,0624	

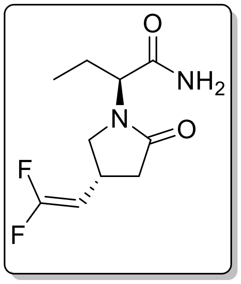

Figure S109: Analytical HPLC chromatogram from screening, top channel = UV, bottom channel = activity. HPLC spectrum of (S)-2-((R)-4-(2,2-difluorovinyl)-2-oxopyrrolidin-1-yl)butanamide **(8a)**. 1 mg precursor dissolved in 1 mL MeCN: H_2O =50:50. **(**MeCN: H_2O =30:70. Flow rate = 1.0 mL/min. Injected volume = 10 uL).

Measurement 240622-Sel-reference Uni Oslo Page 1/1
c:\GINA_NT\LUNAPFP MUDASIR 70_30-H2O_CH3CN\240622-Sel-reference

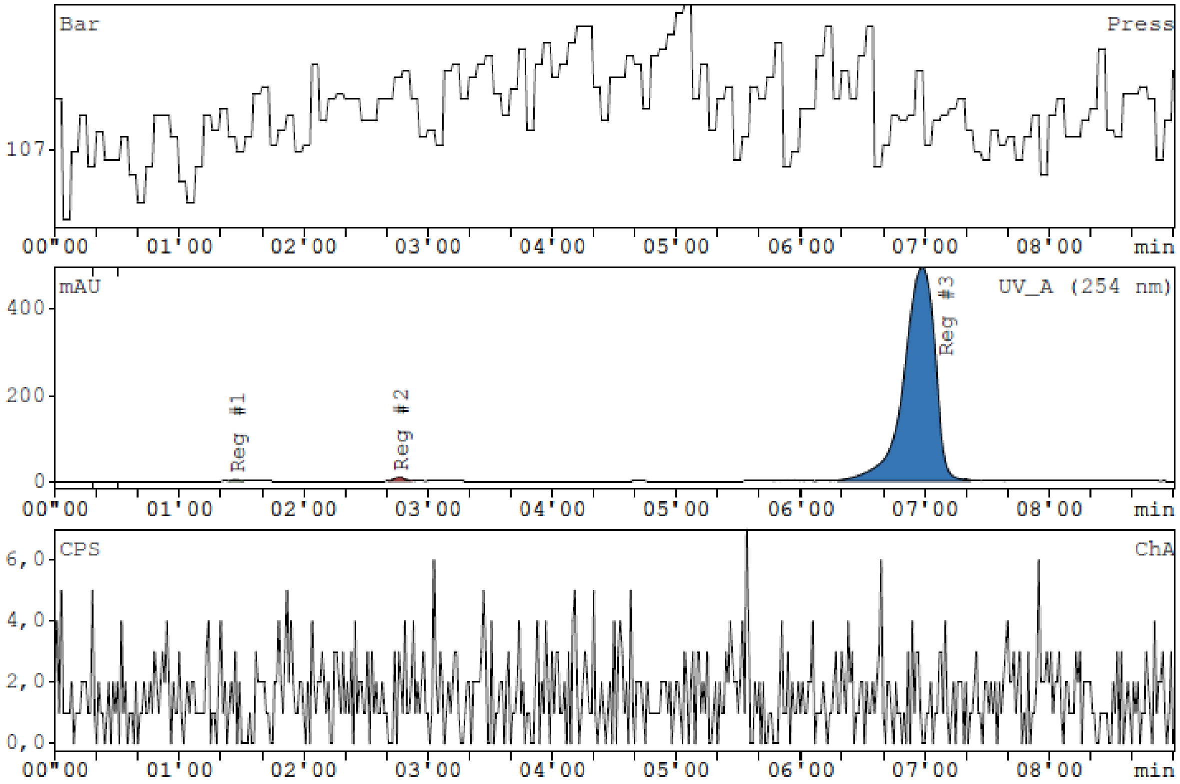

Measurement:	240622-Sel-reference, injection : 24.06.2022 16:14
Method:	LUNAPFP MUDASIR 70_30-H2O_CH3CN from: 11.04.2022 11:26
MeCN:H2O =70:30. Flow rate = 1,0 mL/min. Injected volume = 10 uL.	
Radio detector: raytest Gabi Star	Serial Nr.: #30745 raytest GINA star 20.04.09 Firmware V4.8
Software Version: 5.9, Service Pack 8, Build 5076	

UV_A (254 nm)

Substance	R/T s	Type	Area mAU*s	%Area %
Reg #1	01'27	DD(M	14,930	0,17
Reg #2	02'46	DD(M	52,109	0,59
Reg #3	06'59	DD(M	8711,108	99,24
Sum in ROI			8778,147	100,00
Area (total)			8875,366	
BKG1			-0,0039	

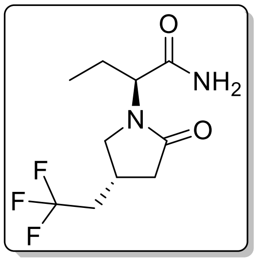

Figure S110: Analytical HPLC chromatogram from screening, top channel = UV, bottom channel = activity. HPLC spectrum of (S)-2-((R)-2-oxo-4-(2,2,2-trifluoroethyl)pyrrolidin-1-yl)butanamide **(8b)**. 1 mg precursor dissolved in 1 mL MeCN: H_2O =50:50. **(**MeCN: H_2O =30:70. Flow rate = 1.0 mL/min. Injected volume = 10 uL).

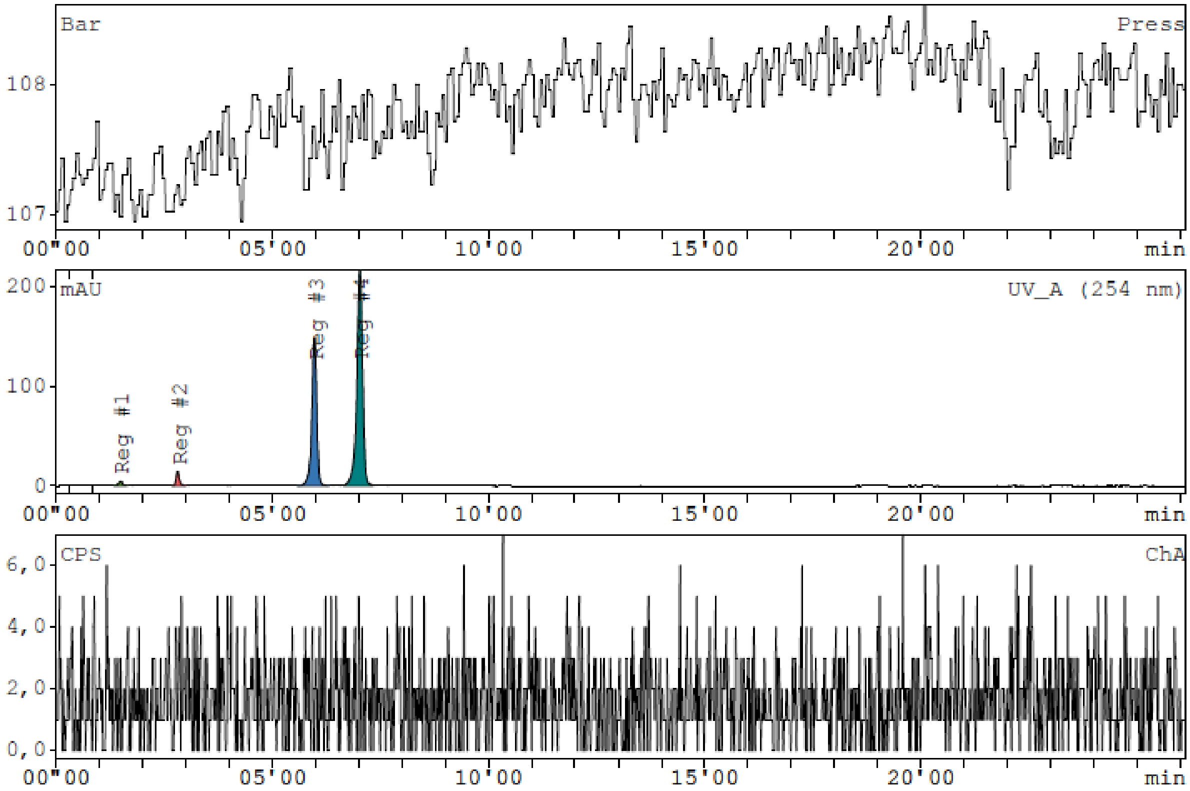

Measurement: 240622-Sel-pre-ref-coinjecti, injection : 24.06.2022 16:36
Method: LUNAPFP MUDASIR 70_30-H2O_CH3CN from: 11.04.2022 11:26
MeCN:H2O =70:30. Flow rate = 1,0 mL/min. Injected volume = 10 uL.
Radio detector: raytest Gabi Star Serial Nr.: #30745 raytest GINA star 20.04.09 Firmware V4.8
Software Version: 5.9, Service Pack 8, Build 5076

UV_A (254 nm)

Substance	R/T s	Type	Area mAU*s	%Area %
Reg #1	01'29	DD(M	31,913	0,99
Reg #2	02'49	DD(M	73,097	2,27
Reg #3	05'59	DD(M	1148,805	35,60
Reg #4	07'02	DD(M	1973,098	61,15
Sum in ROI			3226,912	100,00
Area (total)			3238,066	
BKG1			0,0689	

Figure S111: Analytical HPLC chromatogram from screening, top channel = UV, bottom channel = activity. HPLC spectrum of (S)-2-((R)-4-(2,2-difluorovinyl)-2-oxopyrrolidin-1-yl)butanamide **(8a)** and (S)-2-((R)-2-oxo-4-(2,2,2-trifluoroethyl)pyrrolidin-1-yl)butanamide **(8b)** together as a co-injection (5 uL + 5 uL). (MeCN: H_2O =30:70. Flow rate = 1.0 mL/min. Injected volume = 10 uL).

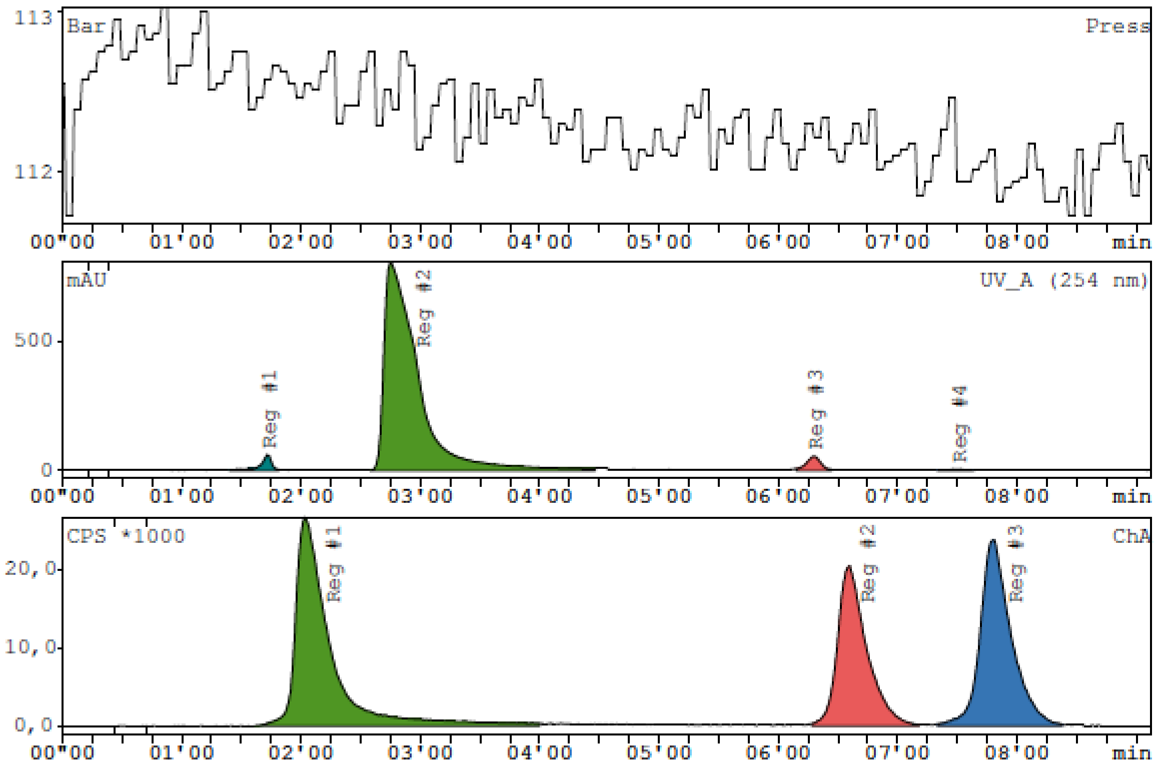

Measurement:	030123-A1, injection : 03.01.2023 12:01
Method:	LUNAPFP MUDASIR 70_30-H2O_CH3CN from: 28.09.2022 11:01
H2O:CH3CN =70:30. Flow rate = 1,0 mL/min. Injected volume = 10 uL.	
Radio detector: raytest Gabi Star	Serial Nr.: #30745 raytest GINA star 20.04.09 Firmware V4.8
Software Version: 5.9, Service Pack 8, Build 5076	

ChA

Substance	R/T s	Type	Area Counts	%Area %
Reg #1	02'02	DD(M	526583,6	40,53
Reg #2	06'36	DD(M	345120,0	26,56
Reg #3	07'48	DD(M	427571,8	32,91
Sum in ROI			1299275,3	100,00
Area (total)			1330780,9	
BKG1			2,50 CPS	
Remainder			31505,53	2,37

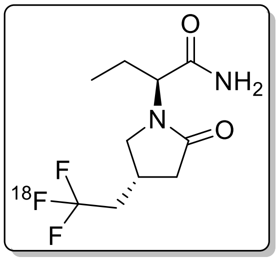

Figure S112: Analytical HPLC chromatogram from screening, top channel = UV, bottom channel = activity. HPLC spectrum of (S)-2-((R)-4-(2,2-difluoro-2-(fluoro-^{18}F)ethyl)-2-oxopyrrolidin-1-yl)butanamide (**[^{18}F]8b).** 100 µL organic phase in 1 mL MeCN: H_2O =50:50. (MeCN: H_2O =30:70. Flow rate = 1.0 mL/min. Injected volume = 10 uL).

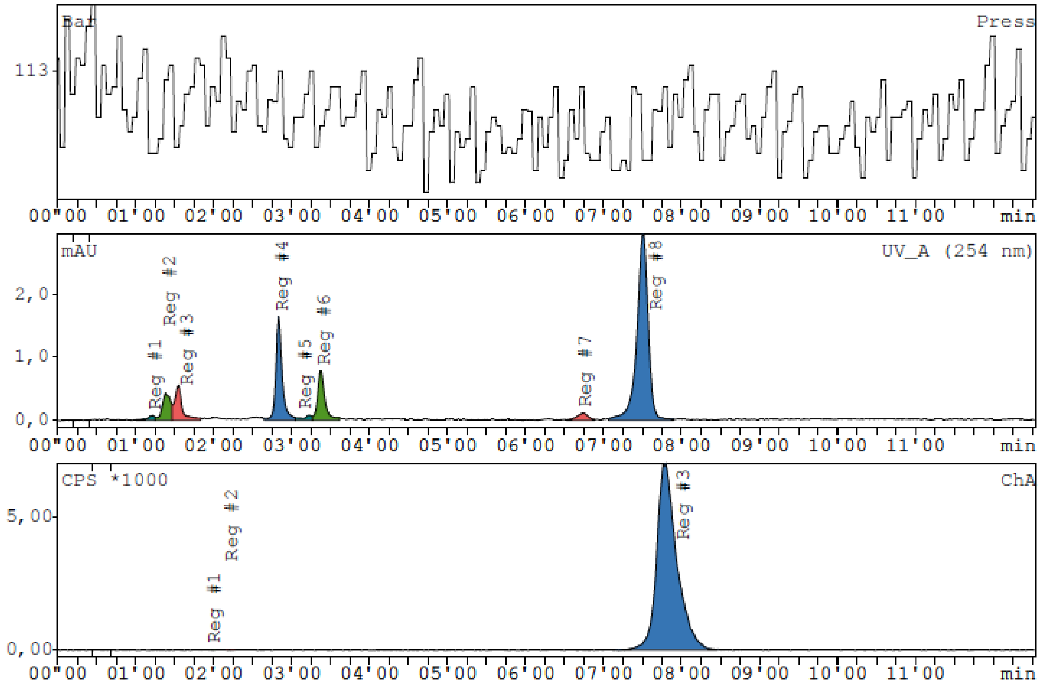

Measurement:	030123-A1-15-2, injection : 03.01.2023 14:52
Method:	LUNAPFP MUDASIR 70_30-H2O_CH3CN from: 28.09.2022 11:01
H2O:CH3CN =70:30. Flow rate = 1,0 mL/min. Injected volume = 10 uL.	
Radio detector: raytest Gabi Star	Serial Nr.: #30745 raytest GINA star 20.04.09 Firmware V4.8
Software Version: 5.9, Service Pack 8, Build 5076	

ChA

Substance	R/T s	Type	Area Counts	%Area %
Reg #1	01'59	DD(M	26,8	0,02
Reg #2	02'12	DD(M	30,6	0,02
Reg #3	07'47	DD(M	124129,9	99,95
Sum in ROI			124187,3	100,00
Area (total)			125047,7	
BKG1			2,07 CPS	
Remainder			860,44	0,69

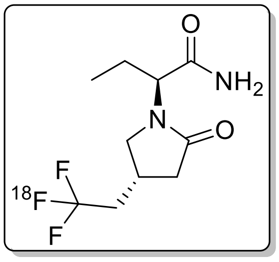

Figure S113: Analytical HPLC chromatogram from screening, top channel = UV, bottom channel = activity. HPLC spectrum of pure (S)-2-((R)-4-(2,2-difluoro-2-(fluoro-^{18}F)ethyl)-2-oxopyrrolidin-1-yl)butanamide **([^{18}F]8b)** purified using semipreperative HPLC**.** 100 µL organic phase in 1 mL MeCN: H_2O =50:50. (MeCN: H_2O =30:70. Flow rate = 1.0 mL/min. Injected volume = 10 uL).

Measurement 280722-A4-ref-co-injection Uni Oslo Page 1/1
c:\GINA_NT\LUNAPFP MUDASIR 70_30-H2O_CH3CN\280722-A4-ref-co-injection

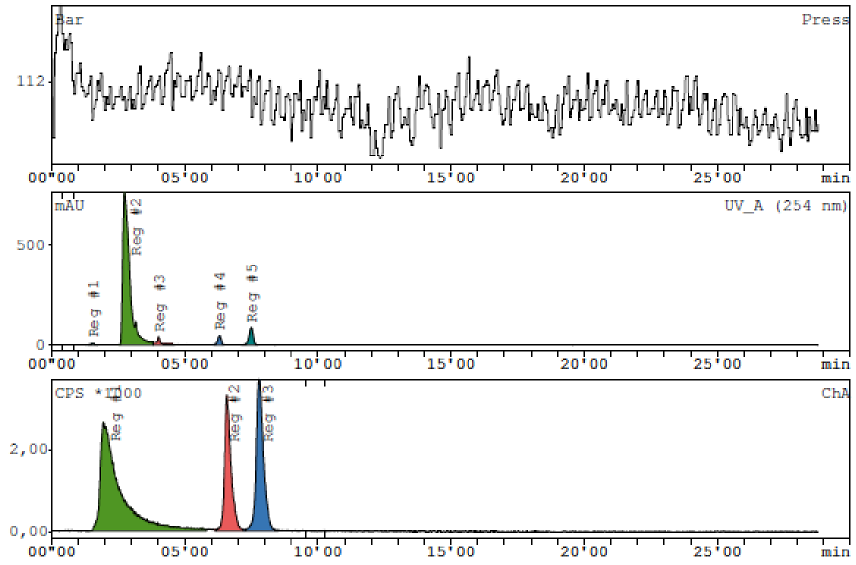

Measurement:	280722-A4-ref-co-injection, injection :	28.07.2022 12:23
Method:	LUNAPFP MUDASIR 70_30-H2O_CH3CN from:	11.04.2022 11:26
MeCN:H2O =70:30. Flow rate = 1,0 mL/min. Injected volume = 10 uL.		
Radio detector: raytest Gabi Star	Serial Nr.: #30745 raytest GINA star 20.04.09 Firmware V4.8	
Software Version: 5.9, Service Pack 8, Build 5076		

ChA

Substance	R/T s	Type	Area Counts	%Area %
Reg #1	01'58	DD(M	134345,9	52,20
Reg #2	06'35	DD(M	55582,8	21,60
Reg #3	07'48	DD(M	67442,4	26,20
Sum in ROI			257371,1	100,00
Area (total)			244428,8	
BKG1			22,58	
Remainder			-12942,27	-5,29

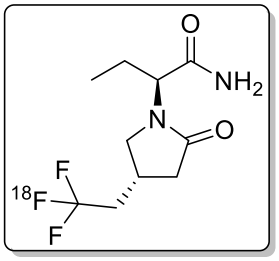

UV_A (254 nm)

Substance	R/T s	Type	Area mAU*s	%Area %
Reg #1	01'33	DD(M	95,86	0,62
Reg #2	02'45	DD(M	13514,93	87,70
Reg #3	04'01	DD(M	524,82	3,41
Reg #4	06'18	DD(M	387,28	2,51
Reg #5	07'30	DD(M	887,33	5,76
Sum in ROI			15410,21	100,00
Area (total)			14875,52	
BKG1			-0,147	

Figure S114: Analytical HPLC chromatogram from screening, top channel = UV, bottom channel = activity. HPLC chromatogram of (S)-2-((R)-4-(2,2-difluoro-2-(fluoro-^{18}F)ethyl)-2-oxopyrrolidin-1-yl)butanamide **([^{18}F]8b)** and (S)-2-((R)-2-oxo-4-(2,2,2-trifluoroethyl)pyrrolidin-1-yl)butanamide (reference) together as a co-injection (5 uL + 5 uL). (MeCN: H_2O =30:70. Flow rate = 1.0 mL/min. Injected volume = 10 uL).

Measurement 250822-B22 Uni Oslo Page 1/2
c:\GINA_NT\LUNAPFP MUDASIR 70_30-H2O_CH3CN\250822-B22

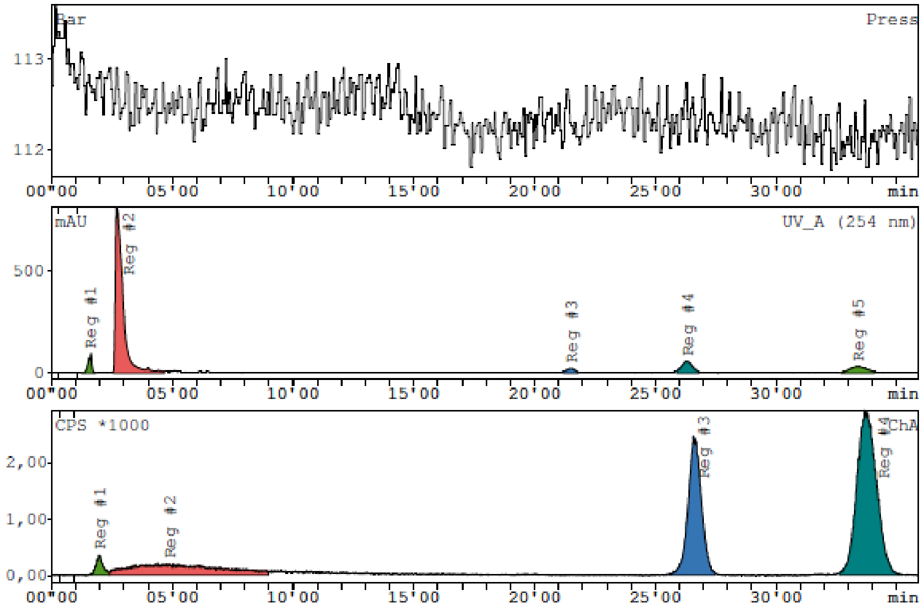

Measurement: 250822-B22, injection : 25.08.2022 14:20
Method: LUNAPFP MUDASIR 70_30-H2O_CH3CN from: 19.08.2022 13:52
MeCN:H2O =70:30. Flow rate = 1,0 mL/min. Injected volume = 10 uL.
Radio detector: raytest Gabi Star Serial Nr.: #30745 raytest GINA star 20.04.09 Firmware V4.8
Software Version: 5.9, Service Pack 8, Build 5076

ChA

Substance	R/T s	Type	Area Counts	%Area %
Reg #1	01'59	DD(M	6948,8	2,22
Reg #2	04'53	DD(M	50424,9	16,10
Reg #3	26'37	DD(M	91923,4	29,35
Reg #4	33'42	DD(M	163927,0	52,34
Sum in ROI			313224,1	100,00
Area (total)			333753,8	
BKG1			15,95	
Remainder			20529,64	6,15

UV_A (254 nm)

Substance	R/T s	Type	Area mAU*s	%Area %
Reg #1	01'36	DD(M	894,87	4,09
Reg #2	02'43	DD(M	17120,96	78,21
Reg #3	21'31	DD(M	537,59	2,46
Reg #4	26'19	DD(M	1782,21	8,14
Reg #5	33'23	DD(M	1555,11	7,10
Sum in ROI			21890,75	100,00
Area (total)			23895,64	
BKG1			-0,060	

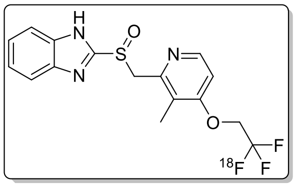

*Figure S115: Analytical HPLC chromatogram from screening, top channel = UV, bottom channel = activity. HPLC spectrum of [^{18}F]Lansoprazole **([^{18}F]9b).** 100 µL organic phase in 1 mL MeCN: H_2O =50:50. (MeCN: H_2O =30:70. Flow rate = 1.0 mL/min. Injected volume = 10 uL).*

Measurement 250822-B22-coinje-repeat Uni Oslo Page 1/2
c:\GINA_NT\LUNAPFP MUDASIR 70_30-H2O_CH3CN\250822-B22-coinje-repeat

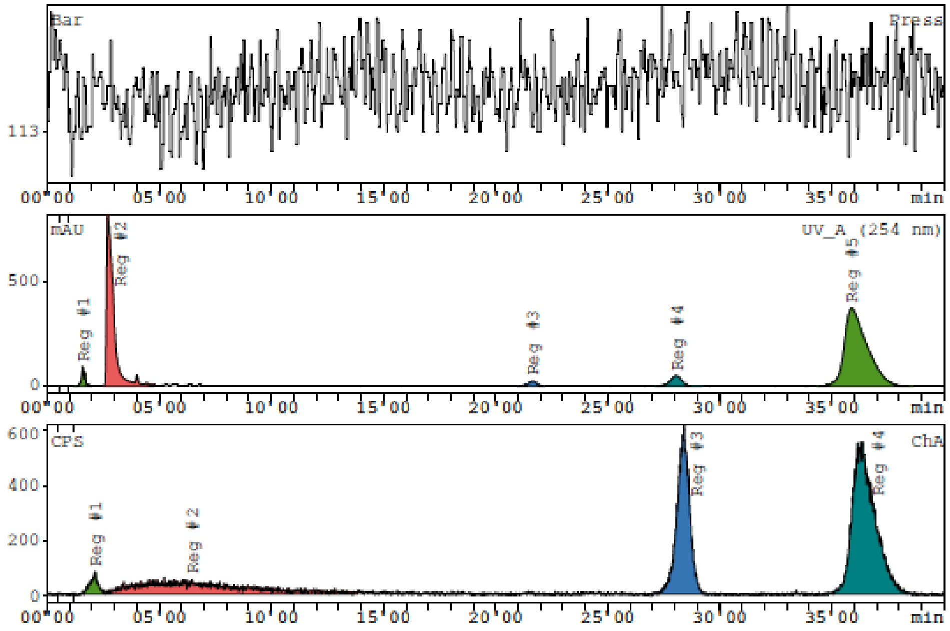

Measurement: 250822-B22-coinje-repeat, injection :	25.08.2022 17:47
Method: LUNAPFP MUDASIR 70_30-H2O_CH3CN from:	19.08.2022 13:52
MeCN:H2O =70:30. Flow rate = 1,0 mL/min. Injected volume = 10 uL.	
Radio detector: raytest Gabi Star Serial Nr.: #30745 raytest GINA star 20.04.09 Firmware V4.8	
Software Version: 5.9, Service Pack 8, Build 5076	

ChA

Substance	R/T s	Type	Area Counts	%Area %
Reg #1	02'08	DD(M	1818,73	2,36
Reg #2	06'28	DD(M	14778,96	19,14
Reg #3	28'24	DD(M	22855,65	29,61
Reg #4	36'17	DD(M	37746,40	48,89
Sum in ROI			77199,73	100,00
Area (total)			78645,94	
BKG1			6,628	
Remainder			1446,21	1,84

UV_A (254 nm)

Substance	R/T s	Type	Area mAU*s	%Area %
Reg #1	01'35	DD(M	909,73	1,96
Reg #2	02'44	DD(M	17173,31	36,97
Reg #3	21'40	DD(M	667,10	1,44
Reg #4	28'04	DD(M	1746,54	3,76
Reg #5	35'54	DD(M	25961,39	55,88
Sum in ROI			46458,08	100,00
Area (total)			48148,36	
BKG1			0,019	

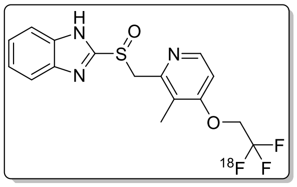

Figure S116: Analytical HPLC chromatogram from screening, top channel = UV, bottom channel = activity. HPLC chromatogram of [^{18}F]Lansoprazole **([^{18}F]9b)** and Lansoprazole (reference) together as a co-injection (5 uL + 5 uL). (MeCN: H_2O =30:70. Flow rate = 1.0 mL/min. Injected volume = 10 uL).

Measurement 170222-A Uni Oslo Page 1/2
c:\GINA_NT\LUNAPFP Mudasir AcN_H2O 50_50\170222-A

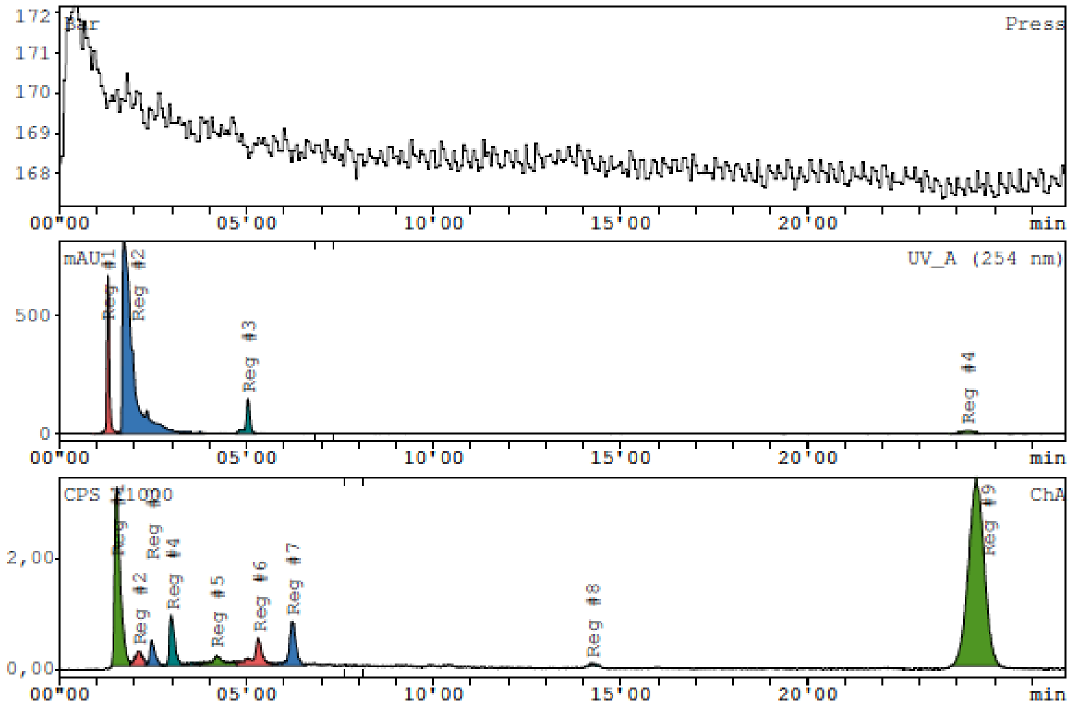

Measurement:	170222-A, injection : 17.02.2022 12:10
Method:	LUNAPFP Mudasir AcN_H2O 50_50 from: 17.12.2020 09:30

MeCN:H2O =50:50. Flow rate = 1,5 mL/min. Injected volume = 10 uL.
Radio detector: raytest Gabi Star Serial Nr.: #30745 raytest GINA star 20.04.09 Firmware V4.8
Software Version: 5.9, Service Pack 8, Build 5076

ChA

Substance	R/T s	Type	Area Counts	%Area %
Reg #1	01'32	DD(M	33059,33	19,54
Reg #2	02'07	DD(M	3595,96	2,13
Reg #3	02'28	DD(M	4269,54	2,52
Reg #4	03'00	DD(M	9437,39	5,58
Reg #5	04'13	DD(M	4271,14	2,52
Reg #6	05'19	DD(M	7914,47	4,68
Reg #7	06'14	DD(M	9738,18	5,76
Reg #8	14'14	DD(M	185,12	0,11
Reg #9	24'30	DD(M	96702,99	57,16
Sum in ROI			169174,11	100,00
Area (total)			118095,25	
BKG1			73,768	
Remainder			-51078,87	-43,25

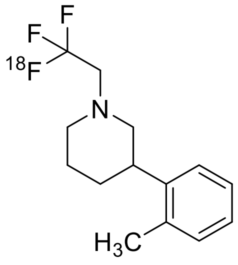

Figure S117: Analytical HPLC chromatogram from screening, top channel = UV, bottom channel = activity. HPLC chromatogram of 1-(2,2-difluoro-2-(fluoro-^{18}F)ethyl)-3-(o-tolyl)piperidine (**[^{18}F]10)**. 100 µL organic phase in 1 mL MeCN: H_2O =50:50. **(**MeCN: H_2O =50:50. Flow rate = 1.5 mL/min. Injected volume = 10 uL).

Measurement 170222-B Uni Oslo Page 1/2
c:\GINA_NT\LUNAPFP Mudasir AcN_H2O 50_50\170222-B

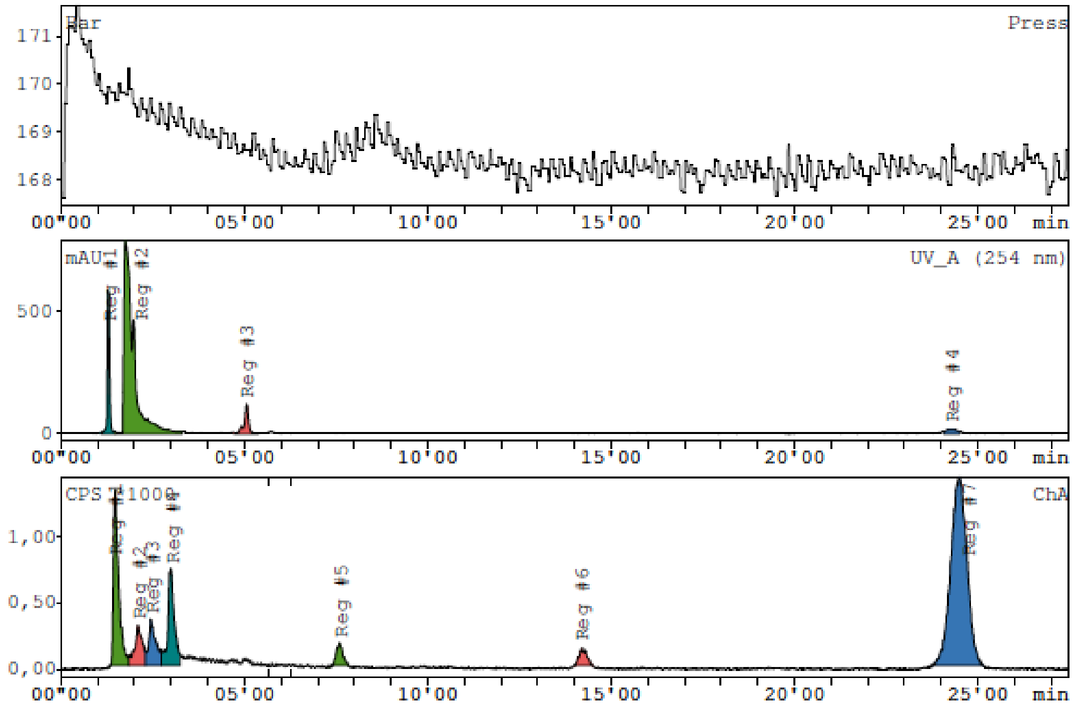

Measurement:	170222-B, injection : 17.02.2022 13:06
Method:	LUNAPFP Mudasir AcN_H2O 50_50 from: 17.12.2020 09:30
MeCN:H2O =50:50. Flow rate = 1,5 mL/min. Injected volume = 10 uL.	
Radio detector: raytest Gabi Star	Serial Nr.: #30745 raytest GINA star 20.04.09 Firmware V4.8
Software Version: 5.9, Service Pack 8, Build 5076	

ChA

Substance	R/T s	Type	Area Counts	%Area %
Reg #1	01'29	DD(M	12497,00	16,36
Reg #2	02'07	DD(M	4113,44	5,39
Reg #3	02'28	DD(M	4581,81	6,00
Reg #4	03'00	DD(M	8665,15	11,35
Reg #5	07'36	DD(M	1812,52	2,37
Reg #6	14'12	DD(M	1729,20	2,26
Reg #7	24'31	DD(M	42972,81	56,27
Sum in ROI			76371,93	100,00
Area (total)			53003,34	
BKG1			32,974	
Remainder			-23368,60	-44,09

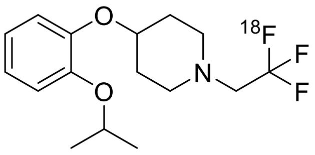

Figure S118: Analytical HPLC chromatogram from screening, top channel = UV, bottom channel = activity. HPLC chromatogram of 1-(2,2-difluoro-2-(fluoro-^{18}F)ethyl)-4-(2-isopropoxyphenoxy)piperidine (*[^{18}F]***11**). 100 µL organic phase in 1 mL MeCN: H_2O =50:50. (MeCN: H_2O =50:50. Flow rate = 1.5 mL/min. Injected volume = 10 uL).

Measurement 170222-B-Co injection Uni Oslo Page 1/2
c:\GINA_NT\LUNAPFP Mudasir AcN_H2O 50_50\170222-B-Co injection

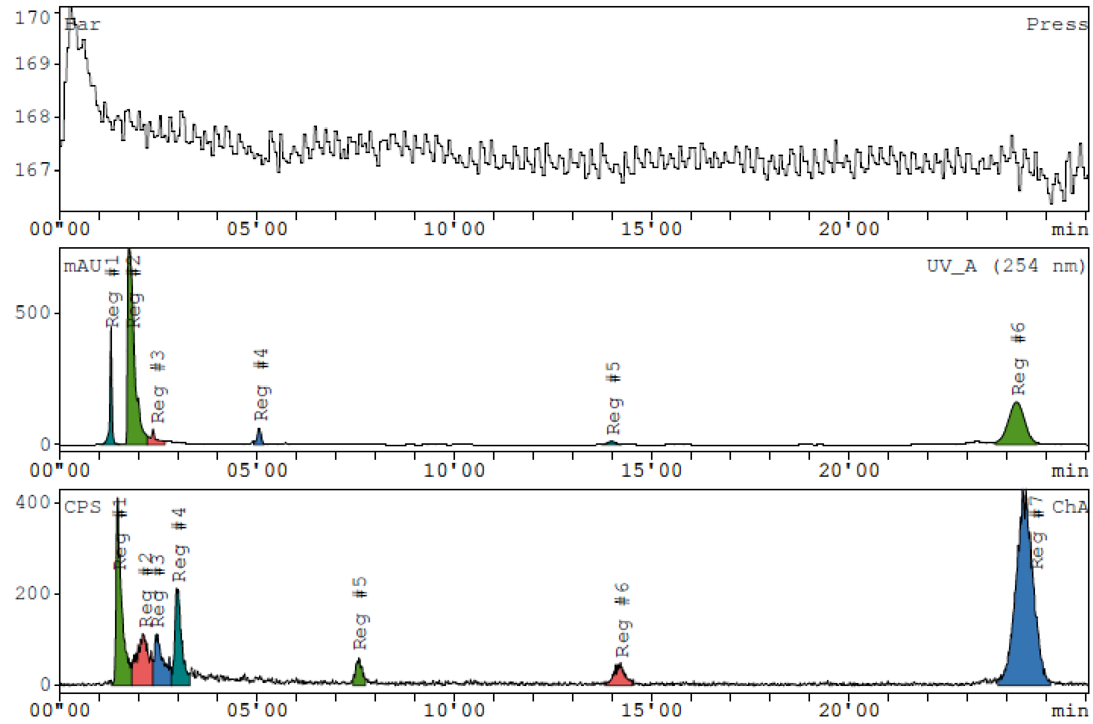

Measurement:	170222-B-Co injection, injection : 17.02.2022 14:47
Method:	LUNAPFP Mudasir AcN_H2O 50_50 from: 17.12.2020 09:30
MeCN:H2O =50:50. Flow rate = 1,5 mL/min. Injected volume = 10 uL.	
Radio detector: raytest Gabi Star	Serial Nr.: #30745 raytest GINA star 20.04.09 Firmware V4.8
Software Version: 5.9, Service Pack 8, Build 5076	

ChA

Substance	R/T s	Type	Area Counts	%Area %
Reg #1	01'29	DD(M	4034,57	16,18
Reg #2	02'08	DD(M	2384,31	9,56
Reg #3	02'27	DD(M	1800,22	7,22
Reg #4	02'59	DD(M	2495,56	10,01
Reg #5	07'34	DD(M	680,05	2,73
Reg #6	14'12	DD(M	912,05	3,66
Reg #7	24'28	DD(M	12627,76	50,64
Sum in ROI			24934,52	100,00
Area (total)			31961,63	
Ext. BKG			0,00 CPS	

Figure S119: Analytical HPLC chromatogram from screening, top channel = UV, bottom channel = activity. HPLC chromatogram of 1-(2,2-difluoro-2-(fluoro-^{18}F)ethyl)-4-(2-isopropoxyphenoxy)piperidine (**[^{18}F]11)** and 4-(2-isopropoxyphenoxy)-1-(2,2,2-trifluoroethyl)piperidines (reference) together as a co-injection (5 uL + 5 uL) 100 µL organic phase in 1 mL MeCN: H_2O =50:50. **(**MeCN: H_2O =50:50. Flow rate = 1.5 mL/min. Injected volume = 10 uL).

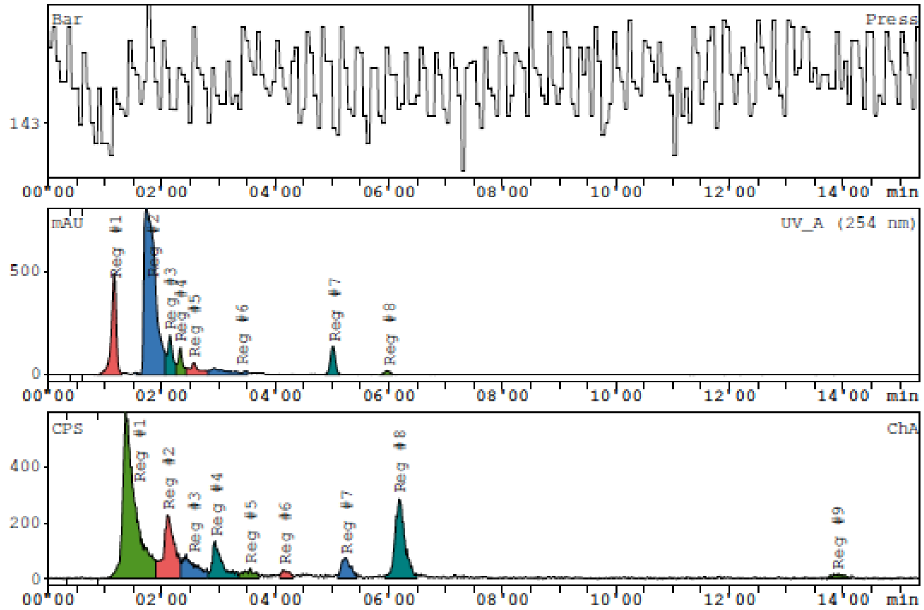

Measurement:	160721-D, injection : 16.07.2021 16:10
Method:	LUNAPFP Mudasir AcN_H2O 50_50 from: 17.12.2020 09:30
CH3CN:H2O=50:50, Flow rate 1,5 mL/min, injection 10 micro L	
Radio detector: raytest Gabi Star	Serial Nr.: #30745 raytest GINA star 20.04.09 Firmware V4.8
Software Version: 5.9, Service Pack 8, Build 5076	

ChA

Substance	R/T s	Type	Area Counts	%Area %
Reg #1	01'23	DD(M	8892,315	44,97
Reg #2	02'07	DD(M	2989,478	15,12
Reg #3	02'34	DD(M	1390,011	7,03
Reg #4	02'57	DD(M	1541,571	7,80
Reg #5	03'33	DD(M	418,893	2,12
Reg #6	04'10	DD(M	269,262	1,36
Reg #7	05'14	DD(M	794,576	4,02
Reg #8	06'11	DD(M	3300,747	16,69
Reg #9	13'53	DD(M	177,004	0,90
Sum in ROI			19773,857	100,00
Area (total)			21208,135	
BKG1			2,6250	
Remainder			1434,28	6,76

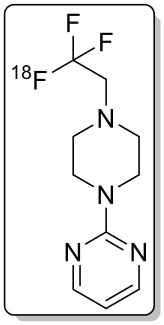

UV_A (254 nm)

Substance	R/T s	Type	Area mAU*s	%Area %
Reg #1	01'10	DD(M	2862,36	15,37
Reg #2	01'45	DD(M	11275,51	60,54
Reg #3	02'09	DD(M	1238,26	6,65

Figure S120: Analytical HPLC chromatogram from screening, top channel = UV, bottom channel = activity. HPLC chromatogram of 2-(4-(2,2-difluoro-2-(fluoro-^{18}F)ethyl)piperazin-1-yl)pyrimidine **([^{18}F]12)**. 100 µL organic phase in 1 mL MeCN: H_2O =50:50. (MeCN: H_2O =50:50. Flow rate = 1.5 mL/min. Injected volume = 10 uL).

Measurement 280922-83B-refernce-repeat Uni Oslo Page 1/2

c:\GINA_NT\LUNAPFP Mudasir AcN_H2O 50_50\280922-83B-refernce-repeat

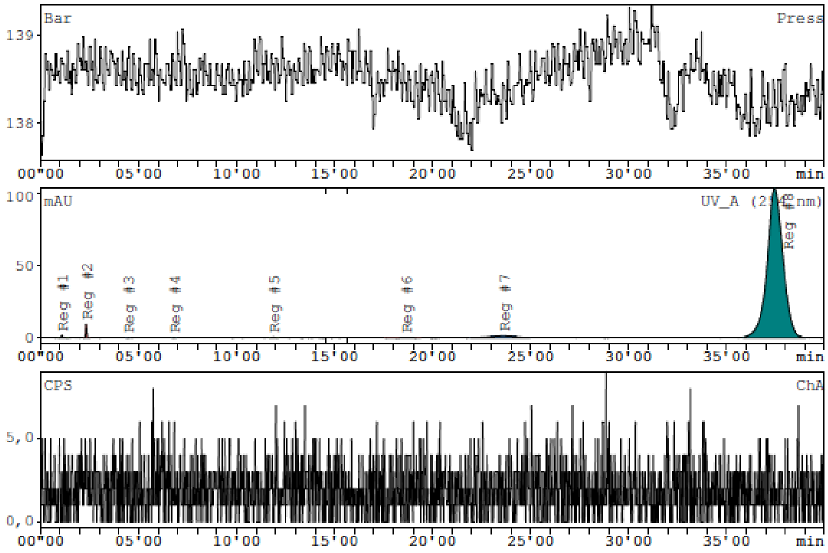

Measurement:	280922-83B-refernce-repeat, injection : 28.09.2022 13:40
Method:	LUNAPFP Mudasir AcN_H2O 50_50 from: 28.09.2022 13:32

MeCN:H2O =50:50. Flow rate = 1,5 mL/min. Injected volume = 10 uL.

Radio detector: raytest Gabi Star Serial Nr.: #30745 raytest GINA star 20.04.09 Firmware V4.8

Software Version: 5.9, Service Pack 8, Build 5076

UV_A (254 nm)

Substance	R/T s	Type	Area mAU*s	%Area %
Reg #1	01'06	DD(M)	10,426	0,17
Reg #2	02'20	DD(M)	32,871	0,55
Reg #3	04'28	DD(M)	1,946	0,03
Reg #4	06'49	DD(M)	1,956	0,03
Reg #5	11'54	DD(M)	7,002	0,12
Reg #6	18'40	DD(M)	14,568	0,24
Reg #7	23'39	DD(M)	141,893	2,36
Reg #8	37'27	DD(M)	5799,781	96,50
Sum in ROI			6010,444	100,00
Area (total)			6078,165	
BKG1			-0,0742	

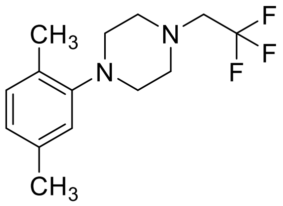

Figure S121: Analytical HPLC chromatogram from screening, top channel = UV, bottom channel = activity. HPLC spectrum of 1-(2,5-dimethylphenyl)-4-(2,2,2-trifluoroethyl)piperazine **(13)** (reference). 1 mg of the sample dissolved in 1 mL MeCN: H_2O =50:50. **(**MeCN: H_2O =50:50. Flow rate = 1.5 mL/min. Injected volume = 10 uL).

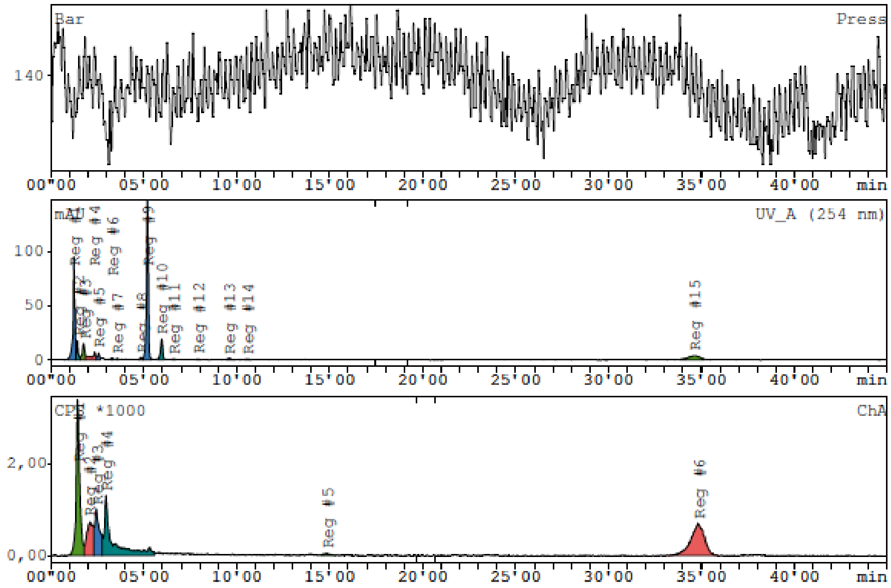

Measurement:	280922-A2, injection :	28.09.2022 15:05
Method:	LUNAPFP Mudasir AcN_H2O 50_50 from:	28.09.2022 14:21
MeCN:H2O =50:50. Flow rate = 1,5 mL/min. Injected volume = 10 uL.		
Radio detector: raytest Gabi Star	Serial Nr.: #30745 raytest GINA star 20.04.09 Firmware V4.8	
Software Version: 5.9, Service Pack 8, Build 5076		

ChA

Substance	R/T s	Type	Area Counts	%Area %
Reg #1	01'26	DD(M)	44278,93	28,96
Reg #2	02'05	DD(M)	16181,11	10,58
Reg #3	02'27	DD(M)	19107,18	12,49
Reg #4	02'58	DD(M)	37112,60	24,27
Reg #5	14'50	DD(M)	1106,50	0,72
Reg #6	34'50	DD(M)	35135,01	22,98
Sum in ROI			152921,33	100,00
Area (total)			164393,96	
BKG1			9,951	
Remainder			11472,63	6,98

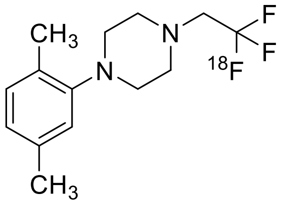

Figure S122: Analytical HPLC chromatogram from screening, top channel = UV, bottom channel = activity. HPLC chromatogram of 1-(2,2-difluoro-2-(fluoro-^{18}F)ethyl)-4-(2,5-dimethylphenyl)piperazine **([^{18}F]13)**. 100 µL organic phase in 1 mL MeCN: H_2O =50:50. **(**MeCN: H_2O =50:50. Flow rate = 1.5 mL/min. Injected volume = 10 uL).

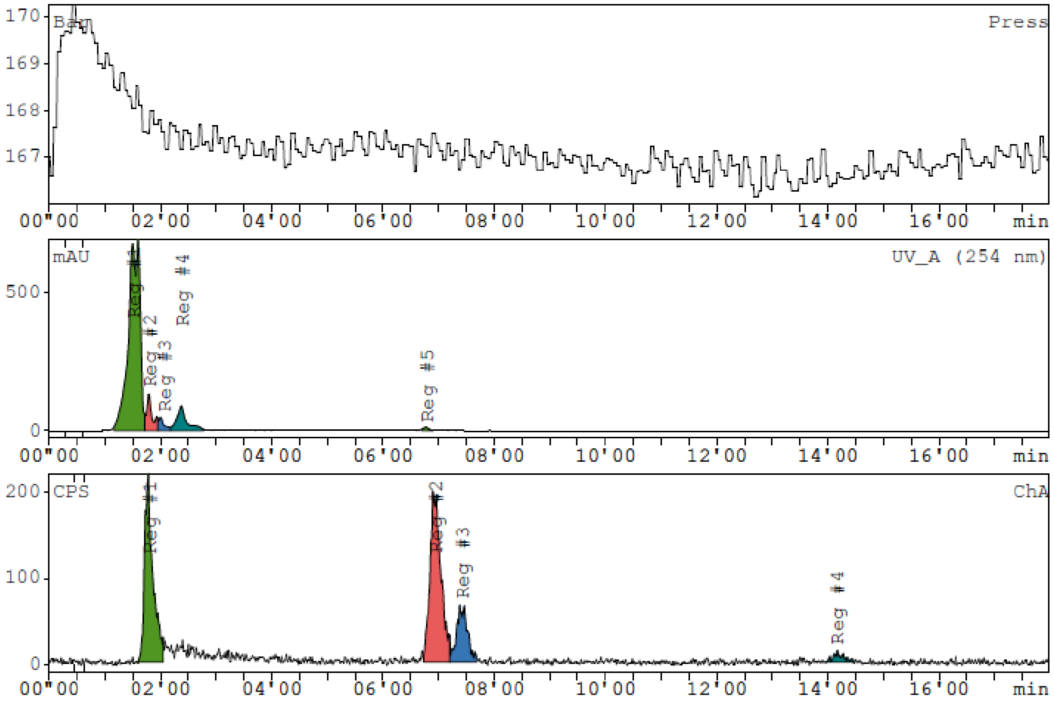

Measurement:	170222-D1, injection : 17.02.2022 16:21
Method:	LUNAPFP Mudasir AcN_H2O 50_50 from: 17.12.2020 09:30
MeCN:H2O =50:50. Flow rate = 1,5 mL/min. Injected volume = 10 uL.	
Radio detector: raytest Gabi Star	Serial Nr.: #30745 raytest GINA star 20.04.09 Firmware V4.8
Software Version: 5.9, Service Pack 8, Build 5076	

ChA

Substance	R/T s	Type	Area Counts	%Area %
Reg #1	01'46	DD(M	2150,756	36,95
Reg #2	06'56	DD(M	2626,870	45,13
Reg #3	07'25	DD(M	904,893	15,55
Reg #4	14'09	DD(M	138,367	2,38
Sum in ROI			5820,885	100,00
Area (total)			7039,738	
BKG1			3,4546	
Remainder			1218,85	17,31

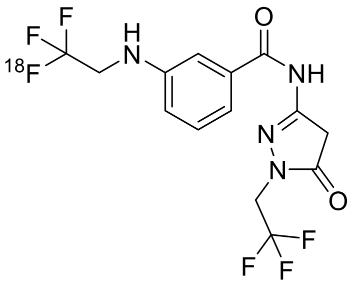

Figure S123: Analytical HPLC chromatogram from screening, top channel = UV, bottom channel = activity. HPLC chromatogram of 3-((2,2-difluoro-2-(fluoro-^{18}F)ethyl)amino)-N-(5-oxo-1-(2,2,2-trifluoroethyl)-4,5-dihydro-1H-pyrazol-3-yl)benzamide **([^{18}F]14)**. 100 µL organic phase in 1 mL MeCN: H_2O =50:50. **(**MeCN: H_2O =50:50. Flow rate = 1.5 mL/min. Injected volume = 10 uL).

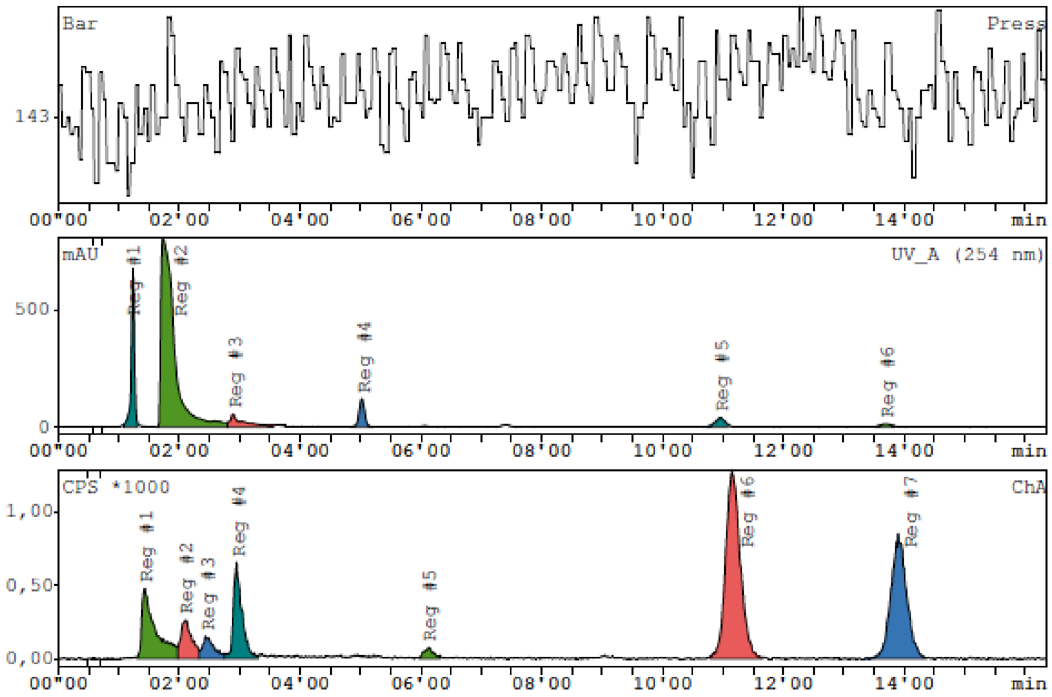

Measurement: 160721-A3, injection : 16.07.2021 13:11
Method: LUNAPFP Mudasir AcN_H2O 50_50 from: 17.12.2020 09:30
CH3CN:H2O=50:50, Flow rate 1,5 mL/min, injection 10 micro L
Radio detector: raytest Gabi Star Serial Nr.: #30745 raytest GINA star 20.04.09 Firmware V4.8
Software Version: 5.9, Service Pack 8, Build 5076

ChA

Substance	R/T s	Type	Area Counts	%Area %
Reg #1	01'26	DD(M	7382,29	13,31
Reg #2	02'06	DD(M	3241,01	5,84
Reg #3	02'28	DD(M	1936,33	3,49
Reg #4	02'57	DD(M	6216,03	11,20
Reg #5	06'08	DD(M	763,07	1,38
Reg #6	11'10	DD(M	21093,04	38,02
Reg #7	13'54	DD(M	14852,75	26,77
Sum in ROI			55484,52	100,00
Area (total)			58787,63	
BKG1			4,000	
Remainder			3303,11	5,62

Figure S124: Analytical HPLC chromatogram from screening, top channel = UV, bottom channel = activity. HPLC chromatogram of 1-(2,2-difluoro-2-(fluoro-^{18}F)ethyl)-4-(4-methoxyphenyl)piperazine **([^{18}F]7b)**. **[^{18}F]1b**, 1-(4-methoxyphenyl)piperazine, DMSO, Cs_2CO_3, 130°C, 40 min. 100 µL organic phase in 1 mL MeCN: H_2O =50:50. **(**MeCN: H_2O =50:50. Flow rate = 1.5 mL/min. Injected volume = 10 uL).

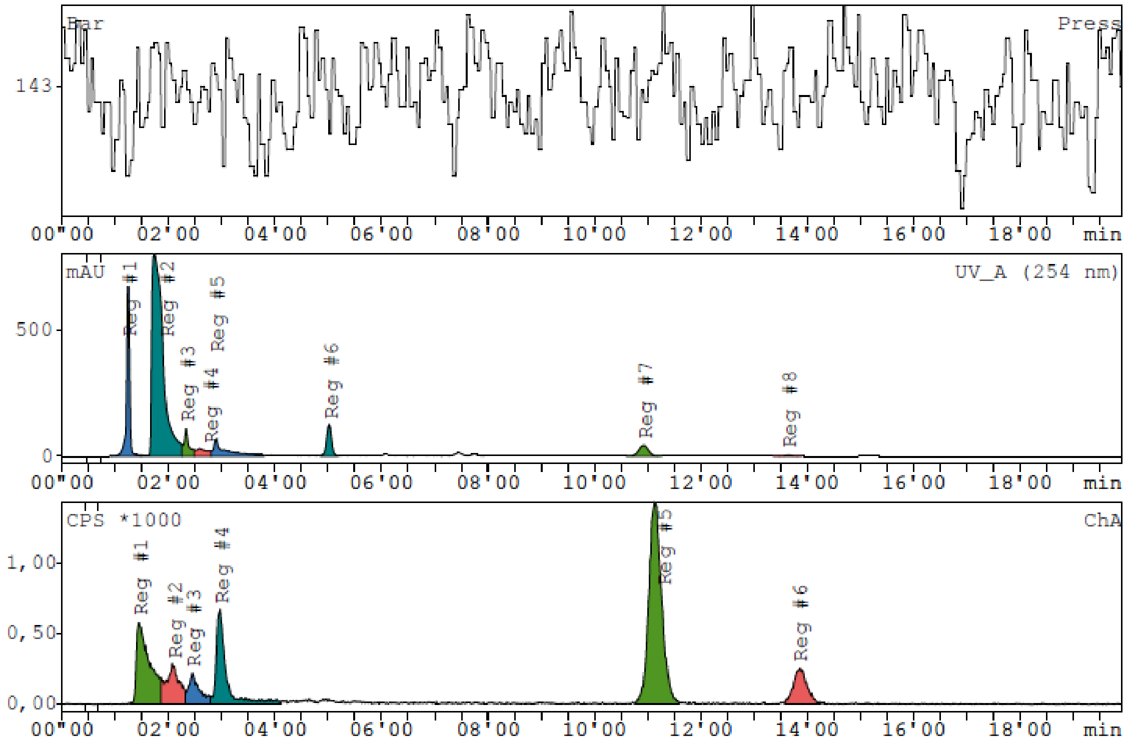

Measurement:	160721-B3, injection : 16.07.2021 13:51
Method:	LUNAPFP Mudasir AcN_H2O 50_50 from: 17.12.2020 09:30
CH3CN:H2O=50:50, Flow rate 1,5 mL/min, injection 10 micro L	
Radio detector: raytest Gabi Star	Serial Nr.: #30745 raytest GINA star 20.04.09 Firmware V4.8
Software Version: 5.9, Service Pack 8, Build 5076	

ChA

Substance	R/T s	Type	Area Counts	%Area %
Reg #1	01'27	DD(M	9849,67	18,21
Reg #2	02'05	DD(M	4808,13	8,89
Reg #3	02'27	DD(M	3034,68	5,61
Reg #4	02'58	DD(M	8340,34	15,42
Reg #5	11'08	DD(M	23622,13	43,67
Reg #6	13'51	DD(M	4437,86	8,20
Sum in ROI			54092,81	100,00
Area (total)			58745,79	
BKG1			2,571	
Remainder			4652,99	7,92

Figure S125: Analytical HPLC chromatogram from screening, top channel = UV, bottom channel = activity. HPLC chromatogram of 1-(2,2-difluoro-2-(fluoro-^{18}F)ethyl)-4-(4-methoxyphenyl)piperazine **([^{18}F]7b)**. **[^{18}F]1b**, 1-(4-methoxyphenyl)piperazine, DMSO, Cs_2CO_3, 140°C, 40 min. 100 µL organic phase in 1 mL MeCN: H_2O =50:50. **(**MeCN: H_2O =50:50. Flow rate = 1.5 mL/min. Injected volume = 10 uL).

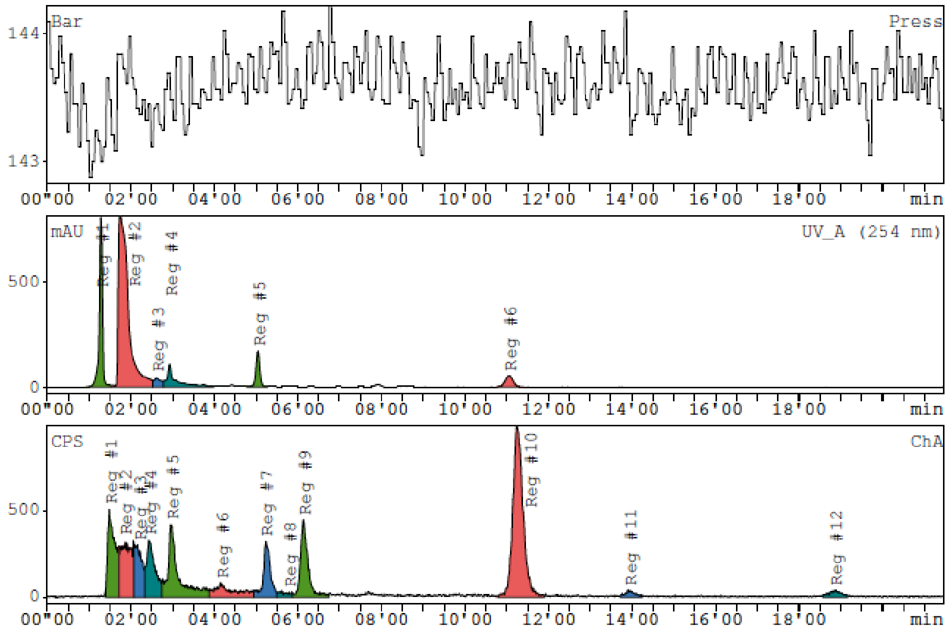

Measurement:	150721-I, injection : 15.07.2021 15:54
Method:	LUNAPFP Mudasir AcN_H2O 50_50 from: 17.12.2020 09:30
CH3CN:H2O=50:50, Flow rate 1,5 mL/min, injection 10 micro L	
Radio detector: raytest Gabi Star	Serial Nr.: #30745 raytest GINA star 20.04.09 Firmware V4.8
Software Version: 5.9, Service Pack 8, Build 5076	

ChA

Substance	R/T s	Type	Area Counts	%Area %
Reg #1	01'30	DD(M	6175,64	10,53
Reg #2	01'52	DD(M	6177,85	10,53
Reg #3	02'12	DD(M	3725,63	6,35
Reg #4	02'27	DD(M	4770,73	8,13
Reg #5	02'58	DD(M	7176,31	12,23
Reg #6	04'10	DD(M	2522,18	4,30
Reg #7	05'15	DD(M	3800,24	6,48
Reg #8	05'47	DD(M	528,02	0,90
Reg #9	06'08	DD(M	5320,29	9,07
Reg #10	11'15	DD(M	17060,61	29,08
Reg #11	13'56	DD(M	618,02	1,05
Reg #12	18'51	DD(M	788,03	1,34
Sum in ROI			58663,56	100,00
Area (total)			64434,65	
Ext. BKG			0,00 CPS	

Figure S126: Analytical HPLC chromatogram from screening, top channel = UV, bottom channel = activity. HPLC chromatogram of 1-(2,2-difluoro-2-(fluoro-^{18}F)ethyl)-4-(4-methoxyphenyl)piperazine **([^{18}F]7b)**. **1b**, 1-(4-methoxyphenyl)piperazine, DMSO, Cs_2CO_3, 150°C, 40 min. 100 µL organic phase in 1 mL MeCN: H_2O =50:50. **(**MeCN: H_2O =50:50. Flow rate = 1.5 mL/min. Injected volume = 10 uL).

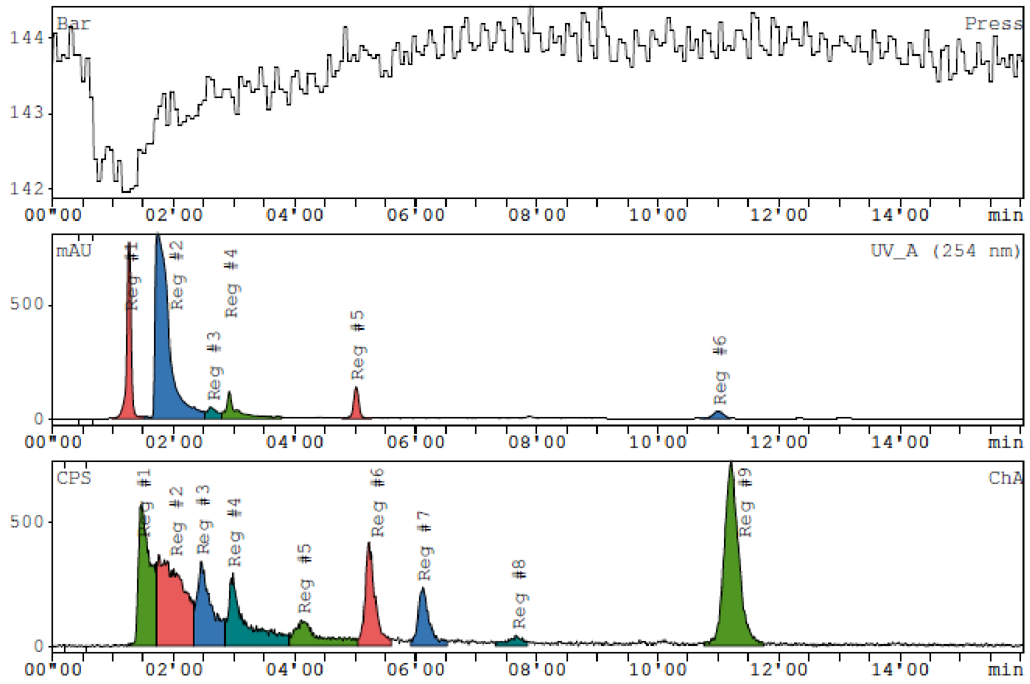

Measurement:	150721-J, injection :	15.07.2021 15:37
Method:	LUNAPFP Mudasir AcN_H2O 50_50 from:	17.12.2020 09:30

CH3CN:H2O=50:50, Flow rate 1,5 mL/min, injection 10 micro L
Radio detector: raytest Gabi Star Serial Nr.: #30745 raytest GINA star 20.04.09 Firmware V4.8
Software Version: 5.9, Service Pack 8, Build 5076

ChA

**Substance**	**R/T s**	**Type**	**Area Counts**	**%Area %**
Reg #1	01'29	DD(M	7543,68	14,24
Reg #2	02'01	DD(M	10300,40	19,44
Reg #3	02'28	DD(M	5677,07	10,72
Reg #4	02'58	DD(M	5822,64	10,99
Reg #5	04'08	DD(M	3017,76	5,70
Reg #6	05'14	DD(M	4766,54	9,00
Reg #7	06'07	DD(M	2991,92	5,65
Reg #8	07'41	DD(M	666,30	1,26
Reg #9	11'13	DD(M	12192,07	23,01
Sum in ROI			52978,38	100,00
Area (total)			56893,16	
BKG1			1,773	
Remainder			3914,78	6,88

Figure S127: Analytical HPLC chromatogram from screening, top channel = UV, bottom channel = activity. HPLC chromatogram of 1-(2,2-difluoro-2-(fluoro-^{18}F)ethyl)-4-(4-methoxyphenyl)piperazine **([^{18}F]7b)**. **[^{18}F]1b**, 1-(4-methoxyphenyl)piperazine, DMSO, Cs_2CO_3, 170°C, 40 min. 100 µL organic phase in 1 mL MeCN: H_2O =50:50. **(**MeCN: H_2O =50:50. Flow rate = 1.5 mL/min. Injected volume = 10 uL).

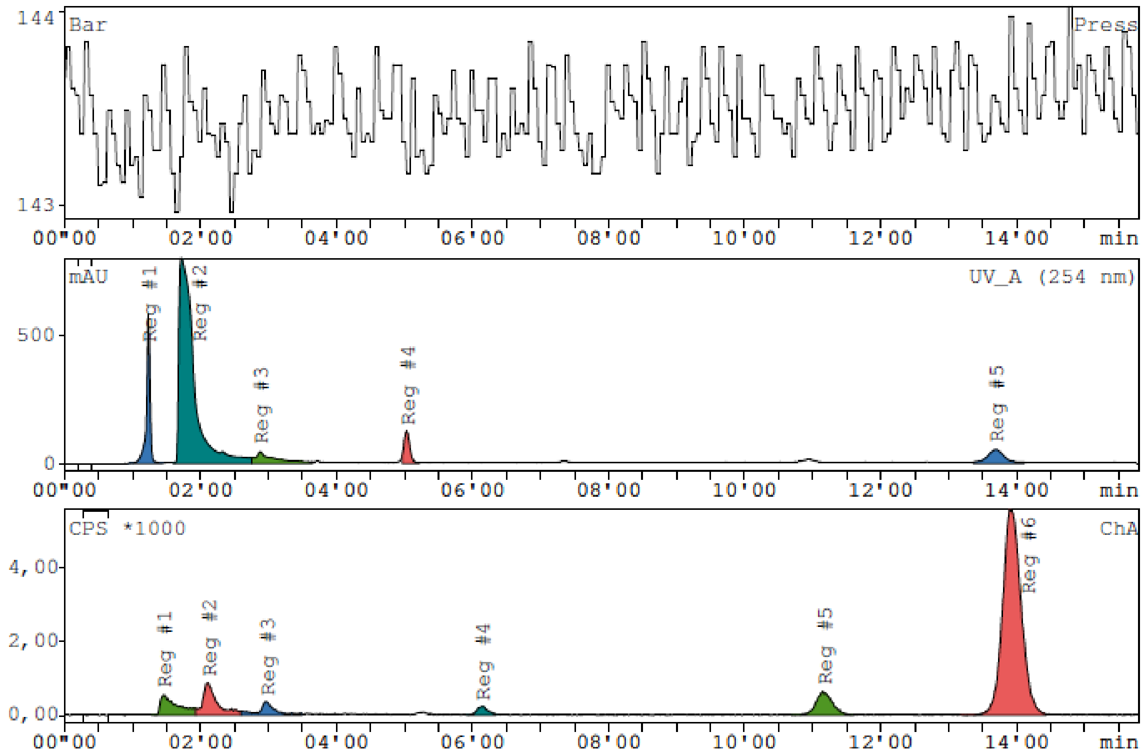

Measurement: 150721-F, injection : 15.07.2021 13:11	
Method: LUNAPFP Mudasir AcN_H2O 50_50 from: 17.12.2020 09:30	
CH3CN:H2O=50:50, Flow rate 1,5 mL/min, injection 10 micro L	
Radio detector: raytest Gabi Star	Serial Nr.: #30745 raytest GINA star 20.04.09 Firmware V4.8
Software Version: 5.9, Service Pack 8, Build 5076	

ChA

Substance	R/T s	Type	Area Counts	%Area %
Reg #1	01'28	DD(M	9509,6	6,49
Reg #2	02'07	DD(M	12629,1	8,63
Reg #3	02'58	DD(M	5945,2	4,06
Reg #4	06'08	DD(M	2702,3	1,85
Reg #5	11'10	DD(M	10708,7	7,31
Reg #6	13'55	DD(M	104923,5	71,66
Sum in ROI			146418,5	100,00
Area (total)			154877,0	
BKG1			1,86 CPS	
Remainder			8458,54	5,46

Figure S128: Analytical HPLC chromatogram from screening, top channel = UV, bottom channel = activity. HPLC chromatogram of 1-(2,2-difluoro-2-(fluoro-^{18}F)ethyl)-4-(4-methoxyphenyl)piperazine **([^{18}F]7b)**. **[^{18}F]1b**, 1-(4-methoxyphenyl)piperazine, DMSO, Cs_2CO_3, 140°C, 10 min. 100 µL organic phase in 1 mL MeCN: H_2O =50:50. **(**MeCN: H_2O =50:50. Flow rate = 1.5 mL/min. Injected volume = 10 uL).

Measurement 150721-G Uni Oslo Page 1/2
c:\GINA_NT\LUNAPFP Mudasir AcN_H2O 50_50\150721-G

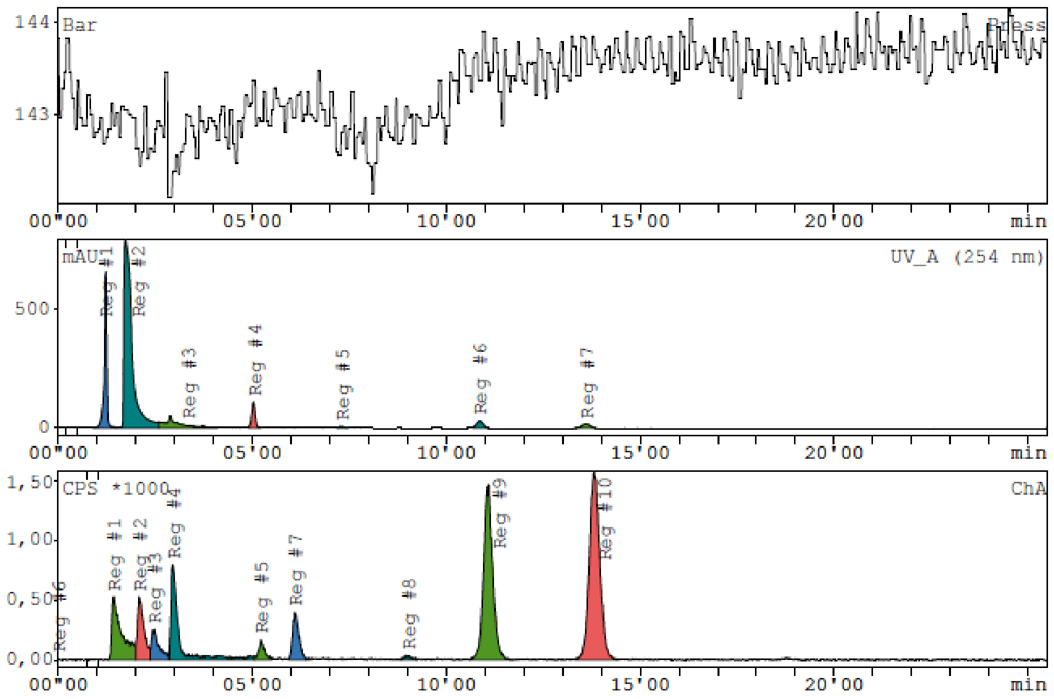

Measurement:	150721-G, injection : 15.07.2021 13:28
Method:	LUNAPFP Mudasir AcN_H2O 50_50 from: 17.12.2020 09:30
CH3CN:H2O=50:50, Flow rate 1,5 mL/min, injection 10 micro L	
Radio detector: raytest Gabi Star	Serial Nr.: #30745 raytest GINA star 20.04.09 Firmware V4.8
Software Version: 5.9, Service Pack 8, Build 5076	

ChA

Substance	R/T s	Type	Area Counts	%Area %
Reg #1	01'26	DD(M	9051,62	10,31
Reg #2	02'06	DD(M	5899,54	6,72
Reg #3	02'28	DD(M	3389,80	3,86
Reg #4	02'58	DD(M	10471,86	11,92
Reg #5	05'14	DD(M	1630,51	1,86
Reg #6	-01"00	DD(M	4,18	0,00
Reg #7	06'06	DD(M	4301,89	4,90
Reg #8	09'01	DD(M	408,26	0,46
Reg #9	11'05	DD(M	23547,21	26,81
Reg #10	13'50	DD(M	29126,27	33,16
Sum in ROI			87831,12	100,00
Area (total)			89518,76	
BKG1			4,177	
Remainder			1687,64	1,89

Figure S129: Analytical HPLC chromatogram from screening, top channel = UV, bottom channel = activity. HPLC chromatogram of 1-(2,2-difluoro-2-(fluoro-^{18}F)ethyl)-4-(4-methoxyphenyl)piperazine **([^{18}F]7b)**. **[^{18}F]1b**, 1-(4-methoxyphenyl)piperazine, DMSO, Cs_2CO_3, 140°C, 30 min. 100 µL organic phase in 1 mL MeCN: H_2O =50:50. **(**MeCN: H_2O =50:50. Flow rate = 1.5 mL/min. Injected volume = 10 uL).

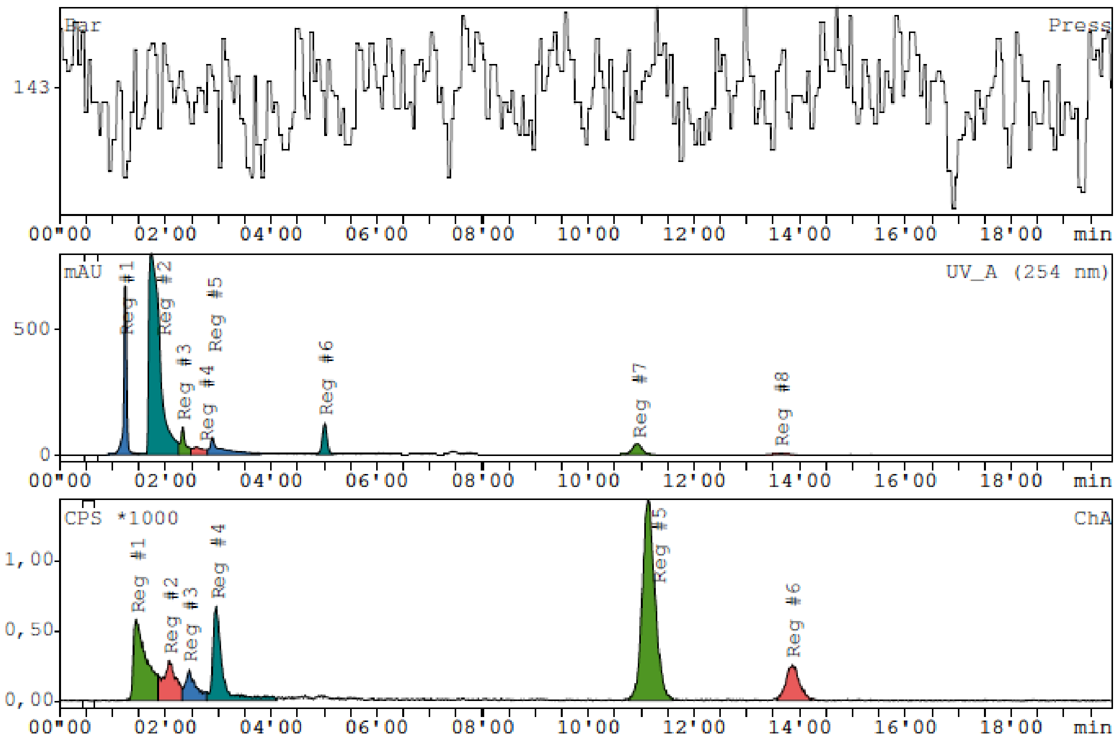

Measurement:	160721-B3, injection :	16.07.2021 13:51	
Method:	LUNAPFP Mudasir AcN_H2O 50_50 from:	17.12.2020 09:30	
CH3CN:H2O=50:50, Flow rate 1,5 mL/min, injection 10 micro L			
Radio detector: raytest Gabi Star	Serial Nr.: #30745 raytest GINA star 20.04.09 Firmware V4.8		
Software Version: 5.9, Service Pack 8, Build 5076			

ChA

Substance	R/T s	Type	Area Counts	%Area %
Reg #1	01'27	DD(M	9849,67	18,21
Reg #2	02'05	DD(M	4808,13	8,89
Reg #3	02'27	DD(M	3034,68	5,61
Reg #4	02'58	DD(M	8340,34	15,42
Reg #5	11'08	DD(M	23622,13	43,67
Reg #6	13'51	DD(M	4437,86	8,20
Sum in ROI			54092,81	100,00
Area (total)			58745,79	
BKG1			2,571	
Remainder			4652,99	7,92

Figure S130: Analytical HPLC chromatogram from screening, top channel = UV, bottom channel = activity. HPLC chromatogram of 1-(2,2-difluoro-2-(fluoro-^{18}F)ethyl)-4-(4-methoxyphenyl)piperazine **([^{18}F]7b)**. **[^{18}F]1b**, 1-(4-methoxyphenyl)piperazine, DMSO, Cs_2CO_3, 140°C, 40 min. 100 µL organic phase in 1 mL MeCN: H_2O =50:50. **(**MeCN: H_2O =50:50. Flow rate = 1.5 mL/min. Injected volume = 10 uL).

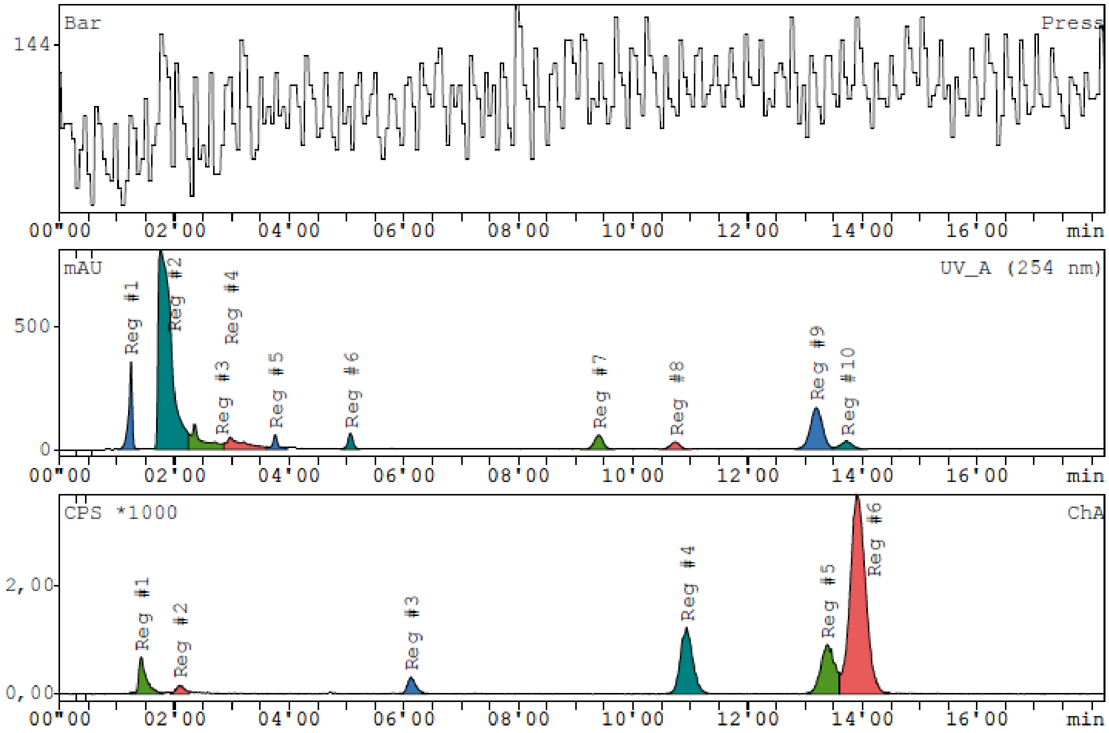

Measurement:	150721-A, injection : 15.07.2021 11:28
Method:	LUNAPFP Mudasir AcN_H2O 50_50 from: 17.12.2020 09:30

CH3CN:H2O=50:50, Flow rate 1,5 mL/min, injection 10 micro L
Radio detector: raytest Gabi Star Serial Nr.: #30745 raytest GINA star 20.04.09 Firmware V4.8
Software Version: 5.9, Service Pack 8, Build 5076

ChA

**Substance**	**R/T s**	**Type**	**Area Counts**	**%Area %**
Reg #1	01'25	DD(M	6317,11	5,58
Reg #2	02'06	DD(M	1682,53	1,49
Reg #3	06'08	DD(M	3225,30	2,85
Reg #4	10'56	DD(M	18004,02	15,90
Reg #5	13'23	DD(M	15199,46	13,42
Reg #6	13'55	DD(M	68817,45	60,77
Sum in ROI			113245,88	100,00
Area (total)			117720,45	
BKG1			1,889	
Remainder			4474,56	3,80

Figure S131: Analytical HPLC chromatogram from screening, top channel = UV, bottom channel = activity. HPLC chromatogram of 1-(2,2-difluoro-2-(fluoro-^{18}F)ethyl)-4-(4-methoxyphenyl)piperazine **([^{18}F]7b)**. **[^{18}F]1b**, 1-(4-methoxyphenyl)piperazine, DMSO, no base, 140°C, 40 min. 100 µL organic phase in 1 mL MeCN: H_2O =50:50. **(**MeCN: H_2O =50:50. Flow rate = 1.5 mL/min. Injected volume = 10 uL).

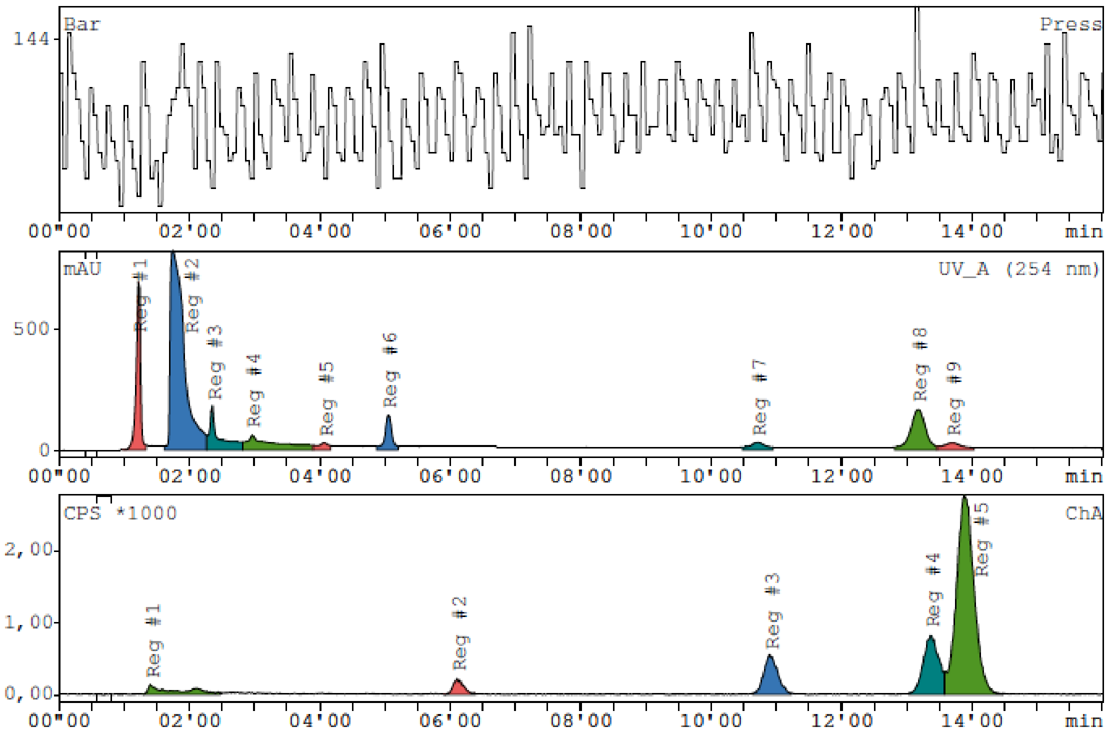

Measurement: 150721-B, injection : 15.07.2021 11:48	
Method: LUNAPFP Mudasir AcN_H2O 50_50 from: 17.12.2020 09:30	
CH3CN:H2O=50:50, Flow rate 1,5 mL/min, injection 10 micro L	
Radio detector: raytest Gabi Star	Serial Nr.: #30745 raytest GINA star 20.04.09 Firmware V4.8
Software Version: 5.9, Service Pack 8, Build 5076	

ChA

Substance	R/T s	Type	Area Counts	%Area %
Reg #1	01'25	DD(M	3656,70	4,62
Reg #2	06'07	DD(M	2362,85	2,98
Reg #3	10'54	DD(M	8056,28	10,18
Reg #4	13'23	DD(M	13409,38	16,94
Reg #5	13'54	DD(M	51675,10	65,28
Sum in ROI			79160,30	100,00
Area (total)			82762,26	
BKG1			2,714	
Remainder			3601,96	4,35

Figure S132: Analytical HPLC chromatogram from screening, top channel = UV, bottom channel = activity. HPLC chromatogram of 1-(2,2-difluoro-2-(fluoro-^{18}F)ethyl)-4-(4-methoxyphenyl)piperazine **([^{18}F]7b)**. **[^{18}F]1b**, 1-(4-methoxyphenyl)piperazine, DMSO, DIPEA (2.3 eq.), 140°C, 40 min. 100 µL organic phase in 1 mL MeCN: H_2O =50:50. **(**MeCN: H_2O =50:50. Flow rate = 1.5 mL/min. Injected volume = 10 uL).

Measurement 150721-C Uni Oslo Page 1/2
c:\GINA_NT\LUNAPFP Mudasir AcN_H2O 50_50\150721-C

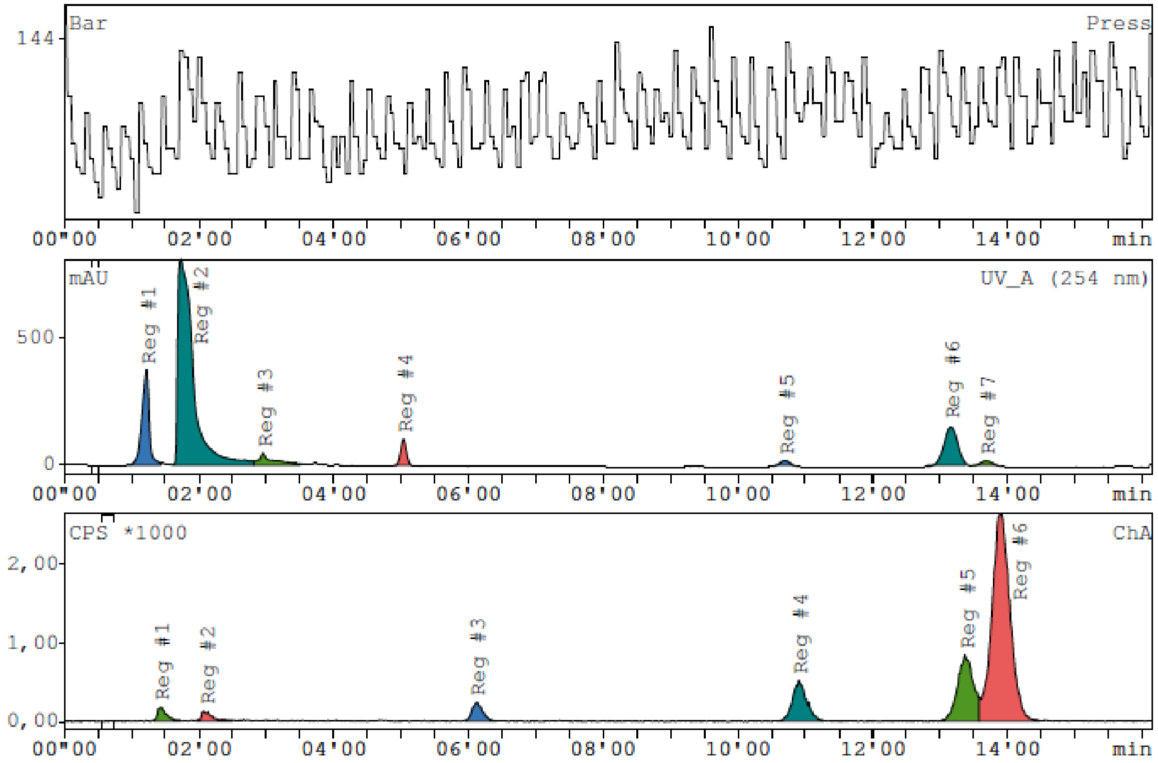

Measurement: 150721-C, injection : 15.07.2021 12:06
Method: LUNAPFP Mudasir AcN_H2O 50_50 from: 17.12.2020 09:30
CH3CN:H2O=50:50, Flow rate 1,5 mL/min, injection 10 micro L
Radio detector: raytest Gabi Star Serial Nr.: #30745 raytest GINA star 20.04.09 Firmware V4.8
Software Version: 5.9, Service Pack 8, Build 5076

ChA

Substance	R/T s	Type	Area Counts	%Area %
Reg #1	01'26	DD(M	1655,14	2,18
Reg #2	02'06	DD(M	1453,41	1,92
Reg #3	06'07	DD(M	2599,93	3,43
Reg #4	10'54	DD(M	7393,18	9,74
Reg #5	13'23	DD(M	13398,43	17,66
Reg #6	13'54	DD(M	49379,98	65,08
Sum in ROI			75880,06	100,00
Area (total)			77708,72	
BKG1			4,400	
Remainder			1828,65	2,35

Figure S133: Analytical HPLC chromatogram from screening, top channel = UV, bottom channel = activity. HPLC chromatogram of 1-(2,2-difluoro-2-(fluoro-^{18}F)ethyl)-4-(4-methoxyphenyl)piperazine **([^{18}F]7b)**. **[^{18}F]1b**, 1-(4-methoxyphenyl)piperazine, DMSO, KH_2PO_4 (2.3 eq.), 140°C, 40 min. 100 µL organic phase in 1 mL MeCN: H_2O =50:50. **(**MeCN: H_2O =50:50. Flow rate = 1.5 mL/min. Injected volume = 10 uL).

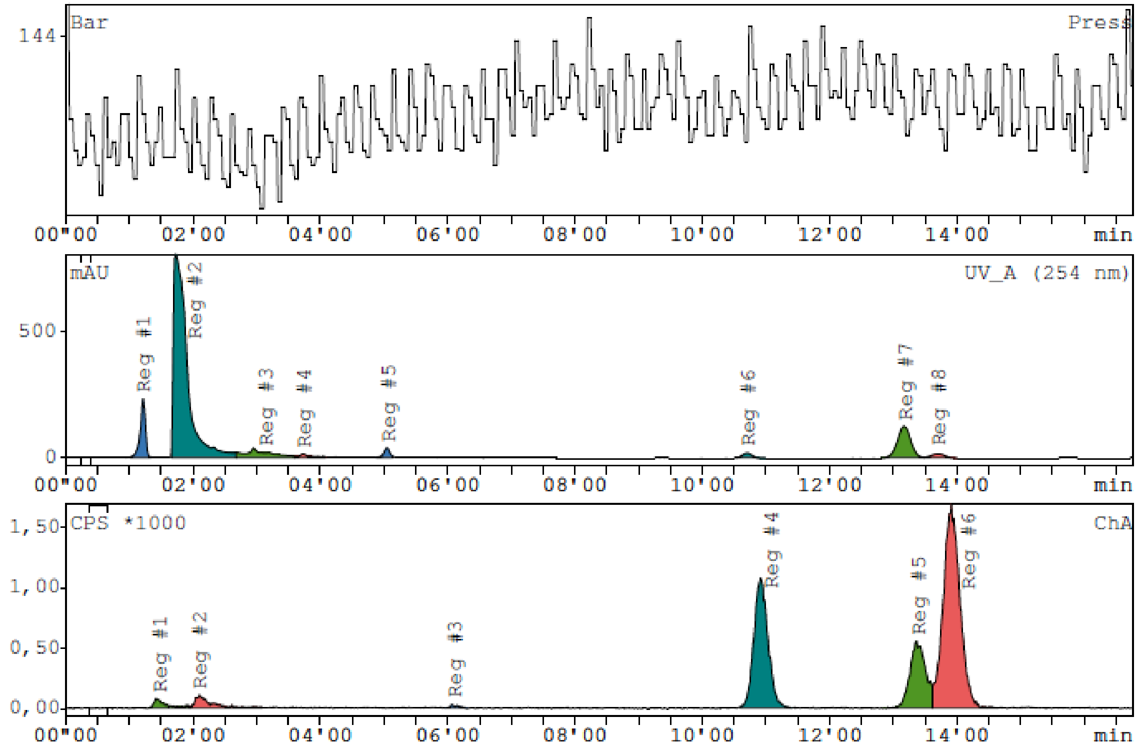

Measurement:	150721-D, injection :	15.07.2021 12:22	
Method:	LUNAPFP Mudasir AcN_H2O 50_50 from:		17.12.2020 09:30
CH3CN:H2O=50:50, Flow rate 1,5 mL/min, injection 10 micro L			
Radio detector: raytest Gabi Star	Serial Nr.: #30745 raytest GINA star 20.04.09 Firmware V4.8		
Software Version: 5.9, Service Pack 8, Build 5076			

ChA

Substance	R/T s	Type	Area Counts	%Area %
Reg #1	01'27	DD(M	1052,97	1,77
Reg #2	02'05	DD(M	1900,19	3,19
Reg #3	06'06	DD(M	239,71	0,40
Reg #4	10'55	DD(M	16377,31	27,53
Reg #5	13'23	DD(M	9344,38	15,71
Reg #6	13'55	DD(M	30573,70	51,39
Sum in ROI			59488,26	100,00
Area (total)			60834,69	
BKG1			2,824	
Remainder			1346,43	2,21

Figure S134: Analytical HPLC chromatogram from screening, top channel = UV, bottom channel = activity. HPLC chromatogram of 1-(2,2-difluoro-2-(fluoro-^{18}F)ethyl)-4-(4-methoxyphenyl)piperazine **([^{18}F]7b)**. **[^{18}F]1b**, 1-(4-methoxyphenyl)piperazine, DMSO, KOTf (2.3 eq.), 140°C, 40 min. 100 µL organic phase in 1 mL MeCN: H_2O =50:50. **(**MeCN: H_2O =50:50. Flow rate = 1.5 mL/min. Injected volume = 10 uL).

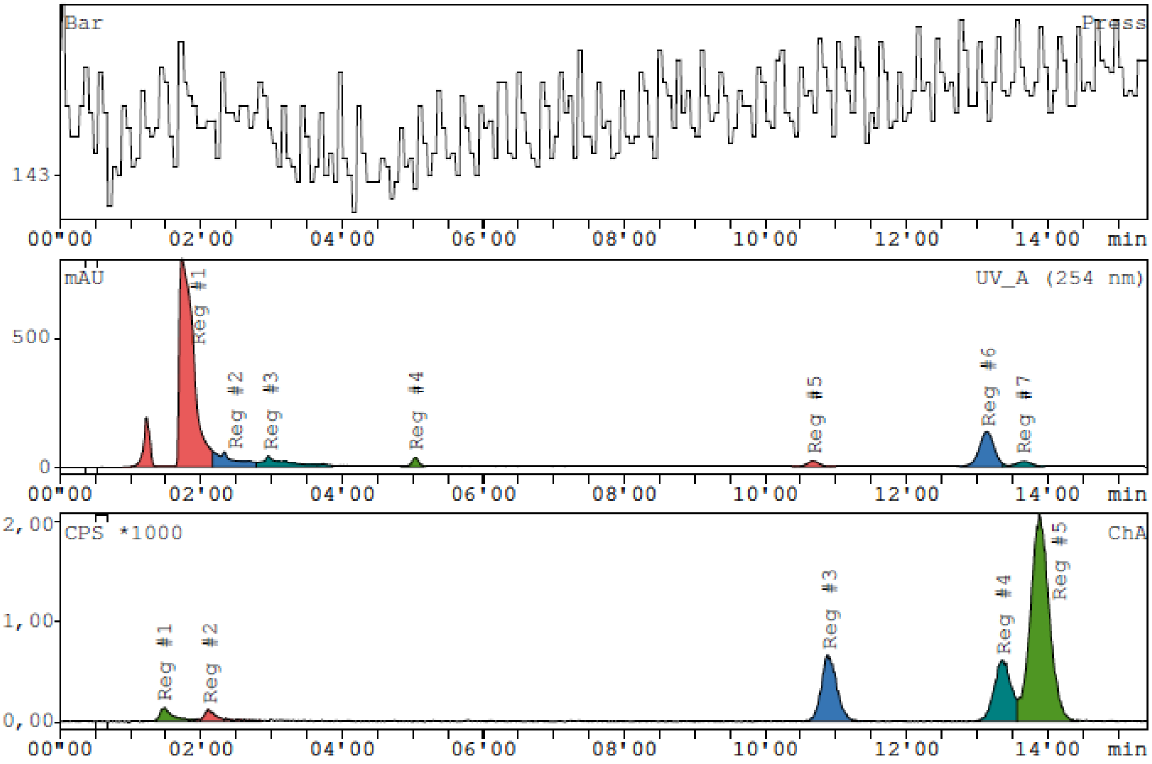

Measurement:	150721-E, injection :	15.07.2021 12:40	
Method:	LUNAPFP Mudasir AcN_H2O 50_50 from:		17.12.2020 09:30
CH3CN:H2O=50:50, Flow rate 1,5 mL/min, injection 10 micro L			
Radio detector: raytest Gabi Star	Serial Nr.: #30745 raytest GINA star 20.04.09 Firmware V4.8		
Software Version: 5.9, Service Pack 8, Build 5076			

ChA

Substance	R/T s	Type	Area Counts	%Area %
Reg #1	01'28	DD(M	1797,70	2,92
Reg #2	02'06	DD(M	1848,20	3,00
Reg #3	10'53	DD(M	9981,06	16,21
Reg #4	13'21	DD(M	9987,50	16,22
Reg #5	13'53	DD(M	37945,99	61,64
Sum in ROI			61560,45	100,00
Area (total)			63241,40	
BKG1			3,273	
Remainder			1680,95	2,66

Figure S135: Analytical HPLC chromatogram from screening, top channel = UV, bottom channel = activity. HPLC chromatogram of 1-(2,2-difluoro-2-(fluoro-^{18}F)ethyl)-4-(4-methoxyphenyl)piperazine **([^{18}F]7b)**. **[^{18}F]1b**, 1-(4-methoxyphenyl)piperazine, DMSO, NaOTf (2.3 eq.), 140°C, 40 min. 100 µL organic phase in 1 mL MeCN: H_2O =50:50. **(**MeCN: H_2O =50:50. Flow rate = 1.5 mL/min. Injected volume = 10 uL).

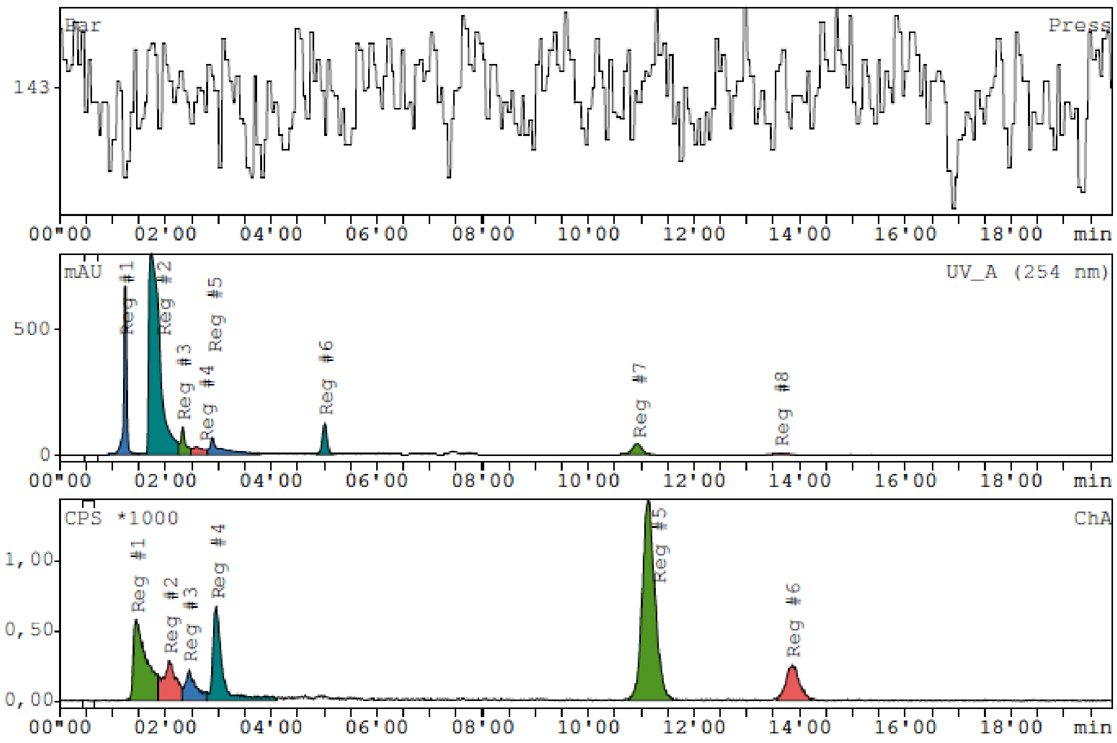

Measurement:	160721-B3, injection : 16.07.2021 13:51
Method:	LUNAPFP Mudasir AcN_H2O 50_50 from: 17.12.2020 09:30
CH3CN:H2O=50:50, Flow rate 1,5 mL/min, injection 10 micro L	
Radio detector: raytest Gabi Star	Serial Nr.: #30745 raytest GINA star 20.04.09 Firmware V4.8
Software Version: 5.9, Service Pack 8, Build 5076	

ChA

Substance	R/T s	Type	Area Counts	%Area %
Reg #1	01'27	DD(M	9849,67	18,21
Reg #2	02'05	DD(M	4808,13	8,89
Reg #3	02'27	DD(M	3034,68	5,61
Reg #4	02'58	DD(M	8340,34	15,42
Reg #5	11'08	DD(M	23622,13	43,67
Reg #6	13'51	DD(M	4437,86	8,20
Sum in ROI			54092,81	100,00
Area (total)			58745,79	
BKG1			2,571	
Remainder			4652,99	7,92

Figure S136: Analytical HPLC chromatogram from screening, top channel = UV, bottom channel = activity. HPLC chromatogram of 1-(2,2-difluoro-2-(fluoro-^{18}F)ethyl)-4-(4-methoxyphenyl)piperazine **([^{18}F]7b)**. **[^{18}F]1b**, 1-(4-methoxyphenyl)piperazine, DMSO, Cs_2CO_3 (2.3 eq.), 140°C, 40 min. 100 µL organic phase in 1 mL MeCN: H_2O =50:50. **(**MeCN: H_2O =50:50. Flow rate = 1.5 mL/min. Injected volume = 10 uL).

Measurement 090621-rxn 1 Uni Oslo Page 1/1
c:\GINA_NT\LUNAPFP Mudasir AcN_H2O 50_50\090621-rxn 1

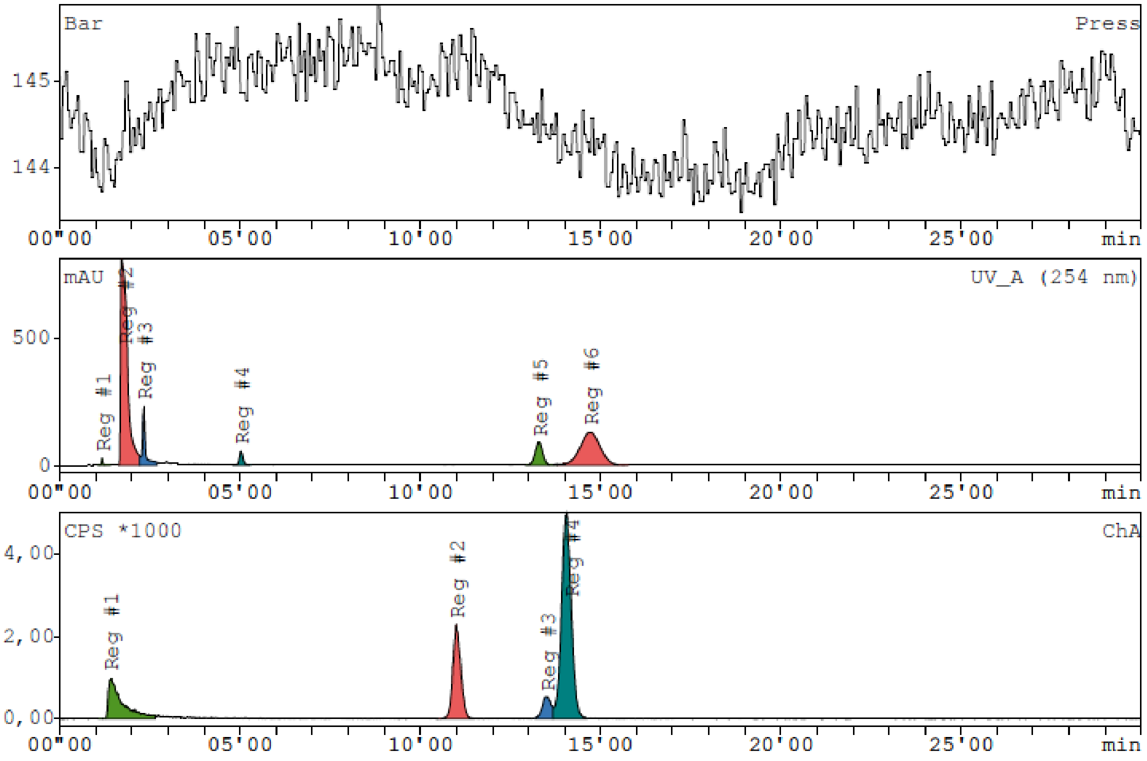

Sample description

Measurement:	090621-rxn 1, injection :	09.06.2021 12:03	
Method:	LUNAPFP Mudasir AcN_H2O 50_50 from:		17.12.2020 09:30
CH3CN:H2O=50:50, Flow rate 1,5 mL/min, injection 10 micro L			
Radio detector: raytest Gabi Star	Serial Nr.: #30745 raytest GINA star 20.04.09 Firmware V4.8		
Software Version: 5.9, Service Pack 8, Build 5076			

Integration ChA

Substance	R/T s	Type	Area Counts	%Area %
Reg #1	01'25	DD(M	26112,33	16,14
Reg #2	11'00	DD(M	34095,32	21,07
Reg #3	13'30	DD(M	8774,78	5,42
Reg #4	14'03	DD(M	92822,66	57,37
Sum in ROI			161805,10	100,00
Area (total)			172264,45	
Ext. BKG			0,00 CPS	

Figure S137: Analytical HPLC chromatogram from screening, top channel = UV, bottom channel = activity. HPLC chromatogram of 1-(2,2-difluoro-2-(fluoro-^{18}F)ethyl)-4-(4-methoxyphenyl)piperazine **([^{18}F]7b)**. **[^{18}F]1b**, 1-(4-methoxyphenyl)piperazine, DMSO, K_2CO_3 (2.3 eq.), 140°C, 40 min. 100 µL organic phase in 1 mL MeCN: H_2O =50:50. **(**MeCN: H_2O =50:50. Flow rate = 1.5 mL/min. Injected volume = 10 uL).

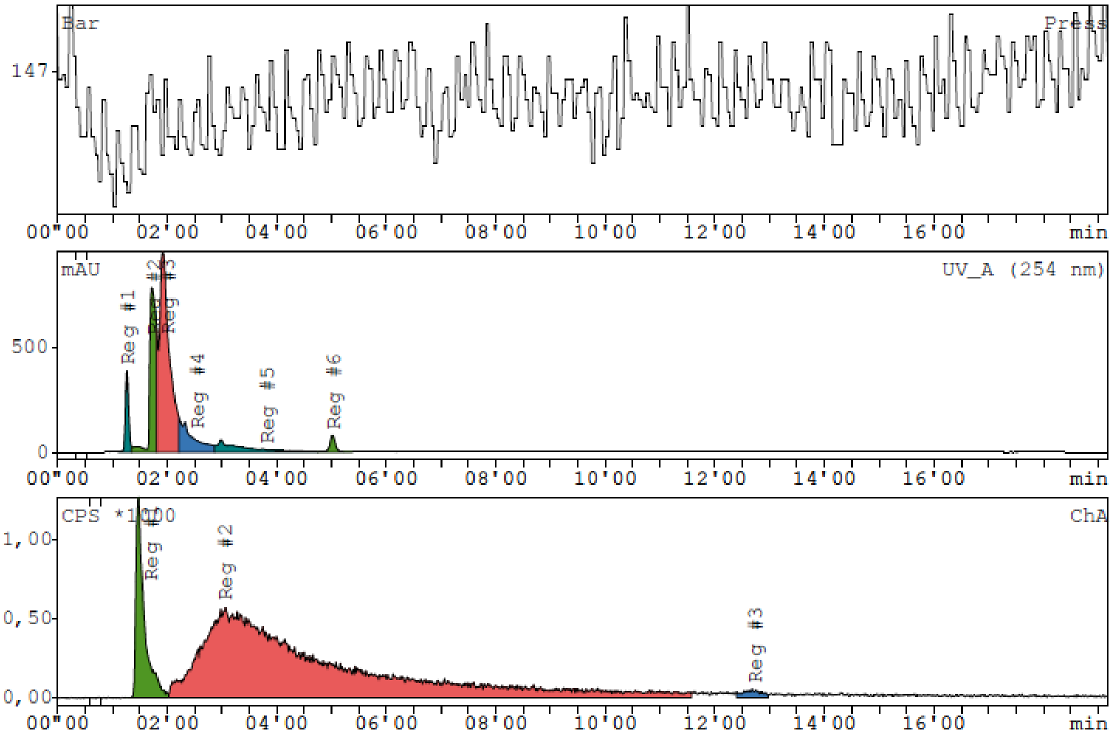

Sample description

Measurement:	220621-A, injection :	22.06.2021 11:27
Method:	LUNAPFP Mudasir AcN_H2O 50_50 from:	17.12.2020 09:30
CH3CN:H2O=50:50, Flow rate 1,5 mL/min, injection 10 micro L		
Radio detector: raytest Gabi Star	Serial Nr.: #30745 raytest GINA star 20.04.09 Firmware V4.8	
Software Version: 5.9, Service Pack 8, Build 5076		

Integration ChA

Substance	R/T s	Type	Area Counts	%Area %
Reg #1	01'29	DD(M	13181,01	12,59
Reg #2	03'03	DD(M	90260,15	86,23
Reg #3	12'43	DD(M	1237,32	1,18
Sum in ROI			104678,49	100,00
Area (total)			112018,55	
BKG1			2,308	
Remainder			7340,07	6,55

Figure S138: Analytical HPLC chromatogram from screening, top channel = UV, bottom channel = activity. HPLC chromatogram of 1-(2,2-difluoro-2-(fluoro-^{18}F)ethyl)-4-(4-methoxyphenyl)piperazine **([^{18}F]7b)**. **[^{18}F]1b**, 1-(4-methoxyphenyl)piperazine, DMF, NaH (2.3 eq.), 120°C, 15 min. 100 µL organic phase in 1 mL MeCN: H_2O =50:50. **(**MeCN: H_2O =50:50. Flow rate = 1.5 mL/min. Injected volume = 10 uL).

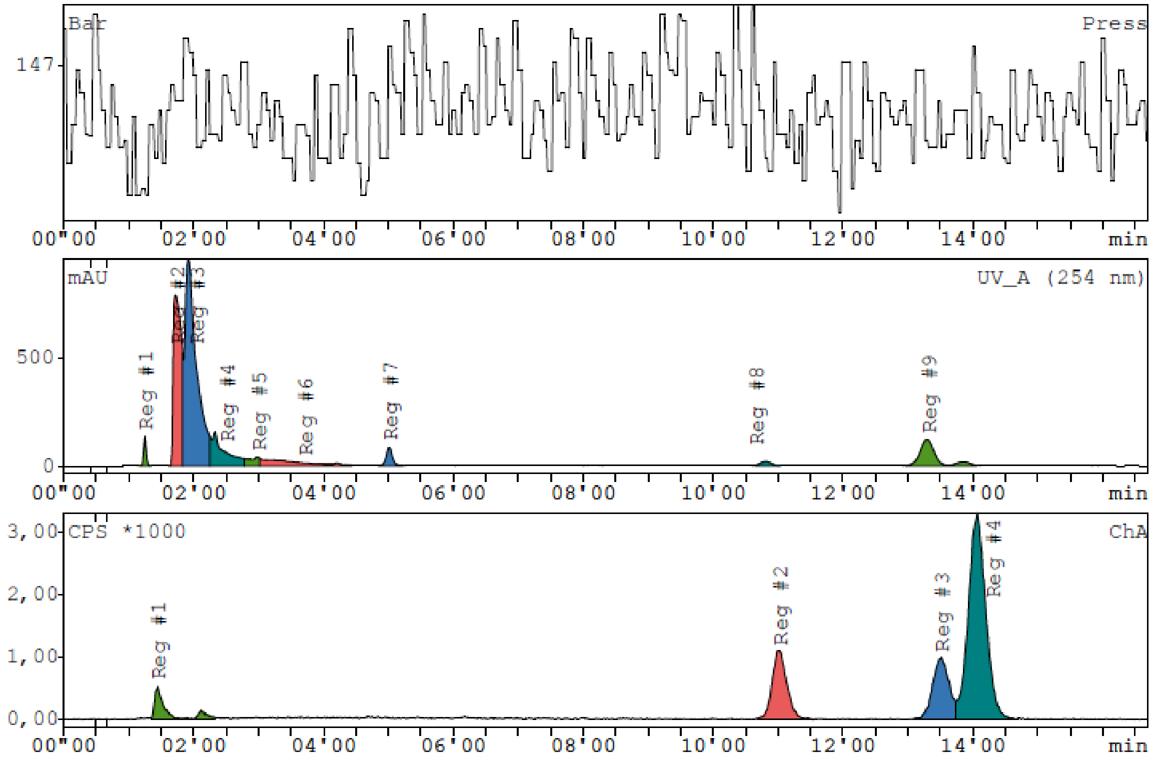

Sample description

Measurement:	220621-B, injection : 22.06.2021 11:48
Method:	LUNAPFP Mudasir AcN_H2O 50_50 from: 17.12.2020 09:30
CH3CN:H2O=50:50, Flow rate 1,5 mL/min, injection 10 micro L	
Radio detector: raytest Gabi Star	Serial Nr.: #30745 raytest GINA star 20.04.09 Firmware V4.8
Software Version: 5.9, Service Pack 8, Build 5076	

Integration ChA

Substance	R/T s	Type	Area Counts	%Area %
Reg #1	01'27	DD(M	5973,47	5,90
Reg #2	11'01	DD(M	17039,84	16,83
Reg #3	13'30	DD(M	16966,95	16,76
Reg #4	14'04	DD(M	61241,23	60,50
Sum in ROI			101221,49	100,00
Area (total)			109381,62	
BKG1			9,700	
Remainder			8160,13	7,46

Figure S139: Analytical HPLC chromatogram from screening, top channel = UV, bottom channel = activity. HPLC chromatogram of 1-(2,2-difluoro-2-(fluoro-^{18}F)ethyl)-4-(4-methoxyphenyl)piperazine **([^{18}F]7b)**. **[^{18}F]1b**, 1-(4-methoxyphenyl)piperazine, DMF, DIPEA (2.3 eq.), 120°C, 15 min. 100 µL organic phase in 1 mL MeCN: H_2O =50:50. **(**MeCN: H_2O =50:50. Flow rate = 1.5 mL/min. Injected volume = 10 uL).

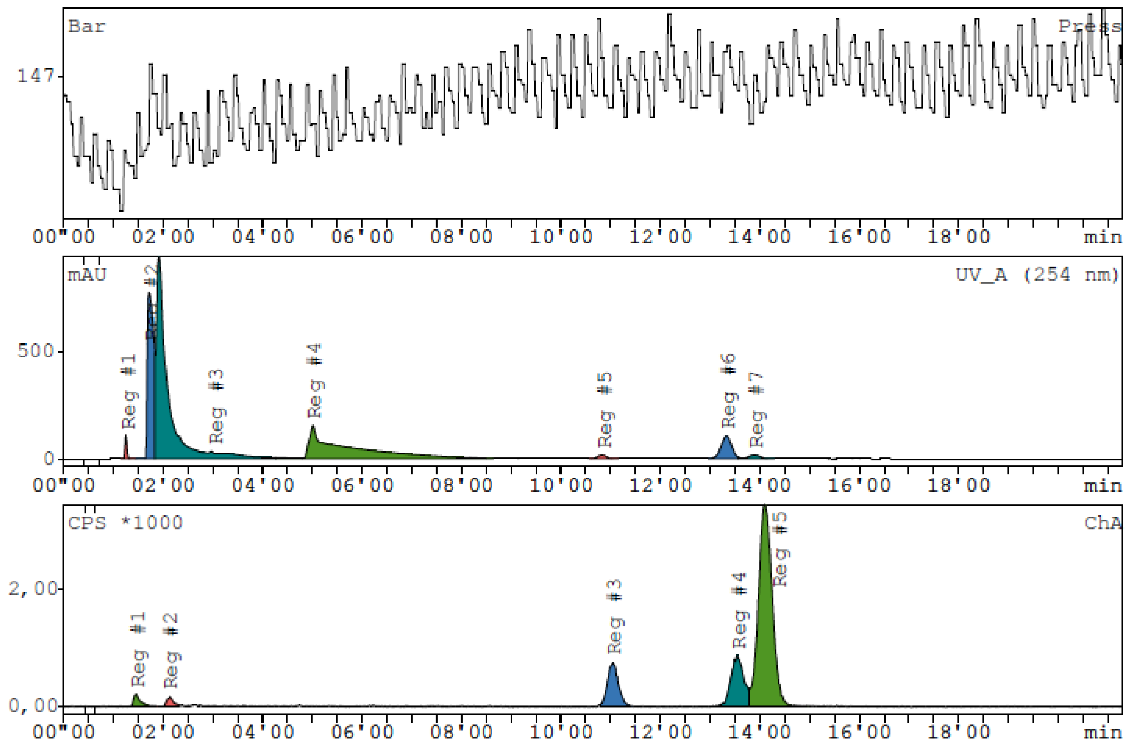

Sample description

Measurement:	220621-C, injection : 22.06.2021 12:05
Method:	LUNAPFP Mudasir AcN_H2O 50_50 from: 17.12.2020 09:30
CH3CN:H2O=50:50, Flow rate 1,5 mL/min, injection 10 micro L	
Radio detector: raytest Gabi Star	Serial Nr.: #30745 raytest GINA star 20.04.09 Firmware V4.8
Software Version: 5.9, Service Pack 8, Build 5076	

Integration ChA

Substance	R/T s	Type	Area Counts	%Area %
Reg #1	01'27	DD(M	1938,48	2,01
Reg #2	02'08	DD(M	1469,29	1,53
Reg #3	11'03	DD(M	11325,90	11,76
Reg #4	13'33	DD(M	15393,06	15,98
Reg #5	14'06	DD(M	66195,95	68,72
Sum in ROI			96322,68	100,00
Area (total)			97150,73	
BKG1			7,000	
Remainder			828,06	0,85

Figure S140: Analytical HPLC chromatogram from screening, top channel = UV, bottom channel = activity. HPLC chromatogram of 1-(2,2-difluoro-2-(fluoro-^{18}F)ethyl)-4-(4-methoxyphenyl)piperazine **([^{18}F]7b)**. **[^{18}F]1b**, 1-(4-methoxyphenyl)piperazine, DMF, pyridine (2.3 eq.), 120°C, 15 min. 100 µL organic phase in 1 mL MeCN: H_2O =50:50. **(**MeCN: H_2O =50:50. Flow rate = 1.5 mL/min. Injected volume = 10 uL).

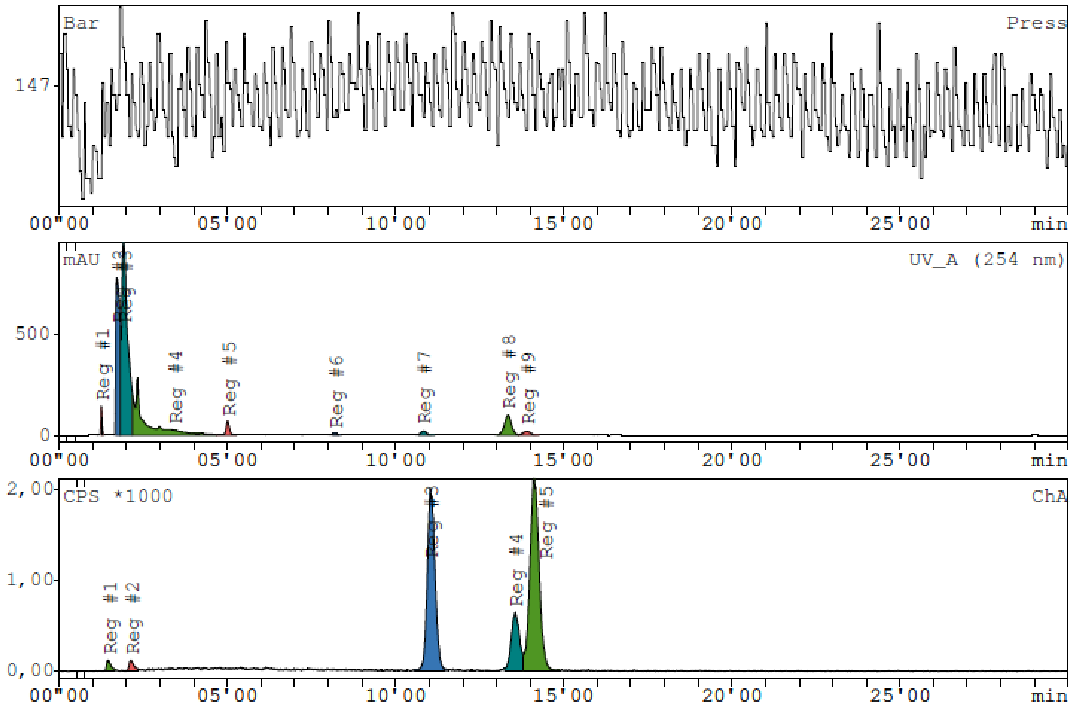

Sample description

Measurement:	220621-D, injection : 22.06.2021 12:29
Method:	LUNAPFP Mudasir AcN_H2O 50_50 from: 17.12.2020 09:30
CH3CN:H2O=50:50, Flow rate 1,5 mL/min, injection 10 micro L	
Radio detector: raytest Gabi Star	Serial Nr.: #30745 raytest GINA star 20.04.09 Firmware V4.8
Software Version: 5.9, Service Pack 8, Build 5076	

Integration ChA

Substance	R/T s	Type	Area Counts	%Area %
Reg #1	01'28	DD(M	993,64	1,19
Reg #2	02'08	DD(M	1128,16	1,35
Reg #3	11'03	DD(M	30849,43	36,83
Reg #4	13'33	DD(M	10834,15	12,94
Reg #5	14'08	DD(M	39951,19	47,70
Sum in ROI			83756,56	100,00
Area (total)			95308,06	
BKG1			3,500	
Remainder			11551,50	12,12

Figure S141: Analytical HPLC chromatogram from screening, top channel = UV, bottom channel = activity. HPLC chromatogram of 1-(2,2-difluoro-2-(fluoro-^{18}F)ethyl)-4-(4-methoxyphenyl)piperazine **([^{18}F]7b)**. **[^{18}F]1b**, 1-(4-methoxyphenyl)piperazine, DMF, Cs_2CO_3 (2.3 eq.), 120°C, 15 min. 100 µL organic phase in 1 mL MeCN: H_2O =50:50. **(**MeCN: H_2O =50:50. Flow rate = 1.5 mL/min. Injected volume = 10 uL).

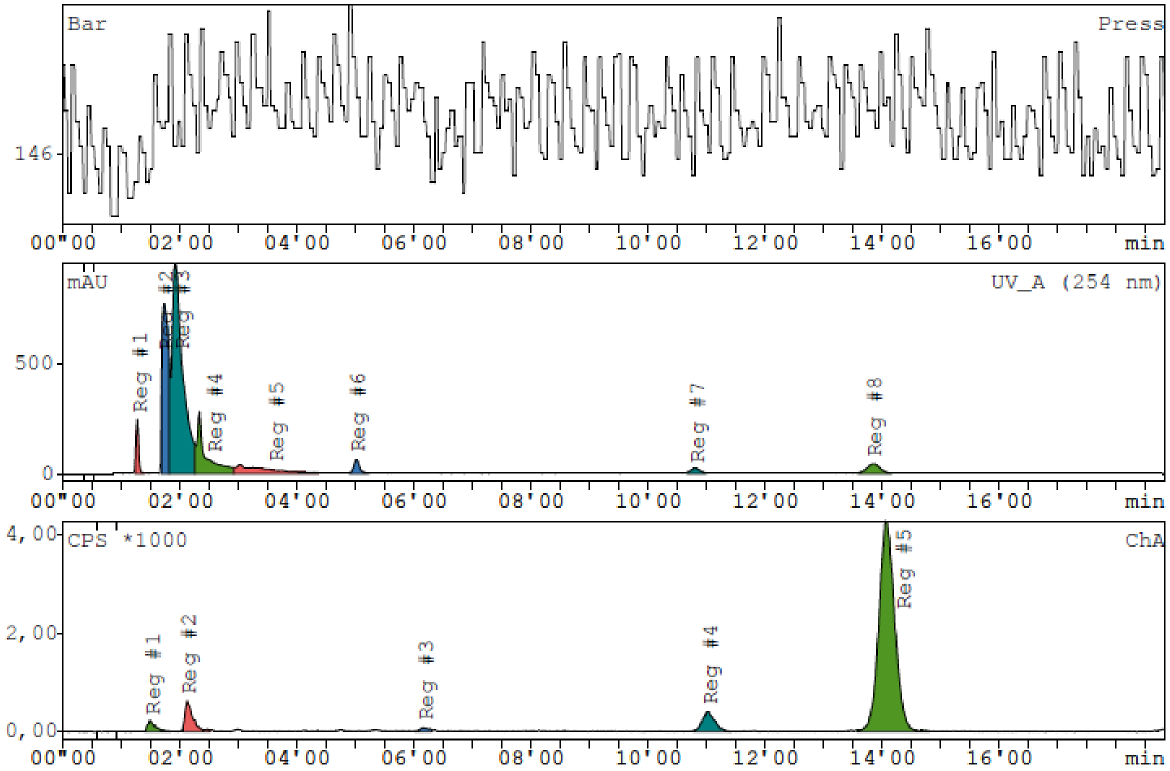

Sample description

Measurement:	220621-E, injection : 22.06.2021 13:46
Method:	LUNAPFP Mudasir AcN_H2O 50_50 from: 17.12.2020 09:30
CH3CN:H2O=50:50, Flow rate 1,5 mL/min, injection 10 micro L	
Radio detector: raytest Gabi Star	Serial Nr.: #30745 raytest GINA star 20.04.09 Firmware V4.8
Software Version: 5.9, Service Pack 8, Build 5076	

Integration ChA

Substance	R/T s	Type	Area Counts	%Area %
Reg #1	01'30	DD(M	1972,92	2,10
Reg #2	02'08	DD(M	5791,77	6,15
Reg #3	06'11	DD(M	829,93	0,88
Reg #4	11'02	DD(M	5955,23	6,33
Reg #5	14'04	DD(M	79594,24	84,55
Sum in ROI			94144,09	100,00
Area (total)			102109,62	
BKG1			2,421	
Remainder			7965,53	7,80

Figure S142: Analytical HPLC chromatogram from screening, top channel = UV, bottom channel = activity. HPLC chromatogram of 1-(2,2-difluoro-2-(fluoro-^{18}F)ethyl)-4-(4-methoxyphenyl)piperazine **([^{18}F]7b)**. **[^{18}F]1b**, 1-(4-methoxyphenyl)piperazine, DMF, K_2CO_3 (2.3 eq.), 120°C, 15 min. 100 µL organic phase in 1 mL MeCN: H_2O =50:50. **(**MeCN: H_2O =50:50. Flow rate = 1.5 mL/min. Injected volume = 10 uL).

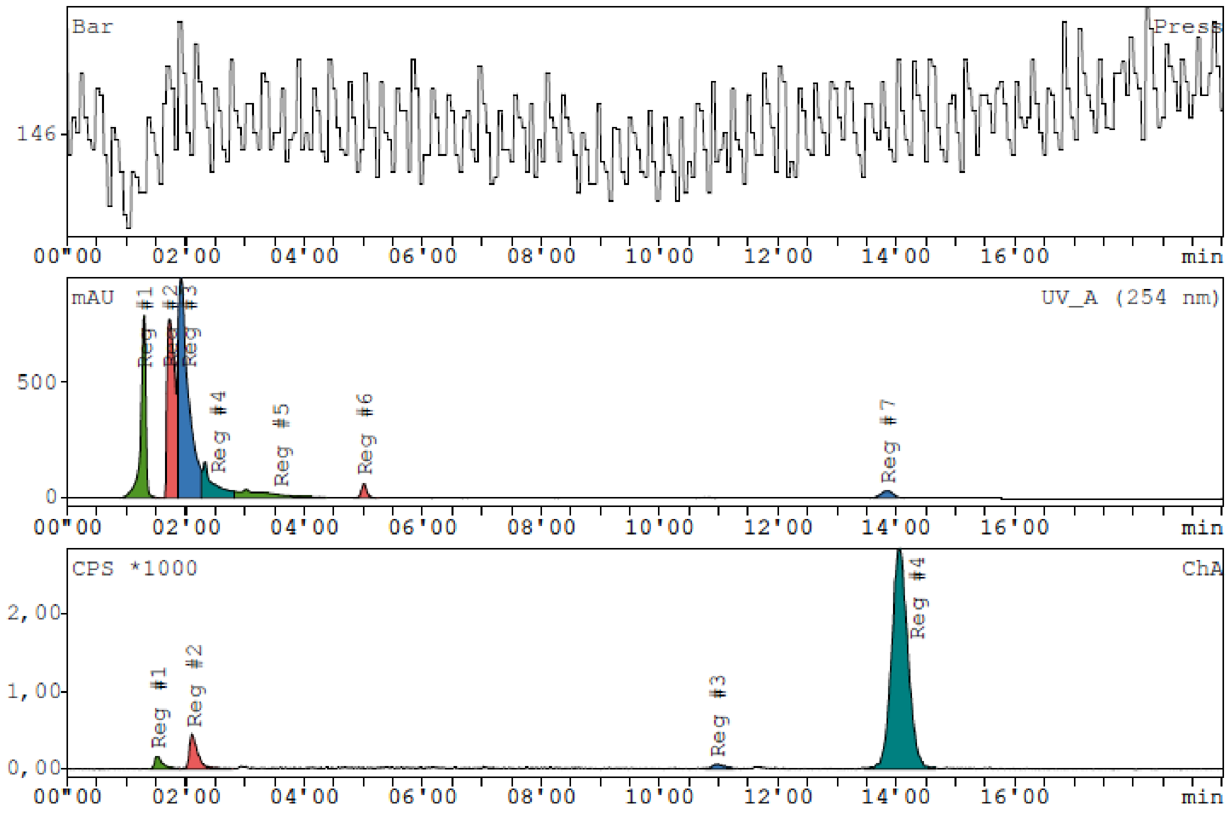

Sample description

Measurement:	220621-F, injection : 22.06.2021 14:06
Method:	LUNAPFP Mudasir AcN_H2O 50_50 from: 17.12.2020 09:30
CH3CN:H2O=50:50, Flow rate 1,5 mL/min, injection 10 micro L	
Radio detector: raytest Gabi Star	Serial Nr.: #30745 raytest GINA star 20.04.09 Firmware V4.8
Software Version: 5.9, Service Pack 8, Build 5076	

Integration ChA

Substance	R/T s	Type	Area Counts	%Area %
Reg #1	01'31	DD(M	1665,28	2,72
Reg #2	02'07	DD(M	4602,00	7,51
Reg #3	10'58	DD(M	946,07	1,54
Reg #4	14'04	DD(M	54034,63	88,22
Sum in ROI			61247,98	100,00
Area (total)			70628,17	
Ext. BKG			0,00 CPS	

Figure S143: Analytical HPLC chromatogram from screening, top channel = UV, bottom channel = activity. HPLC chromatogram of 1-(2,2-difluoro-2-(fluoro-^{18}F)ethyl)-4-(4-methoxyphenyl)piperazine **([^{18}F]7b)**. **[^{18}F]1b**, 1-(4-methoxyphenyl)piperazine, DMF, K_2CO_3 (2.3 eq.), KI (catalytic amount), 120°C, 15 min. 100 µL organic phase in 1 mL MeCN: H_2O =50:50. **(**MeCN: H_2O =50:50. Flow rate = 1.5 mL/min. Injected volume = 10 uL).

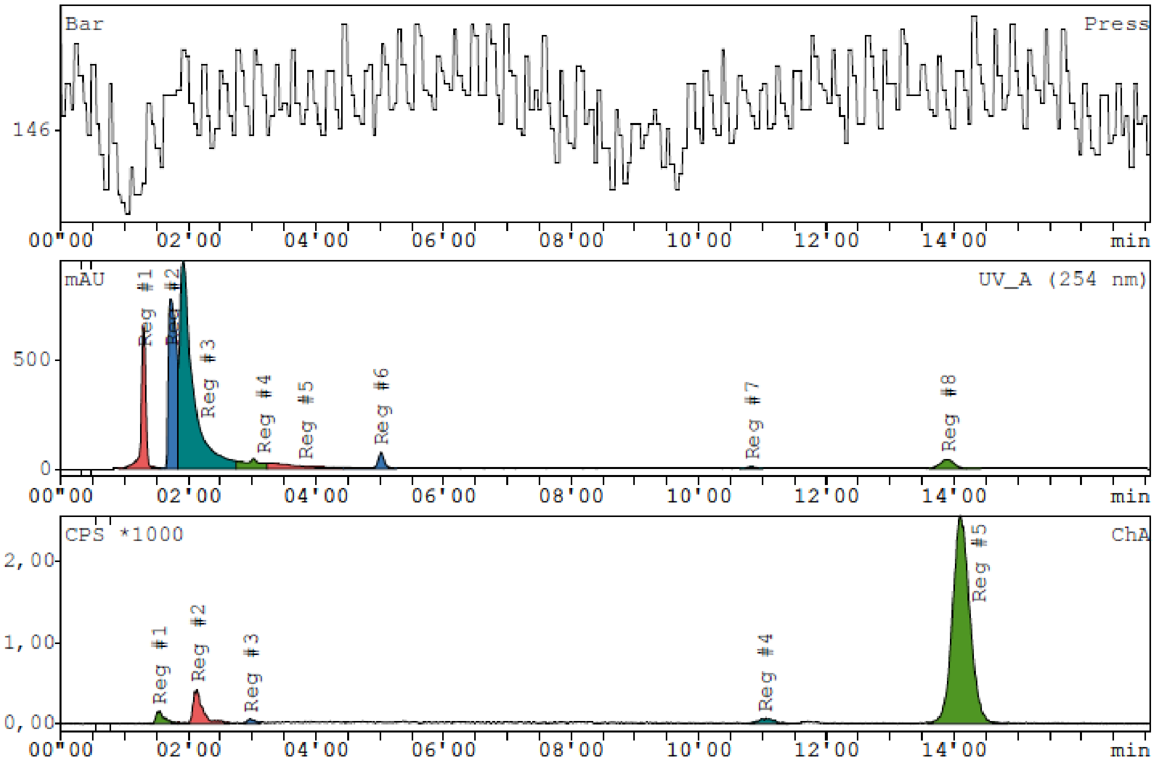

Sample description

Measurement:	220621-G, injection :	22.06.2021 14:28
Method:	LUNAPFP Mudasir AcN_H2O 50_50 from:	17.12.2020 09:30
CH3CN:H2O=50:50, Flow rate 1,5 mL/min, injection 10 micro L		
Radio detector: raytest Gabi Star	Serial Nr.: #30745 raytest GINA star 20.04.09 Firmware V4.8	
Software Version: 5.9, Service Pack 8, Build 5076		

Integration ChA

Substance	R/T s	Type	Area Counts	%Area %
Reg #1	01'32	DD(M	1364,92	2,48
Reg #2	02'08	DD(M	4363,73	7,94
Reg #3	02'58	DD(M	451,11	0,82
Reg #4	11'01	DD(M	1023,39	1,86
Reg #5	14'07	DD(M	47752,02	86,89
Sum in ROI			54955,17	100,00
Area (total)			62093,41	
BKG1			3,308	
Remainder			7138,24	11,50

Figure S144: Analytical HPLC chromatogram from screening, top channel = UV, bottom channel = activity. HPLC chromatogram of 1-(2,2-difluoro-2-(fluoro-^{18}F)ethyl)-4-(4-methoxyphenyl)piperazine **([^{18}F]7b)**. **[^{18}F]1b**, 1-(4-methoxyphenyl)piperazine, DMF, Cs_2CO_3 (2.3 eq.), KI (catalytic amount), 120°C, 15 min. 100 µL organic phase in 1 mL MeCN: H_2O =50:50. **(**MeCN: H_2O =50:50. Flow rate = 1.5 mL/min. Injected volume = 10 uL).

Measurement 220621-H Uni Oslo Page 1/2
c:\GINA_NT\LUNAPFP Mudasir AcN_H2O 50_50\220621-H

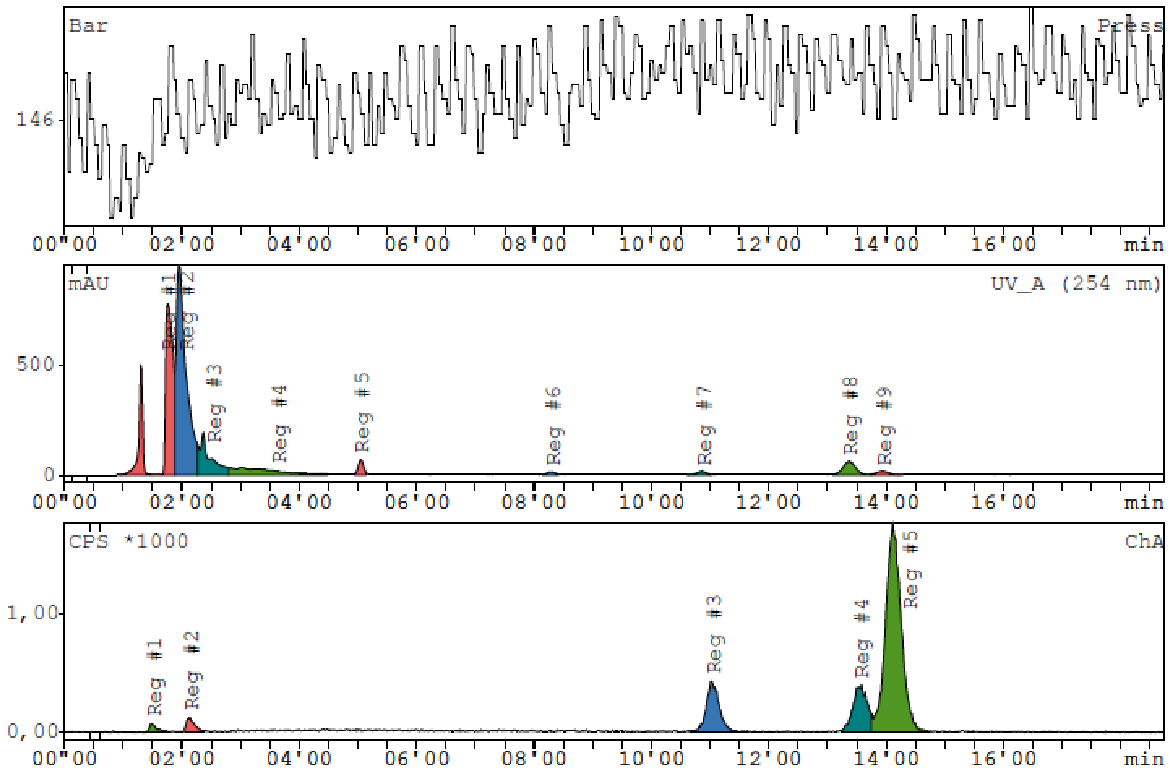

Sample description

Measurement:	220621-H, injection : 22.06.2021 14:46
Method:	LUNAPFP Mudasir AcN_H2O 50_50 from: 17.12.2020 09:30
CH3CN:H2O=50:50, Flow rate 1,5 mL/min, injection 10 micro L	
Radio detector: raytest Gabi Star	Serial Nr.: #30745 raytest GINA star 20.04.09 Firmware V4.8
Software Version: 5.9, Service Pack 8, Build 5076	

Integration ChA

Substance	R/T s	Type	Area Counts	%Area %
Reg #1	01'30	DD(M	564,50	1,20
Reg #2	02'08	DD(M	1141,71	2,44
Reg #3	11'03	DD(M	6462,66	13,79
Reg #4	13'33	DD(M	6456,66	13,78
Reg #5	14'08	DD(M	32224,80	68,78
Sum in ROI			46850,33	100,00
Area (total)			52825,90	
BKG1			4,273	
Remainder			5975,57	11,31

Figure S145: Analytical HPLC chromatogram from screening, top channel = UV, bottom channel = activity. HPLC chromatogram of 1-(2,2-difluoro-2-(fluoro-^{18}F)ethyl)-4-(4-methoxyphenyl)piperazine **([^{18}F]7b)**. **[^{18}F]1b**, 1-(4-methoxyphenyl)piperazine, DMF, K_2CO_3 (2.3 eq.), NaI (catalytic amount), 120°C, 15 min. 100 µL organic phase in 1 mL MeCN: H_2O =50:50. **(**MeCN: H_2O =50:50. Flow rate = 1.5 mL/min. Injected volume = 10 uL).

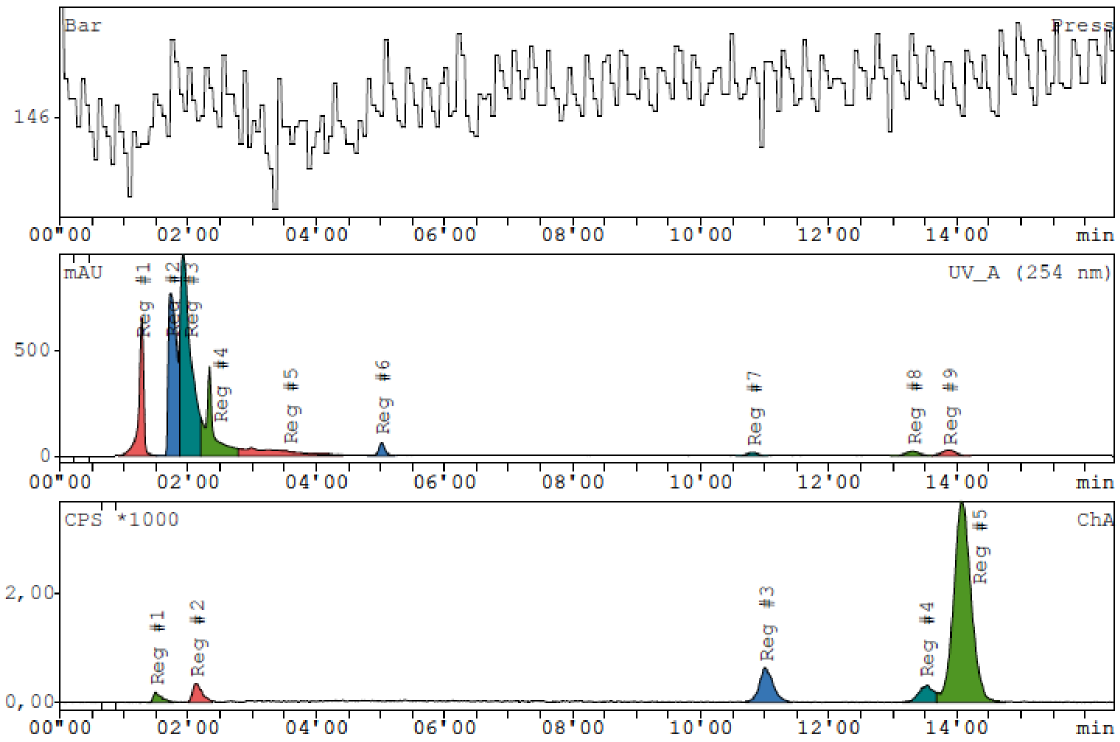

Sample description

Measurement:	220621-I, injection :	22.06.2021 15:07	
Method:	LUNAPFP Mudasir AcN_H2O 50_50 from:		17.12.2020 09:30
CH3CN:H2O=50:50, Flow rate 1,5 mL/min, injection 10 micro L			
Radio detector: raytest Gabi Star	Serial Nr.: #30745 raytest GINA star 20.04.09 Firmware V4.8		
Software Version: 5.9, Service Pack 8, Build 5076			

Integration ChA

Substance	R/T s	Type	Area Counts	%Area %
Reg #1	01'30	DD(M	1711,35	1,91
Reg #2	02'08	DD(M	3651,63	4,07
Reg #3	11'00	DD(M	9573,75	10,68
Reg #4	13'31	DD(M	4896,59	5,46
Reg #5	14'05	DD(M	69842,69	77,88
Sum in ROI			89676,01	100,00
Area (total)			101205,64	
BKG1			3,385	
Remainder			11529,63	11,39

Figure S146: Analytical HPLC chromatogram from screening, top channel = UV, bottom channel = activity. HPLC chromatogram of 1-(2,2-difluoro-2-(fluoro-^{18}F)ethyl)-4-(4-methoxyphenyl)piperazine **([^{18}F]7b)**. **[^{18}F]1b**, 1-(4-methoxyphenyl)piperazine, DMF, Cs_2CO_3 (2.3 eq.), NaI (catalytic amount), 120°C, 15 min. 100 µL organic phase in 1 mL MeCN: H_2O =50:50. **(**MeCN: H_2O =50:50. Flow rate = 1.5 mL/min. Injected volume = 10 uL).

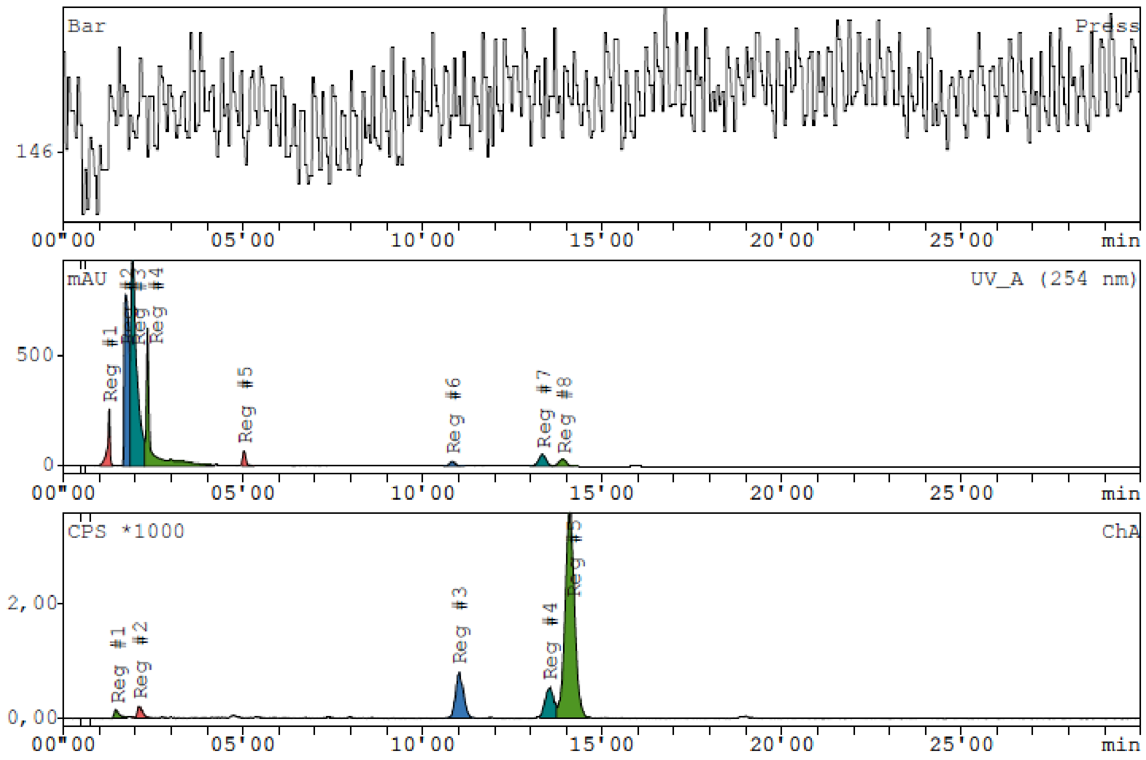

Sample description

Measurement:	220621-J, injection : 22.06.2021 15:24
Method:	LUNAPFP Mudasir AcN_H2O 50_50 from: 17.12.2020 09:30
CH3CN:H2O=50:50, Flow rate 1,5 mL/min, injection 10 micro L	
Radio detector: raytest Gabi Star	Serial Nr.: #30745 raytest GINA star 20.04.09 Firmware V4.8
Software Version: 5.9, Service Pack 8, Build 5076	

Integration ChA

Substance	R/T s	Type	Area Counts	%Area %
Reg #1	01'28	DD(M	1389,81	1,55
Reg #2	02'07	DD(M	2117,09	2,36
Reg #3	11'01	DD(M	11922,05	13,32
Reg #4	13'32	DD(M	8829,90	9,86
Reg #5	14'06	DD(M	65265,50	72,90
Sum in ROI			89524,36	100,00
Area (total)			92537,13	
BKG1			6,286	
Remainder			3012,78	3,26

Figure S147: Analytical HPLC chromatogram from screening, top channel = UV, bottom channel = activity. HPLC chromatogram of 1-(2,2-difluoro-2-(fluoro-^{18}F)ethyl)-4-(4-methoxyphenyl)piperazine **([^{18}F]7b)**. **[^{18}F]1b,** 1-(4-methoxyphenyl)piperazine, DMF, no base, 120°C, 15 min. 100 µL organic phase in 1 mL MeCN: H_2O =50:50. **(**MeCN: H_2O =50:50. Flow rate = 1.5 mL/min. Injected volume = 10 uL).
